# The temporal relationship between severe mental illness diagnosis and chronic physical comorbidity: a UK primary care cohort study of disease burden over 10 years

**DOI:** 10.1016/S2215-0366(22)00225-5

**Published:** 2022-09

**Authors:** Naomi Launders, Leiah Kirsh, David P J Osborn, Joseph F Hayes

**Affiliations:** a Division of Psychiatry, University College London, London, UK; b Camden and Islington NHS Foundation Trust, London, UK

## Abstract

**Background:**

Despite increased rates of physical health problems in people with schizophrenia, bipolar disorder, and other psychotic illnesses, the temporal relationship between physical disease acquisition and diagnosis of a severe mental illness remains unclear. We aimed to determine the cumulative prevalence of 24 chronic physical conditions in people with severe mental illness from 5 years before to 5 years after their diagnosis.

**Methods:**

In this cohort study, we used the UK Clinical Practice Research Datalink (CPRD) to identify patients aged 18–100 years who were diagnosed with severe mental illness between Jan 1, 2000, and Dec 31, 2018. Each patient with severe mental illness was matched with up to four individuals in the CPRD without severe mental illness by sex, 5-year age band, primary care practice, and year of primary care practice registration. Individuals in the matched cohort were assigned an index date equal to the date of severe mental illness diagnosis in the patient with severe mental illness to whom they were matched. Our primary outcome was the cumulative prevalence of 24 physical health conditions, based on the Charlson and Elixhauser comorbidity indices, at 5 years, 3 years, and 1 year before and after severe mental illness diagnosis and at the time of diagnosis. We used logistic regression to compare people with severe mental illness with the matched cohort, adjusting for key variables such as age, sex, and ethnicity.

**Findings:**

We identified 68 789 patients diagnosed with a severe mental illness between Jan 1, 2000, and Dec 31, 2018, and we matched them to 274 827 patients without a severe mental illness diagnosis. In both cohorts taken together, the median age was 40·90 years (IQR 29·46–56·00), 175 138 (50·97%) people were male, and 168 478 (49·03%) were female. The majority of patients were of White ethnicity (59 867 [87·03%] patients with a severe mental illness and 244 566 [88·99%] people in the matched cohort). The most prevalent conditions at the time of diagnosis in people with severe mental illness were asthma (10 581 [15·38%] of 68 789 patients), hypertension (8696 [12·64%]), diabetes (4897 [7·12%]), neurological disease (3484 [5·06%]), and hypothyroidism (2871 [4·17%]). At diagnosis, people with schizophrenia had increased odds of five of 24 chronic physical conditions compared with matched controls, and nine of 24 conditions were diagnosed less frequently than in matched controls. Individuals with bipolar disorder and other psychoses had increased odds of 15 conditions at diagnosis. At 5 years after severe mental illness diagnosis, these numbers had increased to 13 conditions for schizophrenia, 19 for bipolar disorder, and 16 for other psychoses.

**Interpretation:**

Elevated odds of multiple conditions at the point of severe mental illness diagnosis suggest that early intervention on physical health parameters is necessary to reduce morbidity and premature mortality. Some physical conditions might be under-recorded in patients with schizophrenia relative to patients with other severe mental illness subtypes.

**Funding:**

UK Office For Health Improvement and Disparities.

## Introduction

It is well established that individuals with severe mental illness, including schizophrenia, bipolar disorder, and other non-organic psychotic illness, have increased prevalences of a range of chronic physical health problems.^1^, ^2^ These health issues contribute to the reduced life expectancy of people with severe mental illness.^3^ The 2019 *Lancet Psychiatry* Commission on physical health in people with mental illness outlined the need to “focus not only on ‘adding years to life’ but also on ‘adding life to years’—specifically by preventing and reducing the incidence and impact of chronic health conditions”.^2^ To achieve this aim, it is necessary to understand the temporal relationship between severe mental illness and physical health diagnoses.

Knowledge of which physical conditions frequently develop before diagnosis of a severe mental illness and which are more common after diagnosis is crucial because it will provide information about the necessary timing of potential preventive or treatment interventions. For example, physical conditions that develop in advance of the severe mental illness diagnosis might be prodrome or lifestyle related, or might share common antecedents with severe mental illness. However, providing preventive interventions for these comorbidities is not possible unless they are delivered at a population level or in a service catering for patients at high risk or prodromal patients. Knowing which physical conditions are more prevalent at the point of severe mental illness diagnosis would potentially allow for screening and intervention, if suitable early-intervention services were developed.^4^ After diagnosis of a severe mental illness, physical health problems might be additionally related to the course of the illness itself, drug treatment for the illness, and the further accrual of lifestyle risk factors. Interventions aimed at modifiable lifestyle risk factors in people with established severe mental illness have been less effective than in the general population;^5^ however, this might be because a crucial period for intervention has been missed. Drug treatments for severe mental illness, including second-generation antipsychotics, antidepressants, lithium, and anticonvulsants, have adverse effects that are associated with multiple physical health conditions that might develop only after the diagnosis of a severe mental illness has been made and treatment has been initiated.^6^, ^7^ Patterns of these physical conditions by severe mental illness subtype might be different because of differences in genetic vulnerability, illness course, lifestyle risk factors, and drug treatment.

**Research in context**

**Evidence before this study**

*The* Lancet Psychiatry *Commission: a blueprint for protecting physical health in people with mental illness* produced a meta-review of all literature on the association between severe mental illness and chronic physical health conditions. The Commission identified 30 systematic reviews of physical illness in severe mental illness, 18 of which were on cardiometabolic disorders. It found increased risk of all cardiometabolic disorders and asthma, reduced risk of rheumatic disease, and mixed evidence on cancer risk in people with severe mental illness. The Commission also identified that the understanding of multimorbidity in severe mental illness is a knowledge gap. However, the studies included in the Commission did not generally examine the order of onset of severe mental illness and physical health problems. From a systematic search of PubMed, PsycINFO, and Embase for research articles published between Jan 1, 1980, and April 21, 2022, we could only identify a small number of studies that examined the temporal relationship between severe mental illness diagnosis and physical condition onset. We used the search terms “severe mental illness”, “serious mental illness”, “schizophrenia”, “schizoaffective disorder”, “bipolar disorder”, “psychosis”, and terms for 24 physical health outcomes, based on the Charlson and Elixhauser comorbidity indices: “asthma”, “chronic obstructive pulmonary disease”, “cardiac arrhythmia”, “congestive heart failure”, “myocardial infarction”, “cerebrovascular” disease, “neurological disorder”, “cancer”, “diabetes”, “hypothyroidism”, “liver disease”, “renal disease”, “peptic ulcer”, “rheumatic disease”, “paresis”, “paralysis”, “HIV”, “AIDS”, “hypertension”, “peripheral vascular disease”, “pulmonary circulation disorders”, “valvular disease”, “anaemia”, “coagulopathy”, “fluid disorders”, and “electrolyte disorders”. We also used search terms for “Charlson”, “Elixhauser”, “comorbidity”, and “multimorbidity”. No language restriction was applied. We identified two studies of the Taiwanese population that found increased risk of chronic conditions across the cardiovascular, respiratory, gastrointestinal, endocrine, and metabolic systems in the year before diagnosis of schizophrenia or bipolar disorder. A US cohort study found similar increases up to 5 years before schizophrenia diagnosis.

**Added value of this study**

This study describes the temporal relationship between first recorded diagnosis of schizophrenia, bipolar disorder, and other non-organic psychotic illness and 24 chronic health conditions from 5 years before to 5 years after severe mental illness diagnosis. 5 years before diagnosis, people with schizophrenia had increased odds of three conditions, people with bipolar disorder 12 conditions, and people with other psychoses ten conditions compared with the matched cohort. At diagnosis, people with schizophrenia had increased odds of five chronic health conditions compared with the matched cohort, and individuals with bipolar disorder and other psychoses had increased odds of 15 conditions. By 5 years after diagnosis of a severe mental illness, this number had increased to 13 conditions for schizophrenia, 19 for bipolar disorder, and 16 for other psychoses. Patients with schizophrenia frequently had lower odds of receiving a chronic physical condition diagnosis than the matched cohort, suggesting under-recording in this subgroup. At the date of receiving a schizophrenia diagnosis, cardiac arrhythmias, myocardial infarction, cancer, renal disease, peptic ulcer, rheumatic disease, hypertension, peripheral vascular disease, and valvular disease were recorded less frequently than would be expected. This study therefore highlights when intervention is necessary to tackle the excess morbidity observed in the patients with a severe mental illness.

**Implications of all the available evidence**

The mortality gap between people with severe mental illness and the general population has widened in the past 20 years, despite targeted attempts to address it. The elevated risk of several chronic physical conditions at the point of diagnosis of a severe mental illness (and the 5 years before it) suggests that preventive efforts are needed earlier in this population to have an impact on disease burden and mortality. Chronic health problems should not be viewed as the inevitable result of psychotropic medication and long-term health risk factors. We need to consider early intervention for physical health as well as mental health in this population.

Previous studies have focused on the period after the diagnosis of a severe mental illness^2^ or the year before severe mental illness diagnosis,^8^, ^9^ with only one study covering the 5 years before schizophrenia diagnosis.^10^ However, this study did not compare the prevalence of physical health conditions in people with schizophrenia with a group of people without schizophrenia, and it did not examine other severe mental illness subtypes. The vast majority of the existing literature examines cardiometabolic comorbidity.^2^ Furthermore, the onset and accumulation of multiple conditions in people with severe mental illness have not been described, yet multimorbidity carries an additional burden compared with comorbidity and is more complex to treat.

We aimed to determine the cumulative prevalence of 24 chronic physical conditions in people with schizophrenia, bipolar disorder, and other psychotic illness from 5 years before to 5 years after their severe mental illness diagnosis. We also examined the annual odds of receiving a new physical condition diagnosis in people with severe mental illness compared with those without.

## Methods

### Study design and participants

In this cohort study, we identified patients from the UK Clinical Practice Research Datalink (CPRD) Gold and Aurum^11^, ^12^ databases. These databases contain electronic medical records of more than 16 million people registered at UK primary care practices (approximately 24% of the UK population). The patients within the databases are broadly representative of the UK population in terms of age, sex, region, and ethnicity.

We identified patients in the CPRD with a first diagnosis of severe mental illness between Jan 1, 2000, and Dec 31, 2018, via medical codes for schizophrenia, bipolar disorder, or other non-affective psychotic illnesses, as recorded in primary care (code lists are in the appendix pp 5–157).^1^ These diagnoses will have been made by psychiatrists in secondary care using ICD criteria and communicated to primary care physicians. Where patients had more than one severe mental illness diagnosis during the study period, we took the most recent because this diagnosis was likely to represent the most accurate given a more complete clinical history. We retained the first date of any severe mental illness as the date of diagnosis. We excluded patients diagnosed before age 18 years, those older than 100 years at the beginning of the study, or those who were diagnosed after exiting the cohort. Matching was carried out by CPRD before we received the data from them. Each patient with severe mental illness was matched with up to four individuals in CPRD without severe mental illness, and individuals were matched by sex, 5-year age band, primary care practice, and year of primary care practice registration. These comparator individuals were assigned an index date equal to the date of diagnosis of the severe mental illness in the patient to whom they were matched.

Approval for this study was obtained from the Independent Scientific Advisory Committee of CPRD (protocol number 18_288). CPRD obtains annual research ethics approval from the UK Health Research Authority Research Ethics Committee (East Midlands—Derby Research Ethics Committee reference number 05/MRE04/87) to receive and supply patient data for public health research. Therefore, no additional ethics approval was required for this study.

### Procedures

We examined 24 physical health outcomes, which were based on the Charlson and Elixhauser comorbidity indices.^13^ These outcomes were asthma, chronic obstructive pulmonary disease, cardiac arrhythmia, congestive heart failure, myocardial infarction, cerebrovascular disease, neurological disorders (including epilepsy, multiple sclerosis, Parkinson's disease, and seizures, but excluding cerebrovascular disease and dementia), cancer, diabetes (type 1 or 2), hypothyroidism, liver disease, renal disease, peptic ulcers, rheumatic and collagen diseases, paralysis or paresis, HIV/AIDS, hypertension, peripheral vascular disease, pulmonary circulation disorders, valvular disease, deficiency anaemia, blood loss anaemia, coagulopathy, and fluid or electrolyte disorders. Code lists for these conditions are available in the appendix (pp 5–157).^1^

### Outcomes

Cumulative prevalence of these 24 chronic physical health comorbidities was examined cross-sectionally at 5 years, 3 years, and 1 year before severe mental illness diagnosis, at the point of diagnosis, and 1 year, 3 years, and 5 years after diagnosis. We also examined how the total number of these conditions changed for individuals over the same period.

### Statistical analysis

Covariates of interest included age at index date, sex, ethnicity, calendar year of index date, and geographical region of primary care practice. We grouped ethnicity as Asian, Black, mixed, White, or other. Where ethnicity data were missing, individuals were coded as White, in line with previous research.^1^, ^14^ Our previous work has shown multiple imputation of ethnicity results in similar physical health condition estimates.^1^

We calculated the prevalence of each chronic physical health comorbidity relative to the date of severe mental illness diagnosis at the timepoints already mentioned. We also calculated the total number of physical comorbidities people had at these timepoints. In each case, the denominator was the number of patients alive and available for follow-up at that timepoint. We used logistic regression to compare the prevalence of individual comorbidities in patients who had a severe mental illness with those who did not. Sandwich SEs were applied to account for potential clustering by primary care practice. We adjusted for key variables, determined a priori due to their likelihood to confound the relationship between severe mental illness and prevalence of disease: age, sex, ethnicity, index date calendar year, and primary care practice region. We then did a subgroup analysis to divide the severe mental illness cohort by severe mental illness diagnosis. These groups were schizophrenia, bipolar disorder, and other non-organic psychotic illness. We did not adjust for multiple comparisons.^15^ We conducted data analysis in Stata 16, R (version 4.1.2), and RStudio 2021.09.2.

### Role of the funding source

The funder of the study had no role in study design, data collection, data analysis, data interpretation, or writing of the report.

## Results

We identified 68 789 people who received a diagnosis of severe mental illness between Jan 1, 2000, and Dec 31, 2018 (including 15 034 [21·7%] with schizophrenia, 24 423 [35·5%] with bipolar disorder, and 29 332 [42·6%] with other non-organic psychoses) and 274 827 matched individuals (table 1). In both cohorts taken together, the median age was 40·90 years (IQR 29·46–56·00), 175 138 (50·97%) of 343 616 people were male, and 168 478 (49·03%) were female (table 1). When stratified by severe mental illness diagnosis, people with a diagnosis of schizophrenia were more likely than those with bipolar disorder to be male and younger (table 1). Patients with severe mental illness were more likely than those in the matched cohort to be obese or a current smoker at the time of diagnosis (table 1). 59 867 (87·03%) patients with a severe mental illness and 244 566 (88·99%) people in the matched cohort were of White ethnicity. Patients with schizophrenia were more likely to be of Black (1359 [9·04%] *vs* 8566 [3·12%]) or Asian (1030 [6·85%] *vs* 13 536 [4·93%]) ethnicity than comparators, whereas those with bipolar disorder were more likely to be of White ethnicity (22 443 [91·89%] *vs* 244 566 [88·99%]). Throughout the 10-year follow up, 7599 (11·05%) patients with severe mental illness and 19 385 (7·05%) comparators died.

**Table 1:** Patient characteristics

		**Severe mental illness cohort (n=68 789)**	**Schizophrenia (n=15 034)**	**Bipolar disorder (n=24 423)**	**Other psychoses (n=29 332)**	**Matched cohort (n=274 827)**
Sex						
	Male	35 051 (52·95%)	9691 (64·46%)	9973 (40·83%)	15 387 (52·46%)	140 087 (50·97%)
	Female	33 738 (49·05%)	5343 (35·54%)	14 450 (59·17%)	13 945 (47·54%)	134 740 (49·03%)
Age, years		40·92 (29·47–56·05)	38·15 (27·90– 51·37)	41·78 (31·17–54·70)	41·74 (29·00–60·95)	40·90 (29·46–56·00)
Ethnicity						
	Asian	3134 (4·56%)	1030 (6·85%)	749 (3·07%)	1355 (4·62%)	13 536 (4·93%)
	Black	3389 (4·93%)	1359 (9·04%)	486 (1·99%)	1544 (5·26%)	8566 (3·12%)
	Mixed	913 (1·33%)	277 (1·84%)	243 (0·99%)	393 (1·34%)	2271 (0·83%)
	White*	59 867 (87·03%)	12 037 (80·07%)	22 443 (91·89%)	25 387 (86·55%)	244 566 (88·99%)
	Other	1486 (2·16%)	331 (2·20%)	502 (2·06%)	653 (2·23%)	5888 (2·14%)
Died		7599 (11·05%)	1622 (10·79%)	2163 (8·86%)	3814 (13·00%)	19 385 (7·05%)
Body-mass index category at the index date†						
	Underweight	2449 (3·56%)	558 (3·71%)	634 (2·60%)	1257 (4·29%)	6042 (2·20%)
	Normal	22 492 (32·70%)	4289 (28·53%)	8062 (33·01%)	10 141 (34·57%)	88 782 (32·30%)
	Overweight	15 095 (21·94%)	2851 (18·96%)	5996 (24·55%)	6248 (21·30%)	64 338 (23·41%)
	Obese	11 490 (16·70%)	2344 (15·59%)	4892 (20·03%)	4254 (14·50%)	41 145 (14·97%)
	Missing	17 263 (25·10%)	4992 (33·20%)	4839 (19·81%)	7432 (25·34%)	74 520 (27·11%)
Smoking status at index						
	Current smoker	30 188 (43·88%)	6988 (46·48%)	10 655 (43·63%)	12 545 (42·77%)	80 123 (29·15%)
	Ex-smoker	12 242 (17·80%)	1783 (11·86%)	4970 (20·35%)	5489 (18·81%)	56 729 (20·64%)
	Never smoked	18 065 (26·26%)	3362 (22·36%)	6577 (26·93%)	8126 (27·70%)	99 824 (36·32%)
	Missing	8294 (12·06%)	2901 (19·30%)	2221 (9·09%)	3172 (10·81%)	38 151 (13·88%)
Age at death		75 (59–86)	68 (52– 81)	72 (59–82)	80 (62–88)	82 (72–89)
Follow-up after index date†		4·60 (2·39–8·71)	5·21 (2·64–9·93)	5·06 (2·63– 9·12)	4·01 (2·15–7·68)	4·58 (2·10–8·93)

Data are n (%) or median (IQR).

* 24 639 (35·82%) people in the cohort of people with severe mental illness and 119 048 (43·31%) people in the comparison cohort with missing ethnicity data were coded as White.

† The index date is equal to the date of diagnosis of the severe mental illness for a person with a severe mental illness or those individuals matched to that person.

**Figure 1:**
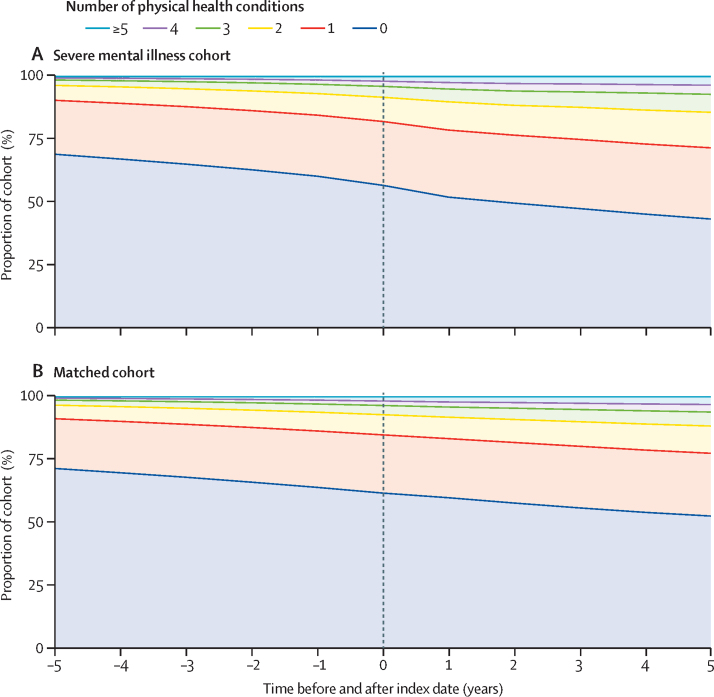
Number of physical health conditions in people with severe mental illness and matched controls The index date (indicated by the dashed line) is equal to the date of diagnosis of the severe mental illness for a person with a severe mental illness or those individuals matched to that person.

At the time of first recorded severe mental illness diagnosis, 29 812 (43·04%) people with a severe mental illness had at least one chronic physical health problem (compared with 105 247 [38·15%] matched comparators; figure 1). The most prevalent conditions were asthma (10 581 [15·38%] of 68 789 patients), hypertension (8696 [12·64%]), diabetes (4897 [7·12%]), neurological disease (3484 [5·06%]), and hypothyroidism (2871 [4·17%]; table 2). In the 38 285 patients with severe mental illness alive and available for follow-up at 5 years after that diagnosis, these prevalences had increased to 6920 (18·07%) for asthma, 6891 (18·00%) for hypertension, 5312 (13·87%) for diabetes, 2738 (7·15%) for neurological disease, and 2932 (7·66%) for hypothyroidism (table 2). At 5 years after diagnosis, 21 727 (56·75%) people with severe mental illness had one or more physical health condition (compared with 71 489 [47·42%] of 150 733 people in the matched cohort; figure 1; appendix p 1). The biggest increases in prevalence between diagnosis and 5 years after diagnosis were in renal disease (113·31% increase from 2046 [2·97%] of 68 789 people to 2429 [6·34%] of 38 285 people), fluid and electrolyte disorders (104·16% increase from 778 [1·13%] to 884 [2·31%]), diabetes (94·90% increase from 4897 [7·12%] to 5312 [13·87%]), hypothyroidism (83·49% increase from 2871 [4·17%] to 2932 [7·66%]), and chronic obstructive pulmonary disease (71·40% increase from 1563 [2·27%] to 1491 [3·89%]; table 2).

**Table 2:** Comorbidity prevalence in people with severe mental illness and matched cohort

	**5 years before diagnosis of severe mental illness**		**3 years before diagnosis of severe mental illness**		**1 year before diagnosis of severe mental illness**		Index date*		**1 year after diagnosis of severe mental illness**		**3 years after diagnosis of severe mental illness**		**5 years after diagnosis of severe mental illness**	
	Severe mental illness cohort (n=68 789)	Matched cohort (n=274 827)	Severe mental illness cohort (n=68 789)	Matched cohort (n=274 827)	Severe mental illness cohort (n=68 789)	Matched cohort (n=274 827)	Severe mental illness cohort (n=68 789)	Matched cohort (n=274 827)	Severe mental illness cohort (n=68 788)	Matched cohort (n=274 731)	Severe mental illness cohort (n=55 647)	Matched cohort (n=209 541)	Severe mental illness cohort (n=38 285)	Matched cohort (n=150 733)
Chronic obstructive pulmonary disease	684 (0·99%)	2346 (0·85%)	959 (1·39%)	3141 (1·14%)	1322 (1·92%)	4189 (1·52%)	1563 (2·27%)	4852 (1·77%)	1917 (2·79%)	5554 (2·02%)	1852 (3·33%)	5055 (2·41%)	1491 (3·89%)	4293 (2·85%)
Asthma	8444 (12·28%)	29 733 (10·82%)	9176 (13·34%)	32 206 (11·72%)	10 002 (14·54%)	34 993 (12·73%)	10 581 (15·38%)	36 497 (13·28%)	11 224 (16·32%)	37 551 (13·67%)	9642 (17·33%)	29 931 (14·28%)	6920 (18·07%)	21 875 (14·51%)
Cardiac arrythmia	894 (1·30%)	3636 (1·32%)	1150 (1·67%)	4753 (1·73%)	1535 (2·23%)	6154 (2·24%)	1832 (2·66%)	7020 (2·55%)	2188 (3·18%)	7924 (2·88%)	1940 (3·49%)	7137 (3·41%)	1339 (3·50%)	5889 (3·91%)
Congestive heart failure	340 (0·49%)	1432 (0·52%)	471 (0·68%)	1943 (0·71%)	666 (0·97%)	2659 (0·97%)	826 (1·20%)	3116 (1·13%)	1023 (1·49%)	3636 (1·32%)	921 (1·66%)	3167 (1·51%)	640 (1·67%)	2432 (1·61%)
Myocardial infarction	552 (0·80%)	2539 (0·92%)	666 (0·97%)	2987 (1·09%)	820 (1·19%)	3489 (1·27%)	930 (1·35%)	3816 (1·39%)	1047 (1·52%)	4143 (1·51%)	905 (1·63%)	3525 (1·68%)	644 (1·68%)	2837 (1·88%)
Cerebrovascular disease	990 (1·44%)	3456 (1·26%)	1293 (1·88%)	4442 (1·62%)	1715 (2·49%)	5643 (2·05%)	2105 (3·06%)	6358 (2·31%)	2509 (3·65%)	7121 (2·59%)	2283 (4·10%)	6089 (2·91%)	1669 (4·36%)	4788 (3·18%)
Neurological disease	2326 (3·38%)	4435 (1·61%)	2672 (3·88%)	4967 (1·81%)	3087 (4·49%)	5647 (2·05%)	3484 (5·06%)	6021 (2·19%)	4113 (5·98%)	6362 (2·32%)	3742 (6·72%)	5186 (2·47%)	2738 (7·15%)	3863 (2·56%)
Cancer	1696 (2·47%)	7437 (2·71%)	2040 (2·97%)	9091 (3·31%)	2511 (3·65%)	11 101 (4·04%)	2863 (4·16%)	12 423 (4·52%)	3246 (4·72%)	13 848 (5·04%)	2980 (5·36%)	12 398 (5·92%)	2225 (5·81%)	10 293 (6·83%)
Diabetes	2255 (3·28%)	8121 (2·95%)	2931 (4·26%)	10 413 (3·79%)	3994 (5·81%)	13 583 (4·94%)	4897 (7·12%)	15 572 (5·67%)	6259 (9·10%)	17 405 (6·34%)	6404 (11·51%)	16 110 (7·69%)	5312 (13·87%)	13 226 (8·77%)
Hypothyroidism	1611 (2·34%)	5348 (1·95%)	1975 (2·87%)	6535 (2·38%)	2483 (3·61%)	7905 (2·88%)	2871 (4·17%)	8707 (3·17%)	3556 (5·17%)	9394 (3·42%)	3545 (6·37%)	8512 (4·06%)	2932 (7·66%)	6989 (4·64%)
Liver disease	726 (1·06%)	2005 (0·73%)	871 (1·27%)	2327 (0·85%)	1087 (1·58%)	2711 (0·99%)	1223 (1·78%)	2959 (1·08%)	1413 (2·05%)	3148 (1·15%)	1257 (2·26%)	2544 (1·21%)	867 (2·26%)	1969 (1·31%)
Renal disease	767 (1·12%)	3157 (1·15%)	1144 (1·66%)	4724 (1·72%)	1665 (2·42%)	6728 (2·45%)	2046 (2·97%)	7975 (2·90%)	2574 (3·74%)	9305 (3·39%)	2745 (4·93%)	9203 (4·39%)	2429 (6·34%)	8276 (5·49%)
Peptic ulcer	1006 (1·46%)	3673 (1·34%)	1083 (1·57%)	3975 (1·45%)	1232 (1·79%)	4323 (1·57%)	1317 (1·91%)	4520 (1·64%)	1423 (2·07%)	4704 (1·71%)	1209 (2·17%)	3991 (1·90%)	854 (2·23%)	3086 (2·05%)
Rheumatic and collagen diseases	834 (1·21%)	3270 (1·19%)	990 (1·44%)	3880 (1·41%)	1159 (1·68%)	4581 (1·67%)	1290 (1·88%)	4983 (1·81%)	1400 (2·04%)	5336 (1·94%)	1215 (2·18%)	4745 (2·26%)	907 (2·37%)	3941 (2·61%)
Paralysis or paresis	360 (0·52%)	747 (0·27%)	385 (0·56%)	825 (0·30%)	417 (0·61%)	893 (0·32%)	452 (0·66%)	935 (0·34%)	491 (0·71%)	989 (0·36%)	442 (0·79%)	828 (0·40%)	306 (0·80%)	634 (0·42%)
HIV/AIDS	161 (0·23%)	415 (0·15%)	244 (0·35%)	602 (0·22%)	339 (0·49%)	889 (0·32%)	436 (0·63%)	1093 (0·40%)	540 (0·79%)	1272 (0·46%)	506 (0·91%)	1055 (0·50%)	348 (0·91%)	779 (0·52%)
Hypertension	5388 (7·83%)	24 638 (8·96%)	6510 (9·46%)	29 476 (10·73%)	7794 (11·33%)	35 044 (12·75%)	8696 (12·64%)	38 218 (13·91%)	9801 (14·25%)	40 882 (14·88%)	8884 (15·96%)	36 627 (17·48%)	6891 (18·00%)	30 010 (19·91%)
Peripheral vascular disease	393 (0·57%)	1645 (0·60%)	513 (0·75%)	2081 (0·76%)	677 (0·98%)	2626 (0·96%)	778 (1·13%)	2940 (1·07%)	902 (1·31%)	3295 (1·20%)	835 (1·50%)	2883 (1·38%)	593 (1·55%)	2419 (1·60%)
Pulmonary circulation disorders	243 (0·35%)	878 (0·32%)	307 (0·45%)	1029 (0·37%)	410 (0·60%)	1228 (0·45%)	486 (0·71%)	1378 (0·50%)	580 (0·84%)	1560 (0·57%)	571 (1·03%)	1384 (0·66%)	415 (1·08%)	1146 (0·76%)
Valvular disease	378 (0·55%)	1536 (0·56%)	458 (0·67%)	1888 (0·69%)	574 (0·83%)	2342 (0·85%)	643 (0·93%)	2598 (0·95%)	754 (1·10%)	2912 (1·06%)	647 (1·16%)	2620 (1·25%)	459 (1·20%)	2149 (1·43%)
Deficiency anaemia	1330 (1·93%)	4742 (1·73%)	1673 (2·43%)	5865 (2·13%)	2159 (3·14%)	7388 (2·69%)	2510 (3·65%)	8316 (3·03%)	2948 (4·29%)	9225 (3·36%)	2777 (4·99%)	8393 (4·01%)	2133 (5·57%)	6739 (4·47%)
Blood loss anaemia	14 (0·02%)	71 (0·03%)	18 (0·03%)	88 (0·03%)	29 (0·04%)	106 (0·04%)	31 (0·05%)	119 (0·04%)	34 (0·05%)	128 (0·05%)	27 (0·05%)	123 (0·06%)	21 (0·05%)	121 (0·08%)
Coagulopathy	199 (0·29%)	681 (0·25%)	232 (0·34%)	830 (0·30%)	270 (0·39%)	1006 (0·37%)	308 (0·45%)	1111 (0·40%)	372 (0·54%)	1218 (0·44%)	359 (0·65%)	1072 (0·51%)	293 (0·77%)	859 (0·57%)
Fluid or electrolyte disorders	261 (0·38%)	858 (0·31%)	370 (0·54%)	1224 (0·45%)	574 (0·83%)	1716 (0·62%)	778 (1·13%)	2067 (0·75%)	1081 (1·57%)	2473 (0·90%)	1105 (1·99%)	2343 (1·12%)	884 (2·31%)	2010 (1·33%)

Data are n (%).

* The index date is equal to the date of diagnosis of the severe mental illness for a person with a severe mental illness or those individuals matched to that person.

**Figure 2:**
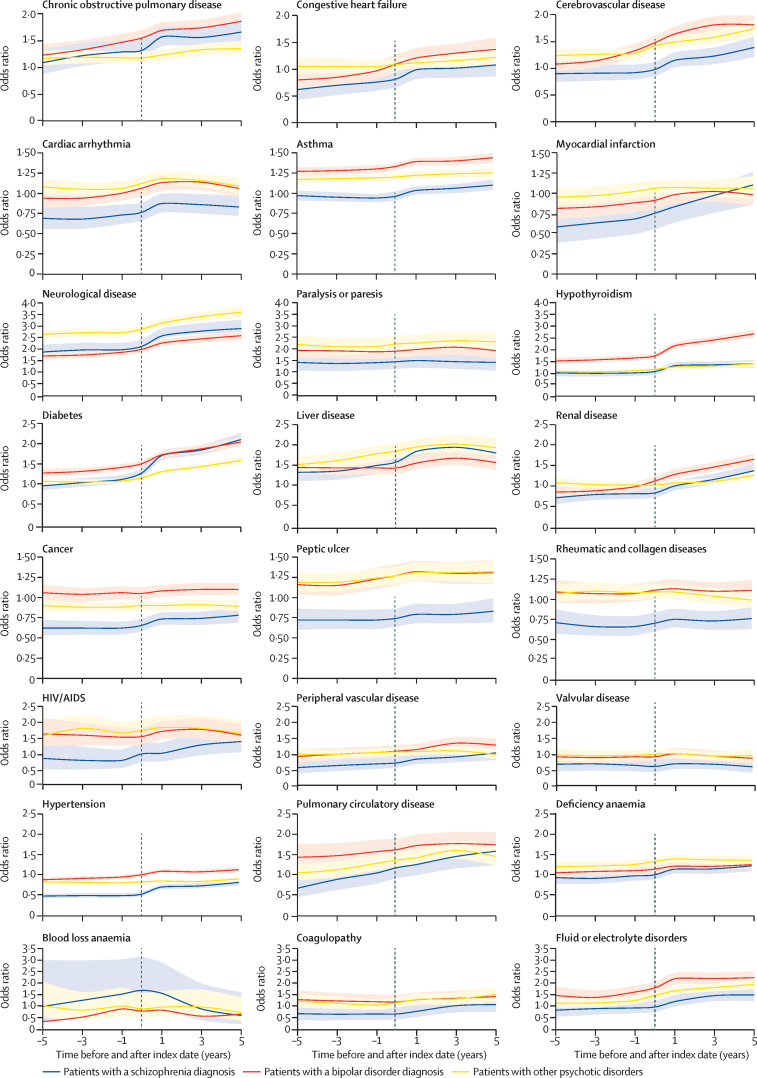
Odds ratios for 24 chronic physical health conditions in people with severe mental illness from 5 years before to 5 years after diagnosis of a severe mental illness The index date (indicated by the dashed lines) is equal to the date of diagnosis of the severe mental illness or those individuals matched to that person. Shaded areas indicate 95% CIs.

At the time of schizophrenia diagnosis, patients had elevated odds of five of 24 physical health conditions compared with matched individuals (figure 2; appendix p 2). Nine of 24 physical conditions were diagnosed less frequently in patients with schizophrenia than in matched individuals at this timepoint. At 5 years after diagnosis, individuals with schizophrenia had elevated odds of 13 conditions. Conditions with most elevated odds ratios by 5 years after schizophrenia diagnosis were neurological disease (adjusted odds ratio 2·89, 95% CI 2·63–3·16), diabetes (2·10, 1·96–2·26), liver disease (1·80, 1·55–2·10), chronic obstructive pulmonary disease (1·66, 1·46–1·89), and pulmonary circulation disorders (1·58, 1·23–2·03).

Individuals with bipolar disorder had increased odds of 15 of the 24 chronic conditions at the point of bipolar disorder diagnosis (figure 2; appendix pp 2–4). Unlike patients with schizophrenia, they did not have reduced odds of any condition compared to the comparator population. At 5 years after bipolar disorder diagnosis, they had elevated odds of 19 chronic conditions. The most elevated odds at this time were hypothyroidism (adjusted OR 2·68, 95% CI 2·52–2·85), neurological disease (2·58, 2·39–2·79), fluid and electrolyte conditions (2·29, 2·04–2·58), diabetes (2·04, 1·93–2·15), and paralysis or paresis (1·92, 1·58–2·33).

Individuals in the other psychosis group had elevated odds of 15 physical conditions and reduced odds of two conditions at the point of diagnosis (figure 2; appendix pp 2–4). At 5 years after diagnosis, they had elevated odds of 16 conditions. At this timepoint, neurological disease (adjusted OR 3·60, 95% CI 3·36–3·86), paralysis or paresis (2·30, 1·91–2·77), fluid and electrolyte conditions (1·99, 1·78–2·22), liver disease (1·93, 1·72–2·17), and cerebrovascular disease (1·74, 1·60–1·90) were the conditions with the most elevated odds in people with other psychotic illness compared with the comparator population.

## Discussion

People with severe mental illness are at an elevated risk of a range of chronic physical health problems. These conditions differ by severe mental illness subtype and by timing relative to the diagnosis. By the point of a diagnosis being recorded, people with bipolar disorder and other psychotic illness have elevated odds of more than half of the chronic conditions that we studied, compared with matched individuals. In the population with schizophrenia, odds were less commonly elevated compared with the matched cohort and were reduced for several chronic conditions. Therefore, it is likely that these conditions—namely, cardiac arrhythmias, myocardial infarction, cancer, renal disease, peptic ulcer, rheumatic disease, hypertension, peripheral vascular disease, and valvular disease—are under-diagnosed at the point of schizophrenia diagnosis. This is despite evidence that health-care use in the 5 years before a diagnosis of schizophrenia is increased^10^ and that people with schizophrenia have more lifestyle-related risk factors.^1^ There is no biological mechanism by which schizophrenia would be protective for these conditions (although this has previously been suggested for cancer).^16^ This discrepancy might therefore be driven by under-investigation or diagnostic overshadowing, which is more common in this severe mental illness subtype.^17^

Some of the chronic conditions with elevated odds ratios at the point of severe mental illness diagnosis are potentially amenable to intervention. In the UK, the National Institute for Health and Care Excellence recommends monitoring of cardiovascular disease, diabetes, obesity, and respiratory disease.^18^ However, it does not make specific recommendations about health monitoring in early-intervention services, and data suggest that at the end of 2021 only about 35% of people on the UK primary care severe mental illness register received annual health checks.^19^ Services that target this patient population at an early stage might be necessary to reduce the premature morbidity and mortality observed.^20^ Importantly, UK initiatives monitored by the National Clinical Audit of Psychosis have overall found modest improvements in the health of individuals under the care of early-intervention services in the past 10 years.^21^ Our findings suggest that a broader scope of interventions to improve physical health might be necessary.

We found that several conditions that are commonly associated with the accrual of risk factors over long time periods were more frequent at the point of severe mental illness diagnosis. Chronic obstructive pulmonary disease, which is strongly associated with a greater than 20 pack-year smoking history,^22^ was associated with increased odds at diagnosis in all subtypes of severe mental illness. Diabetes, which is typically diagnosed 4–7 years after its onset,^23^ was present in 9% of the severe mental illness population. Increased rates of liver disease, particularly metabolic dysfunction-associated fatty liver disease, have previously been attributed to the adverse effects of psychotropic medication,^24^ but in our study, relative odds of liver disease were elevated before severe mental illness diagnosis.

Several conditions were relatively more common in the severe mental illness group even 5 years before severe mental illness diagnosis. These included asthma (bipolar disorder and other psychoses only), neurological conditions (all severe mental illnesses), diabetes (bipolar disorder only), hypothyroidism (bipolar disorder only), liver disease (all severe mental illnesses), peptic ulcer (bipolar disorder and other psychoses), paralysis (all severe mental illness), HIV (bipolar disorder and other psychoses), pulmonary circulation disorders (bipolar disorder only), deficiency anaemia (other psychoses only), and fluid or electrolyte disorders (bipolar disorder only). These findings suggest that several physical health problems develop long before severe mental illness diagnosis and are not, at that time, solely related to psychotropic medication. Some of these conditions are associated with increased risk of developing severe mental illness; for example, there is good evidence that neurological conditions,^25^ and some evidence that asthma and hypothyroidism,^26^, ^27^ are associated with later severe mental illness. There is also evidence for a familial liability for these conditions.^26^, ^27^, ^28^ The increased prevalences of some conditions could be due to risk factors for poor physical health, such as inactivity, obesity, and smoking being present before diagnosis. Several studies have found elevated prevalence of smoking in the prodromal period and before this period.^29^ Another study found that people with a first-episode psychosis were more than eight times as likely to have a history of substance misuse in the 5 years before than matched controls.^30^ In our study, even at the time of diagnosis, the prevalence of both obesity and smoking was higher in people with severe mental illness than in matched individuals.

Some chronic conditions became increasingly common in the severe mental illness population compared with the matched cohort over the 10-year period of follow-up. These conditions include chronic obstructive pulmonary disease, asthma, congestive heart failure, cerebrovascular disease, neurological disease, diabetes, hypothyroidism, renal disease, pulmonary circulation disorders, hypertension, deficiency anaemias, and fluid or electrolyte disorders. This might be due to increased monitoring in the severe mental illness population, increasing effects of risk factors, or adverse effects of medication. There were particular increases in a number of conditions associated with medication side-effects after severe mental illness diagnosis: diabetes, hypothyroidism, renal disease, and hypertension.^7^, ^31^

To our knowledge, this is the first large-scale longitudinal study of multiple physical health conditions during the 5 years before and after severe mental illness diagnosis. The cohort is likely to be representative of the UK population with severe mental illness, and the size allowed us to study rare physical health conditions. We were able to compare patients who had severe mental illness with individuals from the same primary care practice without severe mental illness, matched by age, sex, and year of registration. We also adjusted for these covariates in regression models. This improved relative estimates of odds of physical health problems in severe mental illness compared with using the unmatched general population. We did not attempt to investigate the cause of the increased prevalence of physical health conditions in people with severe mental illness and therefore did not account for factors such as smoking or body-mass index, which are potentially on the causal pathway for many of these conditions. The comparator population is likely to be less healthy than the general population, and this is reflected in their high rate of morbidity and mortality (at the index date, 38% had one or more chronic physical conditions and 7% died during follow-up). Our results therefore accurately reflect the additional health inequalities experienced by people with severe mental illness at times relative to their severe mental illness diagnosis.

The age at which a severe mental illness diagnosis was first entered in primary care records in our cohort was older than expected, given the established epidemiology of severe mental illness. In our cohort, the median age of first coding was 38 years for schizophrenia and 42 years for bipolar disorder and other psychoses, whereas other UK and Nordic studies have found 30 years to be the median age of first contact with services.^32^, ^33^ This age discrepancy suggests that there might have been delays in recording severe mental illness, and there might therefore similarly have been delays in recording physical health conditions in primary care records. It is also possible that there is differential diagnosis of physical health conditions by severe mental illness subtype, which reflects the complexity and illness burden of the condition and the potential for diagnostic overshadowing or under-investigation. For example, it has been found in numerous studies that schizophrenia is associated with lower rates of cancer diagnosis than seen in the general population, despite the elevated cancer risk factors being present in people with schizophrenia.^16^ This could mean we underestimate the true prevalence and incorrectly label the start date of the conditions studied. However, health conditions will be recorded in primary care records once identified and must be coded for appropriate intervention to take place. Our study therefore describes the opportunity to intervene once conditions have been diagnosed.

There is the potential for residual or unmeasured confounding in the adjusted ORs we present; in particular, there might be confounding by socioeconomic status. Individual-level socioeconomic status is not available in CPRD, and area-level deprivation measures are only available for some of the population. We sought to overcome this limitation by matching patients within the same primary care practice. There is also the potential for under-recording of diagnostic information in the primary care record if a diagnosis is made in secondary care, and this might vary by condition; however, we would not expect this to vary between the severe mental illness and non-severe mental illness groups. Recording might also vary by primary care practice and year. We have managed this variability by accounting for these parameters in our analysis. We did not stratify results by other variables because this was outside the scope of this analysis, given the large number of conditions studied. It is likely that rates of physical disease differ by sex, age, and ethnicity, and further research to identify people at highest risk of specific health conditions within the severe mental illness population is warranted. We did not adjust for multiple comparisons because we considered our study descriptive in nature and believed that this approach would lead to fewer errors of interpretation when the data under evaluation were not random numbers but actual observations.^15^

If we are to positively affect the incidence and disability burden of chronic physical health problems in people with severe mental illness, interventions need to start early. Chronic physical health problems should not be viewed as the inevitable result of psychotropic medication's adverse effects and long-term health risk factors such as poor diet, smoking, or drug or alcohol misuse, because many of these conditions are present at the point of severe mental illness diagnosis first being recorded. Potentially, interventions targeted at improving the physical health of people with severe mental illness have been initiated too late relative to disease progression, and we need to consider early intervention for physical health as well as mental health in this population.

## Data sharing

Electronic health records are, by definition, considered to be sensitive data in the UK by the Data Protection Act and cannot be shared via public deposition because of information governance restriction in place to protect patient confidentiality. Access to data is available only once approval has been obtained through the individual constituent entities controlling access to the data. The primary care data can be requested via application to the Clinical Practice Research Datalink.

## Declaration of interests

JFH has received consultancy fees from the Wellcome Trust and juli Health. All other authors declare no competing interests.
