# Supplementary material for: The temporal relationship between severe mental illness diagnosis and chronic physical comorbidity: a UK primary care cohort study of disease burden over 10 years
Source: Lancet Psychiatry. 2022 Sep;9(9):725–35. doi: 10.1016/S2215-0366(22)00225-5 (PMC9630158; doi:10.1016/S2215-0366(22)00225-5)
Supplement: Supplementary appendix [file mmc1.pdf]

THE LANCET
Psychiatry

Supplementary appendix

This appendix formed part of the original submission and has been peer reviewed. We post it as supplied by the authors.

Supplement to: Launders N, Kirsh L, Osborn DPJ, Hayes JF. The temporal relationship between severe mental illness diagnosis and chronic physical comorbidity: a UK primary care cohort study of disease burden over 10 years. *Lancet Psychiatry* 2022; published online July 21. https://doi.org/10.1016/S2215-0366(22)00225-5.

***Supplement Table 1**. Count of physical health conditions in the 5 years before severe mental illness diagnosis, and the five years after*

**SMI**	Prevalence (% of active cohort)										
	**Years before severe mental illness diagnosis**						**Years after severe mental illness diagnosis**				
Number of physical conditions	**-5**	**-4**	**-3**	**-2**	**-1**	**Index**	**+1**	**+2**	**+3**	**+4**	**+5**
0	69.03	67.12	65.09	62.85	60.29	56.66	51.96	49.57	47.41	45.20	43.25
1	21.47	22.18	22.90	23.55	24.31	25.42	26.70	27.06	27.53	27.91	28.34
2	5.95	6.55	7.12	7.84	8.59	9.64	11.25	11.88	12.79	13.52	14.14
3	2.15	2.44	2.82	3.24	3.69	4.35	5.09	5.69	6.11	6.78	7.16
4	0.83	0.98	1.17	1.41	1.71	2.05	2.58	2.97	3.18	3.35	3.64
5	0.38	0.44	0.53	0.62	0.78	1.01	1.21	1.39	1.51	1.69	1.84
6	0.13	0.18	0.23	0.29	0.35	0.48	0.66	0.77	0.76	0.83	0.89
7	0.05	0.07	0.09	0.14	0.17	0.22	0.31	0.35	0.38	0.38	0.40
8	0.01	0.02	0.03	0.04	0.07	0.10	0.14	0.19	0.18	0.16	0.18
9	0.01	0.01	0.01	0.02	0.02	0.04	0.05	0.05	0.07	0.09	0.09
10	<0.01	<0.01	0.01	0.01	0.01	0.02	0.02	0.03	0.03	0.03	0.01
11		<0.01	<0.01	<0.01	<0.01	0.01	0.02	0.02	0.02	0.02	0.02
12			<0.01	<0.01	0.01	0.01	0.01	0.01	0.01	0.02	0.03
13					<0.01	<0.01	<0.01	<0.01	<0.01	<0.01	<0.01
14						<0.01	<0.01	0.01	0.01	<0.01	0.01
15								<0.01	<0.01	<0.01	<0.01
>15							0.01	0.01	0.01		<0.01

**Comparators**							Prevalence (% of active cohort)				
Number of physical conditions	**-5**	**-4**	**-3**	**-2**	**-1**	**Index**	**+1**	**+2**	**+3**	**+4**	**+5**
0	71.50	69.79	68.00	66.04	63.97	61.70	59.86	57.75	55.80	54.03	52.57
1	19.81	20.47	21.08	21.79	22.45	23.17	23.52	24.11	24.54	24.80	24.98
2	5.41	5.87	6.40	6.91	7.47	8.04	8.54	9.16	9.78	10.37	10.86
3	2.01	2.30	2.58	2.92	3.26	3.66	4.04	4.48	4.86	5.25	5.56
4	0.78	0.95	1.12	1.30	1.54	1.78	2.02	2.26	2.48	2.73	2.99
5	0.32	0.40	0.50	0.63	0.73	0.90	1.07	1.15	1.31	1.44	1.55
6	0.11	0.15	0.20	0.25	0.34	0.43	0.53	0.62	0.68	0.74	0.77
7	0.04	0.06	0.08	0.10	0.12	0.17	0.24	0.27	0.32	0.36	0.40
8	0.01	0.02	0.03	0.04	0.07	0.08	0.11	0.13	0.14	0.17	0.20
9	<0.01	0.01	0.01	0.01	0.02	0.03	0.04	0.05	0.05	0.06	0.08
10	<0.01	<0.01	<0.01	0.01	0.01	0.01	0.02	0.02	0.03	0.03	0.03
11		<0.01	<0.01	<0.01	<0.01	<0.01	0.01	0.01	0.01	0.01	0.01
12				<0.01	<0.01	<0.01	<0.01	<0.01	<0.01	<0.01	<0.01
13									<0.01	<0.01	<0.01
14						<0.01	<0.01	<0.01	<0.01		
15											<0.01
>15											

Supplement Table 2. *Adjusted* odds ratios (with 95% CI based on sandwich standard errors) for physical health diagnoses in people with severe mental illness compared to matched comparators*

	Adjusted odds ratios (95% confidence interval)						
	Years before severe mental illness diagnosis				Years after severe mental illness diagnosis		
	**-5**	**-3**	**-1**	**Index**	**+1**	**+3**	**+5**
**COPD**							
All SMI	1.17 (1.07-1.28)	1.23 (1.14-1.33)	1.28 (1.20-1.37)	1.31 (1.24-1.40)	1.42 (1.34-1.50)	1.50 (1.42-1.59)	1.60 (1.50-1.70)
Schizophrenia	1.09 (0.88-1.34)	1.22 (1.03-1.45)	1.29 (1.11-1.50)	1.32 (1.16-1.51)	1.57 (1.40-1.75)	1.56 (1.40-1.75)	1.66 (1.46-1.89)
Bipolar disorder	1.23 (1.06-1.43)	1.33 (1.17-1.51)	1.47 (1.33-1.63)	1.55 (1.41-1.70)	1.69 (1.55-1.84)	1.74 (1.60-1.89)	1.86 (1.70-2.03)
Other	1.17 (1.04-1.31)	1.19 (1.08-1.32)	1.18 (1.08-1.29)	1.18 (1.09-1.29)	1.23 (1.13-1.33)	1.33 (1.22-1.44)	1.35 (1.23-1.49)
**Asthma**							
All SMI	1.16 (1.13-1.20)	1.17 (1.14-1.20)	1.18 (1.15-1.21)	1.20 (1.17-1.23)	1.24 (1.21-1.27)	1.26 (1.22-1.29)	1.29 (1.25-1.33)
Schizophrenia	0.97 (0.91-1.03)	0.95 (0.90-1.01)	0.94 (0.89-0.99)	0.96 (0.91-1.02)	1.03 (0.98-1.08)	1.06 (1.00-1.11)	1.10 (1.04-1.17)
Bipolar disorder	1.27 (1.22-1.32)	1.28 (1.23-1.33)	1.30 (1.25-1.35)	1.33 (1.28-1.37)	1.39 (1.34-1.43)	1.40 (1.35-1.45)	1.44 (1.37-1.50)
Other	1.17 (1.13-1.22)	1.18 (1.14-1.22)	1.19 (1.15-1.23)	1.20 (1.16-1.25)	1.22 (1.18-1.27)	1.24 (1.19-1.29)	1.25 (1.20-1.31)
**Cardiac Arrythmia**							
All SMI	0.98 (0.91-1.06)	0.96 (0.90-1.03)	0.99 (0.93-1.06)	1.05 (0.99-1.11)	1.12 (1.06-1.18)	1.10 (1.04-1.16)	1.03 (0.97-1.10)
Schizophrenia	0.69 (0.56-0.85)	0.68 (0.56-0.83)	0.73 (0.62-0.86)	0.76 (0.66-0.88)	0.87 (0.76-0.99)	0.86 (0.75-0.99)	0.83 (0.72-0.97)
Bipolar disorder	0.94 (0.82-1.08)	0.94 (0.84-1.06)	1.00 (0.91-1.11)	1.06 (0.96-1.17)	1.13 (1.03-1.23)	1.14 (1.04-1.24)	1.06 (0.96-1.17)
Other	1.08 (0.98-1.18)	1.05 (0.97-1.14)	1.06 (0.99-1.15)	1.12 (1.05-1.20)	1.18 (1.11-1.26)	1.16 (1.08-1.24)	1.09 (1.00-1.19)
**Congestive Heart Failure**							
All SMI	0.93 (0.82-1.05)	0.95 (0.86-1.06)	0.99 (0.90-1.08)	1.05 (0.97-1.14)	1.12 (1.04-1.21)	1.18 (1.08-1.27)	1.25 (1.13-1.38)
Schizophrenia	0.62 (0.43-0.88)	0.70 (0.52-0.95)	0.76 (0.60-0.97)	0.82 (0.67-1.02)	0.99 (0.82-1.18)	1.02 (0.84-1.22)	1.08 (0.87-1.34)
Bipolar disorder	0.80 (0.63-1.03)	0.85 (0.69-1.03)	0.97 (0.82-1.14)	1.10 (0.95-1.27)	1.21 (1.06-1.39)	1.30 (1.14-1.48)	1.37 (1.19-1.59)
Other	1.05 (0.91-1.22)	1.05 (0.93-1.20)	1.05 (0.94-1.16)	1.09 (0.98-1.20)	1.12 (1.02-1.22)	1.16 (1.05-1.29)	1.22 (1.07-1.40)
**MI**							
All SMI	0.85 (0.78-0.94)	0.88 (0.80-0.96)	0.93 (0.86-1.00)	0.96 (0.90-1.04)	1.00 (0.93-1.08)	1.03 (0.95-1.12)	1.04 (0.95-1.14)
Schizophrenia	0.58 (0.45-0.75)	0.63 (0.50-0.79)	0.68 (0.56-0.83)	0.75 (0.62-0.89)	0.83 (0.70-0.98)	0.97 (0.82-1.15)	1.10 (0.92-1.33)
Bipolar disorder	0.81 (0.68-0.97)	0.83 (0.71-0.97)	0.88 (0.77-1.02)	0.91 (0.80-1.04)	0.98 (0.87-1.12)	1.02 (0.89-1.16)	0.98 (0.85-1.14)
Other	0.95 (0.85-1.07)	0.97 (0.88-1.08)	1.02 (0.93-1.13)	1.06 (0.96-1.16)	1.07 (0.97-1.17)	1.06 (0.95-1.17)	1.06 (0.93-1.21)
**Cerebrovascular Disease**							
All SMI	1.15 (1.06-1.24)	1.17 (1.09-1.25)	1.23 (1.16-1.31)	1.36 (1.29-1.44)	1.47 (1.40-1.55)	1.59 (1.51-1.68)	1.70 (1.60-1.81)
Schizophrenia	0.90 (0.75-1.09)	0.91 (0.77-1.07)	0.92 (0.80-1.07)	0.98 (0.86-1.12)	1.15 (1.02-1.30)	1.23 (1.08-1.39)	1.39 (1.21-1.59)
Bipolar disorder	1.08 (0.95-1.24)	1.14 (1.02-1.28)	1.33 (1.21-1.46)	1.48 (1.36-1.61)	1.64 (1.51-1.78)	1.81 (1.67-1.97)	1.81 (1.65-1.98)
Other	1.24 (1.13-1.36)	1.26 (1.16-1.36)	1.28 (1.18-1.37)	1.42 (1.33-1.52)	1.49 (1.39-1.59)	1.58 (1.47-1.70)	1.74 (1.60-1.90)
**Neurological Disease**							
All SMI	2.15 (2.05-2.26)	2.21 (2.11-2.32)	2.26 (2.16-2.36)	2.40 (2.30-2.51)	2.71 (2.60-2.82)	2.92 (2.79-3.05)	3.04 (2.89-3.21)
Schizophrenia	1.87 (1.68-2.08)	1.96 (1.77-2.16)	1.97 (1.79-2.16)	2.12 (1.94-2.32)	2.57 (2.37-2.79)	2.78 (2.56-3.02)	2.89 (2.63-3.16)
Bipolar disorder	1.70 (1.57-1.84)	1.74 (1.61-1.88)	1.86 (1.73-2.00)	1.99 (1.85-2.13)	2.26 (2.12-2.41)	2.44 (2.28-2.61)	2.58 (2.39-2.79)
Other	2.64 (2.49-2.81)	2.71 (2.55-2.88)	2.71 (2.56-2.87)	2.86 (2.71-3.02)	3.13 (2.97-3.30)	3.41 (3.23-3.61)	3.60 (3.36-3.86)
**Cancer**							
All SMI	0.90 (0.85-0.95)	0.88 (0.84-0.93)	0.89 (0.85-0.93)	0.91 (0.87-0.95)	0.92 (0.89-0.96)	0.95 (0.91-0.99)	0.95 (0.90-1.00)
Schizophrenia	0.62 (0.53-0.72)	0.62 (0.54-0.71)	0.62 (0.54-0.70)	0.65 (0.58-0.73)	0.73 (0.66-0.81)	0.74 (0.66-0.82)	0.78 (0.69-0.88)
Bipolar disorder	1.06 (0.96-1.16)	1.04 (0.95-1.12)	1.06 (0.98-1.14)	1.05 (0.98-1.13)	1.08 (1.01-1.15)	1.10 (1.03-1.18)	1.10 (1.03-1.18)
Other	0.90 (0.84-0.97)	0.88 (0.82-0.94)	0.88 (0.82-0.94)	0.90 (0.85-0.96)	0.90 (0.84-0.95)	0.91 (0.86-0.97)	0.89 (0.83-0.96)

	Adjusted odds ratios (95% confidence interval)						
	**Years before severe mental illness diagnosis**				**Years after severe mental illness diagnosis**		
	**-5**	**-3**	**-1**	**Index**	**+1**	**+3**	**+5**
**Diabetes**							
All SMI	1.10 (1.05-1.16)	1.12 (1.07-1.17)	1.18 (1.14-1.23)	1.28 (1.24-1.33)	1.51 (1.46-1.56)	1.66 (1.60-1.72)	1.86 (1.79-1.93)
Schizophrenia	0.95 (0.85-1.07)	1.03 (0.93-1.14)	1.11 (1.02-1.22)	1.27 (1.18-1.38)	1.70 (1.59-1.82)	1.84 (1.73-1.97)	2.10 (1.96-2.26)
Bipolar disorder	1.27 (1.18-1.38)	1.31 (1.22-1.40)	1.41 (1.33-1.49)	1.50 (1.42-1.58)	1.72 (1.63-1.81)	1.87 (1.78-1.97)	2.04 (1.93-2.15)
Other	1.06 (0.99-1.13)	1.05 (0.99-1.11)	1.07 (1.02-1.13)	1.15 (1.09-1.21)	1.30 (1.24-1.37)	1.43 (1.35-1.50)	1.58 (1.49-1.67)
**Hypothyroid**							
All SMI	1.23 (1.15-1.30)	1.23 (1.17-1.30)	1.29 (1.23-1.35)	1.36 (1.30-1.43)	1.59 (1.53-1.66)	1.73 (1.65-1.81)	1.91 (1.82-2.01)
Schizophrenia	1.03 (0.89-1.20)	1.01 (0.88-1.15)	1.03 (0.91-1.16)	1.09 (0.97-1.22)	1.33 (1.21-1.47)	1.37 (1.24-1.51)	1.41 (1.26-1.58)
Bipolar disorder	1.53 (1.40-1.67)	1.58 (1.47-1.71)	1.66 (1.55-1.78)	1.75 (1.64-1.87)	2.17 (2.05-2.30)	2.42 (2.28-2.56)	2.68 (2.52-2.85)
Other	1.09 (1.01-1.19)	1.07 (1.00-1.16)	1.12 (1.04-1.20)	1.18 (1.11-1.26)	1.28 (1.20-1.36)	1.32 (1.24-1.41)	1.41 (1.31-1.52)
**Liver Disease**							
All SMI	1.45 (1.32-1.59)	1.49 (1.37-1.63)	1.60 (1.47-1.74)	1.65 (1.52-1.78)	1.79 (1.67-1.93)	1.88 (1.74-2.03)	1.77 (1.62-1.93)
Schizophrenia	1.32 (1.11-1.58)	1.35 (1.14-1.59)	1.49 (1.28-1.73)	1.58 (1.38-1.81)	1.84 (1.62-2.08)	1.94 (1.70-2.22)	1.80 (1.55-2.10)
Bipolar disorder	1.44 (1.26-1.65)	1.43 (1.26-1.62)	1.43 (1.27-1.60)	1.43 (1.28-1.59)	1.55 (1.40-1.72)	1.67 (1.50-1.86)	1.56 (1.37-1.78)
Other	1.51 (1.33-1.71)	1.61 (1.43-1.81)	1.78 (1.59-1.98)	1.85 (1.67-2.05)	1.95 (1.77-2.15)	2.02 (1.82-2.25)	1.93 (1.72-2.17)
**Renal Disease**							
All SMI	0.96 (0.88-1.04)	0.95 (0.89-1.02)	0.98 (0.92-1.03)	1.02 (0.96-1.08)	1.12 (1.06-1.17)	1.23 (1.17-1.29)	1.42 (1.35-1.50)
Schizophrenia	0.71 (0.56-0.88)	0.79 (0.66-0.94)	0.81 (0.69-0.94)	0.83 (0.72-0.96)	1.00 (0.89-1.13)	1.16 (1.04-1.30)	1.37 (1.22-1.53)
Bipolar disorder	0.85 (0.72-0.99)	0.88 (0.77-1.00)	0.98 (0.88-1.09)	1.12 (1.02-1.23)	1.28 (1.18-1.40)	1.46 (1.36-1.58)	1.65 (1.53-1.78)
Other	1.08 (0.98-1.19)	1.03 (0.95-1.12)	1.02 (0.95-1.09)	1.02 (0.95-1.09)	1.07 (1.00-1.14)	1.11 (1.04-1.19)	1.26 (1.17-1.35)
**Peptic Ulcer**							
All SMI	1.09 (1.01-1.17)	1.08 (1.01-1.16)	1.14 (1.06-1.22)	1.16 (1.09-1.24)	1.21 (1.14-1.29)	1.20 (1.12-1.28)	1.21 (1.11-1.31)
Schizophrenia	0.72 (0.60-0.86)	0.72 (0.61-0.85)	0.72 (0.61-0.85)	0.74 (0.64-0.86)	0.79 (0.68-0.92)	0.79 (0.68-0.92)	0.83 (0.69-0.99)
Bipolar disorder	1.16 (1.04-1.31)	1.15 (1.03-1.29)	1.23 (1.11-1.37)	1.27 (1.15-1.41)	1.32 (1.20-1.45)	1.30 (1.17-1.44)	1.31 (1.17-1.47)
Other	1.19 (1.08-1.31)	1.19 (1.08-1.30)	1.24 (1.14-1.36)	1.27 (1.16-1.38)	1.31 (1.21-1.43)	1.31 (1.20-1.43)	1.32 (1.18-1.46)
**Rheumatic Disease**							
All SMI	1.02 (0.94-1.10)	1.02 (0.95-1.09)	1.01 (0.94-1.07)	1.03 (0.97-1.10)	1.05 (0.98-1.11)	1.00 (0.94-1.07)	0.99 (0.92-1.07)
Schizophrenia	0.71 (0.57-0.87)	0.66 (0.55-0.80)	0.66 (0.55-0.79)	0.70 (0.60-0.83)	0.75 (0.65-0.88)	0.73 (0.62-0.85)	0.76 (0.63-0.90)
Bipolar disorder	1.09 (0.96-1.24)	1.07 (0.95-1.20)	1.07 (0.97-1.19)	1.11 (1.00-1.22)	1.13 (1.03-1.25)	1.10 (0.99-1.21)	1.11 (0.99-1.24)
Other	1.07 (0.97-1.18)	1.10 (1.00-1.21)	1.08 (0.99-1.18)	1.09 (1.01-1.19)	1.09 (1.00-1.18)	1.04 (0.95-1.14)	0.99 (0.89-1.10)
**Paralysis or paresis**							
All SMI	1.94 (1.70-2.21)	1.88 (1.65-2.13)	1.87 (1.66-2.12)	1.94 (1.73-2.18)	2.00 (1.78-2.23)	2.05 (1.82-2.31)	1.95 (1.69-2.25)
Schizophrenia	1.41 (1.06-1.86)	1.36 (1.04-1.79)	1.40 (1.09-1.80)	1.44 (1.13-1.84)	1.49 (1.18-1.88)	1.44 (1.13-1.85)	1.41 (1.05-1.89)
Bipolar disorder	1.93 (1.60-2.33)	1.91 (1.59-2.28)	1.87 (1.57-2.23)	1.90 (1.61-2.25)	1.97 (1.67-2.32)	2.07 (1.74-2.45)	1.92 (1.58-2.33)
Other	2.19 (1.85-2.60)	2.09 (1.76-2.47)	2.10 (1.78-2.47)	2.21 (1.89-2.57)	2.25 (1.94-2.61)	2.35 (2.02-2.73)	2.30 (1.91-2.77)
**HIV**							
All SMI	1.48 (1.24-1.77)	1.56 (1.35-1.80)	1.47 (1.30-1.66)	1.54 (1.37-1.73)	1.65 (1.46-1.86)	1.70 (1.47-1.96)	1.58 (1.37-1.82)
Schizophrenia	0.88 (0.55-1.42)	0.82 (0.54-1.24)	0.82 (0.59-1.16)	1.02 (0.76-1.37)	1.04 (0.79-1.37)	1.30 (0.98-1.72)	1.40 (1.08-1.83)
Bipolar disorder	1.64 (1.26-2.13)	1.60 (1.29-1.98)	1.54 (1.29-1.84)	1.56 (1.32-1.83)	1.72 (1.48-2.01)	1.78 (1.50-2.12)	1.60 (1.32-1.93)
Other	1.58 (1.24-2.01)	1.81 (1.48-2.22)	1.67 (1.40-2.00)	1.75 (1.47-2.08)	1.84 (1.55-2.20)	1.81 (1.48-2.22)	1.66 (1.35-2.05)

	Adjusted odds ratios (95% confidence interval)						
	**Years before severe mental illness diagnosis**				**Years after severe mental illness diagnosis**		
	**-5**	**-3**	**-1**	**Index**	**+1**	**+3**	**+5**
**Hypertension**							
All SMI	0.81 (0.78-0.84)	0.81 (0.78-0.84)	0.82 (0.79-0.84)	0.84 (0.82-0.87)	0.91 (0.89-0.94)	0.92 (0.89-0.95)	0.98 (0.95-1.02)
Schizophrenia	0.53 (0.48-0.58)	0.54 (0.50-0.59)	0.54 (0.50-0.59)	0.57 (0.53-0.62)	0.73 (0.68-0.78)	0.76 (0.71-0.82)	0.84 (0.77-0.90)
Bipolar disorder	0.90 (0.85-0.95)	0.93 (0.89-0.98)	0.96 (0.91-1.01)	1.01 (0.96-1.06)	1.09 (1.05-1.14)	1.08 (1.03-1.13)	1.13 (1.07-1.19)
Other	0.85 (0.82-0.90)	0.84 (0.80-0.88)	0.83 (0.80-0.87)	0.85 (0.81-0.88)	0.87 (0.83-0.90)	0.86 (0.82-0.90)	0.92 (0.87-0.97)
**Peripheral Vascular Disease**							
All SMI	0.95 (0.85-1.06)	0.98 (0.89-1.08)	1.02 (0.94-1.11)	1.05 (0.97-1.14)	1.09 (1.01-1.18)	1.17 (1.08-1.27)	1.13 (1.03-1.24)
Schizophrenia	0.63 (0.46-0.85)	0.69 (0.53-0.91)	0.74 (0.59-0.94)	0.77 (0.63-0.96)	0.88 (0.73-1.06)	0.95 (0.79-1.14)	1.07 (0.86-1.32)
Bipolar disorder	0.96 (0.78-1.18)	1.03 (0.86-1.22)	1.08 (0.92-1.26)	1.12 (0.96-1.30)	1.17 (1.02-1.34)	1.36 (1.19-1.54)	1.30 (1.13-1.50)
Other	1.02 (0.89-1.17)	1.03 (0.91-1.16)	1.08 (0.97-1.20)	1.10 (1.00-1.22)	1.11 (1.01-1.23)	1.13 (1.02-1.26)	1.02 (0.89-1.16)
**Pulmonary Circulation Disorders**							
All SMI	1.10 (0.95-1.27)	1.19 (1.05-1.35)	1.33 (1.19-1.49)	1.41 (1.27-1.57)	1.49 (1.35-1.64)	1.63 (1.48-1.81)	1.58 (1.41-1.78)
Schizophrenia	0.66 (0.44-0.99)	0.88 (0.62-1.24)	1.04 (0.78-1.38)	1.17 (0.91-1.51)	1.25 (0.99-1.58)	1.45 (1.15-1.82)	1.58 (1.23-2.03)
Bipolar disorder	1.43 (1.16-1.77)	1.47 (1.21-1.78)	1.57 (1.32-1.87)	1.62 (1.38-1.89)	1.72 (1.49-1.99)	1.77 (1.53-2.06)	1.74 (1.47-2.05)
Other	1.04 (0.86-1.26)	1.12 (0.94-1.33)	1.28 (1.11-1.49)	1.36 (1.19-1.57)	1.42 (1.26-1.61)	1.60 (1.40-1.82)	1.45 (1.22-1.73)
**Valvular Disease**							
All SMI	0.98 (0.88-1.09)	0.97 (0.88-1.07)	0.97 (0.89-1.07)	0.98 (0.90-1.07)	1.03 (0.95-1.12)	0.97 (0.89-1.06)	0.94 (0.85-1.04)
Schizophrenia	0.79 (0.60-1.04)	0.80 (0.62-1.02)	0.76 (0.60-0.95)	0.73 (0.58-0.90)	0.80 (0.66-0.98)	0.79 (0.65-0.97)	0.72 (0.56-0.92)
Bipolar disorder	0.99 (0.82-1.18)	0.97 (0.82-1.14)	0.99 (0.86-1.15)	0.99 (0.86-1.15)	1.07 (0.94-1.23)	1.01 (0.88-1.16)	0.95 (0.81-1.11)
Other	1.04 (0.90-1.20)	1.02 (0.89-1.16)	1.03 (0.92-1.16)	1.06 (0.95-1.18)	1.08 (0.97-1.19)	1.01 (0.91-1.13)	1.02 (0.89-1.18)
**Deficiency Anaemia**							
All SMI	1.12 (1.05-1.19)	1.14 (1.07-1.20)	1.17 (1.11-1.23)	1.21 (1.15-1.27)	1.29 (1.23-1.35)	1.27 (1.22-1.33)	1.29 (1.23-1.36)
Schizophrenia	0.96 (0.82-1.11)	0.94 (0.82-1.08)	1.00 (0.89-1.12)	1.02 (0.92-1.14)	1.15 (1.05-1.27)	1.16 (1.05-1.28)	1.23 (1.11-1.37)
Bipolar disorder	1.07 (0.96-1.18)	1.10 (1.01-1.21)	1.12 (1.03-1.21)	1.15 (1.06-1.24)	1.22 (1.13-1.30)	1.22 (1.14-1.31)	1.26 (1.17-1.36)
Other	1.21 (1.11-1.32)	1.23 (1.14-1.33)	1.26 (1.18-1.35)	1.33 (1.24-1.41)	1.39 (1.31-1.48)	1.37 (1.28-1.46)	1.36 (1.25-1.47)
**Blood Loss Anaemia**							
All SMI	0.77 (0.45-1.33)	0.79 (0.49-1.28)	1.05 (0.70-1.57)	1.00 (0.69-1.46)	1.02 (0.70-1.48)	0.80 (0.52-1.22)	0.66 (0.42-1.05)
Schizophrenia	0.98 (0.32-2.99)	1.25 (0.52-2.98)	1.52 (0.76-3.03)	1.68 (0.90-3.12)	1.55 (0.84-2.88)	0.87 (0.38-1.98)	0.59 (0.22-1.60)
Bipolar disorder	0.32 (0.08-1.31)	0.52 (0.19-1.42)	0.87 (0.42-1.79)	0.78 (0.38-1.58)	0.82 (0.42-1.59)	0.56 (0.27-1.19)	0.65 (0.32-1.30)
Other	1.02 (0.51-2.04)	0.82 (0.41-1.61)	1.00 (0.56-1.77)	0.88 (0.50-1.55)	0.95 (0.55-1.64)	0.96 (0.54-1.70)	0.71 (0.36-1.37)
**Coagulopathy**							
All SMI	1.17 (0.99-1.38)	1.12 (0.96-1.30)	1.07 (0.93-1.23)	1.11 (0.97-1.26)	1.22 (1.08-1.38)	1.28 (1.13-1.46)	1.39 (1.21-1.59)
Schizophrenia	0.76 (0.51-1.13)	0.74 (0.51-1.07)	0.75 (0.53-1.06)	0.75 (0.54-1.04)	0.85 (0.64-1.14)	1.07 (0.81-1.40)	1.11 (0.83-1.49)
Bipolar disorder	1.30 (1.02-1.64)	1.25 (1.00-1.56)	1.22 (0.99-1.50)	1.21 (1.00-1.47)	1.30 (1.08-1.57)	1.35 (1.11-1.64)	1.42 (1.16-1.73)
Other	1.24 (1.00-1.53)	1.16 (0.95-1.42)	1.09 (0.91-1.31)	1.18 (1.00-1.39)	1.31 (1.12-1.53)	1.33 (1.12-1.57)	1.50 (1.25-1.80)
**Fluids and Electrolyte**							
All SMI	1.22 (1.05-1.41)	1.21 (1.07-1.37)	1.34 (1.21-1.49)	1.52 (1.39-1.66)	1.79 (1.66-1.93)	1.92 (1.78-2.07)	2.01 (1.86-2.18)
Schizophrenia	0.86 (0.57-1.30)	0.93 (0.67-1.29)	0.96 (0.73-1.26)	1.00 (0.79-1.28)	1.24 (1.02-1.52)	1.50 (1.26-1.79)	1.53 (1.27-1.85)
Bipolar disorder	1.51 (1.21-1.90)	1.42 (1.17-1.73)	1.66 (1.42-1.94)	1.84 (1.60-2.11)	2.24 (1.99-2.51)	2.25 (2.02-2.51)	2.29 (2.04-2.58)
Other	1.17 (0.97-1.41)	1.18 (1.01-1.38)	1.29 (1.14-1.47)	1.51 (1.35-1.69)	1.71 (1.56-1.88)	1.86 (1.69-2.05)	1.99 (1.78-2.22)

*adjusted for age, sex, ethnicity, index date calendar year, and primary care practice region

Code lists: Severe mental illness

Read code	Term	Group
E110	Manic disorder, single episode	bipolar
E110-1	Hypomanic psychoses	bipolar
E110-99	Mania/hypomania	bipolar
E1100	Single manic episode, unspecified	bipolar
E1101	Single manic episode, mild	bipolar
E1102	Single manic episode, moderate	bipolar
E1103	Single manic episode, severe without mention of psychosis	bipolar
E1104	Single manic episode, severe, with psychosis	bipolar
E1105	Single manic episode in partial or unspecified remission	bipolar
E1106	Single manic episode in full remission	bipolar
E110z	Manic disorder, single episode NOS	bipolar
E111	Recurrent manic episodes	bipolar
E1110	Recurrent manic episodes, unspecified	bipolar
E1111	Recurrent manic episodes, mild	bipolar
E1112	Recurrent manic episodes, moderate	bipolar
E1113	Recurrent manic episodes, severe without mention psychosis	bipolar
E1114	Recurrent manic episodes, severe, with psychosis	bipolar
E1115	Recurrent manic episodes, partial or unspecified remission	bipolar
E1116	Recurrent manic episodes, in full remission	bipolar
E111z	Recurrent manic episode NOS	bipolar
E114	Bipolar affective disorder, currently manic	bipolar
E114-1	Manic-depressive - now manic	bipolar
E1140	Bipolar affective disorder, currently manic, unspecified	bipolar
E1141	Bipolar affective disorder, currently manic, mild	bipolar
E1142	Bipolar affective disorder, currently manic, moderate	bipolar
E1143	Bipolar affect disord, currently manic, severe, no psychosis	bipolar
E1144	Bipolar affect disord, currently manic,severe with psychosis	bipolar
E1145	Bipolar affect disord,currently manic, part/unspec remission	bipolar
E1146	Bipolar affective disorder, currently manic, full remission	bipolar
E114z	Bipolar affective disorder, currently manic, NOS	bipolar
E115	Bipolar affective disorder, currently depressed	bipolar
E115-1	Manic-depressive - now depressed	bipolar
E1150	Bipolar affective disorder, currently depressed, unspecified	bipolar
E1151	Bipolar affective disorder, currently depressed, mild	bipolar
E1152	Bipolar affective disorder, currently depressed, moderate	bipolar
E1153	Bipolar affect disord, now depressed, severe, no psychosis	bipolar
E1154	Bipolar affect disord, now depressed, severe with psychosis	bipolar
E1155	Bipolar affect disord, now depressed, part/unspec remission	bipolar
E1156	Bipolar affective disorder, now depressed, in full remission	bipolar
E115z	Bipolar affective disorder, currently depressed, NOS	bipolar
E116	Mixed bipolar affective disorder	bipolar
E1160	Mixed bipolar affective disorder, unspecified	bipolar
E1161	Mixed bipolar affective disorder, mild	bipolar
E1162	Mixed bipolar affective disorder, moderate	bipolar

E1163	Mixed bipolar affective disorder, severe, without psychosis	bipolar
E1164	Mixed bipolar affective disorder, severe, with psychosis	bipolar
E1165	Mixed bipolar affective disorder, partial/unspec remission	bipolar
E1166	Mixed bipolar affective disorder, in full remission	bipolar
E116z	Mixed bipolar affective disorder, NOS	bipolar
E117	Unspecified bipolar affective disorder	bipolar
E1170	Unspecified bipolar affective disorder, unspecified	bipolar
E1171	Unspecified bipolar affective disorder, mild	bipolar
E1172	Unspecified bipolar affective disorder, moderate	bipolar
E1173	Unspecified bipolar affective disorder, severe, no psychosis	bipolar
E1174	Unspecified bipolar affective disorder,severe with psychosis	bipolar
E1175	Unspecified bipolar affect disord, partial/unspec remission	bipolar
E1176	Unspecified bipolar affective disorder, in full remission	bipolar
E117z	Unspecified bipolar affective disorder, NOS	bipolar
E11y	Other and unspecified manic-depressive psychoses	bipolar
E11y0	Unspecified manic-depressive psychoses	bipolar
E11y1	Atypical manic disorder	bipolar
E11y3	Other mixed manic-depressive psychoses	bipolar
E11yz	Other and unspecified manic-depressive psychoses NOS	bipolar
E11z	Other and unspecified affective psychoses	bipolar
E11z0	Unspecified affective psychoses NOS	bipolar
E11zz	Other affective psychosis NOS	bipolar
Eu20	[X]Schizophrenia	schizophrenia
Eu30	[X]Manic episode	bipolar
Eu30-1	[X]Bipolar disorder, single manic episode	bipolar
Eu300	[X]Hypomania	bipolar
Eu301	[X]Mania without psychotic symptoms	bipolar
Eu302	[X]Mania with psychotic symptoms	bipolar
Eu302-1	[X]Mania with mood-congruent psychotic symptoms	bipolar
Eu302-2	[X]Mania with mood-incongruent psychotic symptoms	bipolar
Eu302-3	[X]Manic stupor	bipolar
Eu30y	[X]Other manic episodes	bipolar
Eu30z	[X]Manic episode, unspecified	bipolar
Eu30z-1	[X]Mania NOS	bipolar
Eu31	[X]Bipolar affective disorder	bipolar
Eu31-1	[X]Manic-depressive illness	bipolar
Eu31-2	[X]Manic-depressive psychosis	bipolar
Eu31-3	[X]Manic-depressive reaction	bipolar
Eu310	[X]Bipolar affective disorder, current episode hypomanic	bipolar
Eu311	[X]Bipolar affect disorder cur epi manic wout psychotic symp	bipolar
Eu312	[X]Bipolar affect disorder cur epi manic with psychotic symp	bipolar
Eu313	[X]Bipolar affect disorder cur epi mild or moderate depressn	bipolar
Eu314	[X]Bipol aff disord, curr epis sev depress, no psychot symp	bipolar
Eu315	[X]Bipolar affect dis cur epi severe depres with psyc symp	bipolar
Eu316	[X]Bipolar affective disorder, current episode mixed	bipolar
Eu317	[X]Bipolar affective disorder, currently in remission	bipolar

Eu318	[X]Bipolar affective disorder type I	bipolar
Eu31y	[X]Other bipolar affective disorders	bipolar
Eu31y-1	[X]Bipolar II disorder	bipolar
Eu31y-2	[X]Recurrent manic episodes	bipolar
Eu31z	[X]Bipolar affective disorder, unspecified	bipolar
Eu333-2	[X]Manic-depress psychosis,depressed type+psychotic symptoms	bipolar
E11	Affective psychoses	bipolar
E11-1	Bipolar psychoses	bipolar
E11-99	Manic-depressive psychoses	bipolar
Eu3z-1	[X]Affective psychosis NOS	bipolar
146D	H/O: manic depressive disorder	bipolar
1S42	Manic mood	bipolar
ZV111-1	[V]Personal history of manic-depressive psychosis	bipolar
ZV111-2	[V]Personal history of manic-depressive psychosis	bipolar
Eu332-3	[X]Manic-depress psychosis,depressd,no psychotic symptoms	bipolar
E104-1	Oneirophrenia	other psychosis
E105	Latent schizophrenia	other psychosis
E1050	Unspecified latent schizophrenia	other psychosis
E1051	Subchronic latent schizophrenia	other psychosis
E1052	Chronic latent schizophrenia	other psychosis
E1053	Acute exacerbation of subchronic latent schizophrenia	other psychosis
E1054	Acute exacerbation of chronic latent schizophrenia	other psychosis
E1055	Latent schizophrenia in remission	other psychosis
E105z	Latent schizophrenia NOS	other psychosis
E107-1	Cyclic schizophrenia	other psychosis
E107-99	Acute schizo affective psychosis	other psychosis
E12	Paranoid states	other psychosis
E12-99	Paranoia	other psychosis
E120	Simple paranoid state	other psychosis
E121	Chronic paranoid psychosis	other psychosis
E121-1	Sander's disease	other psychosis
E122	Paraphrenia	other psychosis
E123	Shared paranoid disorder	other psychosis
E123-1	Folie a deux	other psychosis
E12y	Other paranoid states	other psychosis
E12y0	Paranoia querulans	other psychosis
E12yz	Other paranoid states NOS	other psychosis
E12z	Paranoid psychosis NOS	other psychosis
E13	Other nonorganic psychoses	other psychosis
E13-1	Reactive psychoses	other psychosis
E131	Acute hysterical psychosis	other psychosis
E133	Acute paranoid reaction	other psychosis
E133-1	Bouffee delirante	other psychosis
E134	Psychogenic paranoid psychosis	other psychosis
E13y	Other reactive psychoses	other psychosis
E13y0	Psychogenic stupor	other psychosis

E13y1	Brief reactive psychosis	other psychosis
E13yz	Other reactive psychoses NOS	other psychosis
E13z	Nonorganic psychosis NOS	other psychosis
E13z-1	Psychotic episode NOS	other psychosis
Eu2	[X]Schizophrenia, schizotypal and delusional disorders	other psychosis
Eu21	[X]Schizotypal disorder	other psychosis
Eu21-1	[X]Latent schizophrenic reaction	other psychosis
Eu21-2	[X]Borderline schizophrenia	other psychosis
Eu21-3	[X]Latent schizophrenia	other psychosis
Eu21-4	[X]Prepsychotic schizophrenia	other psychosis
Eu21-5	[X]Prodromal schizophrenia	other psychosis
Eu21-6	[X]Pseudoneurotic schizophrenia	other psychosis
Eu21-7	[X]Pseudopsychopathic schizophrenia	other psychosis
Eu21-8	[X]Schizotypal personality disorder	other psychosis
Eu22	[X]Persistent delusional disorders	other psychosis
Eu220	[X]Delusional disorder	other psychosis
Eu220-1	[X]Paranoid psychosis	other psychosis
Eu220-2	[X]Paranoid state	other psychosis
Eu220-3	[X]Paraphrenia - late	other psychosis
Eu220-4	[X]Sensitiver Beziehungswahn	other psychosis
Eu220-5	[X]Paranoia	other psychosis
Eu221	[X]Delusional misidentification syndrome	other psychosis
Eu221-1	[X]Capgras syndrome	other psychosis
Eu22y	[X]Other persistent delusional disorders	other psychosis
Eu22y-1	[X]Delusional dysmorphophobia	other psychosis
Eu22y-2	[X]Involutional paranoid state	other psychosis
Eu22y-3	[X]Paranoia querulans	other psychosis
Eu22z	[X]Persistent delusional disorder, unspecified	other psychosis
Eu23	[X]Acute and transient psychotic disorders	other psychosis
Eu230	[X]Acute polymorphic psychot disord without symp of schizoph	other psychosis
Eu230-1	[X]Bouffee delirante	other psychosis
Eu230-2	[X]Cycloid psychosis	other psychosis
Eu231	[X]Acute polymorphic psychot disord with symp of schizophren	other psychosis
Eu231-1	[X]Bouffee delirante with symptoms of schizophrenia	other psychosis
Eu231-2	[X]Cycloid psychosis with symptoms of schizophrenia	other psychosis
Eu232	[X]Acute schizophrenia-like psychotic disorder	other psychosis
Eu232-1	[X]Brief schizophreniform disorder	other psychosis
Eu232-2	[X]Brief schizophrenifrm psych	other psychosis
Eu232-3	[X]Oneirophrenia	other psychosis
Eu233	[X]Other acute predominantly delusional psychotic disorders	other psychosis
Eu233-2	[X]Psychogenic paranoid psychosis	other psychosis
Eu23y	[X]Other acute and transient psychotic disorders	other psychosis
Eu23z	[X]Acute and transient psychotic disorder, unspecified	other psychosis
Eu23z-1	[X]Brief reactive psychosis NOS	other psychosis
Eu23z-2	[X]Reactive psychosis	other psychosis
Eu24	[X]Induced delusional disorder	other psychosis

Eu24-1	[X]Folie a deux	other psychosis
Eu24-2	[X]Induced paranoid disorder	other psychosis
Eu24-3	[X]Induced psychotic disorder	other psychosis
Eu25	[X]Schizoaffective disorders	other psychosis
Eu250	[X]Schizoaffective disorder, manic type	other psychosis
Eu250-1	[X]Schizoaffective psychosis, manic type	other psychosis
Eu250-2	[X]Schizophreniform psychosis, manic type	other psychosis
Eu251	[X]Schizoaffective disorder, depressive type	other psychosis
Eu251-1	[X]Schizoaffective psychosis, depressive type	other psychosis
Eu251-2	[X]Schizophreniform psychosis, depressive type	other psychosis
Eu252	[X]Schizoaffective disorder, mixed type	other psychosis
Eu252-1	[X]Cyclic schizophrenia	other psychosis
Eu252-2	[X]Mixed schizophrenic and affective psychosis	other psychosis
Eu25y	[X]Other schizoaffective disorders	other psychosis
Eu25z	[X]Schizoaffective disorder, unspecified	other psychosis
Eu25z-1	[X]Schizoaffective psychosis NOS	other psychosis
Eu2y	[X]Other nonorganic psychotic disorders	other psychosis
Eu2y-1	[X]Chronic hallucinatory psychosis	other psychosis
Eu2z	[X]Unspecified nonorganic psychosis	other psychosis
Eu2z-1	[X]Psychosis NOS	other psychosis
E11-3	Manic psychoses	other psychosis
E1z	Non-organic psychosis NOS	other psychosis
146H	H/O: psychosis	other psychosis
E1	Non-organic psychoses	other psychosis
E1y	Other specified non-organic psychoses	other psychosis
Eu531-1	[X]Puerperal psychosis NOS	other psychosis
Eu843-4	[X]Symbiotic psychosis	other psychosis
E10	Schizophrenic disorders	schizophrenia
E10-98	Schizophrenic psychoses NOS	schizophrenia
E10-99	Schizophrenic psychoses	schizophrenia
E100	Simple schizophrenia	schizophrenia
E100-1	Schizophrenia simplex	schizophrenia
E1000	Unspecified schizophrenia	schizophrenia
E1001	Subchronic schizophrenia	schizophrenia
E1002	Chronic schizophrenic	schizophrenia
E1003	Acute exacerbation of subchronic schizophrenia	schizophrenia
E1004	Acute exacerbation of chronic schizophrenia	schizophrenia
E1005	Schizophrenia in remission	schizophrenia
E100z	Simple schizophrenia NOS	schizophrenia
E101	Hebephrenic schizophrenia	schizophrenia
E1010	Unspecified hebephrenic schizophrenia	schizophrenia
E1011	Subchronic hebephrenic schizophrenia	schizophrenia
E1012	Chronic hebephrenic schizophrenia	schizophrenia
E1013	Acute exacerbation of subchronic hebephrenic schizophrenia	schizophrenia
E1014	Acute exacerbation of chronic hebephrenic schizophrenia	schizophrenia
E1015	Hebephrenic schizophrenia in remission	schizophrenia

E101z	Hebephrenic schizophrenia NOS	schizophrenia
E102	Catatonic schizophrenia	schizophrenia
E1020	Unspecified catatonic schizophrenia	schizophrenia
E1021	Subchronic catatonic schizophrenia	schizophrenia
E1022	Chronic catatonic schizophrenia	schizophrenia
E1023	Acute exacerbation of subchronic catatonic schizophrenia	schizophrenia
E1024	Acute exacerbation of chronic catatonic schizophrenia	schizophrenia
E1025	Catatonic schizophrenia in remission	schizophrenia
E102z	Catatonic schizophrenia NOS	schizophrenia
E103	Paranoid schizophrenia	schizophrenia
E1030	Unspecified paranoid schizophrenia	schizophrenia
E1031	Subchronic paranoid schizophrenia	schizophrenia
E1032	Chronic paranoid schizophrenia	schizophrenia
E1033	Acute exacerbation of subchronic paranoid schizophrenia	schizophrenia
E1034	Acute exacerbation of chronic paranoid schizophrenia	schizophrenia
E1035	Paranoid schizophrenia in remission	schizophrenia
E103z	Paranoid schizophrenia NOS	schizophrenia
E104	Acute schizophrenic episode	schizophrenia
E106	Residual schizophrenia	schizophrenia
E106-1	Restzustand - schizophrenia	schizophrenia
E107	Schizo-affective schizophrenia	schizophrenia
E1070	Unspecified schizo-affective schizophrenia	schizophrenia
E1071	Subchronic schizo-affective schizophrenia	schizophrenia
E1072	Chronic schizo-affective schizophrenia	schizophrenia
E1073	Acute exacerbation subchronic schizo-affective schizophrenia	schizophrenia
E1074	Acute exacerbation of chronic schizo-affective schizophrenia	schizophrenia
E1075	Schizo-affective schizophrenia in remission	schizophrenia
E107z	Schizo-affective schizophrenia NOS	schizophrenia
E10y	Other schizophrenia	schizophrenia
E10y-1	Cenesthopathic schizophrenia	schizophrenia
E10y0	Atypical schizophrenia	schizophrenia
E10y1	Coenesthopathic schizophrenia	schizophrenia
E10yz	Other schizophrenia NOS	schizophrenia
E10z	Schizophrenia NOS	schizophrenia
Eu200	[X]Paranoid schizophrenia	schizophrenia
Eu200-1	[X]Paraphrenic schizophrenia	schizophrenia
Eu201	[X]Hebephrenic schizophrenia	schizophrenia
Eu201-1	[X]Disorganised schizophrenia	schizophrenia
Eu202	[X]Catatonic schizophrenia	schizophrenia
Eu202-1	[X]Catatonic stupor	schizophrenia
Eu202-2	[X]Schizophrenic catalepsy	schizophrenia
Eu202-3	[X]Schizophrenic catatonia	schizophrenia
Eu202-4	[X]Schizophrenic flexibilatis cerea	schizophrenia
Eu203	[X]Undifferentiated schizophrenia	schizophrenia
Eu203-1	[X]Atypical schizophrenia	schizophrenia
Eu204	[X]Post-schizophrenic depression	schizophrenia

Eu205	[X]Residual schizophrenia	schizophrenia
Eu205-1	[X]Chronic undifferentiated schizophrenia	schizophrenia
Eu205-2	[X]Restzustand schizophrenic	schizophrenia
Eu206	[X]Simple schizophrenia	schizophrenia
Eu20y	[X]Other schizophrenia	schizophrenia
Eu20y-1	[X]Cenesthopathic schizophrenia	schizophrenia
Eu20y-2	[X]Schizophreniform disord NOS	schizophrenia
Eu20y-3	[X]Schizophrenifrm psychos NOS	schizophrenia
Eu20z	[X]Schizophrenia, unspecified	schizophrenia
Eu232-4	[X]Schizophrenic reaction	schizophrenia
ZV110	[V]Personal history of schizophrenia	schizophrenia
1464	H/O: schizophrenia	schizophrenia

**Additional EMIS codes**	**Term**	**Group**
EMISQHY1	Hypomanic	bipolar
EGTON118	Obsessional compulsive psychosis	other psychosis
EMISCDE13	Delusions	other psychosis
EMISICD10|F2381	Other acute and transient psychotic disorders, with associated acute stress	other psychosis
EMISQPA1	Paranoid	other psychosis
EMISICD10|F2018	Hebephrenic schizophrenia, other	schizophrenia
EMISICD10|F2035	Undifferentiated schizophrenia, complete remission	schizophrenia
EMISICD10|F2041	Post-schizophrenic depression, episodic with progressive deficit	schizophrenia
EMISICD10|F2054	Residual schizophrenia, incomplete remission	schizophrenia
EMISICD10|F2065	Simple schizophrenia, complete remission	schizophrenia
EMISICD10|F2098	Schizophrenia, unspecified, other	schizophrenia

Code lists: Chronic physical health conditions

**Read code**	**Term**	**Group**
H330000	extrinsic asthma without status asthmaticus	Asthma
38DT.00	asthma control questionnaire	Asthma
38DV.00	mini asthma quality of life questionnaire	Asthma
9NNX.00	under care of asthma specialist nurse	Asthma
8CMA000	patient has a written asthma personal action plan	Asthma
679J000	health education - asthma self management	Asthma
679J100	health education - structured asthma discussion	Asthma
663t.00	asthma causes daytime symptoms 1 to 2 times per month	Asthma
663u.00	asthma causes daytime symptoms 1 to 2 times per week	Asthma
663v.00	asthma causes daytime symptoms most days	Asthma
663r.00	asthma causes night symptoms 1 to 2 times per month	Asthma
663x.00	asthma limits walking on the flat	Asthma
663w.00	asthma limits walking up hills or stairs	Asthma
663s.00	asthma never causes daytime symptoms	Asthma
663y.00	number of asthma exacerbations in past year	Asthma
663V100	mild asthma	Asthma
663V200	moderate asthma	Asthma

663V000	occasional asthma	Asthma
663V300	severe asthma	Asthma
663p.00	asthma treatment compliance unsatisfactory	Asthma
663n.00	asthma treatment compliance satisfactory	Asthma
663q.00	asthma daytime symptoms	Asthma
9hA2.00	excepted from asthma quality indicators: informed dissent	Asthma
9hA..00	exception reporting: asthma quality indicators	Asthma
H33z011	severe asthma attack	Asthma
H330.11	allergic asthma	Asthma
66Y5.00	change in asthma management plan	Asthma
66Y9.00	step up change in asthma management plan	Asthma
66YA.00	step down change in asthma management plan	Asthma
66YC.00	absent from work or school due to asthma	Asthma
66YE.00	asthma monitoring due	Asthma
66YJ.00	asthma annual review	Asthma
66YK.00	asthma follow-up	Asthma
8B3j.00	asthma medication review	Asthma
66YP.00	asthma night-time symptoms	Asthma
H35y600	sequoiosis (red-cedar asthma)	Asthma
H330100	extrinsic asthma with status asthmaticus	Asthma
9hA1.00	excepted from asthma quality indicators: patient unsuitable	Asthma
9OJZ.00	asthma monitoring admin.nos	Asthma
66Yq.00	asthma causes night time symptoms 1 to 2 times per week	Asthma
66Yr.00	asthma causes symptoms most nights	Asthma
663P000	asthma limits activities 1 to 2 times per month	Asthma
663P100	asthma limits activities 1 to 2 times per week	Asthma
663P200	asthma limits activities most days	Asthma
178A.00	asthma trigger - airborne dust	Asthma
178B.00	asthma trigger - exercise	Asthma
1781.00	asthma trigger - pollen	Asthma
1782.00	asthma trigger - tobacco smoke	Asthma
1783.00	asthma trigger - warm air	Asthma
679J200	health education - structured patient focused asthma discuss	Asthma
66Yp.00	asthma review using royal college of physicians three questions	Asthma
388t000	royal college physician asthma assessment 3 question score	Asthma
66Ys.00	asthma never causes night symptoms	Asthma
178..00	asthma trigger	Asthma
679J.00	health education - asthma	Asthma
66YQ.00	asthma monitoring by nurse	Asthma
66YR.00	asthma monitoring by doctor	Asthma
1O2..00	asthma confirmed	Asthma
66Yu.00	number days absent from school due to asthma in past 6 month	Asthma
661N100	asthma self-management plan review	Asthma
661M100	asthma self-management plan agreed	Asthma
H331000	intrinsic asthma without status asthmaticus	Asthma
9OJ..00	asthma monitoring admin.	Asthma

663m.00	asthma accident and emergency attendance since last visit	Asthma
H335.00	chronic asthma with fixed airflow obstruction	Asthma
66YZ.00	does not have asthma management plan	Asthma
8CR0.00	asthma clinical management plan	Asthma
13Y4.00	asthma society member	Asthma
14B4.00	h/o: asthma	Asthma
663N.00	asthma disturbing sleep	Asthma
663N000	asthma causing night waking	Asthma
663N100	asthma disturbs sleep weekly	Asthma
663N200	asthma disturbs sleep frequently	Asthma
663O.00	asthma not disturbing sleep	Asthma
663O000	asthma never disturbs sleep	Asthma
663P.00	asthma limiting activities	Asthma
663Q.00	asthma not limiting activities	Asthma
663U.00	asthma management plan given	Asthma
663V.00	asthma severity	Asthma
663W.00	asthma prophylactic medication used	Asthma
663d.00	emergency asthma admission since last appointment	Asthma
663e.00	asthma restricts exercise	Asthma
663e000	asthma sometimes restricts exercise	Asthma
663e100	asthma severely restricts exercise	Asthma
663f.00	asthma never restricts exercise	Asthma
8793.00	asthma control step 0	Asthma
8794.00	asthma control step 1	Asthma
8795.00	asthma control step 2	Asthma
8796.00	asthma control step 3	Asthma
8797.00	asthma control step 4	Asthma
8798.00	asthma control step 5	Asthma
8CE2.00	asthma leaflet given	Asthma
8H2P.00	emergency admission, asthma	Asthma
9N1d.00	seen in asthma clinic	Asthma
9OJ1.00	attends asthma monitoring	Asthma
9OJ2.00	refuses asthma monitoring	Asthma
9OJ3.00	asthma monitor offer default	Asthma
9OJ9.00	asthma monitoring deleted	Asthma
9NI8.00	asthma outreach clinic	Asthma
H312000	chronic asthmatic bronchitis	Asthma
H33..11	bronchial asthma	Asthma
H33..00	asthma	Asthma
H330z00	extrinsic asthma nos	Asthma
H331z00	intrinsic asthma nos	Asthma
H332.00	mixed asthma	Asthma
H33z.00	asthma unspecified	Asthma
1786.00	asthma trigger - animals	Asthma
H334.00	brittle asthma	Asthma
H330.12	childhood asthma	Asthma

H331.11	late onset asthma	Asthma
H33z200	late-onset asthma	Asthma
H330111	extrinsic asthma with asthma attack	Asthma
H33zz12	allergic asthma nec	Asthma
H330011	hay fever with asthma	Asthma
H330.14	pollen asthma	Asthma
H331111	intrinsic asthma with asthma attack	Asthma
H331.00	intrinsic asthma	Asthma
H33z000	status asthmaticus nos	Asthma
H33z100	asthma attack	Asthma
H33zz00	asthma nos	Asthma
9OJA.11	asthma monitored	Asthma
9OJA.00	asthma monitoring check done	Asthma
H33z111	asthma attack nos	Asthma
663..11	asthma monitoring	Asthma
H333.00	acute exacerbation of asthma	Asthma
663j.00	asthma - currently active	Asthma
663h.00	asthma - currently dormant	Asthma
9OJ4.00	asthma monitor 1st letter	Asthma
9OJ5.00	asthma monitor 2nd letter	Asthma
9OJ6.00	asthma monitor 3rd letter	Asthma
9OJ8.00	asthma monitor phone invite	Asthma
9OJ7.00	asthma monitor verbal invite	Asthma
1788.00	asthma trigger - cold air	Asthma
1785.00	asthma trigger - damp	Asthma
1784.00	asthma trigger - emotion	Asthma
1789.00	asthma trigger - respiratory infection	Asthma
1787.00	asthma trigger - seasonal	Asthma
173A.00	exercise induced asthma	Asthma
H33zz11	exercise induced asthma	Asthma
H330.00	extrinsic (atopic) asthma	Asthma
H47y000	detergent asthma	Asthma
8791.00	further asthma - drug prevent.	Asthma
H330.13	hay fever with asthma	Asthma
1780.00	aspirin induced asthma	Asthma
H35y700	wood asthma	Asthma
173c.00	occupational asthma	Asthma
173d.00	work aggravated asthma	Asthma
H331100	intrinsic asthma with status asthmaticus	Asthma
66Yp.00	asthma review using roy colleg of physicians three questions	Asthma
8H2P.00	\emergency admission, asthma\""	Asthma
D000.11	normocytic anaemia due to chronic blood loss	Blood loss anaemia
D211.11	normocytic anaemia following acute bleed	Blood loss anaemia
D000.12	iron deficiency anaemia due to blood loss	Blood loss anaemia
D000.00	iron deficiency anaemia due to chronic blood loss	Blood loss anaemia
B226.00	mesothelioma	Cancer

B471100	teratoma of descended testis	Cancer
B47z.12	teratoma of testis	Cancer
B470300	teratoma of undescended testis	Cancer
4M23.00	lymphoma stage iv	Cancer
B933.11	cystosarcoma phyllodes	Cancer
B50..00	malignant neoplasm of eye	Cancer
B540000	malignant neoplasm of adrenal cortex	Cancer
B540100	malignant neoplasm of adrenal medulla	Cancer
B43..00	malignant neoplasm of body of uterus	Cancer
B453.00	malignant neoplasm of clitoris	Cancer
B34..00	malignant neoplasm of female breast	Cancer
B600.00	reticulosarcoma	Cancer
B576000	secondary malignant neoplasm of retroperitoneum	Cancer
B106.00	malignant neoplasm, overlapping lesion of oesophagus	Cancer
BB2C.00	[m]squamous cell carcinoma, keratinising type nos	Cancer
BB5R600	[m]mucocarcinoid tumour, malignant	Cancer
ZV10016	[v]personal history of malignant neoplasm of oesophagus	Cancer
ZV10611	[v]personal history of lymphoid leukaemia	Cancer
ZV10613	[v]personal history of myeloid leukaemia	Cancer
ZV10612	[v]personal history of monocytic leukaemia	Cancer
ZV10713	[v]personal history of other haematopoietic neoplasm	Cancer
BBe9.00	[m]triton tumour, malignant	Cancer
B134.00	malignant neoplasm of caecum	Cancer
B10z.11	oesophageal cancer	Cancer
B10..00	malignant neoplasm of oesophagus	Cancer
BBTK.00	[m]epithelioid haemangioendothelioma, malignant	Cancer
BBEV.00	[m]blue naevus, malignant	Cancer
BBr2700	[m]adult t-cell leukaemia/lymphoma	Cancer
B801200	carcinoma in situ of lower 1/3 oesophagus	Cancer
B801100	carcinoma in situ of middle 1/3 oesophagus	Cancer
B801000	carcinoma in situ of upper 1/3 oesophagus	Cancer
B084.00	malignant neoplasm, overlapping lesion of hypopharynx	Cancer
B508.00	malignant neoplasm, overlapping lesion of eye and adnexa	Cancer
B074.00	malignant neoplasm, overlapping lesion of nasopharynx	Cancer
B214.00	malignant neoplasm, overlapping lesion of larynx	Cancer
B017.00	malignant overlapping lesion of tongue	Cancer
B042.00	malignant neoplasm, overlapping lesion of floor of mouth	Cancer
B117.00	malignant neoplasm, overlapping lesion of stomach	Cancer
B124.00	malignant neoplasm, overlapping lesion of small intestine	Cancer
B163.00	malignant neoplasm, overlapping lesion of biliary tract	Cancer
B175.00	malignant neoplasm, overlapping lesion of pancreas	Cancer
B487.00	malignant neoplasm, overlapping lesion of penis	Cancer
B627600	diffuse non-hodgkin's immunoblastic (diffuse) lymphoma	Cancer
BB5S200	[m]bronchiolo-alveolar adenocarcinoma	Cancer
B614.00	hodgkin's disease, nodular sclerosis	Cancer
BBv2.00	[m]angiocentrict-cell lymphoma	Cancer

BBm3.00	[m]letterer - siwe disease	Cancer
BBm4.00	[m]true histiocytic lymphoma	Cancer
BBj..00	[m]hodgkin's disease	Cancer
F122.00	malignant neuroleptic syndrome	Cancer
BB5D800	[m]hepatocellular carcinoma, fibrolamellar	Cancer
BBK0700	[m]myxoid leiomyosarcoma	Cancer
BBEG000	[m]acral lentiginous melanoma, malignant	Cancer
BBLG.00	[m]carcinoma in pleomorphic adenoma	Cancer
BB5f111	[m]follicular carcinoma	Cancer
BBgC.11	[m]lymphocytic lymphoma nos	Cancer
BBgC.12	[m]lymphocytic lymphosarcoma nos	Cancer
BBgG.12	[m]lymphoblastic lymphoma nos	Cancer
BBgJ.11	[m]germinoblastic sarcoma nos	Cancer
BBg7.00	[m]malignant lymphoma, lymphoplasmacytoid type	Cancer
BBQ7200	[m]teratoma, malignant, nos	Cancer
BB1N.00	[m]small cell-large cell carcinoma	Cancer
BBV1.00	[m]osteosarcoma nos	Cancer
BB5D500	[m]hepatocellular carcinoma nos	Cancer
BBmD.00	[m] cutaneous lymphoma	Cancer
BBH1.00	[m]myxosarcoma	Cancer
BBLE.00	[m]adenosarcoma	Cancer
ZV10013	[v]personal history of malignant neoplasm of intestine	Cancer
ZV10018	[v]personal history of malignant neoplasm of stomach	Cancer
ZV10017	[v]personal history of malignant neoplasm of rectum	Cancer
ZV10011	[v]personal history of malignant neoplasm of anus	Cancer
ZV10019	[v]personal history of malignant neoplasm of tongue	Cancer
ZV10014	[v]personal history of malignant neoplasm of large intestine	Cancer
ZV10015	[v]personal history of malignant neoplasm of liver	Cancer
ZV10111	[v]personal history of malignant neoplasm of bronchus	Cancer
ZV10112	[v]personal history of malignant neoplasm of lung	Cancer
ZV10113	[v]personal history of malignant neoplasm of trachea	Cancer
ZV10213	[v]personal history of malignant neoplasm of middle ear	Cancer
ZV10211	[v]personal history of malignant neoplasm - accessory sinus	Cancer
ZV10212	[v]personal history of malignant neoplasm of larynx	Cancer
ZV10214	[v]personal history of malignant neoplasm of nose	Cancer
ZV10416	[v]personal history of malignant neoplasm of testis	Cancer
ZV10417	[v]personal history of malignant neoplasm of uterine body	Cancer
ZV10415	[v]personal history of malignant neoplasm of prostate	Cancer
ZV10414	[v]personal history of malignant neoplasm of ovary	Cancer
ZV10411	[v]personal history of malignant neoplasm of cervix uteri	Cancer
ZV10511	[v]personal history of malignant neoplasm of bladder	Cancer
ZV10513	[v]personal history of malignant neoplasm of kidney	Cancer
ZV10711	[v]personal history of hodgkin's disease	Cancer
ZV10712	[v]personal history of lymphosarcoma	Cancer
ZV10714	[v]personal history of reticulosarcoma	Cancer
ZV10y14	[v]personal history of malignant neoplasm of skin	Cancer

ZV10y11	[v]personal history of malignant neoplasm of bone	Cancer
ZV10y12	[v]personal history of malignant neoplasm of brain	Cancer
ZV10y15	[v]personal history of malignant neoplasm of thyroid	Cancer
ZV10y13	[v]personal history of malignant neoplasm of eye	Cancer
BB5y000	[m]basal cell adenocarcinoma	Cancer
BB91100	[m]infiltrating duct and lobular carcinoma	Cancer
BBrA312	[m]granulocytic sarcoma	Cancer
B41..00	malignant neoplasm of cervix uteri	Cancer
B000.00	malignant neoplasm of upper lip, vermilion border	Cancer
B001100	malignant neoplasm of lower lip, lipstick area	Cancer
B010.11	malignant neoplasm of posterior third of tongue	Cancer
B02..00	malignant neoplasm of major salivary glands	Cancer
B021.00	malignant neoplasm of submandibular gland	Cancer
B055100	malignant neoplasm of roof of mouth	Cancer
B060100	malignant neoplasm of palatine tonsil	Cancer
B060000	malignant neoplasm of faucial tonsil	Cancer
B070.00	malignant neoplasm of roof of nasopharynx	Cancer
B180.00	malignant neoplasm of retroperitoneum	Cancer
B200.00	malignant neoplasm of nasal cavities	Cancer
B203.00	malignant neoplasm of ethmoid sinus	Cancer
B310000	malignant neoplasm of soft tissue of head	Cancer
B310100	malignant neoplasm of soft tissue of face	Cancer
B310200	malignant neoplasm of soft tissue of neck	Cancer
B440.11	cancer of ovary	Cancer
B480.00	malignant neoplasm of prepuce (foreskin)	Cancer
BBj7.00	[m]hodgkin's disease, nodular sclerosis, cellular phase	Cancer
BBE1000	[m]malignant melanoma, regressing	Cancer
BBk7.00	[m]malignant lymphoma, centroblastic type, follicular	Cancer
BBj2.00	[m]hodgkin's disease, mixed cellularity	Cancer
BBEG.00	[m]malignant melanoma in hutchinson's melanotic freckle	Cancer
BBR2.00	[m]choriocarcinoma	Cancer
BBgR.00	[m]malignant lymphoma, large cell, diffuse nos	Cancer
BB5L300	[m]adenocarcinoma in multiple adenomatous polyps	Cancer
BB51000	[m]adenocarcinoma in situ in villous adenoma	Cancer
BBl1.00	[m]sezary's disease	Cancer
BBTA.00	[m]kaposi's sarcoma	Cancer
BBgP.00	[m]malignant lymphoma, mixed small and large cell, diffuse	Cancer
BBp2.00	[m]malignant mastocytosis	Cancer
BB5RA00	[m]merkel cell carcinoma	Cancer
BB51100	[m]adenocarcinoma in situ in tubulovillous adenoma	Cancer
BBE1100	[m]desmoplastic melanoma, malignant	Cancer
BB5D700	[m]combined hepatocellular carcinoma and cholangiocarcinoma	Cancer
BBG1.00	[m]fibrosarcoma nos	Cancer
BB9K000	[m]paget's disease and intraductal carcinoma of breast	Cancer
BB69100	[m]sebaceous adenocarcinoma	Cancer
BB5R900	[m]neuroendocrine carcinoma	Cancer

BB52000	[m]adenocarcinoma in tubulovillous adenoma	Cancer
BB29.00	[m]squamous cell carcinoma in situ nos	Cancer
BB1M.00	[m]small cell carcinoma, intermediate cell	Cancer
BB5L200	[m]adenocarcinoma in situ in adenomatous polyp	Cancer
BBGP.00	[m]pigmented dermatofibrosarcoma protuberans	Cancer
BBN1.00	[m]synovial sarcoma nos	Cancer
BB91000	[m]intraductal papillary adenocarcinoma with invasion	Cancer
BBgL.00	[m]malignant lymphoma, small lymphocytic nos	Cancer
BBJH.00	[m]dedifferentiated liposarcoma	Cancer
BB14.00	[m]carcinomatosis	Cancer
BBj1100	[m]hodgkin,s disease, lymphocytic predominance, nodular	Cancer
BBj9.00	[m]hodgkin's granuloma	Cancer
F373.00	polyneuropathy in malignant disease	Cancer
BBEX.00	[m]melanoma in situ	Cancer
BBg8.00	[m]malignant lymphoma, immunoblastic type	Cancer
BBLH.00	[m]rhabdoid sarcoma	Cancer
BB01.00	[m]neoplasm, uncertain whether benign or malignant	Cancer
BBF4.00	[m]giant cell sarcoma (except of bone)	Cancer
BBEN.11	[m]juvenila melanoma	Cancer
BBf2.00	[m]alveolar soft part sarcoma	Cancer
B823000	carcinoma in situ of skin of forehead skin	Cancer
B323000	malignant melanoma of external surface of cheek	Cancer
B323200	malignant melanoma of eyebrow	Cancer
B323400	malignant melanoma of external surface of nose	Cancer
B585.00	secondary malignant neoplasm of bone and bone marrow	Cancer
B576100	secondary malignant neoplasm of peritoneum	Cancer
BBB7.00	[m]epithelial-myoepithelial carcinoma	Cancer
B62F100	mantle cell lymphoma	Cancer
4M21.00	lymphoma stage ii	Cancer
B674.00	acute panmyelosis	Cancer
H51y700	malignant pleural effusion	Cancer
4M70.00	clark melanoma level 1	Cancer
B630100	solitary myeloma	Cancer
A220.11	malignant pustule	Cancer
1925NB	neuroblastoma	Cancer
B622.00	sezary's disease	Cancer
B622500	sezary's disease of lymph nodes of inguinal region and leg	Cancer
B622700	sezary's disease of spleen	Cancer
44a4.00	squamous cell carcinoma antigen level	Cancer
1O0..00	cancer confirmed	Cancer
8CP0.00	cancer care plan discussed with patient	Cancer
B575z00	secondary malig neop of large intestine or rectum nos	Cancer
B57..00	secondary malig neop of respiratory and digestive systems	Cancer
B57z.00	secondary malig neop of respiratory or digestive system nos	Cancer
B576.00	secondary malig neop of retroperitoneum and peritoneum	Cancer
B576z00	secondary malig neop of retroperitoneum or peritoneum nos	Cancer

B574z00	secondary malig neop of small intestine or duodenum nos	Cancer
B575.00	secondary malignant neoplasm of large intestine and rectum	Cancer
B153.00	secondary malignant neoplasm of liver	Cancer
B58yz00	secondary malignant neoplasm of other specified site nos	Cancer
B58..00	secondary malignant neoplasm of other specified sites	Cancer
B574.00	secondary malignant neoplasm of small intestine and duodenum	Cancer
B560z00	secondary unspec malig neop lymph nodes head/face/neck nos	Cancer
B8yy100	carcinoma in situ of adrenal gland	Cancer
B808600	carcinoma in situ of ampulla of vater	Cancer
B805.00	carcinoma in situ of anal canal	Cancer
B803500	carcinoma in situ of appendix	Cancer
B803600	carcinoma in situ of ascending colon	Cancer
B837.00	carcinoma in situ of bladder	Cancer
B802200	carcinoma in situ of body of stomach	Cancer
B832.11	carcinoma in situ of body of uterus	Cancer
B802000	carcinoma in situ of cardia of stomach	Cancer
B831.00	carcinoma in situ of cervix uteri	Cancer
B803.00	carcinoma in situ of colon	Cancer
B808500	carcinoma in situ of common bile duct	Cancer
B808400	carcinoma in situ of cystic duct	Cancer
B803200	carcinoma in situ of descending colon	Cancer
B807000	carcinoma in situ of duodenum	Cancer
B831000	carcinoma in situ of endocervix	Cancer
B832000	carcinoma in situ of endometrium	Cancer
B810200	carcinoma in situ of epiglottis	Cancer
B81y700	carcinoma in situ of ethmoidal sinus	Cancer
B81y400	carcinoma in situ of eustachian tube	Cancer
B831100	carcinoma in situ of exocervix	Cancer
B8y0.00	carcinoma in situ of eye	Cancer
B833100	carcinoma in situ of fallopian tube	Cancer
B800400	carcinoma in situ of floor of mouth	Cancer
B802100	carcinoma in situ of fundus of stomach	Cancer
B810811	carcinoma in situ of glottis	Cancer
B803000	carcinoma in situ of hepatic flexure of colon	Cancer
B800900	carcinoma in situ of hypopharynx	Cancer
B807200	carcinoma in situ of ileum	Cancer
B808100	carcinoma in situ of intrahepatic bile ducts	Cancer
B807100	carcinoma in situ of jejunum	Cancer
B810.00	carcinoma in situ of larynx	Cancer
B800000	carcinoma in situ of lip	Cancer
B808000	carcinoma in situ of liver	Cancer
B812100	carcinoma in situ of main bronchus	Cancer
B81y500	carcinoma in situ of mastoid air cells	Cancer
B81y600	carcinoma in situ of maxillary sinus	Cancer
B807300	carcinoma in situ of meckel's diverticulum	Cancer
B81y100	carcinoma in situ of nasal cavity	Cancer

B800700	carcinoma in situ of nasopharynx	Cancer
B800800	carcinoma in situ of oropharynx	Cancer
B833000	carcinoma in situ of ovary	Cancer
B800600	carcinoma in situ of palate	Cancer
B80z000	carcinoma in situ of pancreas	Cancer
B8yy200	carcinoma in situ of parathyroid gland	Cancer
B835.00	carcinoma in situ of penis	Cancer
B825800	carcinoma in situ of perianal skin	Cancer
B800.12	carcinoma in situ of pharynx	Cancer
B8yy300	carcinoma in situ of pituitary gland	Cancer
B81y000	carcinoma in situ of pleura	Cancer
B834.00	carcinoma in situ of prostate	Cancer
B802300	carcinoma in situ of pyloric antrum	Cancer
B804000	carcinoma in situ of rectosigmoid junction	Cancer
B804100	carcinoma in situ of rectum	Cancer
B836300	carcinoma in situ of scrotum	Cancer
B803300	carcinoma in situ of sigmoid colon	Cancer
B825200	carcinoma in situ of skin of axilla	Cancer
B825300	carcinoma in situ of skin of back	Cancer
B825000	carcinoma in situ of skin of breast	Cancer
B825700	carcinoma in situ of skin of buttock	Cancer
B823300	carcinoma in situ of skin of cheek	Cancer
B823100	carcinoma in situ of skin of eyebrow	Cancer
B827400	carcinoma in situ of skin of foot	Cancer
B825500	carcinoma in situ of skin of groin	Cancer
B826300	carcinoma in situ of skin of hand	Cancer
B827000	carcinoma in situ of skin of hip	Cancer
B827200	carcinoma in situ of skin of knee	Cancer
B820.00	carcinoma in situ of skin of lip	Cancer
B824100	carcinoma in situ of skin of neck	Cancer
B823400	carcinoma in situ of skin of nose	Cancer
B825600	carcinoma in situ of skin of perineum	Cancer
B826000	carcinoma in situ of skin of shoulder	Cancer
B827100	carcinoma in situ of skin of thigh	Cancer
B82..00	carcinoma in situ of skin	Cancer
B81y900	carcinoma in situ of sphenoidal sinus	Cancer
B80z100	carcinoma in situ of spleen	Cancer
B802.00	carcinoma in situ of stomach	Cancer
B836000	carcinoma in situ of testis	Cancer
B8yy000	carcinoma in situ of thyroid gland	Cancer
B800100	carcinoma in situ of tongue	Cancer
B811.00	carcinoma in situ of trachea	Cancer
B803100	carcinoma in situ of transverse colon	Cancer
B833200	carcinoma in situ of vagina	Cancer
B833300	carcinoma in situ of vulva	Cancer
B625300	letterer-siwe disease of intra-abdominal lymph nodes	Cancer

B625600	letterer-siwe disease of intrapelvic lymph nodes	Cancer
B625200	letterer-siwe disease of intrathoracic lymph nodes	Cancer
B625800	letterer-siwe disease of lymph nodes of multiple sites	Cancer
B625700	letterer-siwe disease of spleen	Cancer
B623300	malignant histiocytosis of intra-abdominal lymph nodes	Cancer
B623600	malignant histiocytosis of intrapelvic lymph nodes	Cancer
B623200	malignant histiocytosis of intrathoracic lymph nodes	Cancer
B623800	malignant histiocytosis of lymph nodes of multiple sites	Cancer
B623700	malignant histiocytosis of spleen	Cancer
B325400	malignant melanoma of perianal skin	Cancer
B615700	hodgkin's disease, mixed cellularity of spleen	Cancer
B614700	hodgkin's disease, nodular sclerosis of spleen	Cancer
B611600	hodgkin's granuloma of intrapelvic lymph nodes	Cancer
B611200	hodgkin's granuloma of intrathoracic lymph nodes	Cancer
B611700	hodgkin's granuloma of spleen	Cancer
B610700	hodgkin's paragranuloma of spleen	Cancer
B612300	hodgkin's sarcoma of intra-abdominal lymph nodes	Cancer
B612600	hodgkin's sarcoma of intrapelvic lymph nodes	Cancer
B612200	hodgkin's sarcoma of intrathoracic lymph nodes	Cancer
B612700	hodgkin's sarcoma of spleen	Cancer
B32..00	malignant melanoma of skin	Cancer
B304100	malignant neoplasm of acromion	Cancer
B540.00	malignant neoplasm of adrenal gland	Cancer
B142.00	malignant neoplasm of anal canal	Cancer
B242.00	malignant neoplasm of anterior mediastinum	Cancer
B545100	malignant neoplasm of aortic body	Cancer
B340100	malignant neoplasm of areola of female breast	Cancer
B350100	malignant neoplasm of areola of male breast	Cancer
B442.00	malignant neoplasm of broad ligament	Cancer
B544.00	malignant neoplasm of carotid body	Cancer
B516.00	malignant neoplasm of cerebellum	Cancer
B303200	malignant neoplasm of clavicle	Cancer
B545200	malignant neoplasm of coccygeal body	Cancer
B308600	malignant neoplasm of cuboid	Cancer
B161000	malignant neoplasm of cystic duct	Cancer
B313100	malignant neoplasm of diaphragm	Cancer
B011.00	malignant neoplasm of dorsal surface of tongue	Cancer
B410.00	malignant neoplasm of endocervix	Cancer
B241100	malignant neoplasm of epicardium	Cancer
B300000	malignant neoplasm of ethmoid bone	Cancer
B411.00	malignant neoplasm of exocervix	Cancer
B307000	malignant neoplasm of femur	Cancer
B307100	malignant neoplasm of fibula	Cancer
B300100	malignant neoplasm of frontal bone	Cancer
B545000	malignant neoplasm of glomus jugulare	Cancer
B313200	malignant neoplasm of great vessels	Cancer

B221100	malignant neoplasm of hilus of lung	Cancer
B306000	malignant neoplasm of ilium	Cancer
B151.00	malignant neoplasm of intrahepatic bile ducts	Cancer
B306100	malignant neoplasm of ischium	Cancer
B066.00	malignant neoplasm of lateral wall of oropharynx	Cancer
B221.00	malignant neoplasm of main bronchus	Cancer
B35..00	malignant neoplasm of male breast	Cancer
B301.00	malignant neoplasm of mandible	Cancer
B300A00	malignant neoplasm of maxilla	Cancer
B300300	malignant neoplasm of nasal bone	Cancer
B340000	malignant neoplasm of nipple of female breast	Cancer
B350000	malignant neoplasm of nipple of male breast	Cancer
B300400	malignant neoplasm of occipital bone	Cancer
B520100	malignant neoplasm of optic nerve	Cancer
B443.00	malignant neoplasm of parametrium	Cancer
B300600	malignant neoplasm of parietal bone	Cancer
B18y400	malignant neoplasm of parietal peritoneum	Cancer
B230.00	malignant neoplasm of parietal pleura	Cancer
B308000	malignant neoplasm of patella	Cancer
B18y500	malignant neoplasm of pelvic peritoneum	Cancer
B180000	malignant neoplasm of periadrenal tissue	Cancer
B335900	malignant neoplasm of perianal skin	Cancer
B241300	malignant neoplasm of pericardium	Cancer
B42..00	malignant neoplasm of placenta	Cancer
B243.00	malignant neoplasm of posterior mediastinum	Cancer
B553100	malignant neoplasm of presacral region	Cancer
B306200	malignant neoplasm of pubis	Cancer
B304300	malignant neoplasm of radius	Cancer
B141.00	malignant neoplasm of rectum	Cancer
B303000	malignant neoplasm of rib	Cancer
B553200	malignant neoplasm of sacrococcygeal region	Cancer
B304000	malignant neoplasm of scapula	Cancer
B337500	malignant neoplasm of skin of ankle	Cancer
B335700	malignant neoplasm of skin of back	Cancer
B335200	malignant neoplasm of skin of breast	Cancer
B335800	malignant neoplasm of skin of buttock	Cancer
B333100	malignant neoplasm of skin of chin	Cancer
B333200	malignant neoplasm of skin of eyebrow	Cancer
B336400	malignant neoplasm of skin of finger	Cancer
B337700	malignant neoplasm of skin of foot	Cancer
B333300	malignant neoplasm of skin of forehead	Cancer
B335500	malignant neoplasm of skin of groin	Cancer
B336300	malignant neoplasm of skin of hand	Cancer
B337000	malignant neoplasm of skin of hip	Cancer
B337200	malignant neoplasm of skin of knee	Cancer
B330.00	malignant neoplasm of skin of lip	Cancer

B334100	malignant neoplasm of skin of neck	Cancer
B335600	malignant neoplasm of skin of perineum	Cancer
B336000	malignant neoplasm of skin of shoulder	Cancer
B337100	malignant neoplasm of skin of thigh	Cancer
B337800	malignant neoplasm of skin of toe	Cancer
B335400	malignant neoplasm of skin of umbilicus	Cancer
B300700	malignant neoplasm of sphenoid bone	Cancer
B303100	malignant neoplasm of sternum	Cancer
B308100	malignant neoplasm of talus	Cancer
B300800	malignant neoplasm of temporal bone	Cancer
B307200	malignant neoplasm of tibia	Cancer
B304400	malignant neoplasm of ulna	Cancer
B013.00	malignant neoplasm of ventral surface of tongue	Cancer
B231.00	malignant neoplasm of visceral pleura	Cancer
B300C00	malignant neoplasm of vomer	Cancer
B300900	malignant neoplasm of zygomatic bone	Cancer
B587.00	secondary malignant neoplasm of adrenal gland	Cancer
B581100	secondary malignant neoplasm of bladder	Cancer
B583000	secondary malignant neoplasm of brain	Cancer
B575000	secondary malignant neoplasm of colon	Cancer
B574000	secondary malignant neoplasm of duodenum	Cancer
B574200	secondary malignant neoplasm of ileum	Cancer
B574100	secondary malignant neoplasm of jejunum	Cancer
B580.00	secondary malignant neoplasm of kidney	Cancer
B577.00	secondary malignant neoplasm of liver	Cancer
B570.00	secondary malignant neoplasm of lung	Cancer
B571.00	secondary malignant neoplasm of mediastinum	Cancer
B586.00	secondary malignant neoplasm of ovary	Cancer
B58y700	secondary malignant neoplasm of penis	Cancer
B572.00	secondary malignant neoplasm of pleura	Cancer
B58y500	secondary malignant neoplasm of prostate	Cancer
B575100	secondary malignant neoplasm of rectum	Cancer
B582600	secondary malignant neoplasm of skin of breast	Cancer
B582100	secondary malignant neoplasm of skin of face	Cancer
B582200	secondary malignant neoplasm of skin of neck	Cancer
B582300	secondary malignant neoplasm of skin of trunk	Cancer
B582.00	secondary malignant neoplasm of skin	Cancer
B583100	secondary malignant neoplasm of spinal cord	Cancer
B58y600	secondary malignant neoplasm of testis	Cancer
B58y900	secondary malignant neoplasm of tongue	Cancer
B581000	secondary malignant neoplasm of ureter	Cancer
B581200	secondary malignant neoplasm of urethra	Cancer
B58y100	secondary malignant neoplasm of uterus	Cancer
B58y300	secondary malignant neoplasm of vagina	Cancer
B58y400	secondary malignant neoplasm of vulva	Cancer
B621300	mycosis fungoides of intra-abdominal lymph nodes	Cancer

B621600	mycosis fungoides of intrapelvic lymph nodes	Cancer
B621200	mycosis fungoides of intrathoracic lymph nodes	Cancer
B621400	mycosis fungoides of lymph nodes of axilla and upper limb	Cancer
B621700	mycosis fungoides of spleen	Cancer
B653.00	myeloid sarcoma	Cancer
B561300	secondary and unspec malig neop ant mediastinal lymph nodes	Cancer
B560800	secondary and unspec malig neop anterior cervical ln	Cancer
B563.00	secondary and unspec malig neop axilla and upper limb ln	Cancer
B563z00	secondary and unspec malig neop axilla and upper limb ln nos	Cancer
B563000	secondary and unspec malig neop axillary lymph nodes	Cancer
B561800	secondary and unspec malig neop bronchopulmonary lymph nodes	Cancer
B565200	secondary and unspec malig neop circumflex iliac ln	Cancer
B562000	secondary and unspec malig neop coeliac lymph nodes	Cancer
B562300	secondary and unspec malig neop common iliac lymph nodes	Cancer
B560900	secondary and unspec malig neop deep cervical ln	Cancer
B564100	secondary and unspec malig neop deep inguinal lymph nodes	Cancer
B560400	secondary and unspec malig neop deep parotid lymph nodes	Cancer
B561200	secondary and unspec malig neop diaphragmatic lymph nodes	Cancer
B562400	secondary and unspec malig neop external iliac lymph nodes	Cancer
B565100	secondary and unspec malig neop inferior epigastric ln	Cancer
B562200	secondary and unspec malig neop inferior mesenteric ln	Cancer
B561700	secondary and unspec malig neop inferior tracheobronchial ln	Cancer
B563200	secondary and unspec malig neop infraclavicular lymph nodes	Cancer
B564.00	secondary and unspec malig neop inguinal and lower limb ln	Cancer
B561100	secondary and unspec malig neop intercostal lymph nodes	Cancer
B565000	secondary and unspec malig neop internal iliac lymph nodes	Cancer
B620300	nodular lymphoma of intra-abdominal lymph nodes	Cancer
B620600	nodular lymphoma of intrapelvic lymph nodes	Cancer
B620200	nodular lymphoma of intrathoracic lymph nodes	Cancer
B561000	secondary and unspec malig neop internal mammary lymph nodes	Cancer
B620800	nodular lymphoma of lymph nodes of multiple sites	Cancer
B620700	nodular lymphoma of spleen	Cancer
B562z00	secondary and unspec malig neop intra-abdominal ln nos	Cancer
B562.00	secondary and unspec malig neop intra-abdominal lymph nodes	Cancer
B565z00	secondary and unspec malig neop intrapelvic ln nos	Cancer
B565.00	secondary and unspec malig neop intrapelvic lymph nodes	Cancer
B150.00	primary malignant neoplasm of liver	Cancer
B561z00	secondary and unspec malig neop intrathoracic ln nos	Cancer
B600300	reticulosarcoma of intra-abdominal lymph nodes	Cancer
B600600	reticulosarcoma of intrapelvic lymph nodes	Cancer
B600200	reticulosarcoma of intrathoracic lymph nodes	Cancer
B561.00	secondary and unspec malig neop intrathoracic lymph nodes	Cancer
B600800	reticulosarcoma of lymph nodes of multiple sites	Cancer
B600700	reticulosarcoma of spleen	Cancer
B560.00	secondary and unspec malig neop lymph nodes head/face/neck	Cancer
B56y.00	secondary and unspec malig neop lymph nodes multiple sites	Cancer

B56z.00	secondary and unspec malig neop lymph nodes nos	Cancer
B565400	secondary and unspec malig neop obturator lymph nodes	Cancer
B560600	secondary and unspec malig neop of facial lymph nodes	Cancer
B564z00	secondary and unspec malig neop of inguinal and leg ln nos	Cancer
B560000	secondary and unspec malig neop of superficial parotid ln	Cancer
B561500	secondary and unspec malig neop paratracheal lymph nodes	Cancer
B563300	secondary and unspec malig neop pectoral lymph nodes	Cancer
B564200	secondary and unspec malig neop popliteal lymph nodes	Cancer
B561400	secondary and unspec malig neop post mediastinal lymph nodes	Cancer
B561900	secondary and unspec malig neop pulmonary lymph nodes	Cancer
B565300	secondary and unspec malig neop sacral lymph nodes	Cancer
B560500	secondary and unspec malig neop submandibular lymph nodes	Cancer
B560700	secondary and unspec malig neop submental lymph nodes	Cancer
B561600	secondary and unspec malig neop superfic tracheobronchial ln	Cancer
B560200	secondary and unspec malig neop superficial cervical ln	Cancer
B564000	secondary and unspec malig neop superficial inguinal ln	Cancer
B562100	secondary and unspec malig neop superficial mesenteric ln	Cancer
B563100	secondary and unspec malig neop supratrochlear lymph nodes	Cancer
B560100	secondary and unspec malignant neoplasm mastoid lymph nodes	Cancer
B560300	secondary and unspec malignant neoplasm occipital lymph node	Cancer
B627A00	diffuse non-hodgkin's large cell lymphoma	Cancer
B620.11	reticulosarcoma - follicular or nodular	Cancer
B600500	reticulosarcoma of lymph nodes of inguinal region and leg	Cancer
4M3..00	breslow depth staging for melanoma	Cancer
B32y000	overlapping malignant melanoma of skin	Cancer
B828000	melanoma in situ of lip	Cancer
B828100	melanoma in situ of eyelid, including canthus	Cancer
B828700	melanoma in situ of scalp	Cancer
B828400	melanoma in situ of trunk	Cancer
B241400	mesothelioma of pericardium	Cancer
B33z000	kaposi's sarcoma of skin	Cancer
B05z000	kaposi's sarcoma of palate	Cancer
B6z0.00	kaposi's sarcoma of lymph nodes	Cancer
B142000	malignant neoplasm of cloacogenic zone	Cancer
B181.00	mesothelioma of peritoneum	Cancer
B471.00	malignant neoplasm of descended testis	Cancer
B830000	lobular carcinoma in situ of breast	Cancer
B830100	intraductal carcinoma in situ of breast	Cancer
1929GL	glioma	Cancer
BB3C.00	[m]superficial basal cell carcinoma	Cancer
BB3D.00	[m]basal cell carcinoma, nodular	Cancer
BB3E.00	[m]basal cell carcinoma, micronodular	Cancer
B4A..11	renal malignant neoplasm	Cancer
BB3F.00	[m]basal cell carcinoma, infiltrative	Cancer
BB3G.00	[m]pigmented basal cell carcinoma	Cancer
BBP3.11	[m]sarcomatoid mesothelioma	Cancer

B49..00	malignant neoplasm of urinary bladder	Cancer
B61..11	hodgkin lymphoma	Cancer
B618.00	nodular sclerosis classical hodgkin lymphoma	Cancer
B619.00	mixed cellularity classical hodgkin lymphoma	Cancer
B61C.00	other classical hodgkin lymphoma	Cancer
B61z.11	hodgkin lymphoma nos	Cancer
B628.00	follicular lymphoma	Cancer
B628000	follicular lymphoma grade 1	Cancer
B628100	follicular lymphoma grade 2	Cancer
B628200	follicular lymphoma grade 3	Cancer
B628600	cutaneous follicle centre lymphoma	Cancer
B628700	other types of follicular lymphoma	Cancer
B62A.00	sarcoma of dendritic cells	Cancer
B62E200	anaplastic large cell lymphoma, alk-negative	Cancer
B62E300	cutaneous t-cell lymphoma	Cancer
B62E700	subcutaneous panniculitic t-cell lymphoma	Cancer
B62E800	blastic nk-cell lymphoma	Cancer
B62Ew00	other mature t/nk-cell lymphoma	Cancer
B62F.00	nonfollicular lymphoma	Cancer
B62F000	small cell b-cell lymphoma	Cancer
B64y500	adult t-cell lymphoma/leukaemia (htlv-1-associated)	Cancer
B630400	solitary plasmacytoma	Cancer
BB1P.00	[m]non-small cell carcinoma	Cancer
4K2M.00	crv smr - hi grade dyskaryosis? invasive squamous carcinoma	Cancer
B627E00	diffuse large b-cell lymphoma	Cancer
B650.00	acute myeloid leukaemia	Cancer
B803400	carcinoma in situ of caecum	Cancer
B801.00	carcinoma in situ of oesophagus	Cancer
B641.00	chronic lymphoid leukaemia	Cancer
B651.00	chronic myeloid leukaemia	Cancer
B670.11	di guglielmo's disease	Cancer
B631.00	plasma cell leukaemia	Cancer
B64y100	prolymphocytic leukaemia	Cancer
B62E.00	t/nk-cell lymphoma	Cancer
B628300	follicular lymphoma grade 3a	Cancer
B628400	follicular lymphoma grade 3b	Cancer
B33..11	basal cell carcinoma	Cancer
BBGM.00	[m]dermatofibrosarcoma nos	Cancer
BBK3100	[m]rhabdomyosarcoma nos	Cancer
BBgM.00	[m]malignant lymphoma, small cleaved cell, diffuse	Cancer
BBj6.00	[m]hodgkin's disease, nodular sclerosis nos	Cancer
BBm1.00	[m]malignant histiocytosis	Cancer
BBm1.11	[m]malignant reticulosis	Cancer
B585000	pathological fracture due to metastatic bone disease	Cancer
B30z000	osteosarcoma	Cancer
B617.00	nodular lymphocyte predominant hodgkin lymphoma	Cancer

B62E100	anaplastic large cell lymphoma, alk-positive	Cancer
B62F200	lymphoblastic (diffuse) lymphoma	Cancer
C333011	waldenstrom macroglobulinaemia	Cancer
B62F.11	non-follicular lymphoma	Cancer
4D56.00	pleural fluid: malignant cells	Cancer
B630200	plasmacytoma nos	Cancer
B182.00	overlapping malign lesion of retroperitoneum and peritoneum	Cancer
B151100	malignant neoplasm of interlobular biliary canals	Cancer
B300B00	malignant neoplasm of turbinate	Cancer
B062200	malignant neoplasm of palatoglossal arch	Cancer
1425000	h/o malignant melanoma	Cancer
B660.00	acute monocytic leukaemia	Cancer
B62E900	angioimmunoblastic t-cell lymphoma	Cancer
B306500	malignant sacral teratoma	Cancer
4F32.00	ascitic fluid: malignant cells	Cancer
B151200	malignant neoplasm of intrahepatic biliary passages	Cancer
B6y0.00	myeloproliferative disorder	Cancer
B62x200	peripheral t-cell lymphoma	Cancer
B62D.00	histiocytic sarcoma	Cancer
B8...00	carcinoma in situ	Cancer
B616.00	hodgkin's disease, lymphocytic depletion	Cancer
B602.00	burkitt's lymphoma	Cancer
B621.00	mycosis fungoides	Cancer
B612.00	hodgkin's sarcoma	Cancer
B615.00	hodgkin's disease, mixed cellularity	Cancer
B61..00	hodgkin's disease	Cancer
B611.00	hodgkin's granuloma	Cancer
B51..00	malignant neoplasm of brain	Cancer
4M74.00	clark melanoma level 5	Cancer
7G03J00	excision of melanoma	Cancer
7H62z00	excision or biopsy of lymph node nos	Cancer
B627.11	non-hodgkin lymphoma	Cancer
A789600	hiv disease resulting in burkitt's lymphoma	Cancer
A789800	hiv disease resulting in multiple malignant neoplasms	Cancer
B61B.00	lymphocyte-rich classical hodgkin lymphoma	Cancer
B627G00	mediastinal (thymic) large b-cell lymphoma	Cancer
B62E500	hepatosplenic t-cell lymphoma	Cancer
B000100	malignant neoplasm of upper lip, lipstick area	Cancer
B000z00	malignant neoplasm of upper lip, vermilion border nos	Cancer
B001000	malignant neoplasm of lower lip, external	Cancer
B001z00	malignant neoplasm of lower lip, vermilion border nos	Cancer
B002300	malignant neoplasm of upper lip, oral aspect	Cancer
B002.00	malignant neoplasm of upper lip, inner aspect	Cancer
B002200	malignant neoplasm of upper lip, mucosa	Cancer
B002100	malignant neoplasm of upper lip, frenulum	Cancer
B002z00	malignant neoplasm of upper lip, inner aspect nos	Cancer

B003000	malignant neoplasm of lower lip, buccal aspect	Cancer
B003100	malignant neoplasm of lower lip, frenulum	Cancer
B003z00	malignant neoplasm of lower lip, inner aspect nos	Cancer
B004.00	malignant neoplasm of lip unspecified, inner aspect	Cancer
B004100	malignant neoplasm of lip unspecified, frenulum	Cancer
B004200	malignant neoplasm of lip unspecified, mucosa	Cancer
B004300	malignant neoplasm of lip, oral aspect	Cancer
B004z00	malignant neoplasm of lip, inner aspect nos	Cancer
B006.00	malignant neoplasm of overlapping lesion of lip	Cancer
B00y.00	malignant neoplasm of other sites of lip	Cancer
B00z.00	malignant neoplasm of vermilion border of lip unspecified	Cancer
B00z000	malignant neoplasm of lip, unspecified, external	Cancer
B00z100	malignant neoplasm of lip, unspecified, lipstick area	Cancer
B00zz00	malignant neoplasm of lip, vermilion border nos	Cancer
B010000	malignant neoplasm of base of tongue dorsal surface	Cancer
B010z00	malignant neoplasm of fixed part of tongue nos	Cancer
B011000	malignant neoplasm of anterior 2/3 of tongue dorsal surface	Cancer
B011100	malignant neoplasm of midline of tongue	Cancer
B011z00	malignant neoplasm of dorsum of tongue nos	Cancer
B012.00	malignant neoplasm of tongue, tip and lateral border	Cancer
B013000	malignant neoplasm of anterior 2/3 of tongue ventral surface	Cancer
B013100	malignant neoplasm of frenulum linguae	Cancer
B013z00	malignant neoplasm of ventral tongue surface nos	Cancer
B014.00	malignant neoplasm of anterior 2/3 of tongue unspecified	Cancer
B015.00	malignant neoplasm of tongue, junctional zone	Cancer
B01y.00	malignant neoplasm of other sites of tongue	Cancer
B01z.00	malignant neoplasm of tongue nos	Cancer
B02y.00	malignant neoplasm of other major salivary glands	Cancer
B02z.00	malignant neoplasm of major salivary gland nos	Cancer
B03y.00	malignant neoplasm of other sites of gum	Cancer
B03z.00	malignant neoplasm of gum nos	Cancer
B040.00	malignant neoplasm of anterior portion of floor of mouth	Cancer
B041.00	malignant neoplasm of lateral portion of floor of mouth	Cancer
B04y.00	malignant neoplasm of other sites of floor of mouth	Cancer
B04z.00	malignant neoplasm of floor of mouth nos	Cancer
B05..00	malignant neoplasm of other and unspecified parts of mouth	Cancer
B051.00	malignant neoplasm of vestibule of mouth	Cancer
B051000	malignant neoplasm of upper buccal sulcus	Cancer
B051100	malignant neoplasm of lower buccal sulcus	Cancer
B051200	malignant neoplasm of upper labial sulcus	Cancer
B051300	malignant neoplasm of lower labial sulcus	Cancer
B051z00	malignant neoplasm of vestibule of mouth nos	Cancer
B055.00	malignant neoplasm of palate unspecified	Cancer
B055000	malignant neoplasm of junction of hard and soft palate	Cancer
B055z00	malignant neoplasm of palate nos	Cancer
B057.00	overlapping lesion of other and unspecified parts of mouth	Cancer

B05y.00	malignant neoplasm of other specified mouth parts	Cancer
B05z.00	malignant neoplasm of mouth nos	Cancer
B060z00	malignant neoplasm tonsil nos	Cancer
B062000	malignant neoplasm of faucial pillar	Cancer
B062.00	malignant neoplasm of tonsillar pillar	Cancer
B062z00	malignant neoplasm of tonsillar fossa nos	Cancer
B064.00	malignant neoplasm of anterior epiglottis	Cancer
B064000	malignant neoplasm of epiglottis, free border	Cancer
B064100	malignant neoplasm of glossoepiglottic fold	Cancer
B064z00	malignant neoplasm of anterior epiglottis nos	Cancer
B065.00	malignant neoplasm of junctional region of epiglottis	Cancer
B067.00	malignant neoplasm of posterior wall of oropharynx	Cancer
B06y.00	malignant neoplasm of oropharynx, other specified sites	Cancer
B06yz00	malignant neoplasm of other specified site of oropharynx nos	Cancer
B06z.00	malignant neoplasm of oropharynx nos	Cancer
B07..00	malignant neoplasm of nasopharynx	Cancer
B071.00	malignant neoplasm of posterior wall of nasopharynx	Cancer
B071100	malignant neoplasm of pharyngeal tonsil	Cancer
B071000	malignant neoplasm of adenoid	Cancer
B071z00	malignant neoplasm of posterior wall of nasopharynx nos	Cancer
B072000	malignant neoplasm of pharyngeal recess	Cancer
B072100	malignant neoplasm of opening of auditory tube	Cancer
B072z00	malignant neoplasm of lateral wall of nasopharynx nos	Cancer
B073.00	malignant neoplasm of anterior wall of nasopharynx	Cancer
B073000	malignant neoplasm of floor of nasopharynx	Cancer
B073100	malignant neoplasm of nasopharyngeal soft palate surface	Cancer
B073z00	malignant neoplasm of anterior wall of nasopharynx nos	Cancer
B07y.00	malignant neoplasm of other specified site of nasopharynx	Cancer
B07z.00	malignant neoplasm of nasopharynx nos	Cancer
B083.00	malignant neoplasm of posterior pharynx	Cancer
B08y.00	malignant neoplasm of other specified hypopharyngeal site	Cancer
B08z.00	malignant neoplasm of hypopharynx nos	Cancer
B0z0.00	malignant neoplasm of pharynx unspecified	Cancer
B0z1.00	malignant neoplasm of waldeyer's ring	Cancer
B0zz.00	malignant neoplasm of lip, oral cavity and pharynx nos	Cancer
B100.00	malignant neoplasm of cervical oesophagus	Cancer
B101.00	malignant neoplasm of thoracic oesophagus	Cancer
B102.00	malignant neoplasm of abdominal oesophagus	Cancer
B103.00	malignant neoplasm of upper third of oesophagus	Cancer
B104.00	malignant neoplasm of middle third of oesophagus	Cancer
B105.00	malignant neoplasm of lower third of oesophagus	Cancer
B10y.00	malignant neoplasm of other specified part of oesophagus	Cancer
B110.00	malignant neoplasm of cardia of stomach	Cancer
B110000	malignant neoplasm of cardiac orifice of stomach	Cancer
B110111	malignant neoplasm of gastro-oesophageal junction	Cancer
B110100	malignant neoplasm of cardio-oesophageal junction of stomach	Cancer

B110z00	malignant neoplasm of cardia of stomach nos	Cancer
B111.00	malignant neoplasm of pylorus of stomach	Cancer
B111100	malignant neoplasm of pyloric canal of stomach	Cancer
B111z00	malignant neoplasm of pylorus of stomach nos	Cancer
B112.00	malignant neoplasm of pyloric antrum of stomach	Cancer
B113.00	malignant neoplasm of fundus of stomach	Cancer
B114.00	malignant neoplasm of body of stomach	Cancer
B115.00	malignant neoplasm of lesser curve of stomach unspecified	Cancer
B116.00	malignant neoplasm of greater curve of stomach unspecified	Cancer
B11y.00	malignant neoplasm of other specified site of stomach	Cancer
B11y000	malignant neoplasm of anterior wall of stomach nec	Cancer
B11y100	malignant neoplasm of posterior wall of stomach nec	Cancer
B11yz00	malignant neoplasm of other specified site of stomach nos	Cancer
B11z.00	malignant neoplasm of stomach nos	Cancer
B123.00	malignant neoplasm of meckel's diverticulum	Cancer
B12z.00	malignant neoplasm of small intestine nos	Cancer
B138.00	malignant neoplasm, overlapping lesion of colon	Cancer
B13y.00	malignant neoplasm of other specified sites of colon	Cancer
B14..00	malignant neoplasm of rectum, rectosigmoid junction and anus	Cancer
B143.00	malignant neoplasm of anus unspecified	Cancer
B15..00	malignant neoplasm of liver and intrahepatic bile ducts	Cancer
B150000	primary carcinoma of liver	Cancer
B150z00	primary malignant neoplasm of liver nos	Cancer
B151000	malignant neoplasm of interlobular bile ducts	Cancer
B151300	malignant neoplasm of intrahepatic canaliculi	Cancer
B151400	malignant neoplasm of intrahepatic gall duct	Cancer
B151z00	malignant neoplasm of intrahepatic bile ducts nos	Cancer
B152.00	malignant neoplasm of liver unspecified	Cancer
B15z.00	malignant neoplasm of liver and intrahepatic bile ducts nos	Cancer
B161100	malignant neoplasm of hepatic duct	Cancer
B161300	malignant neoplasm of sphincter of oddi	Cancer
B161z00	malignant neoplasm of extrahepatic bile ducts nos	Cancer
B171.00	malignant neoplasm of body of pancreas	Cancer
B172.00	malignant neoplasm of tail of pancreas	Cancer
B173.00	malignant neoplasm of pancreatic duct	Cancer
B174.00	malignant neoplasm of islets of langerhans	Cancer
B17y.00	malignant neoplasm of other specified sites of pancreas	Cancer
B17y000	malignant neoplasm of ectopic pancreatic tissue	Cancer
B17yz00	malignant neoplasm of specified site of pancreas nos	Cancer
B17z.00	malignant neoplasm of pancreas nos	Cancer
B18..00	malignant neoplasm of retroperitoneum and peritoneum	Cancer
B180100	malignant neoplasm of perinephric tissue	Cancer
B180200	malignant neoplasm of retrocaecal tissue	Cancer
B180z00	malignant neoplasm of retroperitoneum nos	Cancer
B18y.00	malignant neoplasm of specified parts of peritoneum	Cancer
B18y000	malignant neoplasm of mesocolon	Cancer

B18y100	malignant neoplasm of mesocaecum	Cancer
B18y200	malignant neoplasm of mesorectum	Cancer
B18y600	malignant neoplasm of the pouch of douglas	Cancer
B18yz00	malignant neoplasm of specified parts of peritoneum nos	Cancer
B18z.00	malignant neoplasm of retroperitoneum and peritoneum nos	Cancer
B1z0.00	malignant neoplasm of intestinal tract, part unspecified	Cancer
B1z1.00	malignant neoplasm of spleen nec	Cancer
B1z1000	angiosarcoma of spleen	Cancer
B1z1100	fibrosarcoma of spleen	Cancer
B1z1z00	malignant neoplasm of spleen nos	Cancer
B1z2.00	malignant neoplasm, overlapping lesion of digestive system	Cancer
B1zz.00	malignant neoplasm of digestive tract and peritoneum nos	Cancer
B200000	malignant neoplasm of cartilage of nose	Cancer
B200100	malignant neoplasm of nasal conchae	Cancer
B200300	malignant neoplasm of vestibule of nose	Cancer
B200z00	malignant neoplasm of nasal cavities nos	Cancer
B201000	malignant neoplasm of auditory (eustachian) tube	Cancer
B201100	malignant neoplasm of tympanic cavity	Cancer
B201200	malignant neoplasm of tympanic antrum	Cancer
B206.00	malignant neoplasm, overlapping lesion of accessory sinuses	Cancer
B20z.00	malignant neoplasm of accessory sinus nos	Cancer
B210.00	malignant neoplasm of glottis	Cancer
B211.00	malignant neoplasm of supraglottis	Cancer
B213000	malignant neoplasm of arytenoid cartilage	Cancer
B213100	malignant neoplasm of cricoid cartilage	Cancer
B213200	malignant neoplasm of cuneiform cartilage	Cancer
B213300	malignant neoplasm of thyroid cartilage	Cancer
B213z00	malignant neoplasm of laryngeal cartilage nos	Cancer
B21y.00	malignant neoplasm of larynx, other specified site	Cancer
B21z.00	malignant neoplasm of larynx nos	Cancer
B22..00	malignant neoplasm of trachea, bronchus and lung	Cancer
B220000	malignant neoplasm of cartilage of trachea	Cancer
B220100	malignant neoplasm of mucosa of trachea	Cancer
B220z00	malignant neoplasm of trachea nos	Cancer
B221000	malignant neoplasm of carina of bronchus	Cancer
B221z00	malignant neoplasm of main bronchus nos	Cancer
B222000	malignant neoplasm of upper lobe bronchus	Cancer
B222100	malignant neoplasm of upper lobe of lung	Cancer
B222z00	malignant neoplasm of upper lobe, bronchus or lung nos	Cancer
B223.00	malignant neoplasm of middle lobe, bronchus or lung	Cancer
B223000	malignant neoplasm of middle lobe bronchus	Cancer
B223100	malignant neoplasm of middle lobe of lung	Cancer
B223z00	malignant neoplasm of middle lobe, bronchus or lung nos	Cancer
B224.00	malignant neoplasm of lower lobe, bronchus or lung	Cancer
B224000	malignant neoplasm of lower lobe bronchus	Cancer
B224100	malignant neoplasm of lower lobe of lung	Cancer

B224z00	malignant neoplasm of lower lobe, bronchus or lung nos	Cancer
B22y.00	malignant neoplasm of other sites of bronchus or lung	Cancer
B23y.00	malignant neoplasm of other specified pleura	Cancer
B23z.00	malignant neoplasm of pleura nos	Cancer
B24..00	malignant neoplasm of thymus, heart and mediastinum	Cancer
B241z00	malignant neoplasm of heart nos	Cancer
B24z.00	malignant neoplasm of heart, thymus and mediastinum nos	Cancer
B2zy.00	malignant neoplasm of other site of respiratory tract	Cancer
B2zz.00	malignant neoplasm of respiratory tract nos	Cancer
B300.00	malignant neoplasm of bones of skull and face	Cancer
B300200	malignant neoplasm of malar bone	Cancer
B300500	malignant neoplasm of orbital bone	Cancer
B300z00	malignant neoplasm of bones of skull and face nos	Cancer
B302000	malignant neoplasm of cervical vertebra	Cancer
B302100	malignant neoplasm of thoracic vertebra	Cancer
B302200	malignant neoplasm of lumbar vertebra	Cancer
B302z00	malignant neoplasm of vertebral column nos	Cancer
B303.00	malignant neoplasm of ribs, sternum and clavicle	Cancer
B303300	malignant neoplasm of costal cartilage	Cancer
B303500	malignant neoplasm of xiphoid process	Cancer
B303z00	malignant neoplasm of rib, sternum and clavicle nos	Cancer
B304.00	malignant neoplasm of scapula and long bones of upper arm	Cancer
B304200	malignant neoplasm of humerus	Cancer
B305000	malignant neoplasm of carpal bone - scaphoid	Cancer
B305100	malignant neoplasm of carpal bone - lunate	Cancer
B305200	malignant neoplasm of carpal bone - triquetrum	Cancer
B305300	malignant neoplasm of carpal bone - pisiform	Cancer
B305400	malignant neoplasm of carpal bone - trapezium	Cancer
B305500	malignant neoplasm of carpal bone - trapezoid	Cancer
B305600	malignant neoplasm of carpal bone - capitate	Cancer
B305700	malignant neoplasm of carpal bone - hamate	Cancer
B305800	malignant neoplasm of first metacarpal bone	Cancer
B305900	malignant neoplasm of second metacarpal bone	Cancer
B305A00	malignant neoplasm of third metacarpal bone	Cancer
B305C00	malignant neoplasm of fifth metacarpal bone	Cancer
B305D00	malignant neoplasm of phalanges of hand	Cancer
B305z00	malignant neoplasm of hand bones nos	Cancer
B306.00	malignant neoplasm of pelvic bones, sacrum and coccyx	Cancer
B306300	malignant neoplasm of sacral vertebra	Cancer
B306400	malignant neoplasm of coccygeal vertebra	Cancer
B306z00	malignant neoplasm of pelvis, sacrum or coccyx nos	Cancer
B307.00	malignant neoplasm of long bones of leg	Cancer
B307z00	malignant neoplasm of long bones of leg nos	Cancer
B308200	malignant neoplasm of calcaneum	Cancer
B308300	malignant neoplasm of medial cuneiform	Cancer
B308400	malignant neoplasm of intermediate cuneiform	Cancer

B308500	malignant neoplasm of lateral cuneiform	Cancer
B308700	malignant neoplasm of navicular	Cancer
B308800	malignant neoplasm of first metatarsal bone	Cancer
B308900	malignant neoplasm of second metatarsal bone	Cancer
B308A00	malignant neoplasm of third metatarsal bone	Cancer
B308B00	malignant neoplasm of fourth metatarsal bone	Cancer
B308C00	malignant neoplasm of fifth metatarsal bone	Cancer
B308D00	malignant neoplasm of phalanges of foot	Cancer
B308z00	malignant neoplasm of short bones of leg nos	Cancer
B30z.00	malignant neoplasm of bone and articular cartilage nos	Cancer
B31..00	malignant neoplasm of connective and other soft tissue	Cancer
B310300	malignant neoplasm of cartilage of ear	Cancer
B310400	malignant neoplasm of tarsus of eyelid	Cancer
B310500	malignant neoplasm soft tissues of cervical spine	Cancer
B311000	malignant neoplasm of connective and soft tissue of shoulder	Cancer
B311100	malignant neoplasm of connective and soft tissue, upper arm	Cancer
B311200	malignant neoplasm of connective and soft tissue of fore-arm	Cancer
B311300	malignant neoplasm of connective and soft tissue of hand	Cancer
B311400	malignant neoplasm of connective and soft tissue of finger	Cancer
B311500	malignant neoplasm of connective and soft tissue of thumb	Cancer
B312000	malignant neoplasm of connective and soft tissue of hip	Cancer
B312400	malignant neoplasm of connective and soft tissue of foot	Cancer
B312500	malignant neoplasm of connective and soft tissue of toe	Cancer
B313.00	malignant neoplasm of connective and soft tissue of thorax	Cancer
B313000	malignant neoplasm of connective and soft tissue of axilla	Cancer
B314.00	malignant neoplasm of connective and soft tissue of abdomen	Cancer
B315.00	malignant neoplasm of connective and soft tissue of pelvis	Cancer
B315000	malignant neoplasm of connective and soft tissue of buttock	Cancer
B315200	malignant neoplasm of connective and soft tissue of perineum	Cancer
B31z.00	malignant neoplasm of connective and soft tissue, site nos	Cancer
B31z000	kaposi's sarcoma of soft tissue	Cancer
B320.00	malignant melanoma of lip	Cancer
B322.00	malignant melanoma of ear and external auricular canal	Cancer
B322000	malignant melanoma of auricle (ear)	Cancer
B322100	malignant melanoma of external auditory meatus	Cancer
B322z00	malignant melanoma of ear and external auricular canal nos	Cancer
B323.00	malignant melanoma of other and unspecified parts of face	Cancer
B323100	malignant melanoma of chin	Cancer
B323300	malignant melanoma of forehead	Cancer
B323500	malignant melanoma of temple	Cancer
B323z00	malignant melanoma of face nos	Cancer
B324.00	malignant melanoma of scalp and neck	Cancer
B324000	malignant melanoma of scalp	Cancer
B324100	malignant melanoma of neck	Cancer
B324z00	malignant melanoma of scalp and neck nos	Cancer
B325.00	malignant melanoma of trunk (excluding scrotum)	Cancer

B325000	malignant melanoma of axilla	Cancer
B325100	malignant melanoma of breast	Cancer
B325200	malignant melanoma of buttock	Cancer
B325300	malignant melanoma of groin	Cancer
B325500	malignant melanoma of perineum	Cancer
B325600	malignant melanoma of umbilicus	Cancer
B325z00	malignant melanoma of trunk, excluding scrotum, nos	Cancer
B326000	malignant melanoma of shoulder	Cancer
B326100	malignant melanoma of upper arm	Cancer
B326200	malignant melanoma of fore-arm	Cancer
B326300	malignant melanoma of hand	Cancer
B326400	malignant melanoma of finger	Cancer
B326500	malignant melanoma of thumb	Cancer
B326z00	malignant melanoma of upper limb or shoulder nos	Cancer
B327.00	malignant melanoma of lower limb and hip	Cancer
B327000	malignant melanoma of hip	Cancer
B327100	malignant melanoma of thigh	Cancer
B327200	malignant melanoma of knee	Cancer
B327300	malignant melanoma of popliteal fossa area	Cancer
B327400	malignant melanoma of lower leg	Cancer
B327500	malignant melanoma of ankle	Cancer
B327600	malignant melanoma of heel	Cancer
B327700	malignant melanoma of foot	Cancer
B327800	malignant melanoma of toe	Cancer
B327900	malignant melanoma of great toe	Cancer
B327z00	malignant melanoma of lower limb or hip nos	Cancer
B32y.00	malignant melanoma of other specified skin site	Cancer
B32z.00	malignant melanoma of skin nos	Cancer
B33..14	malignant neoplasm of sebaceous gland	Cancer
B332000	malignant neoplasm of skin of auricle (ear)	Cancer
B332100	malignant neoplasm of skin of external auditory meatus	Cancer
B333000	malignant neoplasm of skin of cheek, external	Cancer
B333400	malignant neoplasm of skin of nose (external)	Cancer
B333500	malignant neoplasm of skin of temple	Cancer
B334.00	malignant neoplasm of scalp and skin of neck	Cancer
B334000	malignant neoplasm of scalp	Cancer
B334z00	malignant neoplasm of scalp or skin of neck nos	Cancer
B335.00	malignant neoplasm of skin of trunk, excluding scrotum	Cancer
B335000	malignant neoplasm of skin of axillary fold	Cancer
B335100	malignant neoplasm of skin of chest, excluding breast	Cancer
B335300	malignant neoplasm of skin of abdominal wall	Cancer
B335z00	malignant neoplasm of skin of trunk, excluding scrotum, nos	Cancer
B336.00	malignant neoplasm of skin of upper limb and shoulder	Cancer
B336100	malignant neoplasm of skin of upper arm	Cancer
B336200	malignant neoplasm of skin of fore-arm	Cancer
B336500	malignant neoplasm of skin of thumb	Cancer

B336z00	malignant neoplasm of skin of upper limb or shoulder nos	Cancer
B337.00	malignant neoplasm of skin of lower limb and hip	Cancer
B337300	malignant neoplasm of skin of popliteal fossa area	Cancer
B337400	malignant neoplasm of skin of lower leg	Cancer
B337600	malignant neoplasm of skin of heel	Cancer
B337900	malignant neoplasm of skin of great toe	Cancer
B337z00	malignant neoplasm of skin of lower limb or hip nos	Cancer
B33y.00	malignant neoplasm of other specified skin sites	Cancer
B33z.00	malignant neoplasm of skin nos	Cancer
B340.00	malignant neoplasm of nipple and areola of female breast	Cancer
B340z00	malignant neoplasm of nipple or areola of female breast nos	Cancer
B341.00	malignant neoplasm of central part of female breast	Cancer
B342.00	malignant neoplasm of upper-inner quadrant of female breast	Cancer
B343.00	malignant neoplasm of lower-inner quadrant of female breast	Cancer
B344.00	malignant neoplasm of upper-outer quadrant of female breast	Cancer
B345.00	malignant neoplasm of lower-outer quadrant of female breast	Cancer
B346.00	malignant neoplasm of axillary tail of female breast	Cancer
B347.00	malignant neoplasm, overlapping lesion of breast	Cancer
B34y.00	malignant neoplasm of other site of female breast	Cancer
B34y000	malignant neoplasm of ectopic site of female breast	Cancer
B34yz00	malignant neoplasm of other site of female breast nos	Cancer
B34z.00	malignant neoplasm of female breast nos	Cancer
B350.00	malignant neoplasm of nipple and areola of male breast	Cancer
B350z00	malignant neoplasm of nipple or areola of male breast nos	Cancer
B35z.00	malignant neoplasm of other site of male breast	Cancer
B35z000	malignant neoplasm of ectopic site of male breast	Cancer
B35zz00	malignant neoplasm of male breast nos	Cancer
B40..00	malignant neoplasm of uterus, part unspecified	Cancer
B410000	malignant neoplasm of endocervical canal	Cancer
B410100	malignant neoplasm of endocervical gland	Cancer
B410z00	malignant neoplasm of endocervix nos	Cancer
B412.00	malignant neoplasm, overlapping lesion of cervix uteri	Cancer
B41y.00	malignant neoplasm of other site of cervix	Cancer
B41y000	malignant neoplasm of cervical stump	Cancer
B41y100	malignant neoplasm of squamocolumnar junction of cervix	Cancer
B41yz00	malignant neoplasm of other site of cervix nos	Cancer
B41z.00	malignant neoplasm of cervix uteri nos	Cancer
B911013	choriocarcinoma	Cancer
B430.00	malignant neoplasm of corpus uteri, excluding isthmus	Cancer
B430000	malignant neoplasm of cornu of corpus uteri	Cancer
B430100	malignant neoplasm of fundus of corpus uteri	Cancer
B430200	malignant neoplasm of endometrium of corpus uteri	Cancer
B430300	malignant neoplasm of myometrium of corpus uteri	Cancer
B430z00	malignant neoplasm of corpus uteri nos	Cancer
B431.00	malignant neoplasm of isthmus of uterine body	Cancer
B431000	malignant neoplasm of lower uterine segment	Cancer

B431z00	malignant neoplasm of isthmus of uterine body nos	Cancer
B432.00	malignant neoplasm of overlapping lesion of corpus uteri	Cancer
B43y.00	malignant neoplasm of other site of uterine body	Cancer
B43z.00	malignant neoplasm of body of uterus nos	Cancer
B44..00	malignant neoplasm of ovary and other uterine adnexa	Cancer
B444.00	malignant neoplasm of round ligament	Cancer
B44y.00	malignant neoplasm of other site of uterine adnexa	Cancer
B44z.00	malignant neoplasm of uterine adnexa nos	Cancer
B450000	malignant neoplasm of gartner's duct	Cancer
B450100	malignant neoplasm of vaginal vault	Cancer
B450z00	malignant neoplasm of vagina nos	Cancer
B451000	malignant neoplasm of greater vestibular (bartholin's) gland	Cancer
B451z00	malignant neoplasm of labia majora nos	Cancer
B45y.00	malignant neoplasm of other specified female genital organ	Cancer
B45z.00	malignant neoplasm of female genital organ nos	Cancer
B470.00	malignant neoplasm of undescended testis	Cancer
B470000	malignant neoplasm of ectopic testis	Cancer
B470z00	malignant neoplasm of undescended testis nos	Cancer
B48..00	malignant neoplasm of penis and other male genital organs	Cancer
B482.00	malignant neoplasm of body of penis	Cancer
B483.00	malignant neoplasm of penis, part unspecified	Cancer
B48y.00	malignant neoplasm of other male genital organ	Cancer
B48y000	malignant neoplasm of seminal vesicle	Cancer
B48y100	malignant neoplasm of tunica vaginalis	Cancer
B48yz00	malignant neoplasm of other male genital organ nos	Cancer
B48z.00	malignant neoplasm of penis and other male genital organ nos	Cancer
B490.00	malignant neoplasm of trigone of urinary bladder	Cancer
B491.00	malignant neoplasm of dome of urinary bladder	Cancer
B492.00	malignant neoplasm of lateral wall of urinary bladder	Cancer
B493.00	malignant neoplasm of anterior wall of urinary bladder	Cancer
B494.00	malignant neoplasm of posterior wall of urinary bladder	Cancer
B495.00	malignant neoplasm of bladder neck	Cancer
B496.00	malignant neoplasm of ureteric orifice	Cancer
B49y.00	malignant neoplasm of other site of urinary bladder	Cancer
B49y000	malignant neoplasm, overlapping lesion of bladder	Cancer
B49z.00	malignant neoplasm of urinary bladder nos	Cancer
B4A0.00	malignant neoplasm of kidney parenchyma	Cancer
B4A0000	hypernephroma	Cancer
B4A1100	malignant neoplasm of ureteropelvic junction	Cancer
B4A1z00	malignant neoplasm of renal pelvis nos	Cancer
B4Ay.00	malignant neoplasm of other urinary organs	Cancer
B4Ay000	malignant neoplasm of overlapping lesion of urinary organs	Cancer
B4Az.00	malignant neoplasm of kidney or urinary organs nos	Cancer
B4z..00	malignant neoplasm of genitourinary organ nos	Cancer
B500000	malignant neoplasm of ciliary body	Cancer
B500100	malignant neoplasm of iris	Cancer

B500200	malignant neoplasm of crystalline lens	Cancer
B500300	malignant neoplasm of sclera	Cancer
B500z00	malignant neoplasm of eyeball nos	Cancer
B501000	malignant neoplasm of connective tissue of orbit	Cancer
B501100	malignant neoplasm of extraocular muscle of orbit	Cancer
B501z00	malignant neoplasm of orbit nos	Cancer
B502.00	malignant neoplasm of lacrimal gland	Cancer
B507000	malignant neoplasm of lacrimal sac	Cancer
B507100	malignant neoplasm of nasolacrimal duct	Cancer
B507z00	malignant neoplasm of lacrimal duct nos	Cancer
B50y.00	malignant neoplasm of other specified site of eye	Cancer
B50z.00	malignant neoplasm of eye nos	Cancer
B510000	malignant neoplasm of basal ganglia	Cancer
B510100	malignant neoplasm of cerebral cortex	Cancer
B510200	malignant neoplasm of corpus striatum	Cancer
B510300	malignant neoplasm of globus pallidus	Cancer
B510400	malignant neoplasm of hypothalamus	Cancer
B510500	malignant neoplasm of thalamus	Cancer
B510z00	malignant neoplasm of cerebrum nos	Cancer
B512000	malignant neoplasm of hippocampus	Cancer
B512100	malignant neoplasm of uncus	Cancer
B512z00	malignant neoplasm of temporal lobe nos	Cancer
B515000	malignant neoplasm of choroid plexus	Cancer
B515100	malignant neoplasm of floor of cerebral ventricle	Cancer
B515z00	malignant neoplasm of cerebral ventricle nos	Cancer
B517000	malignant neoplasm of cerebral peduncle	Cancer
B517100	malignant neoplasm of medulla oblongata	Cancer
B517200	malignant neoplasm of midbrain	Cancer
B517300	malignant neoplasm of pons	Cancer
B51y.00	malignant neoplasm of other parts of brain	Cancer
B51y000	malignant neoplasm of corpus callosum	Cancer
B51y100	malignant neoplasm of tapetum	Cancer
B51yz00	malignant neoplasm of other part of brain nos	Cancer
B51z.00	malignant neoplasm of brain nos	Cancer
B520000	malignant neoplasm of olfactory bulb	Cancer
B520z00	malignant neoplasm of cranial nerves nos	Cancer
B521000	malignant neoplasm of cerebral dura mater	Cancer
B521100	malignant neoplasm of cerebral arachnoid mater	Cancer
B521200	malignant neoplasm of cerebral pia mater	Cancer
B521z00	malignant neoplasm of cerebral meninges nos	Cancer
B523000	malignant neoplasm of spinal dura mater	Cancer
B523100	malignant neoplasm of spinal arachnoid mater	Cancer
B523200	malignant neoplasm of spinal pia mater	Cancer
B523z00	malignant neoplasm of spinal meninges nos	Cancer
B524300	malignant neoplasm of peripheral nerve of thorax	Cancer
B524400	malignant neoplasm of peripheral nerve of abdomen	Cancer

B524500	malignant neoplasm of peripheral nerve of pelvis	Cancer
B52y.00	malignant neoplasm of other specified part of nervous system	Cancer
B52z.00	malignant neoplasm of nervous system nos	Cancer
B540z00	malignant neoplasm of adrenal gland nos	Cancer
B542100	malignant neoplasm of craniopharyngeal duct	Cancer
B545.00	malignant neoplasm of aortic body and other paraganglia	Cancer
B545z00	malignant neoplasm of aortic body or paraganglia nos	Cancer
B54y.00	malignant neoplasm of other specified endocrine gland	Cancer
B55..00	malignant neoplasm of other and ill-defined sites	Cancer
B550.00	malignant neoplasm of head, neck and face	Cancer
B550000	malignant neoplasm of head nos	Cancer
B550100	malignant neoplasm of cheek nos	Cancer
B550200	malignant neoplasm of nose nos	Cancer
B550300	malignant neoplasm of jaw nos	Cancer
B550400	malignant neoplasm of neck nos	Cancer
B550500	malignant neoplasm of supraclavicular fossa nos	Cancer
B550z00	malignant neoplasm of head, neck and face nos	Cancer
B551.00	malignant neoplasm of thorax	Cancer
B551000	malignant neoplasm of axilla nos	Cancer
B551100	malignant neoplasm of chest wall nos	Cancer
B551200	malignant neoplasm of intrathoracic site nos	Cancer
B551z00	malignant neoplasm of thorax nos	Cancer
B552.00	malignant neoplasm of abdomen	Cancer
B553000	malignant neoplasm of inguinal region nos	Cancer
B553z00	malignant neoplasm of pelvis nos	Cancer
B554.00	malignant neoplasm of upper limb nos	Cancer
B555.00	malignant neoplasm of lower limb nos	Cancer
B55y.00	malignant neoplasm of other specified sites	Cancer
B55y000	malignant neoplasm of back nos	Cancer
B55y100	malignant neoplasm of trunk nos	Cancer
B55y200	malignant neoplasm of flank nos	Cancer
B55yz00	malignant neoplasm of specified site nos	Cancer
B573.00	secondary malignant neoplasm of other respiratory organs	Cancer
B57y.00	secondary malignant neoplasm of other digestive organ	Cancer
B581.00	secondary malignant neoplasm of other urinary organs	Cancer
B581z00	secondary malignant neoplasm of other urinary organ nos	Cancer
B582000	secondary malignant neoplasm of skin of head	Cancer
B582400	secondary malignant neoplasm of skin of shoulder and arm	Cancer
B582500	secondary malignant neoplasm of skin of hip and leg	Cancer
B582z00	secondary malignant neoplasm of skin nos	Cancer
B583.00	secondary malignant neoplasm of brain and spinal cord	Cancer
B583z00	secondary malignant neoplasm of brain or spinal cord nos	Cancer
B584.00	secondary malignant neoplasm of other part of nervous system	Cancer
B58y200	secondary malignant neoplasm of cervix uteri	Cancer
B58y211	secondary cancer of the cervix	Cancer
B58y800	secondary malignant neoplasm of epididymis and vas deferens	Cancer

B58z.00	secondary malignant neoplasm of other specified site nos	Cancer
B59..00	malignant neoplasm of unspecified site	Cancer
B591.00	other malignant neoplasm nos	Cancer
B592.00	malignant neoplasms of independent (primary) multiple sites	Cancer
B59z.00	malignant neoplasm of unspecified site nos	Cancer
B5z..00	malignant neoplasm of other and unspecified site nos	Cancer
B60..00	lymphosarcoma and reticulosarcoma	Cancer
B600000	reticulosarcoma of unspecified site	Cancer
B600100	reticulosarcoma of lymph nodes of head, face and neck	Cancer
B600400	reticulosarcoma of lymph nodes of axilla and upper limb	Cancer
B600z00	reticulosarcoma nos	Cancer
B601.00	lymphosarcoma	Cancer
B601000	lymphosarcoma of unspecified site	Cancer
B601100	lymphosarcoma of lymph nodes of head, face and neck	Cancer
B601200	lymphosarcoma of intrathoracic lymph nodes	Cancer
B601300	lymphosarcoma of intra-abdominal lymph nodes	Cancer
B601400	lymphosarcoma of lymph nodes of axilla and upper limb	Cancer
B601600	lymphosarcoma of intrapelvic lymph nodes	Cancer
B601700	lymphosarcoma of spleen	Cancer
B601800	lymphosarcoma of lymph nodes of multiple sites	Cancer
B601z00	lymphosarcoma nos	Cancer
B602000	burkitt's lymphoma of unspecified site	Cancer
B602100	burkitt's lymphoma of lymph nodes of head, face and neck	Cancer
B602200	burkitt's lymphoma of intrathoracic lymph nodes	Cancer
B602300	burkitt's lymphoma of intra-abdominal lymph nodes	Cancer
B602400	burkitt's lymphoma of lymph nodes of axilla and upper limb	Cancer
B602600	burkitt's lymphoma of intrapelvic lymph nodes	Cancer
B602700	burkitt's lymphoma of spleen	Cancer
B602800	burkitt's lymphoma of lymph nodes of multiple sites	Cancer
B602z00	burkitt's lymphoma nos	Cancer
B60y.00	other specified reticulosarcoma or lymphosarcoma	Cancer
B60z.00	reticulosarcoma or lymphosarcoma nos	Cancer
B610000	hodgkin's paragranuloma of unspecified site	Cancer
B610200	hodgkin's paragranuloma of intrathoracic lymph nodes	Cancer
B610300	hodgkin's paragranuloma of intra-abdominal lymph nodes	Cancer
B610600	hodgkin's paragranuloma of intrapelvic lymph nodes	Cancer
B610800	hodgkin's paragranuloma of lymph nodes of multiple sites	Cancer
B610z00	hodgkin's paragranuloma nos	Cancer
B611000	hodgkin's granuloma of unspecified site	Cancer
B611100	hodgkin's granuloma of lymph nodes of head, face and neck	Cancer
B611300	hodgkin's granuloma of intra-abdominal lymph nodes	Cancer
B611400	hodgkin's granuloma of lymph nodes of axilla and upper limb	Cancer
B611800	hodgkin's granuloma of lymph nodes of multiple sites	Cancer
B611z00	hodgkin's granuloma nos	Cancer
B612000	hodgkin's sarcoma of unspecified site	Cancer
B612100	hodgkin's sarcoma of lymph nodes of head, face and neck	Cancer

B612400	hodgkin's sarcoma of lymph nodes of axilla and upper limb	Cancer
B612800	hodgkin's sarcoma of lymph nodes of multiple sites	Cancer
B612z00	hodgkin's sarcoma nos	Cancer
B613700	hodgkin's, lymphocytic-histiocytic predominance of spleen	Cancer
B613z00	hodgkin's, lymphocytic-histiocytic predominance nos	Cancer
B614000	hodgkin's disease, nodular sclerosis of unspecified site	Cancer
B614z00	hodgkin's disease, nodular sclerosis nos	Cancer
B615000	hodgkin's disease, mixed cellularity of unspecified site	Cancer
B615z00	hodgkin's disease, mixed cellularity nos	Cancer
B616000	hodgkin's lymphocytic depletion of unspecified site	Cancer
B616700	hodgkin's disease, lymphocytic depletion of spleen	Cancer
B616z00	hodgkin's disease, lymphocytic depletion nos	Cancer
B61zz00	hodgkin's disease nos	Cancer
B61z000	hodgkin's disease nos, unspecified site	Cancer
B61z100	hodgkin's disease nos of lymph nodes of head, face and neck	Cancer
B61z200	hodgkin's disease nos of intrathoracic lymph nodes	Cancer
B61z300	hodgkin's disease nos of intra-abdominal lymph nodes	Cancer
B61z600	hodgkin's disease nos of intrapelvic lymph nodes	Cancer
B61z700	hodgkin's disease nos of spleen	Cancer
B61z800	hodgkin's disease nos of lymph nodes of multiple sites	Cancer
B62..00	other malignant neoplasm of lymphoid and histiocytic tissue	Cancer
B620000	nodular lymphoma of unspecified site	Cancer
B620100	nodular lymphoma of lymph nodes of head, face and neck	Cancer
B620400	nodular lymphoma of lymph nodes of axilla and upper limb	Cancer
B620z00	nodular lymphoma nos	Cancer
B621000	mycosis fungoides of unspecified site	Cancer
B621800	mycosis fungoides of lymph nodes of multiple sites	Cancer
B621z00	mycosis fungoides nos	Cancer
B622000	sezary's disease of unspecified site	Cancer
B622100	sezary's disease of lymph nodes of head, face and neck	Cancer
B622200	sezary's disease of intrathoracic lymph nodes	Cancer
B622300	sezary's disease of intra-abdominal lymph nodes	Cancer
B622400	sezary's disease of lymph nodes of axilla and upper limb	Cancer
B622600	sezary's disease of intrapelvic lymph nodes	Cancer
B622800	sezary's disease of lymph nodes of multiple sites	Cancer
B622z00	sezary's disease nos	Cancer
B623000	malignant histiocytosis of unspecified site	Cancer
B623z00	malignant histiocytosis nos	Cancer
B624000	leukaemic reticuloendotheliosis of unspecified sites	Cancer
B624700	leukaemic reticuloendotheliosis of spleen	Cancer
B624z00	leukaemic reticuloendotheliosis nos	Cancer
B625000	letterer-siwe disease of unspecified sites	Cancer
B625z00	letterer-siwe disease nos	Cancer
B626.00	malignant mast cell tumours	Cancer
B626000	mast cell malignancy of unspecified site	Cancer
B626100	mast cell malignancy of lymph nodes of head, face and neck	Cancer

B626200	mast cell malignancy of intrathoracic lymph nodes	Cancer
B626300	mast cell malignancy of intra-abdominal lymph nodes	Cancer
B626400	mast cell malignancy of lymph nodes of axilla and upper limb	Cancer
B626600	mast cell malignancy of intrapelvic lymph nodes	Cancer
B626700	mast cell malignancy of spleen	Cancer
B626800	mast cell malignancy of lymph nodes of multiple sites	Cancer
B626z00	malignant mast cell tumour nos	Cancer
B627300	diffuse non-hodgkin's small cell (diffuse) lymphoma	Cancer
B627400	diffuse non-hodgkin's small cleaved cell (diffuse) lymphoma	Cancer
B627800	diffuse non-hodgkin's lymphoma undifferentiated (diffuse)	Cancer
B627B00	other types of follicular non-hodgkin's lymphoma	Cancer
B62x.00	malignant lymphoma otherwise specified	Cancer
B62x500	malignant immunoproliferative small intestinal disease	Cancer
B62yz00	malignant lymphoma nos	Cancer
B62y000	malignant lymphoma nos of unspecified site	Cancer
B62y100	malignant lymphoma nos of lymph nodes of head, face and neck	Cancer
B62y200	malignant lymphoma nos of intrathoracic lymph nodes	Cancer
B62y300	malignant lymphoma nos of intra-abdominal lymph nodes	Cancer
B62y600	malignant lymphoma nos of intrapelvic lymph nodes	Cancer
B62y700	malignant lymphoma nos of spleen	Cancer
B62y800	malignant lymphoma nos of lymph nodes of multiple sites	Cancer
B62z.00	malignant neoplasms of lymphoid and histiocytic tissue nos	Cancer
B630000	malignant plasma cell neoplasm, extramedullary plasmacytoma	Cancer
B63y.00	other immunoproliferative neoplasms	Cancer
B63z.00	immunoproliferative neoplasm or myeloma nos	Cancer
B64..11	lymphatic leukaemia	Cancer
B64..00	lymphoid leukaemia	Cancer
B642.00	subacute lymphoid leukaemia	Cancer
B64y.00	other lymphoid leukaemia	Cancer
B64y000	aleukaemic lymphoid leukaemia	Cancer
B64y200	adult t-cell leukaemia	Cancer
B64yz00	other lymphoid leukaemia nos	Cancer
B64z.00	lymphoid leukaemia nos	Cancer
B65..00	myeloid leukaemia	Cancer
B651000	chronic eosinophilic leukaemia	Cancer
B651200	chronic neutrophilic leukaemia	Cancer
B651z00	chronic myeloid leukaemia nos	Cancer
B652.00	subacute myeloid leukaemia	Cancer
B653000	chloroma	Cancer
B653100	granulocytic sarcoma	Cancer
B653z00	myeloid sarcoma nos	Cancer
B65y.00	other myeloid leukaemia	Cancer
B65y000	aleukaemic myeloid leukaemia	Cancer
B65yz00	other myeloid leukaemia nos	Cancer
B65z.00	myeloid leukaemia nos	Cancer
B66..00	monocytic leukaemia	Cancer

B66..12	monoblastic leukaemia	Cancer
B66..11	histiocytic leukaemia	Cancer
B661.00	chronic monocytic leukaemia	Cancer
B662.00	subacute monocytic leukaemia	Cancer
B66y.00	other monocytic leukaemia	Cancer
B66y000	aleukaemic monocytic leukaemia	Cancer
B66yz00	other monocytic leukaemia nos	Cancer
B66z.00	monocytic leukaemia nos	Cancer
B67..00	other specified leukaemia	Cancer
B672.11	thrombocytic leukaemia	Cancer
B672.00	megakaryocytic leukaemia	Cancer
B67y.00	other and unspecified leukaemia	Cancer
B67yz00	other and unspecified leukaemia nos	Cancer
B67z.00	other specified leukaemia nos	Cancer
B68..00	leukaemia of unspecified cell type	Cancer
B680.00	acute leukaemia nos	Cancer
B681.00	chronic leukaemia nos	Cancer
B682.00	subacute leukaemia nos	Cancer
B68y.00	other leukaemia of unspecified cell type	Cancer
B68z.00	leukaemia nos	Cancer
B69..00	myelomonocytic leukaemia	Cancer
B692.00	subacute myelomonocytic leukaemia	Cancer
B6z..00	malignant neoplasm lymphatic or haematopoietic tissue nos	Cancer
B628500	diffuse follicle centre lymphoma	Cancer
B800z00	carcinoma in situ of lip, oral cavity and pharynx nos	Cancer
B801z00	carcinoma in situ of oesophagus nos	Cancer
B802z00	carcinoma in situ of stomach nos	Cancer
B803700	carcinoma in situ of splenic flexure of colon	Cancer
B803z00	carcinoma in situ of colon nos	Cancer
B804.00	carcinoma in situ of rectum and rectosigmoid junction	Cancer
B804z00	carcinoma in situ of rectum or rectosigmoid junction nos	Cancer
B806.00	carcinoma in situ of anus nos	Cancer
B807.00	carcinoma in situ of other and unspecified small intestine	Cancer
B808200	carcinoma in situ of hepatic duct	Cancer
B808z00	carcinoma in situ of liver or biliary system nos	Cancer
B80z.00	carcinoma in situ of other and unspecified digestive organs	Cancer
B81..00	carcinoma in situ of respiratory system	Cancer
B810000	carcinoma in situ of thyroid cartilage	Cancer
B810100	carcinoma in situ of cricoid cartilage	Cancer
B810300	carcinoma in situ of arytenoid cartilage	Cancer
B810600	carcinoma in situ of aryepiglottic fold	Cancer
B810700	carcinoma in situ of vestibular fold	Cancer
B810z00	carcinoma in situ of larynx nos	Cancer
B812.00	carcinoma in situ of bronchus and lung	Cancer
B812000	carcinoma in situ of carina of bronchus	Cancer
B812200	carcinoma in situ of upper lobe bronchus and lung	Cancer

B812300	carcinoma in situ of middle lobe bronchus and lung	Cancer
B812400	carcinoma in situ of lower lobe bronchus and lung	Cancer
B812z00	carcinoma in situ of bronchus or lung nos	Cancer
B81z.00	carcinoma in situ of respiratory organ nos	Cancer
B821.00	carcinoma in situ of skin of eyelid including canthus	Cancer
B822000	carcinoma in situ of skin of auricle	Cancer
B823500	carcinoma in situ of skin of temple	Cancer
B823600	carcinoma in situ of skin of jaw	Cancer
B824.00	carcinoma in situ of scalp and skin of neck	Cancer
B824000	carcinoma in situ of scalp	Cancer
B825.00	carcinoma in situ of skin of trunk, excluding scrotum	Cancer
B825100	carcinoma in situ of skin of chest wall nos	Cancer
B825400	carcinoma in situ of skin of abdominal wall	Cancer
B825z00	carcinoma in situ of skin of trunk nos	Cancer
B826.00	carcinoma in situ of skin of upper limb and shoulder	Cancer
B826100	carcinoma in situ of skin of upper arm	Cancer
B826200	carcinoma in situ of skin of lower arm	Cancer
B826z00	carcinoma in situ of skin of upper limb or shoulder nos	Cancer
B827.00	carcinoma in situ of skin of lower limb and hip	Cancer
B827300	carcinoma in situ of skin of lower leg	Cancer
B827z00	carcinoma in situ of skin of lower limb or hip nos	Cancer
B828300	melanoma in situ of scalp and neck	Cancer
B82y.00	carcinoma in situ of other specified sites of skin	Cancer
B82z.00	carcinoma in situ of skin nos	Cancer
B83..00	carcinoma in situ of breast and genitourinary system	Cancer
B830.00	carcinoma in situ of breast	Cancer
B833z00	carcinoma in situ of female genital organs nos	Cancer
B83z.00	carcinoma in situ of urinary organs nos	Cancer
B8y..00	carcinoma in situ of other and unspecified sites	Cancer
B8yy.00	carcinoma in situ of other specified site	Cancer
B8yyz00	carcinoma in situ of other specified site nos	Cancer
B8z..00	carcinoma in situ nos	Cancer
BB06.00	[m]tumour cells, uncertain whether benign or malignant	Cancer
BB12.00	[m]carcinoma nos	Cancer
BB13.00	[m]carcinoma, metastatic, nos	Cancer
BB17.00	[m]large cell carcinoma nos	Cancer
BB18.00	[m]carcinoma, undifferentiated type, nos	Cancer
BB19.00	[m]carcinoma, anaplastic type, nos	Cancer
BB1G.00	[m]spheroidal cell carcinoma	Cancer
BB1J.00	[m]small cell carcinoma nos	Cancer
BB22.00	[m]papillary carcinoma nos	Cancer
BB24.00	[m]verrucous carcinoma nos	Cancer
BB2A.00	[m]squamous cell carcinoma nos	Cancer
BB2B.00	[m]squamous cell carcinoma, metastatic nos	Cancer
BB31.00	[m]basal cell carcinoma nos	Cancer
BB43.00	[m]transitional cell carcinoma nos	Cancer

BB4z.00	[m]transitional cell papilloma or carcinoma nos	Cancer
BB52.00	[m]adenocarcinoma nos	Cancer
BB53.00	[m]adenocarcinoma, metastatic, nos	Cancer
BB5B.00	[m]pancreatic adenomas and carcinomas	Cancer
BB5Bz00	[m]pancreatic adenoma or carcinoma nos	Cancer
BB5C.00	[m]gastrinoma and carcinomas	Cancer
BB5Cz00	[m]gastrinoma or carcinoma nos	Cancer
BB5D.00	[m]hepatobiliary tract adenomas and carcinomas	Cancer
BB5Dz00	[m]hepatobiliary adenoma or carcinoma nos	Cancer
BB5L.00	[m]adenomatous and adenocarcinomatous polyps	Cancer
BB5Lz00	[m]adenomatous or adenocarcinomatous polyp nos	Cancer
BB5M.00	[m]tubular adenomas and adenocarcinomas	Cancer
BB5Mz00	[m]tubular adenoma or adenocarcinoma nos	Cancer
BB5Nz00	[m]adenomatous or adenocarcinomatous polyps of the colon nos	Cancer
BB5P.00	[m]solid carcinoma nos	Cancer
BB5R100	[m]carcinoid tumour, malignant	Cancer
BB5R500	[m]carcinoid tumour, nonargentaffin, malignant	Cancer
BB5S.00	[m]respiratory tract adenomas and adenocarcinomas	Cancer
BB5Sz00	[m]respiratory tract adenoma or adenocarcinoma nos	Cancer
BB5T.00	[m]papillary adenomas and adenocarcinomas	Cancer
BB5T100	[m]papillary adenocarcinoma nos	Cancer
BB5Tz00	[m]papillary adenoma or adenocarcinoma nos	Cancer
BB5U.00	[m]villous adenomas and adenocarcinomas	Cancer
BB5U100	[m]adenocarcinoma in villous adenoma	Cancer
BB5Uz00	[m]villous adenoma or adenocarcinoma nos	Cancer
BB5V.00	[m]pituitary adenomas and carcinomas	Cancer
BB5Vz00	[m]pituitary adenoma or carcinoma nos	Cancer
BB5W.00	[m]oxyphilic adenomas and adenocarcinomas	Cancer
BB5Wz00	[m]oxyphilic adenoma or adenocarcinoma nos	Cancer
BB5X.00	[m]clear cell adenomas and adenocarcinomas	Cancer
BB5X100	[m]clear cell adenocarcinoma nos	Cancer
BB5Xz00	[m]clear cell adenoma or adenocarcinoma nos	Cancer
BB5a.00	[m]renal adenoma and carcinoma	Cancer
BB5az00	[m]renal adenoma or carcinoma nos	Cancer
BB5c.00	[m]parathyroid adenomas and adenocarcinomas	Cancer
BB5cz00	[m]parathyroid adenoma or adenocarcinoma nos	Cancer
BB5f.00	[m]thyroid adenoma and adenocarcinoma	Cancer
BB5f100	[m]follicular adenocarcinoma nos	Cancer
BB5fz00	[m]thyroid adenoma or adenocarcinoma nos	Cancer
BB5j.00	[m]endometrioid adenomas and carcinomas	Cancer
BB5jz00	[m]endometrioid adenoma or carcinoma nos	Cancer
BB5z.00	[m]adenoma or adenocarcinoma nos	Cancer
BB60.00	[m]skin appendage adenoma and carcinoma	Cancer
BB60100	[m]skin appendage carcinoma	Cancer
BB61.00	[m]sweat gland adenoma and adenocarcinomas	Cancer
BB61200	[m]sweat gland adenocarcinoma	Cancer

BB62.00	[m]apocrine adenoma and adenocarcinomas	Cancer
BB62z00	[m]apocrine adenoma or adenocarcinoma nos	Cancer
BB69.00	[m]sebaceous adenoma and adenocarcinoma	Cancer
BB69z00	[m]sebaceous adenoma or adenocarcinoma nos	Cancer
BB6A.00	[m]ceruminous adenoma and adenocarcinoma	Cancer
BB80.00	[m]cystadenoma and carcinoma	Cancer
BB80100	[m]cystadenocarcinoma nos	Cancer
BB80z00	[m]cystadenoma or carcinoma nos	Cancer
BB81200	[m]serous cystadenocarcinoma, nos	Cancer
BB81500	[m]papillary cystadenocarcinoma, nos	Cancer
BB81E00	[m]mucinous cystadenocarcinoma nos	Cancer
BB82.00	[m]mucinous adenoma and adenocarcinoma	Cancer
BB82z00	[m]mucinous adenoma or adenocarcinoma nos	Cancer
BB85z00	[m]signet ring carcinoma nos	Cancer
BB93.00	[m]comedocarcinoma nos	Cancer
BB9B.00	[m]medullary carcinoma nos	Cancer
BB9E000	[m]intraductal carcinoma and lobular carcinoma in situ	Cancer
BB9F.00	[m]lobular carcinoma nos	Cancer
BBE..00	[m]naevi and melanomas	Cancer
BBEQ.00	[m]spindle cell melanoma nos	Cancer
BBEz.00	[m]naevi or melanoma nos	Cancer
BBF..00	[m]soft tissue tumours and sarcomas nos	Cancer
BBF1.00	[m]sarcoma nos	Cancer
BBF2.00	[m]sarcomatosis nos	Cancer
BBFz.00	[m]soft tissue tumour or sarcoma nos	Cancer
BBJ1.00	[m]liposarcoma nos	Cancer
BBK0200	[m]leiomyosarcoma nos	Cancer
BBK2.00	[m]myoma and myosarcoma	Cancer
BBK2z00	[m]myoma or myosarcoma nos	Cancer
BBL4.00	[m]mixed tumour, malignant, nos	Cancer
BBL9.00	[m]carcinosarcoma nos	Cancer
BBM8.00	[m]cystosarcoma phyllodes nos	Cancer
BBN5.00	[m]clear cell sarcoma of tendons and aponeuroses	Cancer
BBQ3.00	[m]embryonal carcinoma nos	Cancer
BBVA.00	[m] small cell osteosarcoma	Cancer
BBVz.00	[m]osteoma or osteosarcoma nos	Cancer
BBW4.00	[m]chondrosarcoma nos	Cancer
BBg..00	[m]lymphomas, nos or diffuse	Cancer
BBg1000	[m]malignant lymphoma, diffuse nos	Cancer
BBg3.00	[m]malignant lymphoma, undifferentiated cell type nos	Cancer
BBg5.00	[m]malignant lymphoma, convoluted cell type nos	Cancer
BBgA.00	[m]malignant lymphoma, centroblastic-centrocytic, diffuse	Cancer
BBgB.00	[m]malignant lymphoma, follicular centre cell nos	Cancer
BBgC.00	[m]malignant lymphoma, lymphocytic, well differentiated nos	Cancer
BBgE.00	[m]malignant lymphoma, centrocytic	Cancer
BBgH.00	[m]prolymphocytic lymphosarcoma	Cancer

BBgJ.00	[m]malignant lymphoma, centroblastic type nos	Cancer
BBgQ.00	[m]malignant lymphomatous polyposis	Cancer
BBgS.00	[m]malignant lymphoma, large cell, cleaved, diffuse	Cancer
BBj1.00	[m]hodgkin's disease, lymphocytic predominance	Cancer
BBj1000	[m]hodgkin,s disease, lymphocytic predominance, diffuse	Cancer
BBj6100	[m]hodgkin,s disease, nodular sclerosis, mixed cellularity	Cancer
BBk2.00	[m]malignant lymphoma, centroblastic-centrocytic, follicular	Cancer
BBkz.00	[m]lymphoma, nodular or follicular nos	Cancer
BBlz.00	[m]mycosis fungoides nos	Cancer
BBm2.00	[m]histiocytic medullary reticulosis	Cancer
BBm5.00	[m] peripheral t-cell lymphoma nos	Cancer
BBmH.00	[m] large cell lymphoma	Cancer
BBp0.00	[m]mastocytoma nos	Cancer
BBv0.00	[m]monocytoid b-cell lymphoma	Cancer
By...00	neoplasms otherwise specified	Cancer
Byu..00	[x]additional neoplasm classification terms	Cancer
Byu1.00	[x]malignant neoplasm of digestive organs	Cancer
Byu1000	[x]other sarcomas of the liver	Cancer
Byu1100	[x]other specified carcinomas of liver	Cancer
Byu1200	[x]malignant neoplasm of intestinal tract, part unspecified	Cancer
Byu2000	[x]malignant neoplasm of bronchus or lung, unspecified	Cancer
Byu2500	[x]malignant neoplasm of mediastinum, part unspecified	Cancer
Byu3.00	[x]malignant neoplasm of bone and articular cartilage	Cancer
Byu4.00	[x]melanoma and other malignant neoplasms of skin	Cancer
Byu4100	[x]malignant melanoma of skin, unspecified	Cancer
Byu4300	[x]malignant neoplasm of skin, unspecified	Cancer
Byu5.00	[x]malignant neoplasm of mesothelial and soft tissue	Cancer
Byu5100	[x]mesothelioma, unspecified	Cancer
Byu5200	[x]kaposi's sarcoma of multiple organs	Cancer
Byu5300	[x]kaposi's sarcoma, unspecified	Cancer
Byu5700	[x]malignant neoplasm of peritoneum, unspecified	Cancer
Byu5A00	[x]malignant neoplasm overlapping lesion of skin	Cancer
Byu5B00	[x]kaposi's sarcoma of other sites	Cancer
Byu6.00	[x]malignant neoplasm of breast	Cancer
Byu7.00	[x]malignant neoplasm of female genital organs	Cancer
Byu7000	[x]malignant neoplasm of uterine adnexa, unspecified	Cancer
Byu7300	[x]malignant neoplasm of female genital organ, unspecified	Cancer
Byu8.00	[x]malignant neoplasm of male genital organs	Cancer
Byu8200	[x]malignant neoplasm of male genital organ, unspecified	Cancer
Byu9.00	[x]malignant neoplasm of urinary tract	Cancer
Byu9000	[x]malignant neoplasm of urinary organ, unspecified	Cancer
ByuA200	[x]malignant neoplasm of meninges, unspecified	Cancer
ByuB.00	[x]malignant neoplasm of thyroid and other endocrine glands	Cancer
ByuB100	[x]malignant neoplasm of endocrine gland, unspecified	Cancer
ByuC000	[x]malignant neoplasm of other specified sites	Cancer
ByuC700	[x]secondary malignant neoplasm of other specified sites	Cancer

ByuC800	[x]malignant neoplasm without specification of site	Cancer
ByuD000	[x]other hodgkin's disease	Cancer
ByuD100	[x]other types of follicular non-hodgkin's lymphoma	Cancer
ByuD200	[x]other types of diffuse non-hodgkin's lymphoma	Cancer
ByuD300	[x]other specified types of non-hodgkin's lymphoma	Cancer
ByuD400	[x]other malignant immunoproliferative diseases	Cancer
ByuD500	[x]other lymphoid leukaemia	Cancer
ByuD600	[x]other myeloid leukaemia	Cancer
ByuD700	[x]other monocytic leukaemia	Cancer
ByuD800	[x]other specified leukaemias	Cancer
ByuD900	[x]other leukaemia of unspecified cell type	Cancer
ByuDC00	[x]diffuse non-hodgkin's lymphoma, unspecified	Cancer
ByuDE00	[x]unspecified b-cell non-hodgkin's lymphoma	Cancer
ByuF100	[x]carcinoma in situ of other specified digestive organs	Cancer
ByuF300	[x]carcinoma in situ of other parts of respiratory system	Cancer
ByuF600	[x]melanoma in situ of other sites	Cancer
ByuF900	[x]carcinoma in situ of skin, unspecified	Cancer
ByuFA00	[x]carcinoma in situ of other parts of cervix	Cancer
ByuFF00	[x]melanoma in situ, unspecified	Cancer
ByuFG00	[x]other carcinoma in situ of breast	Cancer
Bz...00	neoplasms nos	Cancer
C333.00	macroglobulinaemia	Cancer
C333000	waldenstrom's macroglobulinaemia	Cancer
C333z00	macroglobulinaemia nos	Cancer
C37y700	histiocytosis, unspecified	Cancer
BBm9.00	[m] monocytoid b-cell lymphoma	Cancer
BBA2.00	[m]acinar cell carcinoma	Cancer
BBZG.11	[m]adamantinoma, malignant	Cancer
BB5N100	[m]adenocarcinoma in adenomatous polposis coli	Cancer
BB5L100	[m]adenocarcinoma in adenomatous polyp	Cancer
BB51.00	[m]adenocarcinoma in situ	Cancer
BBB5.00	[m]adenocarcinoma with apocrine metaplasia	Cancer
BBB3.00	[m]adenocarcinoma with cartilaginous and osseous metaplasia	Cancer
BBB4.00	[m]adenocarcinoma with spindle cell metaplasia	Cancer
BBB2.00	[m]adenocarcinoma with squamous metaplasia	Cancer
BB57.00	[m]adenocarcinoma, intestinal type	Cancer
BB5..11	[m]adenocarcinomas	Cancer
BB5J.00	[m]adenoid cystic carcinoma	Cancer
BB2G.00	[m]adenoid squamous cell carcinoma	Cancer
BBB1.00	[m]adenolymphoma	Cancer
BB5N.11	[m]adenoma or or adenocarcinoma in polyposis coli	Cancer
BB5..00	[m]adenomas and adenocarcinomas	Cancer
BBL7111	[m]adenosarcoma	Cancer
BBB0.00	[m]adenosquamous carcinoma	Cancer
BB5h100	[m]adrenal cortical carcinoma	Cancer
BB5S400	[m]alveolar adenocarcinoma	Cancer

BB5S211	[m]alveolar cell carcinoma	Cancer
BBK3700	[m]alveolar rhabdomyosarcoma	Cancer
BBEA.00	[m]amelanotic melanoma	Cancer
BBZN.00	[m]ameloblastic fibrosarcoma	Cancer
BBZC.00	[m]ameloblastic odontosarcoma	Cancer
BBZG.00	[m]ameloblastoma, malignant	Cancer
BBK1100	[m]angiomyosarcoma	Cancer
BBT1.11	[m]angiosarcoma	Cancer
BB62100	[m]apocrine adenocarcinoma	Cancer
BBE4.00	[m]balloon cell melanoma	Cancer
BB34.00	[m]basal cell carcinoma, fibroepithelial type	Cancer
BB33.00	[m]basal cell carcinoma, morphoea type	Cancer
BB48.00	[m]basaloid carcinoma	Cancer
BB5V700	[m]basophil carcinoma	Cancer
BB35.00	[m]basosquamous carcinoma	Cancer
BB5D111	[m]bile duct carcinoma	Cancer
BB5D300	[m]bile duct cystadenocarcinoma	Cancer
BB5D.11	[m]biliary tract adenomas and adenocarcinomas	Cancer
BBM0100	[m]brenner tumour, malignant	Cancer
BBk0.11	[m]brill - symmers' disease	Cancer
BB5S212	[m]bronchiolar carcinoma	Cancer
BB9B.11	[m]c cell carcinoma	Cancer
BB11.00	[m]carcinoma in situ nos	Cancer
BB58.00	[m]carcinoma, diffuse type	Cancer
BBLA.00	[m]carcinosarcoma, embryonal type	Cancer
BB5D100	[m]cholangiocarcinoma	Cancer
BBV2.00	[m]chondroblastic osteosarcoma	Cancer
BBW8.00	[m]chondroblastoma, malignant	Cancer
BBR3.00	[m]choriocarcinoma combined with teratoma	Cancer
BB5V100	[m]chromophobe carcinoma	Cancer
BBLJ.00	[m]clear cell sarcoma of kidney	Cancer
BB49.00	[m]cloacogenic carcinoma	Cancer
BB82111	[m]colloid adenocarcinoma	Cancer
BB92.00	[m]comedocarcinoma, noninfiltrating	Cancer
BBG8.11	[m]congenital fibrosarcoma	Cancer
BB5K.00	[m]cribriform carcinoma	Cancer
BB5J.11	[m]cylindroid adenocarcinoma	Cancer
BBM7.11	[m]cystosarcoma phyllodes, benign	Cancer
BBM9.00	[m]cystosarcoma phyllodes, malignant	Cancer
BBQ9.00	[m]dermoid cyst with malignant transformation	Cancer
BB91.11	[m]duct carcinoma nos	Cancer
BBK3600	[m]embryonal rhabdomyosarcoma	Cancer
BBLD.00	[m]embryonal sarcoma	Cancer
BBL0.00	[m]endometrial stromal sarcoma	Cancer
BB5j500	[m]endometrioid adenofibroma, malignant	Cancer
BB5j200	[m]endometrioid carcinoma	Cancer

BBY0.11	[m]endothelial bone sarcoma	Cancer
BB5V311	[m]eosinophil carcinoma	Cancer
BB29.11	[m]epidermoid carcinoma in situ	Cancer
BB2A.11	[m]epidermoid carcinoma nos	Cancer
BB2C.11	[m]epidermoid carcinoma, keratinising type	Cancer
BBEP.00	[m]epithelioid cell melanoma	Cancer
BBF6.00	[m]epithelioid cell sarcoma	Cancer
BBK0400	[m]epithelioid leiomyosarcoma	Cancer
BBP5.00	[m]epithelioid mesothelioma, malignant	Cancer
BB16.00	[m]epithelioma, malignant	Cancer
BBY0.00	[m]ewing's sarcoma	Cancer
BBV3.00	[m]fibroblastic osteosarcoma	Cancer
BBW4.11	[m]fibrochondrosarcoma	Cancer
BBJ1.11	[m]fibroliposarcoma	Cancer
BBG3.00	[m]fibromyxosarcoma	Cancer
BBGJ.00	[m]fibroxanthoma, malignant	Cancer
BB5f300	[m]follicular adenocarcinoma, trabecular type	Cancer
BB5f200	[m]follicular adenocarcinoma, well differentiated type	Cancer
BBk0.12	[m]follicular lymphosarcoma nos	Cancer
BB5C100	[m]gastrinoma, malignant	Cancer
BB82112	[m]gelatinous adenocarcinoma	Cancer
BB1B.00	[m]giant cell and spindle cell carcinoma	Cancer
BBX1.11	[m]giant cell bone sarcoma	Cancer
BB1C.00	[m]giant cell carcinoma	Cancer
BBX1.00	[m]giant cell tumour of bone, malignant	Cancer
BBk0.13	[m]giant follicular lymphoma	Cancer
BBb0.00	[m]glioma, malignant	Cancer
BBb0.12	[m]gliosarcoma	Cancer
BBDB.00	[m]glomangiosarcoma	Cancer
BBDB.11	[m]glomoid sarcoma	Cancer
BB5B500	[m]glucagonoma, malignant	Cancer
BB5b.00	[m]granular cell carcinoma	Cancer
BBf..00	[m]granular cell tumours and alveolar soft part sarcoma	Cancer
BBC4.00	[m]granulosa cell tumour, malignant	Cancer
BBT7100	[m]haemangioendothelioma, malignant	Cancer
BBTD200	[m]haemangiopericytoma, malignant	Cancer
BBT1.00	[m]haemangiosarcoma	Cancer
BBL8.00	[m]hepatoblastoma	Cancer
BB5D512	[m]hepatoma, malignant	Cancer
BBj0.00	[m]hodgkin's disease nos	Cancer
BBj4.00	[m]hodgkin's disease,lymphocytic depletion,diffuse fibrosis	Cancer
BBj6200	[m]hodgkin,s disease, nodular sclerosis, lymphocytic deplet	Cancer
BBj6000	[m]hodgkin,s disease, nodular sclerosis, lymphocytic predom	Cancer
BB5W111	[m]hurthle cell adenocarcinoma	Cancer
BBQ4.11	[m]infantile embryonal carcinoma	Cancer
BBG8.00	[m]infantile fibrosarcoma	Cancer

BB91.00	[m]infiltrating duct carcinoma	Cancer
BB9G.00	[m]infiltrating ductular carcinoma	Cancer
BB9H.00	[m]inflammatory carcinoma	Cancer
BB5B300	[m]insulinoma, malignant	Cancer
BB9M.00	[m]intracystic carcinoma nos	Cancer
BB90.00	[m]intraductal carcinoma, noninfiltrating nos	Cancer
BB29.12	[m]intraepidermal carcinoma nos	Cancer
BB11.11	[m]intraepithelial carcinoma nos	Cancer
BB29.13	[m]intraepithelial squamous cell carcinoma	Cancer
BBZ2.11	[m]intraosseous carcinoma	Cancer
BB5B100	[m]islet cell carcinoma	Cancer
BB94.00	[m]juvenile breast carcinoma	Cancer
BBW6.00	[m]juxtacortical chondrosarcoma	Cancer
BBV..11	[m]juxtacortical osteogenic sarcoma	Cancer
BBEG.11	[m]lentigo maligna melanoma	Cancer
BBd2.11	[m]leptomeningeal sarcoma	Cancer
BBCC100	[m]leydig cell tumour, malignant	Cancer
BBJ3.00	[m]liposarcoma, well differentiated type	Cancer
BB5D513	[m]liver cell carcinoma	Cancer
BB9E.00	[m]lobular carcinoma in situ	Cancer
BBgG.11	[m]lymphoblastic lymphosarcoma nos	Cancer
BBgG.13	[m]lymphoblastoma nos	Cancer
BB2M.00	[m]lymphoepithelial carcinoma	Cancer
BBg1.11	[m]lymphoma nos	Cancer
BBgz.00	[m]lymphoma, diffuse or nos	Cancer
BBk..00	[m]lymphomas, nodular or follicular	Cancer
BBg0.00	[m]lymphomatous tumour, benign	Cancer
BBg6.00	[m]lymphosarcoma nos	Cancer
BBgK.00	[m]malig lymphoma, follicular centre cell, non-cleaved nos	Cancer
BBgD.00	[m]malig lymphoma, lymphocytic, intermediate different nos	Cancer
BBk3.00	[m]malig lymphoma, lymphocytic, well differentiated,nodular	Cancer
N237300	pseudosarcomatous fibromatosis	Cancer
BBgN.00	[m]malign lymphoma,lymphocytic,intermediate differn, diffuse	Cancer
BBX3.00	[m]malignant giant cell tumour of soft parts	Cancer
BBg1.00	[m]malignant lymphoma nos	Cancer
BBgT.00	[m]malignant lymphoma, large cell, noncleaved, diffuse	Cancer
BBgG.00	[m]malignant lymphoma, lymphocytic, poorly different nos	Cancer
BBg2.00	[m]malignant lymphoma, non hodgkin's type	Cancer
BBgV.00	[m]malignant lymphoma, small cell, noncleaved, diffuse	Cancer
BBg4.00	[m]malignant lymphoma, stem cell type	Cancer
BBEM.00	[m]malignant melanoma in giant pigmented naevus	Cancer
BBEC.00	[m]malignant melanoma in junctional naevus	Cancer
BBQ7500	[m]malignant teratoma, intermediate type	Cancer
BBR4.00	[m]malignant teratoma, trophoblastic	Cancer
BBQ7400	[m]malignant teratoma, undifferentiated type	Cancer
BB0A.00	[m]malignant tumour, fusiform cell type	Cancer

BB09.00	[m]malignant tumour, giant cell type	Cancer
BB08.00	[m]malignant tumour, small cell type	Cancer
BBp1.00	[m]mast cell sarcoma	Cancer
N330900	osteoporosis in multiple myelomatosis	Cancer
BB9C.00	[m]medullary carcinoma with amyloid stroma	Cancer
BB9D.00	[m]medullary carcinoma with lymphoid stroma	Cancer
BBE1.11	[m]melanocarcinoma	Cancer
BBE1.12	[m]melanoma nos	Cancer
BBE1.13	[m]melanosarcoma nos	Cancer
BBdB.00	[m]meningeal sarcomatosis	Cancer
BBd2.00	[m]meningioma, malignant	Cancer
BBd2.12	[m]meningothelial sarcoma	Cancer
BBW9.00	[m]mesenchymal chondrosarcoma	Cancer
BBLC100	[m]mesenchymoma, malignant	Cancer
BBP7.00	[m]mesothelioma, biphasic type, malignant	Cancer
BBP1.00	[m]mesothelioma, malignant	Cancer
BB85100	[m]metastatic signet ring cell carcinoma	Cancer
BB36.00	[m]metatypical carcinoma	Cancer
BBm..00	[m]miscellaneous reticuloendothelial neoplasms	Cancer
BBK3300	[m]mixed cell rhabdomyosarcoma	Cancer
BBET.00	[m]mixed epithelioid and spindle melanoma	Cancer
BB5B600	[m]mixed islet cell and exocrine adenocarcinoma	Cancer
BBJ8.00	[m]mixed type liposarcoma	Cancer
BB84.00	[m]mucin-producing adenocarcinoma	Cancer
BB82100	[m]mucinous adenocarcinoma	Cancer
BB71.00	[m]mucoepidermoid carcinoma	Cancer
BB5V711	[m]mucoid cell carcinoma	Cancer
BB82114	[m]mucous adenocarcinoma	Cancer
BB32.00	[m]multicentric basal cell carcinoma	Cancer
BBn0.11	[m]multiple myeloma	Cancer
BBl..00	[m]mycosis fungoides	Cancer
BBl0.00	[m]mycosis fungoides	Cancer
BBn0.13	[m]myelomatosis	Cancer
BBK2100	[m]myosarcoma	Cancer
BBV9.00	[m]myxoid chondrosarcoma	Cancer
BBJ5.00	[m]myxoid liposarcoma	Cancer
BBJ5.12	[m]myxoliposarcoma	Cancer
BBE1.14	[m]naevocarcinoma	Cancer
BB04.00	[m]neoplasm, malig, uncertain whether primary or metastatic	Cancer
BB02.00	[m]neoplasm, malignant	Cancer
BB03.00	[m]neoplasm, metastatic	Cancer
BBe7.00	[m]neurilemmoma, malignant	Cancer
BBe2.00	[m]neurofibrosarcoma	Cancer
BBy2.00	[m]no microscopic confirmation tumour, clinically metastatic	Cancer
BBE2.00	[m]nodular melanoma	Cancer
BBg2.11	[m]non hodgkins lymphoma	Cancer

BB5f700	[m]nonencapsulated sclerosing carcinoma	Cancer
BB96.00	[m]noninfiltrating intraductal papillary adenocarcinoma	Cancer
BB1K.00	[m]oat cell carcinoma	Cancer
BBZN.11	[m]odontogenic fibrosarcoma	Cancer
BBZ2.00	[m]odontogenic tumour, malignant	Cancer
BB5W112	[m]oncytic adenocarcinoma	Cancer
BBV1.11	[m]osteoblastic sarcoma	Cancer
BBV1.12	[m]osteochondrosarcoma	Cancer
BBX1.12	[m]osteoclastoma, malignant	Cancer
BBV1.13	[m]osteogenic sarcoma nos	Cancer
BBV5.00	[m]osteosarcoma in paget's disease of bone	Cancer
BB81.11	[m]ovarian cystadenoma or carcinoma	Cancer
BB5W100	[m]oxyphilic adenocarcinoma	Cancer
BB9K.00	[m]paget's disease and infiltrating breast duct carcinoma	Cancer
BB9J.11	[m]paget's disease, breast	Cancer
BB5f600	[m]papillary and follicular adenocarcinoma	Cancer
BB21.00	[m]papillary carcinoma in situ	Cancer
BB26.11	[m]papillary epidermoid carcinoma	Cancer
BB81H00	[m]papillary mucinous cystadenocarcinoma	Cancer
BB81800	[m]papillary serous cystadenocarcinoma	Cancer
BB26.00	[m]papillary squamous cell carcinoma	Cancer
BB4A.00	[m]papillary transitional cell carcinoma	Cancer
BBD1.00	[m]paraganglioma, malignant	Cancer
BBV..12	[m]parosteal osteosarcoma	Cancer
BBV..13	[m]periosteal osteogenic sarcoma	Cancer
BBDA.00	[m]phaeochromocytoma, malignant	Cancer
BBn3.00	[m]plasma cell tumour, malignant	Cancer
BBn2.00	[m]plasmacytoma nos	Cancer
BBn1.11	[m]plasmacytoma, benign	Cancer
BB1A.00	[m]pleomorphic carcinoma	Cancer
BBF4.11	[m]pleomorphic cell sarcoma	Cancer
BBJ7.00	[m]pleomorphic liposarcoma	Cancer
BBK3200	[m]pleomorphic rhabdomyosarcoma	Cancer
BB1F.00	[m]polygonal cell carcinoma	Cancer
BB81E11	[m]pseudomucinous adenocarcinoma	Cancer
BB1E.00	[m]pseudosarcomatous carcinoma	Cancer
BB5a000	[m]renal cell carcinoma	Cancer
BBh0.00	[m]reticulosarcoma nos	Cancer
BBh2.00	[m]reticulosarcoma, nodular	Cancer
BBh..00	[m]reticulosarcomas	Cancer
BB1J.12	[m]round cell carcinoma	Cancer
BBJ6.00	[m]round cell liposarcoma	Cancer
BBF5.11	[m]round cell sarcoma	Cancer
BBK3611	[m]sarcoma botryoides	Cancer
BB46.00	[m]schneiderian carcinoma	Cancer
BBe7.11	[m]schwannoma, malignant	Cancer

BB54.00	[m]scirrhous adenocarcinoma	Cancer
BB03.11	[m]secondary neoplasm	Cancer
BB94.11	[m]secretory breast carcinoma	Cancer
BB81B00	[m]serous surface papillary carcinoma	Cancer
BBCA.00	[m]sertoli cell carcinoma	Cancer
BB85.00	[m]signet ring carcinoma	Cancer
BB85000	[m]signet ring cell carcinoma	Cancer
BB1L.00	[m]small cell carcinoma, fusiform cell type	Cancer
BBF5.00	[m]small cell sarcoma	Cancer
BB1D.00	[m]spindle cell carcinoma	Cancer
BBES.00	[m]spindle cell melanoma, type b	Cancer
BBF3.00	[m]spindle cell sarcoma	Cancer
BB2A.12	[m]spinous cell carcinoma	Cancer
BB2D.00	[m]squamous cell carcinoma, large cell, non-keratinising	Cancer
BB2J.00	[m]squamous cell carcinoma, microinvasive	Cancer
BB2E.00	[m]squamous cell carcinoma, small cell, non-keratinising	Cancer
BB2F.00	[m]squamous cell carcinoma, spindle cell type	Cancer
B911000	malignant hydatidiform mole	Cancer
BBQA100	[m]struma ovarii, malignant	Cancer
BB56.00	[m]superficial spreading adenocarcinoma	Cancer
BBEH.00	[m]superficial spreading melanoma	Cancer
BBN4.00	[m]synovial sarcoma, biphasic type	Cancer
BBN2.00	[m]synovial sarcoma, spindle cell type	Cancer
BBV4.00	[m]telangiectatic osteosarcoma	Cancer
BBQ7213	[m]teratoblastoma, malignant	Cancer
BBQ7300	[m]teratocarcinoma	Cancer
BBB6100	[m]thymoma, malignant	Cancer
BB5F.00	[m]trabecular adenocarcinoma	Cancer
BB42.00	[m]transitional cell carcinoma in situ	Cancer
BB47.00	[m]transitional cell carcinoma, spindle cell type	Cancer
BB4..00	[m]transitional cell papillomas and carcinomas	Cancer
BB5M100	[m]tubular adenocarcinoma	Cancer
BB07.00	[m]tumour cells, malignant	Cancer
BB43.11	[m]urothelial carcinoma	Cancer
BB24.11	[m]verrucous epidermoid carcinoma	Cancer
BB24.12	[m]verrucous squamous cell carcinoma	Cancer
BB5U200	[m]villous adenocarcinoma	Cancer
BBmK.00	[m]waldenstrom's macroglobulinaemia	Cancer
B828.00	melanoma in situ of skin	Cancer
ZV67700	[v]follow-up exam after radiotherapy for malignant neoplasm	Cancer
ZV67600	[v]follow-up examination aft surgery for malignant neoplasm	Cancer
ZV67A00	[v]folow-up exam aft other treatment for malignant neoplasm	Cancer
ZV67B00	[v]folow-up exam aft unspec treatment for malignant neoplasm	Cancer
B150300	hepatocellular carcinoma	Cancer
B150100	hepatoblastoma of liver	Cancer
B150200	primary angiosarcoma of liver	Cancer

ZV10413	[v]personal history malignant neoplasm - male genital organ	Cancer
B62x000	t-zone lymphoma	Cancer
B62x100	lymphoepithelioid lymphoma	Cancer
ZV10000	[v]personal history of malig neop of gastrointestinal tract	Cancer
ZV10012	[v]personal history of malig neop of gastrointestinal tract	Cancer
ZV10100	[v]personal history of malig neop of trachea/bronchus/lung	Cancer
ZV10200	[v]personal history of malig neop other intrathoracic organ	Cancer
B62x600	true histiocytic lymphoma	Cancer
B630.11	kahler's disease	Cancer
B630.12	myelomatosis	Cancer
B630.00	multiple myeloma	Cancer
B675.00	acute myelofibrosis	Cancer
208 RV	polycythaemia rubra vera	Cancer
ZV10400	[v]personal history of malignant neoplasm of genital organ	Cancer
ZV10512	[v]personal history of malignant neoplasm of kidney	Cancer
ZV10y16	[v]personal history of malignant neoplasm of tongue	Cancer
ZV10700	[v]personal history other lymphatic/haematopoietic neoplasm	Cancer
C37y800	haemophagocytic lymphohistiocytosis	Cancer
B576200	malignant ascites	Cancer
4M72.00	clark melanoma level 3	Cancer
ByuC500	[x]2ndry malignant neoplasm/bladder+oth+unsp urinary organs	Cancer
ByuC600	[x]2ndry malignant neoplasm/oth+unspec parts/nervous system	Cancer
ByuC200	[x]2ndry+unspcf malignant neoplasm lymph nodes/multi regions	Cancer
ByuFC00	[x]carcinoma in situ of oth+unspecified male genital organs	Cancer
4M73.00	clark melanoma level 4	Cancer
BBGJ.11	[m]fibroxanthosarcoma	Cancer
B141.11	carcinoma of rectum	Cancer
B141.12	rectal carcinoma	Cancer
B338.00	squamous cell carcinoma of skin	Cancer
B595.00	malignant tumour of unknown origin	Cancer
B4...11	carcinoma of genitourinary organ	Cancer
B3...12	sarcoma of bone and connective tissue	Cancer
B3...11	carcinoma of bone, connective tissue, skin and breast	Cancer
B0...11	carcinoma of lip, oral cavity and pharynx	Cancer
B2...11	carcinoma of respiratory tract and intrathoracic organs	Cancer
B134.11	carcinoma of caecum	Cancer
B142.11	anal carcinoma	Cancer
B161211	carcinoma common bile duct	Cancer
B305.11	malignant neoplasm of carpal bones	Cancer
B305.12	malignant neoplasm of metacarpal bones	Cancer
B47z.11	seminoma of testis	Cancer
B57..12	secondary carcinoma of respiratory and/or digestive systems	Cancer
B58..11	secondary carcinoma of other specified sites	Cancer
B5...11	carcinoma of other and unspecified sites	Cancer
B822.11	carcinoma in situ of ear	Cancer
Byu5800	[x]mal neoplasm/connective+soft tissue of trunk,unspecified	Cancer

Byu5500	[x]mal neoplasm/overlap les/periph nerv+autonomic nerv systm	Cancer
Byu3000	[x]mal neoplasm/overlap lesion/bone+articular cartilage/limb	Cancer
Byu5600	[x]mal neoplasm/periph nerves+autonomic nervous system,unspc	Cancer
ByuA300	[x]malig neopl, overlap lesion brain & other part of cns	Cancer
Byu4000	[x]malignant melanoma of other+unspecified parts of face	Cancer
Byu2300	[x]malignant neopl/overlapping les/resp+intrathoracic organs	Cancer
ByuA.00	[x]malignant neoplasm of eye, brain and other parts of cent	Cancer
ByuC.00	[x]malignant neoplasm of ill-defined, secondary and unspeci	Cancer
Byu0.00	[x]malignant neoplasm of lip, oral cavity and pharynx	Cancer
Byu2.00	[x]malignant neoplasm of respiratory and intrathoracic orga	Cancer
Byu3300	[x]malignant neoplasm/bone+articular cartilage, unspecified	Cancer
Byu3100	[x]malignant neoplasm/bones+articular cartilage/limb,unspfd	Cancer
ByuA100	[x]malignant neoplasm/central nervous system, unspecified	Cancer
Byu5900	[x]malignant neoplasm/connective + soft tissue,unspecified	Cancer
Byu2400	[x]malignant neoplasm/ill-defined sites within resp system	Cancer
ByuA000	[x]malignant neoplasm/other and unspecified cranial nerves	Cancer
Byu7100	[x]malignant neoplasm/other specified female genital organs	Cancer
Byu8000	[x]malignant neoplasm/other specified male genital organs	Cancer
Byu3200	[x]malignant neoplasm/overlap lesion/bone+articulr cartilage	Cancer
Byu2100	[x]malignant neoplasm/overlap lesion/heart,mediastinm+pleura	Cancer
ByuC100	[x]malignant neoplasm/overlap lesion/other+ill-defined sites	Cancer
Byu7200	[x]malignant neoplasm/overlapping lesion/feml genital organs	Cancer
Byu8100	[x]malignant neoplasm/overlapping lesion/male genital organs	Cancer
Byu5400	[x]malignant neoplasm/peripheral nerves of trunk,unspecified	Cancer
Byu2200	[x]malignant neoplasm/upper resp tract, part unspecified	Cancer
ByuD.00	[x]malignant neoplasms of lymphoid, haematopoietic and rela	Cancer
ByuE.00	[x]malignant neoplasms/independent (primary) multiple sites	Cancer
ByuE000	[x]malignant neoplasms/independent(primary)multiple sites	Cancer
Byu1300	[x]malignant neoplsm/ill-defin sites within digestive system	Cancer
B62xX00	oth and unspecif peripheral & cutaneous t-cell lymphomas	Cancer
142..00	h/o: malignant neoplasm (*)	Cancer
Byu5011	[x]mesothelioma of lung	Cancer
ByuDF11	[x]non-hodgkin's lymphoma nos	Cancer
B1...00	malignant neoplasm of digestive organs and peritoneum	Cancer
B10z.00	malignant neoplasm of oesophagus nos	Cancer
B13z.00	malignant neoplasm of colon nos	Cancer
B222.00	malignant neoplasm of upper lobe, bronchus or lung	Cancer
B22z.00	malignant neoplasm of bronchus or lung nos	Cancer
B30..00	malignant neoplasm of bone and articular cartilage	Cancer
B305.00	malignant neoplasm of hand bones	Cancer
B308.00	malignant neoplasm of short bones of leg	Cancer
B33..00	other malignant neoplasm of skin	Cancer
B454.00	malignant neoplasm of vulva unspecified	Cancer
B47z.00	malignant neoplasm of testis nos	Cancer
B590.00	disseminated malignancy nos	Cancer
B6...00	malignant neoplasm of lymphatic and haemopoietic tissue	Cancer

B62zz00	lymphoid and histiocytic malignancy nos	Cancer
B832.00	carcinoma in situ of other and unspecified parts of uterus	Cancer
BB5N.00	[m]adenomatous and adenocarcinomatous polyps of colon	Cancer
BBE1.00	[m]malignant melanoma nos	Cancer
BBV..00	[m]osteomas and osteosarcomas	Cancer
BBj0.11	[m]lymphogranuloma, malignant	Cancer
BBk0.00	[m]malignant lymphoma, nodular nos	Cancer
Byu5000	[x]mesothelioma of other sites	Cancer
B00..11	carcinoma of lip	Cancer
ByuDD00	[x]oth and unspecif peripheral & cutaneous t-cell lymphomas	Cancer
Byu4200	[x]oth malignant neoplasm/skin of oth+unspecfd parts of face	Cancer
ByuDA00	[x]oth spcf mal neoplsm/lymphoid,haematopoietic+rltd tissue	Cancer
B0...00	malignant neoplasm of lip, oral cavity and pharynx	Cancer
B800.00	carcinoma in situ of lip, oral cavity and pharynx	Cancer
ByuDF00	[x]non-hodgkin's lymphoma, unspecified type	Cancer
B4...00	malignant neoplasm of genitourinary organ	Cancer
B5...00	malignant neoplasm of other and unspecified sites	Cancer
B58y.00	secondary malignant neoplasm of other specified sites	Cancer
B808.00	carcinoma in situ of liver and biliary system	Cancer
B56..00	secondary and unspecified malignant neoplasm of lymph nodes	Cancer
B003.00	malignant neoplasm of lower lip, inner aspect	Cancer
B30..12	osteoma	Cancer
B594.00	secondary malignant neoplasm of unknown site	Cancer
1739B	epithelioma basal cell	Cancer
B454.11	primary vulval cancer	Cancer
B62zz11	immunoproliferative neoplasm	Cancer
142..12	h/o: carcinoma	Cancer
B339.00	dermatofibrosarcoma protuberans	Cancer
B627900	mucosa-associated lymphoma	Cancer
B627000	follicular non-hodgkin's small cleaved cell lymphoma	Cancer
B627200	follicular non-hodgkin's large cell lymphoma	Cancer
B62E600	enteropathy-associated t-cell lymphoma	Cancer
B222.11	pancoast's syndrome	Cancer
ByuC300	[x]secondary malignant neoplasm/oth+unspc respiratory organs	Cancer
ByuC400	[x]secondary malignant neoplasm/oth+unspcfd digestive organs	Cancer
B540.11	phaeochromocytoma	Cancer
B627D00	diffuse non-hodgkin's centroblastic lymphoma	Cancer
B623.00	malignant histiocytosis	Cancer
B62x400	malignant reticulosis	Cancer
B590.11	carcinomatosis	Cancer
B6y1.00	myelosclerosis with myeloid metaplasia	Cancer
D212000	anaemia in ovarian carcinoma	Cancer
B215.00	malignant neoplasm of epiglottis nos	Cancer
B627C00	follicular non-hodgkin's lymphoma	Cancer
B936.12	plasmacytoma nos	Cancer
C354A00	metastatic calcification	Cancer

B007.00	malignant neoplasm of lip, unspecified	Cancer
BBjz.00	[m]hodgkin's disease nos	Cancer
B325700	malignant melanoma of back	Cancer
B593.00	primary malignant neoplasm of unknown site	Cancer
B331000	malignant neoplasm of canthus	Cancer
B332200	malignant neoplasm of pinna nec	Cancer
B335A00	malignant neoplasm of skin of scapular region	Cancer
B670.00	acute erythraemia and erythroleukaemia	Cancer
B325800	malignant melanoma of chest wall	Cancer
B331100	malignant neoplasm of upper eyelid	Cancer
B331200	malignant neoplasm of lower eyelid	Cancer
B630300	lambda light chain myeloma	Cancer
B470200	seminoma of undescended testis	Cancer
B471000	seminoma of descended testis	Cancer
B690.00	acute myelomonocytic leukaemia	Cancer
B36..00	local recurrence of malignant tumour of breast	Cancer
B498.00	local recurrence of malignant tumour of urinary bladder	Cancer
B828800	melanoma in situ of back of hand	Cancer
B828900	melanoma in situ of back	Cancer
1D18.00	pain from metastases	Cancer
B65y100	acute promyelocytic leukaemia	Cancer
ZV10.00	[v]personal history of malignant neoplasm	Cancer
ZV10300	[v]personal history of malignant neoplasm of breast	Cancer
ZV10412	[v]personal history of malignant neoplasm of genital organ	Cancer
ZV10500	[v]personal history of malignant neoplasm of urinary organ	Cancer
ZV10600	[v]personal history of leukaemia	Cancer
ZV10y00	[v]personal history of other specified malignant neoplasm	Cancer
ZV10z00	[v]personal history of unspecified malignant neoplasm	Cancer
B471z00	malignant neoplasm of descended testis nos	Cancer
B691.00	chronic myelomonocytic leukaemia	Cancer
B18y700	malignant neoplasm of mesentery	Cancer
B18y300	malignant neoplasm of omentum	Cancer
B451.00	malignant neoplasm of labia majora	Cancer
B452.00	malignant neoplasm of labia minora	Cancer
B511.00	malignant neoplasm of frontal lobe	Cancer
B512.00	malignant neoplasm of temporal lobe	Cancer
B513.00	malignant neoplasm of parietal lobe	Cancer
B514.00	malignant neoplasm of occipital lobe	Cancer
B515.00	malignant neoplasm of cerebral ventricles	Cancer
B521.00	malignant neoplasm of cerebral meninges	Cancer
B523.00	malignant neoplasm of spinal meninges	Cancer
B525.00	malignant neoplasm of cauda equina	Cancer
B33z100	naevoid basal cell carcinoma syndrome	Cancer
4M20.00	lymphoma stage i	Cancer
B640.00	acute lymphoid leukaemia	Cancer
B800500	carcinoma in situ of cheek	Cancer

B810800	carcinoma in situ of vocal fold - glottis	Cancer
B800.11	carcinoma in situ of oral cavity	Cancer
B802400	carcinoma in situ of pyloric canal	Cancer
B624600	leukaemic reticuloendotheliosis of intrapelvic lymph nodes	Cancer
B624200	leukaemic reticuloendotheliosis of intrathoracic lymph nodes	Cancer
B58y000	secondary malignant neoplasm of breast	Cancer
B58y411	secondary cancer of the vulva	Cancer
B602500	burkitt's lymphoma of lymph nodes of inguinal region and leg	Cancer
B34..11	ca female breast	Cancer
B....11	cancers	Cancer
B160.11	carcinoma gallbladder	Cancer
B808.11	carcinoma in situ of biliary system	Cancer
B80..00	carcinoma in situ of digestive organs	Cancer
B808300	carcinoma in situ of gall bladder	Cancer
B800300	carcinoma in situ of gums	Cancer
B81y.11	carcinoma in situ of nasal sinuses	Cancer
B81y.00	carcinoma in situ of other specified part respiratory system	Cancer
B800200	carcinoma in situ of salivary glands	Cancer
B827.11	carcinoma in situ of skin of leg	Cancer
B823.00	carcinoma in situ of skin of other parts of face	Cancer
B81yz00	carcinoma in situ of specified parts respiratory system nos	Cancer
B833.00	carcinoma in situ other and unspecified female genital organ	Cancer
B836.00	carcinoma in situ other and unspecified male genital organs	Cancer
B807z00	carcinoma in situ other and unspecified small intestine nos	Cancer
B822.00	carcinoma in situ skin of ear and external auricular canal	Cancer
B822z00	carcinoma in situ skin of ear/external auricular canal nos	Cancer
B1...11	carcinoma of digestive organs and peritoneum	Cancer
B583200	cerebral metastasis	Cancer
B51..11	cerebral tumour - malignant	Cancer
B41..11	cervical carcinoma (uterus)	Cancer
B30..11	chondroma	Cancer
B420.00	choriocarcinoma	Cancer
B671.00	chronic erythraemia	Cancer
B651.11	chronic granulocytic leukaemia	Cancer
B641.11	chronic lymphatic leukaemia	Cancer
B33z.11	squamous cell carcinoma of skin nos	Cancer
BB2A.13	[m]squamous cell carcinoma of skin nos	Cancer
B831.11	cin iii - carcinoma in situ of cervix	Cancer
B13z.11	colonic cancer	Cancer
B627.00	non - hodgkin's lymphoma	Cancer
7G05D00	excision biopsy of basal cell carcinoma	Cancer
B627500	diffuse non-hodgkin mixed sml & lge cell (diffuse) lymphoma	Cancer
B627700	diffuse non-hodgkin's lymphoblastic (diffuse) lymphoma	Cancer
B627X00	diffuse non-hodgkin's lymphoma, unspecified	Cancer
B118.00	siewert type ii adenocarcinoma	Cancer
B119.00	siewert type iii adenocarcinoma	Cancer

B107.00	siewert type i adenocarcinoma	Cancer
B33..12	epithelioma	Cancer
7G03K00	excision malignant skin tumour	Cancer
D401100	familial erythrophagocytic lymph histiocytosis	Cancer
B620.00	nodular lymphoma (brill - symmers disease)	Cancer
B620500	nodular lymphoma of lymph nodes of inguinal region and leg	Cancer
B....00	neoplasms	Cancer
F396200	myopathy due to malignant disease	Cancer
B6y0.11	myeloproliferative disease	Cancer
B621500	mycosis fungoides of lymph nodes of inguinal region and leg	Cancer
B621100	mycosis fungoides of the lymph nodes of head, face and neck	Cancer
B63..00	multiple myeloma and immunoproliferative neoplasms	Cancer
B6y1.11	megakaryocytic myelosclerosis	Cancer
B57..11	metastases of respiratory and/or digestive systems	Cancer
B232.00	mesothelioma of pleura	Cancer
B828200	melanoma in situ of ear and external auricular canal	Cancer
B828600	melanoma in situ of lower limb, including hip	Cancer
B828X00	melanoma in situ of other and unspecified parts of face	Cancer
B828500	melanoma in situ of upper limb, including shoulder	Cancer
B828W00	melanoma in situ, unspecified	Cancer
B673.00	mast cell leukaemia	Cancer
B626500	mast cell malignancy of lymph nodes inguinal region and leg	Cancer
B935.12	mastocytoma nos	Cancer
B204.00	malignant neoplasm of frontal sinus	Cancer
B023.00	malignant neoplasm, overlapping lesion of major saliv gland	Cancer
B524600	malignant neoplasm,overlap lesion periph nerve & auton ns	Cancer
B54X.00	malignant neoplasm-pluriglandular involvement,unspecified	Cancer
B30X.00	malignant neoplasm/bones+articular cartilage/limb,unspfd	Cancer
B30W.00	malignant neoplasm/overlap lesion/bone+articulr cartilage	Cancer
B45X.00	malignant neoplasm/overlapping lesion/feml genital organs	Cancer
B524X00	malignant neoplasm/peripheral nerves of trunk,unspecified	Cancer
B62x300	malignant reticuloendotheliosis	Cancer
B302.00	malignant neoplasm of vertebral column	Cancer
B16y.00	malignant neoplasm other gallbladder/extrahepatic bile duct	Cancer
B1zy.00	malignant neoplasm other spec digestive tract and peritoneum	Cancer
B33X.00	malignant neoplasm overlapping lesion of skin	Cancer
B542.00	malignant neoplasm pituitary gland and craniopharyngeal duct	Cancer
B073200	malignant neoplasm posterior margin nasal septum and choanae	Cancer
B14z.00	malignant neoplasm rectum,rectosigmoid junction and anus nos	Cancer
B332.00	malignant neoplasm skin of ear and external auricular canal	Cancer
B333.00	malignant neoplasm skin of other and unspecified parts face	Cancer
B333z00	malignant neoplasm skin other and unspec part of face nos	Cancer
B309.00	malignant neoplasm, overlap les bone and artic cart of limbs	Cancer
B317.00	malignant neoplasm, overlap lesion connective & soft tissue	Cancer
B26..00	malignant neoplasm, overlap lesion of resp & intrathor orgs	Cancer
B48y200	malignant neoplasm, overlapping lesion male genital orgs	Cancer

B51y200	malignant neoplasm, overlapping lesion of brain	Cancer
B030.00	malignant neoplasm of upper gum	Cancer
B002000	malignant neoplasm of upper lip, buccal aspect	Cancer
B000000	malignant neoplasm of upper lip, external	Cancer
B497.00	malignant neoplasm of urachus	Cancer
B4A2.00	malignant neoplasm of ureter	Cancer
B4A3.00	malignant neoplasm of urethra	Cancer
B054.00	malignant neoplasm of uvula	Cancer
B450.00	malignant neoplasm of vagina	Cancer
B33..15	malignant neoplasm of sweat gland	Cancer
B47..00	malignant neoplasm of testis	Cancer
B240.00	malignant neoplasm of thymus	Cancer
B53..00	malignant neoplasm of thyroid gland	Cancer
B01..00	malignant neoplasm of tongue	Cancer
B060.00	malignant neoplasm of tonsil	Cancer
B061.00	malignant neoplasm of tonsillar fossa	Cancer
B220.00	malignant neoplasm of trachea	Cancer
B131.00	malignant neoplasm of transverse colon	Cancer
B063.00	malignant neoplasm of vallecula	Cancer
B12..00	malignant neoplasm of small intestine and duodenum	Cancer
B053.00	malignant neoplasm of soft palate	Cancer
B485.00	malignant neoplasm of spermatic cord	Cancer
B205.00	malignant neoplasm of sphenoidal sinus	Cancer
B522.00	malignant neoplasm of spinal cord	Cancer
B137.00	malignant neoplasm of splenic flexure of colon	Cancer
B11..00	malignant neoplasm of stomach	Cancer
B212.00	malignant neoplasm of subglottis	Cancer
B022.00	malignant neoplasm of sublingual gland	Cancer
B200200	malignant neoplasm of septum of nose	Cancer
B133.00	malignant neoplasm of sigmoid colon	Cancer
B111000	malignant neoplasm of prepylorus of stomach	Cancer
B46..00	malignant neoplasm of prostate	Cancer
B081.00	malignant neoplasm of pyriform sinus	Cancer
B140.00	malignant neoplasm of rectosigmoid junction	Cancer
B4A1000	malignant neoplasm of renal calyces	Cancer
B4A1.00	malignant neoplasm of renal pelvis	Cancer
B470100	malignant neoplasm of retained testis	Cancer
B505.00	malignant neoplasm of retina	Cancer
B056.00	malignant neoplasm of retromolar area	Cancer
B553.00	malignant neoplasm of pelvis	Cancer
B524200	malignant neoplasm of peripheral nerve of low limb, incl hip	Cancer
B524100	malignant neoplasm of peripheral nerve,upp limb,incl should	Cancer
B524000	malignant neoplasm of peripheral nerves of head, face & neck	Cancer
B543.00	malignant neoplasm of pineal gland	Cancer
B542000	malignant neoplasm of pituitary gland	Cancer
B23..00	malignant neoplasm of pleura	Cancer

B080.00	malignant neoplasm of postcricoid region	Cancer
B486.00	malignant neoplasm of scrotum	Cancer
B12y.00	malignant neoplasm of other specified site small intestine	Cancer
B440.00	malignant neoplasm of ovary	Cancer
B225.00	malignant neoplasm of overlapping lesion of bronchus & lung	Cancer
B060200	malignant neoplasm of overlapping lesion of tonsil	Cancer
B45y000	malignant neoplasm of overlapping lesion of vulva	Cancer
B062300	malignant neoplasm of palatopharyngeal arch	Cancer
B17..00	malignant neoplasm of pancreas	Cancer
B541.00	malignant neoplasm of parathyroid gland	Cancer
B4A4.00	malignant neoplasm of paraurethral glands	Cancer
B020.00	malignant neoplasm of parotid gland	Cancer
B501.00	malignant neoplasm of orbit	Cancer
B06..00	malignant neoplasm of oropharynx	Cancer
B55z.00	malignant neoplasm of other and ill defined site nos	Cancer
B5y..00	malignant neoplasm of other and unspecified site os	Cancer
B0zy.00	malignant neoplasm of other sites lip, oral cavity, pharynx	Cancer
B308.11	malignant neoplasm of metatarsal bones of foot	Cancer
B241200	malignant neoplasm of myocardium	Cancer
B031.00	malignant neoplasm of lower gum	Cancer
B003200	malignant neoplasm of lower lip, mucosa	Cancer
B003300	malignant neoplasm of lower lip, oral aspect	Cancer
B001.00	malignant neoplasm of lower lip, vermilion border	Cancer
B201300	malignant neoplasm of mastoid air cells	Cancer
B202.00	malignant neoplasm of maxillary sinus	Cancer
B24X.00	malignant neoplasm of mediastinum, part unspecified	Cancer
B52X.00	malignant neoplasm of meninges, unspecified	Cancer
B507.00	malignant neoplasm of lacrimal duct	Cancer
B213.00	malignant neoplasm of laryngeal cartilage	Cancer
B0z2.00	malignant neoplasm of laryngopharynx	Cancer
B21..00	malignant neoplasm of larynx	Cancer
B072.00	malignant neoplasm of lateral wall of nasopharynx	Cancer
B016.00	malignant neoplasm of lingual tonsil	Cancer
B00..00	malignant neoplasm of lip	Cancer
B004000	malignant neoplasm of lip unspecified, buccal aspect	Cancer
B052.00	malignant neoplasm of hard palate	Cancer
B170.00	malignant neoplasm of head of pancreas	Cancer
B241.00	malignant neoplasm of heart	Cancer
B130.00	malignant neoplasm of hepatic flexure of colon	Cancer
B6...11	malignant neoplasm of histiocytic tissue	Cancer
B08..00	malignant neoplasm of hypopharynx	Cancer
B122.00	malignant neoplasm of ileum	Cancer
B121.00	malignant neoplasm of jejunum	Cancer
B04..00	malignant neoplasm of floor of mouth	Cancer
B160.00	malignant neoplasm of gallbladder	Cancer
B4y..00	malignant neoplasm of genitourinary organ os	Cancer

B481.00	malignant neoplasm of glans penis	Cancer
B062100	malignant neoplasm of glossopalatine fold	Cancer
B03..00	malignant neoplasm of gum	Cancer
B132.00	malignant neoplasm of descending colon	Cancer
B120.00	malignant neoplasm of duodenum	Cancer
B241000	malignant neoplasm of endocardium	Cancer
B484.00	malignant neoplasm of epididymis	Cancer
B161.00	malignant neoplasm of extrahepatic bile ducts	Cancer
B331.00	malignant neoplasm of eyelid including canthus	Cancer
B441.00	malignant neoplasm of fallopian tube	Cancer
B13..00	malignant neoplasm of colon	Cancer
B005.00	malignant neoplasm of commissure of lip	Cancer
B161200	malignant neoplasm of common bile duct	Cancer
B503.00	malignant neoplasm of conjunctiva	Cancer
B504.00	malignant neoplasm of cornea	Cancer
B303400	malignant neoplasm of costo-vertebral joint	Cancer
B520.00	malignant neoplasm of cranial nerves	Cancer
B050.00	malignant neoplasm of cheek mucosa	Cancer
B506.00	malignant neoplasm of choroid	Cancer
B135.00	malignant neoplasm of appendix	Cancer
B136.00	malignant neoplasm of ascending colon	Cancer
B010.00	malignant neoplasm of base of tongue	Cancer
B517.00	malignant neoplasm of brain stem	Cancer
B517z00	malignant neoplasm of brain stem nos	Cancer
B06y000	malignant neoplasm of branchial cleft	Cancer
B050.11	malignant neoplasm of buccal mucosa	Cancer
B326.00	malignant melanoma of upper limb and shoulder	Cancer
B082.00	malignant neoplasm aryepiglottic fold, hypopharyngeal aspect	Cancer
B510.00	malignant neoplasm cerebrum (excluding lobes and ventricles)	Cancer
B16..00	malignant neoplasm gallbladder and extrahepatic bile ducts	Cancer
B16z.00	malignant neoplasm gallbladder/extrahepatic bile ducts nos	Cancer
B6y..00	malignant neoplasm lymphatic or haematopoietic tissue os	Cancer
B520200	malignant neoplasm of acoustic nerve	Cancer
B162.00	malignant neoplasm of ampulla of vater	Cancer
B321.00	malignant melanoma of eyelid including canthus	Cancer
B315300	malig neopl of connective and soft tissue - sacrum or coccyx	Cancer
B524.00	malig neopl peripheral nerves and autonomic nervous system	Cancer
B52W.00	malig neopl, overlap lesion brain & other part of cns	Cancer
B314100	malig neoplasm of connective and soft tissues of lumb spine	Cancer
B313300	malig neoplasm of connective and soft tissues of thor spine	Cancer
B623100	malignant histiocytosis of lymph nodes head, face and neck	Cancer
B623500	malignant histiocytosis of lymph nodes inguinal and leg	Cancer
B623400	malignant histiocytosis of lymph nodes of axilla and arm	Cancer
SN5y100	malignant hypothermia due to anaesthetic	Cancer
B62y.00	malignant lymphoma nos	Cancer
B62y500	malignant lymphoma nos of lymph node inguinal region and leg	Cancer

B62y400	malignant lymphoma nos of lymph nodes of axilla and arm	Cancer
B31y.00	malig neop connective and soft tissue other specified site	Cancer
B311.00	malig neop connective and soft tissue upper limb/shoulder	Cancer
B311z00	malig neop connective soft tissue upper limb/shoulder nos	Cancer
B500.00	malig neop eyeball excl conjunctiva, cornea, retina, choroid	Cancer
B20..00	malig neop nasal cavities, middle ear and accessory sinuses	Cancer
B3...00	malig neop of bone, connective tissue, skin and breast	Cancer
B3z..00	malig neop of bone, connective tissue, skin and breast nos	Cancer
B3y..00	malig neop of bone, connective tissue, skin and breast os	Cancer
B310.00	malig neop of connective and soft tissue head, face and neck	Cancer
B314z00	malig neop of connective and soft tissue of abdomen nos	Cancer
B314000	malig neop of connective and soft tissue of abdominal wall	Cancer
B312600	malig neop of connective and soft tissue of great toe	Cancer
B312.00	malig neop of connective and soft tissue of hip and leg	Cancer
B315100	malig neop of connective and soft tissue of inguinal region	Cancer
B312300	malig neop of connective and soft tissue of lower leg	Cancer
B315z00	malig neop of connective and soft tissue of pelvis nos	Cancer
B313z00	malig neop of connective and soft tissue of thorax nos	Cancer
B312100	malig neop of connective and soft tissue thigh and upper leg	Cancer
B316.00	malig neop of connective and soft tissue trunk unspecified	Cancer
B54z.00	malig neop of endocrine gland or related structure nos	Cancer
B4A..00	malig neop of kidney and other unspecified urinary organs	Cancer
B45..00	malig neop of other and unspecified female genital organs	Cancer
B52..00	malig neop of other and unspecified parts of nervous system	Cancer
B54..00	malig neop of other endocrine glands and related structures	Cancer
B24y.00	malig neop of other site of heart, thymus and mediastinum	Cancer
B2...00	malig neop of respiratory tract and intrathoracic organs	Cancer
B304z00	malig neop of scapula and long bones of upper arm nos	Cancer
B2z0.00	malig neop of upper respiratory tract, part unspecified	Cancer
B1z..00	malig neop oth/ill-defined sites digestive tract/peritoneum	Cancer
B20y.00	malig neop other site nasal cavity, middle ear and sinuses	Cancer
B14y.00	malig neop other site rectum, rectosigmoid junction and anus	Cancer
B0z..00	malig neop other/ill-defined sites lip, oral cavity, pharynx	Cancer
B2z..00	malig neop other/ill-defined sites resp/intrathoracic organs	Cancer
B332z00	malig neop skin of ear and external auricular canal nos	Cancer
B25..00	malig neo, overlapping lesion of heart, mediastinum & pleura	Cancer
B201.00	malig neop auditory tube, middle ear and mastoid air cells	Cancer
B201z00	malig neop auditory tube, middle ear, mastoid air cells nos	Cancer
B310z00	malig neop connective and soft tissue head, face, neck nos	Cancer
B542z00	malig neop pituitary gland or craniopharyngeal duct nos	Cancer
B312z00	malig neop connective and soft tissue hip and leg nos	Cancer
B312200	malig neop connective and soft tissue of popliteal space	Cancer
B524W00	mal neoplasm/periph nerves+autonomic nervous system,unspc	Cancer
B67y000	lymphosarcoma cell leukaemia	Cancer
B601500	lymphosarcoma of lymph nodes of inguinal region and leg	Cancer
B56..11	lymph node metastases	Cancer

B22z.11	lung cancer	Cancer
B577.11	liver metastases	Cancer
B624800	leukaemic reticuloend of lymph nodes of multiple sites	Cancer
B624.00	leukaemic reticuloendotheliosis	Cancer
B624.11	leukaemic reticuloendotheliosis	Cancer
B625.00	letterer-siwe disease	Cancer
B625500	letterer-siwe disease of lymph nodes inguinal region and leg	Cancer
B625400	letterer-siwe disease of lymph nodes of axilla and arm	Cancer
B625100	letterer-siwe disease of lymph nodes of head, face and neck	Cancer
B624300	leukaemic reticuloend of intra-abdominal lymph nodes	Cancer
B624500	leukaemic reticuloend of lymph nodes inguinal region and leg	Cancer
B624400	leukaemic reticuloend of lymph nodes of axilla and arm	Cancer
B624100	leukaemic reticuloend of lymph nodes of head, face and neck	Cancer
B592X00	kaposi's sarcoma of multiple organs	Cancer
B59zX00	kaposi's sarcoma, unspecified	Cancer
B627C11	follicular lymphoma nos	Cancer
B627100	follicular non-hodg mixed sml cleavd & lge cell lymphoma	Cancer
B11..11	gastric neoplasm	Cancer
B62z300	unspec malig neop lymphoid/histiocytic intra-abdominal nodes	Cancer
B62z400	unspec malig neop lymphoid/histiocytic lymph node axilla/arm	Cancer
B62z100	unspec malig neop lymphoid/histiocytic lymph node head/neck	Cancer
B62z500	unspec malig neop lymphoid/histiocytic nodes inguinal/leg	Cancer
B62z600	unspec malig neop lymphoid/histiocytic of intrapelvic nodes	Cancer
B62z200	unspec malig neop lymphoid/histiocytic of intrathoracic node	Cancer
B62z800	unspec malig neop lymphoid/histiocytic of multiple sites	Cancer
B62z700	unspec malig neop lymphoid/histiocytic of spleen	Cancer
B62z000	unspec malig neop lymphoid/histiocytic of unspecified site	Cancer
B671.11	heilmeyer - schoner disease	Cancer
B627W00	unspecified b-cell non-hodgkin's lymphoma	Cancer
B935.11	histiocytic tumour nos	Cancer
B625.11	histiocytosis x (acute, progressive)	Cancer
A789700	hiv dis resulting oth types of non-hodgkin's lymphoma	Cancer
A789500	hiv disease resulting in kaposi's sarcoma	Cancer
A788W00	hiv disease resulting in unspecified malignant neoplasm	Cancer
B61z.00	hodgkin's disease nos	Cancer
B61z500	hodgkin's disease nos of lymph nodes inguinal region and leg	Cancer
B61z400	hodgkin's disease nos of lymph nodes of axilla and arm	Cancer
B613.00	hodgkin's disease, lymphocytic-histiocytic predominance	Cancer
B611500	hodgkin's granuloma lymph nodes of inguinal region and leg	Cancer
B616300	hodgkin's lymphocytic depletion intra-abdominal lymph nodes	Cancer
B616400	hodgkin's lymphocytic depletion lymph nodes axilla and arm	Cancer
B616500	hodgkin's lymphocytic depletion lymph nodes inguinal and leg	Cancer
B616800	hodgkin's lymphocytic depletion lymph nodes multiple sites	Cancer
B616100	hodgkin's lymphocytic depletion of head, face and neck	Cancer
B616600	hodgkin's lymphocytic depletion of intrapelvic lymph nodes	Cancer
B616200	hodgkin's lymphocytic depletion of intrathoracic lymph nodes	Cancer

B615300	hodgkin's mixed cellularity of intra-abdominal lymph nodes	Cancer
B615600	hodgkin's mixed cellularity of intrapelvic lymph nodes	Cancer
B615200	hodgkin's mixed cellularity of intrathoracic lymph nodes	Cancer
B615100	hodgkin's mixed cellularity of lymph nodes head, face, neck	Cancer
B615500	hodgkin's mixed cellularity of lymph nodes inguinal and leg	Cancer
B615400	hodgkin's mixed cellularity of lymph nodes of axilla and arm	Cancer
B615800	hodgkin's mixed cellularity of lymph nodes of multiple sites	Cancer
B614100	hodgkin's nodular sclerosis of head, face and neck	Cancer
B614500	hodgkin's nodular sclerosis of inguinal region and leg	Cancer
B614300	hodgkin's nodular sclerosis of intra-abdominal lymph nodes	Cancer
B614600	hodgkin's nodular sclerosis of intrapelvic lymph nodes	Cancer
B614200	hodgkin's nodular sclerosis of intrathoracic lymph nodes	Cancer
B614400	hodgkin's nodular sclerosis of lymph nodes of axilla and arm	Cancer
B614800	hodgkin's nodular sclerosis of lymph nodes of multiple sites	Cancer
B610.00	hodgkin's paragranuloma	Cancer
B610500	hodgkin's paragranuloma lymph nodes inguinal region and leg	Cancer
B610400	hodgkin's paragranuloma of lymph nodes of axilla and arm	Cancer
B610100	hodgkin's paragranuloma of lymph nodes of head, face, neck	Cancer
B612500	hodgkin's sarcoma of lymph nodes of inguinal region and leg	Cancer
B613400	hodgkin's, lymphocytic-histiocytic pred axilla and arm	Cancer
B613500	hodgkin's, lymphocytic-histiocytic pred inguinal and leg	Cancer
B613300	hodgkin's, lymphocytic-histiocytic pred intra-abdominal node	Cancer
B613600	hodgkin's, lymphocytic-histiocytic pred intrapelvic nodes	Cancer
B613200	hodgkin's, lymphocytic-histiocytic pred intrathoracic nodes	Cancer
B613100	hodgkin's, lymphocytic-histiocytic pred of head, face, neck	Cancer
B613800	hodgkin's, lymphocytic-histiocytic pred of multiple sites	Cancer
B613000	hodgkin's, lymphocytic-histiocytic predominance unspec site	Cancer
B1z0.11	cancer of bowel	Cancer
4M71.00	clark melanoma level 2	Cancer
4M2..00	lymphoma staging system	Cancer
1409C	lip carcinoma	Cancer
1419C	tongue carcinoma	Cancer
174 C	carcinoma breast	Cancer
188 C	carcinoma bladder	Cancer
209	myelofibrosis	Cancer
4M22.00	lymphoma stage iii	Cancer
C37yB00	langerhans' cell histiocytosis	Cancer
B430211	malignant neoplasm of endometrium	Cancer
2020FL	follicular lymphoma	Cancer
2021	mycosis fungoides	Cancer
203 T	myelomatosis	Cancer
203 A	solitary myeloma	Cancer
201	hodgkin's disease	Cancer
2072DG	di guglielmo's disease	Cancer
2001	lymphosarcoma	Cancer
1560C	carcinoma gallbladder	Cancer

203 KA	kahler's disease	Cancer
201 G	hodgkin's granuloma	Cancer
2022CL	chloroma	Cancer
2079MK	megakaryocytic leukaemia	Cancer
2059M	myeloid leukaemia	Cancer
1991MT	mesothelioma	Cancer
1890CH	hypernephroma	Cancer
1976A	malignant ascites	Cancer
9hF1.00	excepted from atrial fibrillation qual indic: inform dissent	Cardiac arrythmia
G562100	left posterior fascicular block	Cardiac arrythmia
G562.11	left bundle branch block	Cardiac arrythmia
G566000	sinoatrial block	Cardiac arrythmia
G571.00	paroxysmal ventricular tachycardia	Cardiac arrythmia
G570.00	paroxysmal supraventricular tachycardia	Cardiac arrythmia
G576100	supraventricular ectopic beats	Cardiac arrythmia
G57y600	nodal rhythm disorder	Cardiac arrythmia
G574000	ventricular fibrillation	Cardiac arrythmia
ZV53013	[v]fitting or adjustment of neuropacemaker device	Cardiac arrythmia
G565500	bifascicular block	Cardiac arrythmia
G57..00	cardiac dysrhythmias	Cardiac arrythmia
G57..11	cardiac arrhythmias	Cardiac arrythmia
G561311	mobitz type 1 second degree atrioventricular block	Cardiac arrythmia
G56y400	right fascicular block	Cardiac arrythmia
G567400	wolff-parkinson-white syndrome	Cardiac arrythmia
G565400	trifascicular block	Cardiac arrythmia
7936G00	implantat intraven single chamber cardiac pacemaker system	Cardiac arrythmia
7936H00	implantat intravenous dual chamber cardiac pacemaker system	Cardiac arrythmia
7936J00	implantat intravenous biventricular cardiac pacemaker system	Cardiac arrythmia
793M100	perc transluminal ablation of atrial wall for atrial flutter	Cardiac arrythmia
793M300	perc translum ablat conduct sys heart for atrial flutter nec	Cardiac arrythmia
G565200	right bbb with left anterior fascicular block	Cardiac arrythmia
G565100	right bbb with left posterior fascicular block	Cardiac arrythmia
G566200	right fascicular block	Cardiac arrythmia
8HRF.00	referral for cardiac pacemaker check	Cardiac arrythmia
G55A.00	tachycardiomyopathy	Cardiac arrythmia
329B.00	ecg: trifascicular block	Cardiac arrythmia
329C.00	ecg: bifascicular block	Cardiac arrythmia
329D.00	ecg: left anterior fascicular block	Cardiac arrythmia
329E.00	ecg: left posterior fascicular block	Cardiac arrythmia
329F.00	ecg: right bundle branch and left anterior fascicular block	Cardiac arrythmia
329G.00	ecg: right bundle branch and left posterior fascicular block	Cardiac arrythmia
8HTy.00	referral to atrial fibrillation clinic	Cardiac arrythmia
3297.11	electrocardiogram: mobitz type 1 second degree av block	Cardiac arrythmia
329H.00	electrocardiogram: mobitz type 2 second degree av block	Cardiac arrythmia
7936F00	renewal of intravenous cardiac pacemaker system	Cardiac arrythmia
G574100	ventricular flutter	Cardiac arrythmia

2JS..00	patient with internal cardiac defibrillator pacemaker	Cardiac arrythmia
7936400	removal of intravenous cardiac pacemaker system	Cardiac arrythmia
G572100	bouveret-hoffmann syndrome	Cardiac arrythmia
G572000	essential paroxysmal tachycardia	Cardiac arrythmia
G56y600	pacemaker twiddler's syndrome	Cardiac arrythmia
G576.11	premature beats	Cardiac arrythmia
662S.00	atrial fibrillation monitoring	Cardiac arrythmia
G56..11	conduction disorders of heart	Cardiac arrythmia
8CMW200	atrial fibrillation care pathway	Cardiac arrythmia
G56..00	conduction disorders	Cardiac arrythmia
8OAD.00	provision of written information about atrial fibrillation	Cardiac arrythmia
14AD.00	h/o ventricular fibrillation	Cardiac arrythmia
14V1.00	h/o: cardiac pacemaker in situ	Cardiac arrythmia
14V1.11	h/o: cardiac pacemaker	Cardiac arrythmia
3264.00	ecg: atrial ectopics	Cardiac arrythmia
327..00	ecg: supraventricular arrhythmia	Cardiac arrythmia
3272.00	ecg: atrial fibrillation	Cardiac arrythmia
3273.00	ecg: atrial flutter	Cardiac arrythmia
3274.00	ecg: paroxysmal atrial tachy.	Cardiac arrythmia
327Z.00	ecg: supraventric. arryth. nos	Cardiac arrythmia
328..00	ecg: ventricular arrhythmia	Cardiac arrythmia
3282.00	ecg: ventricular tachycardia	Cardiac arrythmia
3283.00	ecg: ventricular fibrillation	Cardiac arrythmia
328Z.00	ecg: ventricular arrhythmia nos	Cardiac arrythmia
329..00	ecg: heart block	Cardiac arrythmia
3297.00	ecg: wenckebach phenomenon	Cardiac arrythmia
3299.00	ecg: right bundle branch block	Cardiac arrythmia
329A.00	ecg: left bundle branch block	Cardiac arrythmia
329Z.00	ecg: heart block nos	Cardiac arrythmia
32D2.00	ecg: s wave abnormal	Cardiac arrythmia
32F2.00	ecg: t wave abnormal	Cardiac arrythmia
32I2.00	ecg: p-r interval abnormal	Cardiac arrythmia
32J2.00	ecg: qrs complex abnormal	Cardiac arrythmia
32K2.00	ecg: q-t interval abnormal	Cardiac arrythmia
G576200	ventricular ectopic beats	Cardiac arrythmia
G55A.11	tachycardia-induced cardiomyopathy	Cardiac arrythmia
7L1H700	external ventricular defibrillation	Cardiac arrythmia
G573600	paroxysmal atrial flutter	Cardiac arrythmia
14AR.00	history of atrial flutter	Cardiac arrythmia
7937900	implantation of biventricular cardiac pacemaker system	Cardiac arrythmia
7930000	open ablation of atrioventricular node	Cardiac arrythmia
7934000	percutaneous transluminal ablation of atrioventricular node	Cardiac arrythmia
7936.11	introduction of intravenous cardiac pacemaker system	Cardiac arrythmia
7936000	implantation of intravenous cardiac pacemaker system	Cardiac arrythmia
7936100	resiting of lead of intravenous pacemaker system	Cardiac arrythmia
7936300	maintenance of intravenous cardiac pacemaker system nec	Cardiac arrythmia

7936600	implantation of permanent intravenous cardiac pacemaker	Cardiac arrythmia
7936700	implantation of intravenous fixed-rate cardiac pacemaker	Cardiac arrythmia
7936800	implantation of intravenous triggered cardiac pacemaker	Cardiac arrythmia
7936900	implantation of intravenous atrial overdrive pacemaker	Cardiac arrythmia
7936y00	other specified cardiac pacemaker system introduced via vein	Cardiac arrythmia
7936z00	cardiac pacemaker system introduced via vein nos	Cardiac arrythmia
7937.00	other cardiac pacemaker system	Cardiac arrythmia
7937000	implantation of cardiac pacemaker system nec	Cardiac arrythmia
7937100	resiting of lead of cardiac pacemaker system nec	Cardiac arrythmia
7937200	maintenance of battery of cardiac pacemaker system nec	Cardiac arrythmia
7937300	maintenance of cardiac pacemaker system nec	Cardiac arrythmia
7937400	removal of cardiac pacemaker system nec	Cardiac arrythmia
7937y00	other specified other cardiac pacemaker system	Cardiac arrythmia
7937z00	other cardiac pacemaker system nos	Cardiac arrythmia
G567000	accelerated atrioventricular conduction	Cardiac arrythmia
9hF..00	exception reporting: atrial fibrillation quality indicators	Cardiac arrythmia
212R.00	atrial fibrillation resolved	Cardiac arrythmia
G561.00	partial atrioventricular block	Cardiac arrythmia
G561000	atrioventricular block unspecified	Cardiac arrythmia
G561400	second degree atrioventricular block	Cardiac arrythmia
G561z00	atrioventricular block nos	Cardiac arrythmia
G562z00	left bundle branch hemiblock nos	Cardiac arrythmia
G563.00	left main stem bundle branch block	Cardiac arrythmia
G565.00	other bundle branch block	Cardiac arrythmia
G565000	bundle branch block unspecified	Cardiac arrythmia
G565300	other bilateral bundle branch block	Cardiac arrythmia
G565z00	other bundle branch block nos	Cardiac arrythmia
G566.00	other heart block	Cardiac arrythmia
G566100	interventricular block nos	Cardiac arrythmia
G566z00	other heart block nos	Cardiac arrythmia
G567300	ventricular pre-excitation	Cardiac arrythmia
G567z00	anomalous atrioventricular excitation nos	Cardiac arrythmia
G56y.00	other conduction disorders	Cardiac arrythmia
G56yz00	other conduction disorders nos	Cardiac arrythmia
G56z.00	conduction disorders unspecified	Cardiac arrythmia
G56zz00	conduction disorders nos	Cardiac arrythmia
G570000	paroxysmal atrial tachycardia	Cardiac arrythmia
G570100	paroxysmal atrioventricular tachycardia	Cardiac arrythmia
G570200	paroxysmal junctional tachycardia	Cardiac arrythmia
G570300	paroxysmal nodal tachycardia	Cardiac arrythmia
G570z00	paroxysmal supraventricular tachycardia nos	Cardiac arrythmia
G572.00	paroxysmal tachycardia unspecified	Cardiac arrythmia
G572z00	paroxysmal tachycardia nos	Cardiac arrythmia
G573.00	atrial fibrillation and flutter	Cardiac arrythmia
G573z00	atrial fibrillation and flutter nos	Cardiac arrythmia
G574.00	ventricular fibrillation and flutter	Cardiac arrythmia

G574z00	ventricular fibrillation and flutter nos	Cardiac arrythmia
G57y400	sinoatrial node dysfunction nos	Cardiac arrythmia
G57y500	wandering atrial pacemaker	Cardiac arrythmia
G57y900	supraventricular tachycardia nos	Cardiac arrythmia
G57yA00	re-entry ventricular arrhythmia	Cardiac arrythmia
G57yz00	other cardiac dysrhythmia nos	Cardiac arrythmia
G57z.00	cardiac dysrhythmia nos	Cardiac arrythmia
Gyu5U00	[x]other and unspecified atrioventricular block	Cardiac arrythmia
Gyu5V00	[x]other and unspecified fascicular block	Cardiac arrythmia
Gyu5W00	[x]other and unspecified right bundle-branch block	Cardiac arrythmia
Gyu5X00	[x]other specified heart block	Cardiac arrythmia
Gyu5Y00	[x]other specified conduction disorders	Cardiac arrythmia
Gyu5a00	[x]other specified cardiac arrhythmias	Cardiac arrythmia
G567.00	anomalous atrioventricular excitation	Cardiac arrythmia
14AP.00	history of ventricular tachycardia	Cardiac arrythmia
14AQ.00	history of supraventricular tachycardia	Cardiac arrythmia
P6y5000	congenital heart block, unspecified	Cardiac arrythmia
SP00100	mechanical complication of cardiac pacemaker	Cardiac arrythmia
G573300	non-rheumatic atrial fibrillation	Cardiac arrythmia
G56..12	heart block	Cardiac arrythmia
7P19.00	pacemaker testing	Cardiac arrythmia
G576400	junctional premature depolarization	Cardiac arrythmia
G576500	ventricular premature depolarization	Cardiac arrythmia
7937700	implantation of single chamber cardiac pacemaker system	Cardiac arrythmia
7937800	implantation of dual chamber cardiac pacemaker system	Cardiac arrythmia
7P19y00	other specified pacemaker testing	Cardiac arrythmia
7P19z00	pacemaker testing nos	Cardiac arrythmia
7P19000	distant pacemaker test	Cardiac arrythmia
7936500	implantation of emergency intravenous cardiac pacemaker	Cardiac arrythmia
G57y.00	other cardiac dysrhythmias	Cardiac arrythmia
G561100	first degree atrioventricular block	Cardiac arrythmia
9Os..00	atrial fibrillation monitoring administration	Cardiac arrythmia
6A9..00	atrial fibrillation annual review	Cardiac arrythmia
9Os0.00	atrial fibrillation monitoring first letter	Cardiac arrythmia
9Os1.00	atrial fibrillation monitoring second letter	Cardiac arrythmia
9Os2.00	atrial fibrillation monitoring third letter	Cardiac arrythmia
9Os3.00	atrial fibrillation monitoring verbal invite	Cardiac arrythmia
9Os4.00	atrial fibrillation monitoring telephone invite	Cardiac arrythmia
G559.00	arrhythmogenic right ventricular cardiomyopathy	Cardiac arrythmia
G573200	paroxysmal atrial fibrillation	Cardiac arrythmia
G576300	atrial premature depolarization	Cardiac arrythmia
G571.11	ventricular tachycardia	Cardiac arrythmia
7936511	implantation of temporary intravenous cardiac pacemaker	Cardiac arrythmia
7936E00	implantation of intravenous dual chamber permanent pacemaker	Cardiac arrythmia
14AN.00	h/o: atrial fibrillation	Cardiac arrythmia
9N2b.00	seen by cardiac pacemaker technician	Cardiac arrythmia

ZV45000	[v]cardiac pacemaker in situ	Cardiac arrythmia
ZV53300	[v]fitting or adjustment of cardiac pacemaker	Cardiac arrythmia
G560.00	complete atrioventricular block	Cardiac arrythmia
G560.11	third degree atrioventricular block	Cardiac arrythmia
G561200	mobitz type ii atrioventricular block	Cardiac arrythmia
G567200	pre-excitation atrioventricular conduction	Cardiac arrythmia
G567100	accessory atrioventricular conduction	Cardiac arrythmia
ZV45M00	[v]biventricular pacemaker in situ	Cardiac arrythmia
G56y100	atrioventricular dissociation	Cardiac arrythmia
G574011	cardiac arrest-ventricular fibrillation	Cardiac arrythmia
P6y5100	congenital complete atrio-ventricular heart block	Cardiac arrythmia
P6y5200	congenital incomplete atrio-ventricular heart block	Cardiac arrythmia
7L1H.13	defibrillation	Cardiac arrythmia
G562000	left anterior fascicular block	Cardiac arrythmia
3298.00	ecg: complete a-v block	Cardiac arrythmia
32I3.00	ecg: p-r interval prolonged	Cardiac arrythmia
3295.00	ecg: partial a-v block - 2:1	Cardiac arrythmia
3296.00	ecg: partial a-v block - 3:1	Cardiac arrythmia
3292.00	ecg: partial sinu-atrial block	Cardiac arrythmia
32K3.00	ecg: q-t interval prolonged	Cardiac arrythmia
32K4.00	ecg: q-t interval shortened	Cardiac arrythmia
32J3.00	ecg: qrs complex prolonged	Cardiac arrythmia
3293.00	ecg:complete sinu-atrial block	Cardiac arrythmia
3294.00	ecg:partial a-v block-long p-r	Cardiac arrythmia
G573500	persistent atrial fibrillation	Cardiac arrythmia
G573400	permanent atrial fibrillation	Cardiac arrythmia
Q491.00	neonatal cardiac dysrhythmia	Cardiac arrythmia
G561300	mobitz type i (wenckebach) atrioventricular block	Cardiac arrythmia
7936200	maintenance of battery of intravenous cardiac pacemaker syst	Cardiac arrythmia
7936K00	implantation of intravenous cardiac pacemaker system nec	Cardiac arrythmia
P6y5.00	congenital heart block	Cardiac arrythmia
7936B00	implantation simple one wire intravenous cardiac pacemaker	Cardiac arrythmia
7936A00	implant intravenous pacemaker for atrial fibrillation	Cardiac arrythmia
TB01000	implant of cardiac pacemaker with complication, no blame	Cardiac arrythmia
7936D00	implantation complex two wire intravenous cardiac pacemaker	Cardiac arrythmia
7936C00	implantation of complex 1 wire intravenous cardiac pacemaker	Cardiac arrythmia
G573000	atrial fibrillation	Cardiac arrythmia
G56y000	lown-ganong-levine syndrome	Cardiac arrythmia
G562.00	left bundle branch hemiblock	Cardiac arrythmia
G564.00	right bundle branch block	Cardiac arrythmia
G573100	atrial flutter	Cardiac arrythmia
G6...00	cerebrovascular disease	Cerebrovascular disease
G650.00	basilar artery syndrome	Cerebrovascular disease
G656.00	vertebrobasilar insufficiency	Cerebrovascular disease
G634.00	carotid artery stenosis	Cerebrovascular disease
G679.00	small vessel cerebrovascular disease	Cerebrovascular disease

8CRB.00	transient ischaemic attack clinical management plan	Cerebrovascular disease
662M100	stroke 6 month review	Cerebrovascular disease
G640.00	cerebral thrombosis	Cerebrovascular disease
G64..00	cerebral arterial occlusion	Cerebrovascular disease
G64..13	stroke due to cerebral arterial occlusion	Cerebrovascular disease
G61..12	stroke due to intracerebral haemorrhage	Cerebrovascular disease
G66..12	stroke unspecified	Cerebrovascular disease
ZV12511	[v]personal history of stroke	Cerebrovascular disease
ZV12512	[v]personal history of cerebrovascular accident (cva)	Cerebrovascular disease
G70y011	carotid artery disease	Cerebrovascular disease
G60X.00	subarachnoid haemorrh from intracranial artery, unspecif	Cerebrovascular disease
G603.00	subarachnoid haemorrhage from anterior communicating artery	Cerebrovascular disease
G605.00	subarachnoid haemorrhage from basilar artery	Cerebrovascular disease
G602.00	subarachnoid haemorrhage from middle cerebral artery	Cerebrovascular disease
G604.00	subarachnoid haemorrhage from posterior communicating artery	Cerebrovascular disease
G641.00	cerebral embolism	Cerebrovascular disease
G63..12	stenosis of precerebral arteries	Cerebrovascular disease
G631.11	stenosis, carotid artery	Cerebrovascular disease
G64z111	lateral medullary syndrome	Cerebrovascular disease
9h2..00	exception reporting: stroke quality indicators	Cerebrovascular disease
9h21.00	excepted from stroke quality indicators: patient unsuitable	Cerebrovascular disease
9h22.00	excepted from stroke quality indicators: informed dissent	Cerebrovascular disease
G682.00	sequelae of other nontraumatic intracranial haemorrhage	Cerebrovascular disease
G68X.00	sequelae of stroke,not specfd as h'morrhage or infarction	Cerebrovascular disease
G68W.00	sequelae/other + unspecified cerebrovascular diseases	Cerebrovascular disease
G64z.12	cerebellar infarction	Cerebrovascular disease
G61X100	right sided intracerebral haemorrhage, unspecified	Cerebrovascular disease
662e.00	stroke/cva annual review	Cerebrovascular disease
38G3.00	hyperten, abnorm renal/liver funct, stroke, bled score	Cerebrovascular disease
8IEC.00	ref multidisciplinary stroke function improvement declined	Cerebrovascular disease
L440300	puerperal cerebrovascular disorder with antenatal comp	Cerebrovascular disease
661M700	stroke self-management plan agreed	Cerebrovascular disease
662e.11	stroke annual review	Cerebrovascular disease
5C10.00	carotid artery doppler abnormal	Cerebrovascular disease
G64..12	infarction - cerebral	Cerebrovascular disease
9Om..00	stroke/transient ischaemic attack monitoring administration	Cerebrovascular disease
8HBJ.00	stroke / transient ischaemic attack referral	Cerebrovascular disease
G64z400	infarction of basal ganglia	Cerebrovascular disease
13YA.00	stroke group member	Cerebrovascular disease
14A7.12	h/o: stroke	Cerebrovascular disease
9Om2.00	stroke/transient ischaemic attack monitoring third letter	Cerebrovascular disease
G623.00	subdural haemorrhage nos	Cerebrovascular disease
9Om1.00	stroke/transient ischaemic attack monitoring second letter	Cerebrovascular disease
9Om0.00	stroke/transient ischaemic attack monitoring first letter	Cerebrovascular disease
662o.00	haemorrhagic stroke monitoring	Cerebrovascular disease
662M.00	stroke monitoring	Cerebrovascular disease

7004300	evacuation of intracerebral haematoma nec	Cerebrovascular disease
G677400	occlusion+stenosis of multiple and bilat cerebral arteries	Cerebrovascular disease
G677.00	occlusion/stenosis cerebral arts not result cerebral infarct	Cerebrovascular disease
7A20311	carotid endarterectomy and patch	Cerebrovascular disease
7A20400	endarterectomy of carotid artery nec	Cerebrovascular disease
E030400	acute confusional state, of cerebrovascular origin	Cerebrovascular disease
E031400	subacute confusional state, of cerebrovascular origin	Cerebrovascular disease
F11x200	cerebral degeneration due to cerebrovascular disease	Cerebrovascular disease
G600.00	ruptured berry aneurysm	Cerebrovascular disease
G601.00	subarachnoid haemorrhage from carotid siphon and bifurcation	Cerebrovascular disease
G606.00	subarachnoid haemorrhage from vertebral artery	Cerebrovascular disease
G60z.00	subarachnoid haemorrhage nos	Cerebrovascular disease
G616.00	external capsule haemorrhage	Cerebrovascular disease
G617.00	intracerebral haemorrhage, intraventricular	Cerebrovascular disease
G61z.00	intracerebral haemorrhage nos	Cerebrovascular disease
G62..00	other and unspecified intracranial haemorrhage	Cerebrovascular disease
G621.00	subdural haemorrhage - nontraumatic	Cerebrovascular disease
G62z.00	intracranial haemorrhage nos	Cerebrovascular disease
G630.00	basilar artery occlusion	Cerebrovascular disease
G632.00	vertebral artery occlusion	Cerebrovascular disease
G633.00	multiple and bilateral precerebral arterial occlusion	Cerebrovascular disease
G63y.00	other precerebral artery occlusion	Cerebrovascular disease
G63y000	cerebral infarct due to thrombosis of precerebral arteries	Cerebrovascular disease
G63y100	cerebral infarction due to embolism of precerebral arteries	Cerebrovascular disease
G63z.00	precerebral artery occlusion nos	Cerebrovascular disease
G640000	cerebral infarction due to thrombosis of cerebral arteries	Cerebrovascular disease
G641000	cerebral infarction due to embolism of cerebral arteries	Cerebrovascular disease
G653.00	carotid artery syndrome hemispheric	Cerebrovascular disease
G654.00	multiple and bilateral precerebral artery syndromes	Cerebrovascular disease
G65y.00	other transient cerebral ischaemia	Cerebrovascular disease
G65zz00	transient cerebral ischaemia nos	Cerebrovascular disease
G65z100	intermittent cerebral ischaemia	Cerebrovascular disease
G660.00	middle cerebral artery syndrome	Cerebrovascular disease
G661.00	anterior cerebral artery syndrome	Cerebrovascular disease
G662.00	posterior cerebral artery syndrome	Cerebrovascular disease
G664.00	cerebellar stroke syndrome	Cerebrovascular disease
G667.00	left sided cva	Cerebrovascular disease
G668.00	right sided cva	Cerebrovascular disease
G67..00	other cerebrovascular disease	Cerebrovascular disease
G671z00	generalised ischaemic cerebrovascular disease nos	Cerebrovascular disease
G671000	acute cerebrovascular insufficiency nos	Cerebrovascular disease
G677000	occlusion and stenosis of middle cerebral artery	Cerebrovascular disease
G677100	occlusion and stenosis of anterior cerebral artery	Cerebrovascular disease
G677200	occlusion and stenosis of posterior cerebral artery	Cerebrovascular disease
G677300	occlusion and stenosis of cerebellar arteries	Cerebrovascular disease
G67y.00	other cerebrovascular disease os	Cerebrovascular disease

G67z.00	other cerebrovascular disease nos	Cerebrovascular disease
G68..00	late effects of cerebrovascular disease	Cerebrovascular disease
G680.00	sequelae of subarachnoid haemorrhage	Cerebrovascular disease
G681.00	sequelae of intracerebral haemorrhage	Cerebrovascular disease
G683.00	sequelae of cerebral infarction	Cerebrovascular disease
G6y..00	other specified cerebrovascular disease	Cerebrovascular disease
G6z..00	cerebrovascular disease nos	Cerebrovascular disease
Gyu6000	[x]subarachnoid haemorrhage from other intracranial arteries	Cerebrovascular disease
Gyu6100	[x]other subarachnoid haemorrhage	Cerebrovascular disease
Gyu6200	[x]other intracerebral haemorrhage	Cerebrovascular disease
Gyu6400	[x]other cerebral infarction	Cerebrovascular disease
Gyu6500	[x]occlusion and stenosis of other precerebral arteries	Cerebrovascular disease
Gyu6600	[x]occlusion and stenosis of other cerebral arteries	Cerebrovascular disease
Gyu6700	[x]other specified cerebrovascular diseases	Cerebrovascular disease
Gyu6F00	[x]intracerebral haemorrhage in hemisphere, unspecified	Cerebrovascular disease
L440000	puerperal cerebrovascular disorder unspecified	Cerebrovascular disease
L440100	puerperal cerebrovascular disorder - delivered	Cerebrovascular disease
P7y0.00	cerebrovascular system anomalies	Cerebrovascular disease
P7y0y00	other specified cerebrovascular anomaly	Cerebrovascular disease
P7y0z00	cerebrovascular system anomaly nos	Cerebrovascular disease
1JA1.00	suspected cerebrovascular disease	Cerebrovascular disease
S621.00	open traumatic subarachnoid haemorrhage	Cerebrovascular disease
S628.00	traumatic subdural haemorrhage	Cerebrovascular disease
S63z.00	other cerebral haemorrhage following injury nos	Cerebrovascular disease
F124.00	vascular parkinsonism	Cerebrovascular disease
G63..11	infarction - precerebral	Cerebrovascular disease
G64z.11	brainstem infarction nos	Cerebrovascular disease
G665.00	pure motor lacunar syndrome	Cerebrovascular disease
G666.00	pure sensory lacunar syndrome	Cerebrovascular disease
G619.00	lobar cerebral haemorrhage	Cerebrovascular disease
G657.00	carotid territory transient ischaemic attack	Cerebrovascular disease
Q200011	intracerebral haemorrhage in fetus or newborn	Cerebrovascular disease
Gyu6G00	[x]cereb infarct due unsp occlus/stenos precerebr arteries	Cerebrovascular disease
Gyu6300	[x]cerebrl infarctn due/unspcf occlusn or sten/cerebrl artrs	Cerebrovascular disease
Gyu6.00	[x]cerebrovascular diseases	Cerebrovascular disease
7P24200	delivery of rehabilitation for stroke	Cerebrovascular disease
S62..12	subarachnoid haemorrhage following injury	Cerebrovascular disease
S627.00	traumatic subarachnoid haemorrhage	Cerebrovascular disease
G63..00	precerebral arterial occlusion	Cerebrovascular disease
G631.00	carotid artery occlusion	Cerebrovascular disease
G64z.00	cerebral infarction nos	Cerebrovascular disease
G65..12	transient ischaemic attack	Cerebrovascular disease
G65..00	transient cerebral ischaemia	Cerebrovascular disease
S620.00	closed traumatic subarachnoid haemorrhage	Cerebrovascular disease
G66..00	stroke and cerebrovascular accident unspecified	Cerebrovascular disease
Gyu6A00	[x]other cerebrovascular disorders in diseases ce	Cerebrovascular disease

L440.12	stroke in the puerperium	Cerebrovascular disease
1M4..00	central post-stroke pain	Cerebrovascular disease
Fyu5700	[x]other vascular syndroms/brain in cerebrovasculr diseases	Cerebrovascular disease
Gyu6D00	[x]sequelae/other + unspecified cerebrovascular diseases	Cerebrovascular disease
Gyu6E00	[x]subarachnoid haemorrh from intracranial artery, unspecif	Cerebrovascular disease
8Hd6.00	admission to stroke unit	Cerebrovascular disease
G64z200	left sided cerebral infarction	Cerebrovascular disease
G64z300	right sided cerebral infarction	Cerebrovascular disease
1477.00	h/o: cerebrovascular disease	Cerebrovascular disease
14AK.00	h/o: stroke in last year	Cerebrovascular disease
F117.00	infantile posthaemorrhagic hydrocephalus	Cerebrovascular disease
G60..00	subarachnoid haemorrhage	Cerebrovascular disease
G615.00	bulbar haemorrhage	Cerebrovascular disease
G610.00	cortical haemorrhage	Cerebrovascular disease
G611.00	internal capsule haemorrhage	Cerebrovascular disease
G650.11	insufficiency - basilar artery	Cerebrovascular disease
G613.00	cerebellar haemorrhage	Cerebrovascular disease
G614.00	pontine haemorrhage	Cerebrovascular disease
G612.00	basal nucleus haemorrhage	Cerebrovascular disease
G663.00	brain stem stroke syndrome	Cerebrovascular disease
G64z000	brainstem infarction	Cerebrovascular disease
G676000	cereb infarct due cerebral venous thrombosis, nonpyogenic	Cerebrovascular disease
G6W..00	cereb infarct due unsp occlus/stenos precerebr arteries	Cerebrovascular disease
G641.11	cerebral embolus	Cerebrovascular disease
G6X..00	cerebrl infarctn due/unspcf occlusn or sten/cerebrl artrs	Cerebrovascular disease
L440.00	cerebrovascular disorders in the puerperium	Cerebrovascular disease
G64z100	wallenberg syndrome	Cerebrovascular disease
G651.00	vertebral artery syndrome	Cerebrovascular disease
G64..11	cva - cerebral artery occlusion	Cerebrovascular disease
G61..11	cva - cerebrovascular accid due to intracerebral haemorrhage	Cerebrovascular disease
L440.11	cva - cerebrovascular accident in the puerperium	Cerebrovascular disease
G66..13	cva - cerebrovascular accident unspecified	Cerebrovascular disease
G66..11	cva unspecified	Cerebrovascular disease
7A20300	endarterectomy and patch repair of carotid artery	Cerebrovascular disease
G651000	vertebro-basilar artery syndrome	Cerebrovascular disease
G65..13	vertebro-basilar insufficiency	Cerebrovascular disease
Q417000	intracerebral (nontraumatic) haemorrhage of fet and newborn	Cerebrovascular disease
G61..00	intracerebral haemorrhage	Cerebrovascular disease
G61X.00	intracerebral haemorrhage in hemisphere, unspecified	Cerebrovascular disease
G618.00	intracerebral haemorrhage, multiple localized	Cerebrovascular disease
G61X000	left sided intracerebral haemorrhage, unspecified	Cerebrovascular disease
G671.00	generalised ischaemic cerebrovascular disease nos	Cerebrovascular disease
14A7.00	h/o: cva/stroke	Cerebrovascular disease
4369B	stroke	Cerebrovascular disease
G65z.00	transient cerebral ischaemia nos	Cerebrovascular disease
8HHM.00	ref to multidisciplinary stroke function improvement service	Cerebrovascular disease

9Om3.00	stroke/transient ischaemic attack monitoring verbal invitati	Cerebrovascular disease
4309	subarachnoid haemorrhage	Cerebrovascular disease
4389	cerebrovascular disease	Cerebrovascular disease
1477	h/o: cerebrovascular disease	Cerebrovascular disease
C391211	thrombocytopenic eczema with immunodeficiency	Coagulopathy
D313200	thrombocytopenic purpura with absent radius	Coagulopathy
D306.12	disseminated intravascular coagulation	Coagulopathy
D306.00	defibrination syndrome	Coagulopathy
BBs4.00	[m]idiopathic thrombocythaemia	Coagulopathy
D311000	hereditary haemorrhagic thrombasthenia	Coagulopathy
42P2.00	thrombocytopenia	Coagulopathy
D314.00	secondary thrombocytopenia	Coagulopathy
42Qr.00	prothrombin consumption	Coagulopathy
G756100	thrombotic thrombocytopenic purpura	Coagulopathy
88A8.11	fibrinolysis	Coagulopathy
42P2.11	auto-immune thrombocytopenia	Coagulopathy
D314300	heparin-induced thrombocytopenia	Coagulopathy
B937411	primary thrombocythaemia	Coagulopathy
9b93100	haemophilia - specialty	Coagulopathy
D311.11	bernard - soulier thrombopathy	Coagulopathy
42P8.00	heparin induced thrombocytopenia screening test	Coagulopathy
42QB.11	plasma factor viii level	Coagulopathy
B937500	idiopathic thrombocythaemia	Coagulopathy
42P3.00	thrombocythaemia	Coagulopathy
D303.00	congenital deficiency of other clotting factors	Coagulopathy
D303z00	congenital deficiency of other clotting factor nos	Coagulopathy
D305.00	haemorrhagic disorder due to circulating anticoagulants	Coagulopathy
D305000	haemorrhagic disorder due to antithrombinaemia	Coagulopathy
D305100	haemorrhagic disorder due to hyperheparinaemia	Coagulopathy
D307000	deficiency of coagulation factor due to liver disease	Coagulopathy
D307100	deficiency of coagulation factor due to vitamin k deficiency	Coagulopathy
D307200	acquired factor ii deficiency	Coagulopathy
D307211	acquired prothrombin deficiency	Coagulopathy
D307y00	other specified acquired coagulation factor deficiency	Coagulopathy
D307z00	acquired coagulation factor deficiency nos	Coagulopathy
D31..00	purpura and other haemorrhagic conditions	Coagulopathy
D311z00	qualitative platelet deficiency nos	Coagulopathy
D313300	[x]essential thrombocytopenia nos	Coagulopathy
D313y00	other specified primary thrombocytopenia	Coagulopathy
D314100	thrombocytopenia due to drugs	Coagulopathy
D314200	thrombocytopenia due to extracorporeal circulation of blood	Coagulopathy
D314y00	other specified secondary thrombocytopenia	Coagulopathy
D315.00	thrombocytopenia nos	Coagulopathy
D31yz00	other specified haemorrhagic condition nos	Coagulopathy
D31z.00	haemorrhagic condition nos	Coagulopathy
Dyu3000	[x]other specified coagulation defects	Coagulopathy

Dyu3200	[x]other primary thrombocytopenia	Coagulopathy
Dyu3300	[x]other specified haemorrhagic conditions	Coagulopathy
Dyu3400	[x]haemorrhagic condition, unspecified	Coagulopathy
L363z00	postpartum coagulation defects nos	Coagulopathy
Q451z00	transient neonatal thrombocytopenia nos	Coagulopathy
D313z11	essential thrombocytopenia nos	Coagulopathy
D313111	hereditary thrombocytopenia nec	Coagulopathy
Q451y11	neonatal thrombocytopenia due to platelet alloimmunisation	Coagulopathy
L363.00	postpartum coagulation defects	Coagulopathy
D311.00	qualitative platelet defects	Coagulopathy
D313.00	primary thrombocytopenia	Coagulopathy
D313100	congenital thrombocytopenic purpura	Coagulopathy
D313z00	primary thrombocytopenia nos	Coagulopathy
D314z00	secondary thrombocytopenia nos	Coagulopathy
42QB.00	factor viii assay	Coagulopathy
D307.00	acquired coagulation factor deficiency	Coagulopathy
D313.15	thrombocytopenic purpura	Coagulopathy
D307212	acquired hypoprothrombinaemia	Coagulopathy
42Ql.00	factor viii von willebrands factor ratio	Coagulopathy
D306.11	afibrinogenaemia - acquired	Coagulopathy
D304.00	von willebrand's disease	Coagulopathy
D303611	fibrin stabilising factor deficiency	Coagulopathy
D300.12	haemophilia a	Coagulopathy
D303000	congenital afibrinogenaemia	Coagulopathy
D300.11	antihaemophilic globulin deficiency	Coagulopathy
N032000	arthropathy due to haemophilia	Coagulopathy
D301.12	haemophilia b	Coagulopathy
D302.11	haemophilia c	Coagulopathy
D313000	idiopathic thrombocytopenic purpura	Coagulopathy
D313011	idiopathic purpura	Coagulopathy
D30..00	coagulation defects	Coagulopathy
D303y00	congenital deficiency of other clotting factor os	Coagulopathy
D303100	deficiency of factor ii or prothrombin	Coagulopathy
D303200	deficiency of factor v or labile factor	Coagulopathy
D303300	deficiency of factor vii or stable factor	Coagulopathy
D303400	deficiency of factor x or stuart-prower factor	Coagulopathy
D303500	deficiency of factor xii or hageman factor	Coagulopathy
D303600	deficiency of factor xiii or fibrin stabilizing factor	Coagulopathy
B937400	essential (haemorrhagic) thrombocythaemia	Coagulopathy
D313.11	evan's syndrome	Coagulopathy
42Qj.00	factor viii inhibitor activity	Coagulopathy
D301.11	christmas disease	Coagulopathy
D313012	itp - idiopathic thrombocytopenic purpura	Coagulopathy
B937.12	idiopathic thrombocythaemia	Coagulopathy
D313.12	idiopathic thrombocytopenic purpura	Coagulopathy
D303111	hypoprothrombinaemia	Coagulopathy

D300.00	haemophilia-a (factor viii deficiency)	Coagulopathy
D301.00	haemophilia-b (factor ix deficiency)	Coagulopathy
D302.00	haemophilia-c (factor xi deficiency)	Coagulopathy
D302.12	rosenthal's disease	Coagulopathy
C290.00	vitamin k deficiency	Coagulopathy
G58..00	heart failure	Congestive heart failure
G58z.11	weak heart	Congestive heart failure
G58..11	cardiac failure	Congestive heart failure
G581.00	left ventricular failure	Congestive heart failure
G580100	chronic congestive heart failure	Congestive heart failure
8HBE.00	heart failure follow-up	Congestive heart failure
662W.00	heart failure annual review	Congestive heart failure
1O1..00	heart failure confirmed	Congestive heart failure
G5yyA00	left ventricular diastolic dysfunction	Congestive heart failure
G581.12	pulmonary oedema - acute	Congestive heart failure
G581.11	asthma - cardiac	Congestive heart failure
9hH0.00	excepted heart failure quality indicators: patient unsuitabl	Congestive heart failure
9hH1.00	excepted heart failure quality indicators: informed dissent	Congestive heart failure
G583.00	heart failure with normal ejection fraction	Congestive heart failure
G583.11	hfnef - heart failure with normal ejection fraction	Congestive heart failure
8IE0.00	referral to heart failure education group declined	Congestive heart failure
8HgD.00	discharge from heart failure nurse service	Congestive heart failure
8IE1.00	referral to heart failure exercise programme declined	Congestive heart failure
8CMK.00	has heart failure management plan	Congestive heart failure
G554000	congestive cardiomyopathy	Congestive heart failure
8CeC.00	preferred place of care for next exacerbation heart failure	Congestive heart failure
G584.00	right ventricular failure	Congestive heart failure
G580000	acute congestive heart failure	Congestive heart failure
G580.12	right heart failure	Congestive heart failure
661M500	heart failure self-management plan agreed	Congestive heart failure
679W100	education about deteriorating heart failure	Congestive heart failure
585f.00	echocardiogram shows left ventricular systolic dysfunction	Congestive heart failure
585g.00	echocardiogram shows left ventricular diastolic dysfunction	Congestive heart failure
662T.00	congestive heart failure monitoring	Congestive heart failure
G5yy900	left ventricular systolic dysfunction	Congestive heart failure
8HTL.00	referral to heart failure clinic	Congestive heart failure
8CMW800	heart failure clinical pathway	Congestive heart failure
G583.12	heart failure with preserved ejection fraction	Congestive heart failure
G58z.12	cardiac failure nos	Congestive heart failure
8HTL000	referral to rapid access heart failure clinic	Congestive heart failure
14A6.00	h/o: heart failure	Congestive heart failure
585k.00	echocardiogram shows normal left ventricular function	Congestive heart failure
9N2p.00	seen by community heart failure nurse	Congestive heart failure
G5yyB00	right ventricular diastolic dysfunction	Congestive heart failure
8H2S.00	admit heart failure emergency	Congestive heart failure
9N0k.00	seen in heart failure clinic	Congestive heart failure

8HHb.00	referral to heart failure nurse	Congestive heart failure
662f.00	new york heart association classification - class i	Congestive heart failure
662g.00	new york heart association classification - class ii	Congestive heart failure
662h.00	new york heart association classification - class iii	Congestive heart failure
662i.00	new york heart association classification - class iv	Congestive heart failure
679X.00	heart failure education	Congestive heart failure
G580400	congestive heart failure due to valvular disease	Congestive heart failure
G580200	decompensated cardiac failure	Congestive heart failure
G580300	compensated cardiac failure	Congestive heart failure
G581000	acute left ventricular failure	Congestive heart failure
G5y4z00	post cardiac operation heart failure nos	Congestive heart failure
9N6T.00	referred by heart failure nurse specialist	Congestive heart failure
8Hg8.00	discharge from practice nurse heart failure clinic	Congestive heart failure
9N4s.00	did not attend practice nurse heart failure clinic	Congestive heart failure
67D4.00	heart failure information given to patient	Congestive heart failure
8HHz.00	referral to heart failure exercise programme	Congestive heart failure
9Or..00	heart failure monitoring administration	Congestive heart failure
9Or0.00	heart failure review completed	Congestive heart failure
8CL3.00	heart failure care plan discussed with patient	Congestive heart failure
8Hk0.00	referred to heart failure education group	Congestive heart failure
Q48y100	congenital cardiac failure	Congestive heart failure
R2y1000	[d]cardiorespiratory failure	Congestive heart failure
SP11200	cardiorespiratory failure as a complication of care	Congestive heart failure
SP11111	heart failure as a complication of care	Congestive heart failure
G58z.00	heart failure nos	Congestive heart failure
J615700	cardiac portal cirrhosis	Congestive heart failure
SP11100	cardiac insufficiency as a complication of care	Congestive heart failure
662p.00	heart failure 6 month review	Congestive heart failure
9Or1.00	heart failure monitoring telephone invite	Congestive heart failure
9Or2.00	heart failure monitoring verbal invite	Congestive heart failure
9Or3.00	heart failure monitoring first letter	Congestive heart failure
9Or4.00	heart failure monitoring second letter	Congestive heart failure
9Or5.00	heart failure monitoring third letter	Congestive heart failure
9hH..00	exception reporting: heart failure quality indicators	Congestive heart failure
G581.13	impaired left ventricular function	Congestive heart failure
Q490.00	neonatal cardiac failure	Congestive heart failure
8B29.00	cardiac failure therapy	Congestive heart failure
14AM.00	h/o: heart failure in last year	Congestive heart failure
G580.13	right ventricular failure	Congestive heart failure
G580.11	congestive cardiac failure	Congestive heart failure
G211100	benign hypertensive heart disease with ccf	Congestive heart failure
G580.14	biventricular failure	Congestive heart failure
G5yyC00	diastolic dysfunction	Congestive heart failure
G580.00	congestive heart failure	Congestive heart failure
G210100	malignant hypertensive heart disease with ccf	Congestive heart failure
G1yz100	rheumatic left ventricular failure	Congestive heart failure

9m5..00	high risk of heart failure screening invitation	Congestive heart failure
G21z100	hypertensive heart disease nos with ccf	Congestive heart failure
G232.00	hypertensive heart&renal dis wth (congestive) heart failure	Congestive heart failure
G234.00	hyperten heart&renal dis+both(congestv)heart and renal fail	Congestive heart failure
388D.00	new york heart assoc classification heart failure symptoms	Congestive heart failure
G582.00	acute heart failure	Congestive heart failure
7824BW	weak heart	Congestive heart failure
7824FH	heart failure	Congestive heart failure
ZRad.00	new york heart assoc classification heart failure symptoms	Congestive heart failure
4270C	congestive cardiac failure	Congestive heart failure
4270	congestive heart failure	Congestive heart failure
425 CC	congestive cardiomyopathy	Congestive heart failure
H31..00	chronic bronchitis	COPD
H322.00	centrilobular emphysema	COPD
38Dd.00	clinical chronic obstructive pulmonary disease questionnaire	COPD
38Dg.00	chronic obstructive pulmonary disease assessment test	COPD
H3y..11	other specified chronic obstructive pulmonary disease	COPD
H3z..11	chronic obstructive pulmonary disease nos	COPD
H32y200	macleod's unilateral emphysema	COPD
H311.00	mucopurulent chronic bronchitis	COPD
9h5..00	exception reporting: copd quality indicators	COPD
9h51.00	excepted from copd quality indicators: patient unsuitable	COPD
9h52.00	excepted from copd quality indicators: informed dissent	COPD
H311100	fetid chronic bronchitis	COPD
66YB.00	chronic obstructive pulmonary disease monitoring	COPD
66YD.00	chronic obstructive pulmonary disease monitoring due	COPD
66YL.00	chronic obstructive pulmonary disease follow-up	COPD
66YM.00	chronic obstructive pulmonary disease annual review	COPD
8CE6.00	chronic obstructive pulmonary disease leaflet given	COPD
8BMW.00	issue of chronic obstructive pulmonary disease rescue pack	COPD
66YB000	chronic obstructive pulmonary disease 3 monthly review	COPD
66YB100	chronic obstructive pulmonary disease 6 monthly review	COPD
9kf..00	copd - enhanced services administration	COPD
9kf0.00	copd patient unsuitable for pulmonary rehab - enh serv admin	COPD
9kf0.11	copd patient unsuitable for pulmonary rehabilitation	COPD
9kf1.11	referred for copd structured smoking assessment	COPD
9kf1.00	refer copd structured smoking assessment - enhanc serv admin	COPD
9kf2.00	copd structured smoking assessment declined - enh serv admin	COPD
9kf2.11	copd structured smoking assessment declined	COPD
14B3.12	history of chronic obstructive pulmonary disease	COPD
66YS.00	chronic obstructive pulmonary disease monitoring by nurse	COPD
66YT.00	chronic obstructive pulmonary disease monitoring by doctor	COPD
8BMa000	chronic obstructiv pulmonary disease medication optimisation	COPD
8CeD.00	preferred place of care for next exacerbation of copd	COPD
8Hkw.00	referral to copd community nursing team	COPD
9e03.00	gp ooh service notified of copd care plan	COPD

661M300	copd self-management plan agreed	COPD
661N300	copd self-management plan review	COPD
9NgP.00	on chronic obstructive pulmonary disease supprtv cre pathway	COPD
9NgP.11	on copd (chr obstruc pulmonary disease) supportv cre pathway	COPD
H30..12	recurrent wheezy bronchitis	COPD
8IEy.00	chronic obstructive pulmon dis wr self managem plan declined	COPD
9Oi0.00	chronic obstructive pulmonary disease monitoring 1st letter	COPD
H581.00	interstitial emphysema	COPD
9Oi1.00	chronic obstructive pulmonary disease monitoring 2nd letter	COPD
9Oi2.00	chronic obstructive pulmonary disease monitoring 3rd letter	COPD
8CMV.00	has chronic obstructive pulmonary disease care plan	COPD
8H2R.00	admit copd emergency	COPD
H3A..00	end stage chronic obstructive airways disease	COPD
8CMW500	chronic obstructive pulmonary disease care pathway	COPD
9Nk7000	seen in chronic obstructive pulmonary disease clinic	COPD
8IEZ.00	chronic obstructive pulmonary disease rescue pack declined	COPD
H320200	giant bullous emphysema	COPD
H312100	emphysematous bronchitis	COPD
H312.00	obstructive chronic bronchitis	COPD
66Yg.00	chronic obstructive pulmonary disease disturbs sleep	COPD
66Yh.00	chronic obstructive pulmonary disease does not disturb sleep	COPD
H310z00	simple chronic bronchitis nos	COPD
H311z00	mucopurulent chronic bronchitis nos	COPD
H312011	chronic wheezy bronchitis	COPD
H312200	acute exacerbation of chronic obstructive airways disease	COPD
H312z00	obstructive chronic bronchitis nos	COPD
H313.00	mixed simple and mucopurulent chronic bronchitis	COPD
H31y.00	other chronic bronchitis	COPD
H31yz00	other chronic bronchitis nos	COPD
H31z.00	chronic bronchitis nos	COPD
H320.00	chronic bullous emphysema	COPD
H320000	segmental bullous emphysema	COPD
H320100	zonal bullous emphysema	COPD
H320z00	chronic bullous emphysema nos	COPD
H32y.00	other emphysema	COPD
H32y000	acute vesicular emphysema	COPD
H32z.00	emphysema nos	COPD
H3y..00	other specified chronic obstructive airways disease	COPD
H3z..00	chronic obstructive airways disease nos	COPD
H464000	chronic emphysema due to chemical fumes	COPD
Hyu3000	[x]other emphysema	COPD
Hyu3100	[x]other specified chronic obstructive pulmonary disease	COPD
H320300	bullous emphysema with collapse	COPD
H32y100	atrophic (senile) emphysema	COPD
H32yz00	other emphysema nos	COPD
679V.00	health education - chronic obstructive pulmonary disease	COPD

H36..00	mild chronic obstructive pulmonary disease	COPD
H37..00	moderate chronic obstructive pulmonary disease	COPD
H38..00	severe chronic obstructive pulmonary disease	COPD
H32y111	acute interstitial emphysema	COPD
H3...00	chronic obstructive pulmonary disease	COPD
H311000	purulent chronic bronchitis	COPD
H310.00	simple chronic bronchitis	COPD
H310000	chronic catarrhal bronchitis	COPD
H39..00	very severe chronic obstructive pulmonary disease	COPD
H3y1.00	chron obstruct pulmonary dis wth acute exacerbation, unspec	COPD
H3y0.00	chronic obstruct pulmonary dis with acute lower resp infectn	COPD
H3...11	chronic obstructive airways disease	COPD
H582.00	compensatory emphysema	COPD
H32..00	emphysema	COPD
SK07.00	subcutaneous emphysema	COPD
66YI.00	copd self-management plan given	COPD
66YL.11	copd follow-up	COPD
H31y100	chronic tracheobronchitis	COPD
H321.00	panlobular emphysema	COPD
8CR1.00	chronic obstructive pulmonary disease clini management plan	COPD
9Oi..00	chronic obstructive pulmonary disease monitoring admin	COPD
9Oi3.00	chronic obstructive pulmonary disease monitoring verb invite	COPD
9Oi4.00	chronic obstructive pulmonary disease monitor phone invite	COPD
66Yd.00	copd accident and emergency attendance since last visit	COPD
66Ye.00	emergency copd admission since last appointment	COPD
66Yf.00	number of copd exacerbations in past year	COPD
66Yi.00	multiple copd emergency hospital admissions	COPD
H3y1.00	\chron obstruct pulmonary dis wth acute exacerbation, unspec\""	COPD
D00y012	plummer-vinson syndrome	Deficiency anaemia
D00y000	sideropenic dysphagia	Deficiency anaemia
D00y.12	plummer - vinson syndrome	Deficiency anaemia
D010.11	addison's anaemia	Deficiency anaemia
D011011	imerslund - grasbeck syndrome	Deficiency anaemia
1452.00	h/o: anaemia vit.b12 deficient	Deficiency anaemia
66E6.00	reticulcytosis after b12	Deficiency anaemia
D001.00	iron deficiency anaemia due to dietary causes	Deficiency anaemia
D00yz00	other specified iron deficiency anaemia nos	Deficiency anaemia
D00z.00	unspecified iron deficiency anaemia	Deficiency anaemia
D00z100	chlorotic anaemia	Deficiency anaemia
D00z200	idiopathic hypochromic anaemia	Deficiency anaemia
D00zz00	iron deficiency anaemia nos	Deficiency anaemia
D011z00	other vitamin b12 deficiency anaemia nos	Deficiency anaemia
D013.00	other specified megaloblastic anaemia nec	Deficiency anaemia
D013000	combined b12 and folate deficiency anaemia	Deficiency anaemia
D013z00	other specified megaloblastic anaemia nec nos	Deficiency anaemia
D014.00	protein-deficiency anaemia	Deficiency anaemia

D014z00	protein-deficiency anaemia nos	Deficiency anaemia
D01y000	vitamin c deficiency anaemia	Deficiency anaemia
D01y100	vitamin e deficiency anaemia	Deficiency anaemia
D01yz00	other specified nutritional deficiency anaemia nos	Deficiency anaemia
D01z000	[x]megaloblastic anaemia nos	Deficiency anaemia
D0y..00	other specified deficiency anaemias	Deficiency anaemia
D0z..00	deficiency anaemias nos	Deficiency anaemia
Dyu0000	[x]other iron deficiency anaemias	Deficiency anaemia
Dyu0100	[x]other dietary vitamin b12 deficiency anaemia	Deficiency anaemia
Dyu0200	[x]other vitamin b12 deficiency anaemias	Deficiency anaemia
Dyu0300	[x]other folate deficiency anaemias	Deficiency anaemia
Dyu0600	[x]vitamin b12 deficiency anaemia, unspecified	Deficiency anaemia
F381500	myasthenic syndrome due to pernicious anaemia	Deficiency anaemia
L182500	iron deficiency anaemia of pregnancy	Deficiency anaemia
D01z.11	megaloblastic anaemia nos	Deficiency anaemia
D012112	megaloblastic anaemia due to dietary causes	Deficiency anaemia
D011013	vegan's anaemia	Deficiency anaemia
D010.12	biermer's congenital pernicious anaemia	Deficiency anaemia
D0...00	deficiency anaemias	Deficiency anaemia
D00y.00	other specified iron deficiency anaemia	Deficiency anaemia
D01..00	other deficiency anaemias	Deficiency anaemia
D011.00	other vitamin b12 deficiency anaemias	Deficiency anaemia
D011000	vitamin b12 deficiency anaemia due to dietary causes	Deficiency anaemia
D01z.00	other deficiency anaemias nos	Deficiency anaemia
U604100	[x]vit b12/folic/oth ant-megalobl-anaem caus adv ef ther use	Deficiency anaemia
D014000	amino-acid deficiency anaemia	Deficiency anaemia
D01..11	megaloblastic anaemia	Deficiency anaemia
D00z000	achlorhydric anaemia	Deficiency anaemia
D010.00	pernicious anaemia	Deficiency anaemia
D012.11	folic acid deficiency anaemia	Deficiency anaemia
D0...12	sideropenic anaemia	Deficiency anaemia
C294300	iron deficiency	Deficiency anaemia
D011100	vit b12 defic anaemia due to malabsorption with proteinuria	Deficiency anaemia
D011.11	vitamin b12 deficiency anaemia	Deficiency anaemia
D011X00	vitamin b12 deficiency anaemia, unspecified	Deficiency anaemia
D00..12	microcytic - hypochromic anaemia	Deficiency anaemia
D00..00	iron deficiency anaemias	Deficiency anaemia
D012.00	folate-deficiency anaemia	Deficiency anaemia
D012100	folate-deficiency anaemia due to dietary causes	Deficiency anaemia
D012400	folate-deficiency anaemia due to liver disorders	Deficiency anaemia
D012300	folate-deficiency anaemia due to malabsorption	Deficiency anaemia
D012z00	folate-deficiency anaemia nos	Deficiency anaemia
D012200	folate-deficiency anaemia, drug induced	Deficiency anaemia
D012111	goat's milk anaemia	Deficiency anaemia
8HgC.00	discharged from diabetes shared care programme	Diabetes
42W..00	test request : haemoglobin a1c	Diabetes

66AQ000	unsuitable for diabetes year of care programme	Diabetes
66AQ100	declined consent for diabetes year of care programme	Diabetes
679L000	education in self management of diabetes	Diabetes
66As.00	diabetic on subcutaneous treatment	Diabetes
C10..00	diabetes mellitus	Diabetes
C10FJ11	insulin treated type ii diabetes mellitus	Diabetes
C109J11	insulin treated non-insulin dependent diabetes mellitus	Diabetes
ZV65312	[v]dietary counselling in diabetes mellitus	Diabetes
F171100	autonomic neuropathy due to diabetes	Diabetes
C10G.00	secondary pancreatic diabetes mellitus	Diabetes
C11y000	steroid induced diabetes	Diabetes
C10B000	steroid induced diabetes mellitus without complication	Diabetes
C108y00	other specified diabetes mellitus with multiple comps	Diabetes
C106y00	other specified diabetes mellitus with neurological comps	Diabetes
C105y00	other specified diabetes mellitus with ophthalmic complicatn	Diabetes
C10yy00	other specified diabetes mellitus with other spec comps	Diabetes
C10zy00	other specified diabetes mellitus with unspecified comps	Diabetes
9h4..00	exception reporting: diabetes quality indicators	Diabetes
9h42.00	excepted from diabetes quality indicators: informed dissent	Diabetes
F420600	non proliferative diabetic retinopathy	Diabetes
9N2d.00	seen by diabetologist	Diabetes
2BBJ.00	o/e - no right diabetic retinopathy	Diabetes
2BBK.00	o/e - no left diabetic retinopathy	Diabetes
2BBL.00	o/e - diabetic maculopathy present both eyes	Diabetes
2BBM.00	o/e - diabetic maculopathy absent both eyes	Diabetes
8H7r.00	refer to diabetic foot screener	Diabetes
9N4I.00	dna - did not attend diabetic clinic	Diabetes
2G5E.00	o/e - right diabetic foot at low risk	Diabetes
2G5G.00	o/e - right diabetic foot at high risk	Diabetes
2G5H.00	o/e - right diabetic foot - ulcerated	Diabetes
2G5L.00	o/e - left diabetic foot - ulcerated	Diabetes
2G5I.00	o/e - left diabetic foot at low risk	Diabetes
2G5K.00	o/e - left diabetic foot at high risk	Diabetes
2G5J.00	o/e - left diabetic foot at moderate risk	Diabetes
2G5F.00	o/e - right diabetic foot at moderate risk	Diabetes
66AW.00	diabetic foot risk assessment	Diabetes
6761.00	diabetic pre-pregnancy counselling	Diabetes
8B3l.00	diabetes medication review	Diabetes
C10FK00	hyperosmolar non-ketotic state in type 2 diabetes mellitus	Diabetes
C10N.00	secondary diabetes mellitus	Diabetes
9OLZ.00	diabetes monitoring admin.nos	Diabetes
8I83.00	did not complete desmond diabetes structured educat program	Diabetes
8I84.00	did not complete xpert diabetes structured education program	Diabetes
9NiD.00	did not attend desmond diabetes structured education program	Diabetes
66At.00	diabetic dietary review	Diabetes
66At000	type i diabetic dietary review	Diabetes

66At100	type ii diabetic dietary review	Diabetes
8IAs.00	diabetic dietary review declined	Diabetes
66At111	type 2 diabetic dietary review	Diabetes
66At011	type 1 diabetic dietary review	Diabetes
2G5d.00	o/e - left diabetic foot at increased risk	Diabetes
2G5e.00	o/e - right diabetic foot at increased risk	Diabetes
C10FK11	hyperosmolar non-ketotic state in type ii diabetes mellitus	Diabetes
C10EQ11	type i diabetes mellitus with gastroparesis	Diabetes
9NiZ.00	did not attend diabetes foot screening	Diabetes
2BBF.00	retinal abnormality - diabetes related	Diabetes
9m0..00	diabetic retinopathy screening administrative status	Diabetes
9m02.00	eligibility temporarily inactive for diabetic retinop screen	Diabetes
9m03.00	eligibility permanently inactive for diabetic retinop screen	Diabetes
9m07.00	excluded diabetc retinop screen as under care ophthalmolgist	Diabetes
9m0C.00	excluded frm diabetic retinopathy screen as terminal illness	Diabetes
9m0D.00	excluded from diabetic retinopthy screen as learn disability	Diabetes
9m0E.00	excluded from diabetic retinopathy screen physical disorder	Diabetes
66Au.00	diabetic erectile dysfunction review	Diabetes
66Av.00	diabetic assessment of erectile dysfunction	Diabetes
1IA..00	no evidence of diabetic nephropathy	Diabetes
9h43.00	excepted from diabetes qual indicators: service unavailable	Diabetes
9OLN.00	diabetes monitor invitation by sms (short message service)	Diabetes
8IE2.00	diabetes care plan declined	Diabetes
67IJ100	pre-conception advice for diabetes mellitus	Diabetes
66Ac.00	diabetic peripheral neuropathy screening	Diabetes
C10EL00	type 1 diabetes mellitus with persistent microalbuminuria	Diabetes
66Ab.00	diabetic foot examination	Diabetes
F420700	high risk proliferative diabetic retinopathy	Diabetes
F420000	background diabetic retinopathy	Diabetes
C350011	bronzed diabetes	Diabetes
8IEa.00	referral to dafne diabetes structured educn prog declined	Diabetes
66o..00	further diabetic monitoring	Diabetes
C10N000	secondary diabetes mellitus without complication	Diabetes
9M00.00	informed consent for diabetes national audit	Diabetes
9M10.00	informed dissent for diabetes national audit	Diabetes
C10F.00	type 2 diabetes mellitus	Diabetes
C10E.00	type 1 diabetes mellitus	Diabetes
C10G000	secondary pancreatic diabetes mellitus without complication	Diabetes
66AJ100	brittle diabetes	Diabetes
8CA4100	pt advised re diabetic diet	Diabetes
C104.11	diabetic nephropathy	Diabetes
661N400	diabetes self-management plan review	Diabetes
8Hlc.00	referral to community diabetes service	Diabetes
661M400	diabetes self-management plan agreed	Diabetes
L180X00	pre-existing diabetes mellitus, unspecified	Diabetes
F372.00	polyneuropathy in diabetes	Diabetes

67D8.00	provision of diabetes clinical summary	Diabetes
8BL2.00	patient on maximal tolerated therapy for diabetes	Diabetes
9N1i.00	seen in diabetic foot clinic	Diabetes
918T.00	diabetes key contact	Diabetes
8HBG.00	diabetic retinopathy 12 month review	Diabetes
8HBH.00	diabetic retinopathy 6 month review	Diabetes
9NN8.00	under care of diabetologist	Diabetes
9NN9.00	under care of diabetes specialist nurse	Diabetes
9NND.00	under care of diabetic foot screener	Diabetes
8I6F.00	diabetic retinopathy screening not indicated	Diabetes
8I6G.00	diabetic foot examination not indicated	Diabetes
9360.00	patient held diabetic record issued	Diabetes
2BBP.00	o/e - right eye background diabetic retinopathy	Diabetes
2BBQ.00	o/e - left eye background diabetic retinopathy	Diabetes
2BBR.00	o/e - right eye preproliferative diabetic retinopathy	Diabetes
2BBS.00	o/e - left eye preproliferative diabetic retinopathy	Diabetes
2BBT.00	o/e - right eye proliferative diabetic retinopathy	Diabetes
2BBV.00	o/e - left eye proliferative diabetic retinopathy	Diabetes
2BBW.00	o/e - right eye diabetic maculopathy	Diabetes
2BBX.00	o/e - left eye diabetic maculopathy	Diabetes
9b92000	diabetic medicine	Diabetes
C10H.00	diabetes mellitus induced by non-steroid drugs	Diabetes
66AU.00	diabetes care by hospital only	Diabetes
68A7.00	diabetic retinopathy screening	Diabetes
9OL..00	diabetes monitoring admin.	Diabetes
8IEQ.00	referral to community diabetes specialist nurse declined	Diabetes
8HTE100	referral to community diabetes clinic	Diabetes
8CMW700	diabetes clinical pathway	Diabetes
8OA3.00	provision of written information about diabetes and driving	Diabetes
9Oy0000	diabetic foot screening invitation	Diabetes
9Oy0200	diabetic foot screening invitation first letter	Diabetes
9Oy0300	diabetic foot screening invitation second letter	Diabetes
9Oy0400	diabetic foot screening invitation third letter	Diabetes
C100111	maturity onset diabetes	Diabetes
K27y700	erectile dysfunction due to diabetes mellitus	Diabetes
9NJy.00	in-house diabetic foot screening	Diabetes
66AH200	conversion to insulin by diabetes specialist nurse	Diabetes
679L200	education about diabetes and driving	Diabetes
679L211	advice about diabetes and driving	Diabetes
8I94.00	diabetes structured education programme not available	Diabetes
66AS000	diabetes year of care annual review	Diabetes
C10P.00	diabetes mellitus in remission	Diabetes
C10P000	type i diabetes mellitus in remission	Diabetes
C10P011	type 1 diabetes mellitus in remission	Diabetes
C10P100	type ii diabetes mellitus in remission	Diabetes
8I3W.00	diabetic foot examination declined	Diabetes

8CR2.00	diabetes clinical management plan	Diabetes
8I3X.00	diabetic retinopathy screening refused	Diabetes
7276.00	pan retinal photocoagulation for diabetes	Diabetes
C10C.12	maturity onset diabetes in youth type 1	Diabetes
13B1.00	diabetic diet	Diabetes
13Y1.00	diabetic association member	Diabetes
1434	h/o: diabetes mellitus	Diabetes
9OL..11	diabetes clinic administration	Diabetes
8CP2.00	transition of diabetes care options discussed	Diabetes
2G5W.00	o/e - left chronic diabetic foot ulcer	Diabetes
2G5V.00	o/e - right chronic diabetic foot ulcer	Diabetes
9OLB.00	attended diabetes structured education programme	Diabetes
8HTe.00	referral to diabetes preconception counselling clinic	Diabetes
66Af.00	patient diabetes education review	Diabetes
8Hj0.00	referral to diabetes structured education programme	Diabetes
68A9.00	diabetic retinopathy screening offered	Diabetes
8CS0.00	diabetes care plan agreed	Diabetes
8Hg4.00	discharged from care of diabetes specialist nurse	Diabetes
8I57.00	patient held diabetic record declined	Diabetes
9N0n.00	seen in community diabetes specialist clinic	Diabetes
679R.00	patient offered diabetes structured education programme	Diabetes
9N0o.00	seen in community diabetic specialist nurse clinic	Diabetes
9N0m.00	seen in diabetic nurse consultant clinic	Diabetes
2BBo.00	o/e - sight threatening diabetic retinopathy	Diabetes
9OLD.00	diabetic patient unsuitable for digital retinal photography	Diabetes
42WZ.00	hb. a1c - diabetic control nos	Diabetes
44V3.00	glucose tol. test diabetic	Diabetes
C101.00	diabetes mellitus with ketoacidosis	Diabetes
66A..00	diabetic monitoring	Diabetes
66A1.00	initial diabetic assessment	Diabetes
66A2.00	follow-up diabetic assessment	Diabetes
66A3.00	diabetic on diet only	Diabetes
66A4.00	diabetic on oral treatment	Diabetes
66A5.00	diabetic on insulin	Diabetes
66A8.00	has seen dietician - diabetes	Diabetes
66A9.00	understands diet - diabetes	Diabetes
66AD.00	fundoscopy - diabetic check	Diabetes
66AG.00	diabetic drug side effects	Diabetes
66AH.00	diabetic treatment changed	Diabetes
66AI.00	diabetic - good control	Diabetes
66AJz00	diabetic - poor control nos	Diabetes
66AK.00	diabetic - cooperative patient	Diabetes
66AL.00	diabetic-uncooperative patient	Diabetes
66AM.00	diabetic - follow-up default	Diabetes
66AN.00	date diabetic treatment start	Diabetes
66AP.00	diabetes: practice programme	Diabetes

66AQ.00	diabetes: shared care programme	Diabetes
66AR.00	diabetes management plan given	Diabetes
66AS.00	diabetic annual review	Diabetes
66AT.00	annual diabetic blood test	Diabetes
66AZ.00	diabetic monitoring nos	Diabetes
C10ER00	latent autoimmune diabetes mellitus in adult	Diabetes
679L.00	health education - diabetes	Diabetes
C109D00	non-insulin dependent diabetes mellitus with hypoglyca coma	Diabetes
C109A00	non-insulin dependent diabetes mellitus with mononeuropathy	Diabetes
C109C00	non-insulin dependent diabetes mellitus with nephropathy	Diabetes
C109B00	non-insulin dependent diabetes mellitus with polyneuropathy	Diabetes
C109400	non-insulin dependent diabetes mellitus with ulcer	Diabetes
C109F00	non-insulin-dependent d m with peripheral angiopath	Diabetes
C109.00	non-insulin dependent diabetes mellitus	Diabetes
C109300	non-insulin-dependent diabetes mellitus with multiple comps	Diabetes
C109200	non-insulin-dependent diabetes mellitus with neuro comps	Diabetes
C109E00	non-insulin depend diabetes mellitus with diabetic cataract	Diabetes
C109700	non-insulin dependent diabetes mellitus - poor control	Diabetes
C109H00	non-insulin dependent d m with neuropathic arthropathy	Diabetes
C109500	non-insulin dependent diabetes mellitus with gangrene	Diabetes
8A12.00	diabetic crisis monitoring	Diabetes
8A13.00	diabetic stabilisation	Diabetes
8CE0.00	diabetic leaflet given	Diabetes
8H2J.00	admit diabetic emergency	Diabetes
8H3O.00	non-urgent diabetic admission	Diabetes
8H7C.00	refer, diabetic liaison nurse	Diabetes
8HKE.00	diabetology d.v. requested	Diabetes
8HLE.00	diabetology d.v. done	Diabetes
8HME.00	listed for diabetology admissn	Diabetes
8HVU.00	private referral to diabetologist	Diabetes
9N1Q.00	seen in diabetic clinic	Diabetes
9OL1.00	attends diabetes monitoring	Diabetes
9OL2.00	refuses diabetes monitoring	Diabetes
9OL3.00	diabetes monitoring default	Diabetes
C100.00	diabetes mellitus with no mention of complication	Diabetes
C100z00	diabetes mellitus nos with no mention of complication	Diabetes
C101y00	other specified diabetes mellitus with ketoacidosis	Diabetes
C101z00	diabetes mellitus nos with ketoacidosis	Diabetes
C102.00	diabetes mellitus with hyperosmolar coma	Diabetes
C102000	diabetes mellitus, juvenile type, with hyperosmolar coma	Diabetes
C102100	diabetes mellitus, adult onset, with hyperosmolar coma	Diabetes
C102z00	diabetes mellitus nos with hyperosmolar coma	Diabetes
C103y00	other specified diabetes mellitus with coma	Diabetes
C103z00	diabetes mellitus nos with ketoacidotic coma	Diabetes
C104y00	other specified diabetes mellitus with renal complications	Diabetes
C104z00	diabetes mellitis with nephropathy nos	Diabetes

C105z00	diabetes mellitus nos with ophthalmic manifestation	Diabetes
C106z00	diabetes mellitus nos with neurological manifestation	Diabetes
C107z00	diabetes mellitus nos with peripheral circulatory disorder	Diabetes
C10E511	type i diabetes mellitus with ulcer	Diabetes
C10E500	type 1 diabetes mellitus with ulcer	Diabetes
C10E600	type 1 diabetes mellitus with gangrene	Diabetes
C10E611	type i diabetes mellitus with gangrene	Diabetes
C10E800	type 1 diabetes mellitus - poor control	Diabetes
C10E811	type i diabetes mellitus - poor control	Diabetes
C10E911	type i diabetes mellitus maturity onset	Diabetes
C10E900	type 1 diabetes mellitus maturity onset	Diabetes
C108z00	unspecified diabetes mellitus with multiple complications	Diabetes
C10F311	type ii diabetes mellitus with multiple complications	Diabetes
C10F300	type 2 diabetes mellitus with multiple complications	Diabetes
C10F400	type 2 diabetes mellitus with ulcer	Diabetes
C10F411	type ii diabetes mellitus with ulcer	Diabetes
C10F511	type ii diabetes mellitus with gangrene	Diabetes
C10F500	type 2 diabetes mellitus with gangrene	Diabetes
C10F711	type ii diabetes mellitus - poor control	Diabetes
C10F700	type 2 diabetes mellitus - poor control	Diabetes
C10A100	malnutrition-related diabetes mellitus with ketoacidosis	Diabetes
C10y.00	diabetes mellitus with other specified manifestation	Diabetes
C10yz00	diabetes mellitus nos with other specified manifestation	Diabetes
C10z.00	diabetes mellitus with unspecified complication	Diabetes
C10zz00	diabetes mellitus nos with unspecified complication	Diabetes
Cyu2000	[x]other specified diabetes mellitus	Diabetes
Cyu2300	[x]unspecified diabetes mellitus with renal complications	Diabetes
F345000	diabetic mononeuritis multiplex	Diabetes
F35z000	diabetic mononeuritis nos	Diabetes
F372000	acute painful diabetic neuropathy	Diabetes
F372100	chronic painful diabetic neuropathy	Diabetes
F372200	asymptomatic diabetic neuropathy	Diabetes
F374z00	polyneuropathy in disease nos	Diabetes
F420200	preproliferative diabetic retinopathy	Diabetes
F420300	advanced diabetic maculopathy	Diabetes
F420z00	diabetic retinopathy nos	Diabetes
F440700	diabetic iritis	Diabetes
66Ai.00	diabetic 6 month review	Diabetes
C10EQ00	type 1 diabetes mellitus with gastroparesis	Diabetes
C10FR00	type 2 diabetes mellitus with gastroparesis	Diabetes
8HTi.00	referral to multidisciplinary diabetic clinic	Diabetes
9N1o.00	seen in multidisciplinary diabetic clinic	Diabetes
9N4p.00	did not attend diabetic retinopathy clinic	Diabetes
66Ak.00	diabetic monitoring - lower risk albumin excretion	Diabetes
66Al.00	diabetic monitoring - higher risk albumin excretion	Diabetes
G73y000	diabetic peripheral angiopathy	Diabetes

K01x100	nephrotic syndrome in diabetes mellitus	Diabetes
K01x111	kimmelstiel - wilson disease	Diabetes
Kyu0300	[x]glomerular disorders in diabetes mellitus	Diabetes
L180500	pre-existing diabetes mellitus, insulin-dependent	Diabetes
L180600	pre-existing diabetes mellitus, non-insulin-dependent	Diabetes
L180700	pre-existing malnutrition-related diabetes mellitus	Diabetes
8HHy.00	referral to diabetic register	Diabetes
93C4.00	patient consent given for addition to diabetic register	Diabetes
M037200	cellulitis in diabetic foot	Diabetes
8HTk.00	referral to diabetic eye clinic	Diabetes
M271000	ischaemic ulcer diabetic foot	Diabetes
M271200	mixed diabetic ulcer - foot	Diabetes
N030000	diabetic cheiroarthropathy	Diabetes
N030011	diabetic cheiropathy	Diabetes
R054200	[d]gangrene of toe in diabetic	Diabetes
R054300	[d]widespread diabetic foot gangrene	Diabetes
TJ23z00	adverse reaction to insulins and antidiabetic agents nos	Diabetes
F372.12	diabetic neuropathy	Diabetes
C106.12	diabetes mellitus with neuropathy	Diabetes
F3y0.00	diabetic mononeuropathy	Diabetes
F420400	diabetic maculopathy	Diabetes
C314.11	renal diabetes	Diabetes
K08yA11	clinical diabetic nephropathy	Diabetes
K08yA00	proteinuric diabetic nephropathy	Diabetes
U602311	[x] adverse reaction to insulins and antidiabetic agents	Diabetes
U60231E	[x] adverse reaction to insulins and antidiabetic agents nos	Diabetes
C10D.11	maturity onset diabetes in youth type 2	Diabetes
Cyu2.00	[x]diabetes mellitus	Diabetes
F381300	myasthenic syndrome due to diabetic amyotrophy	Diabetes
66AJ.00	diabetic - poor control	Diabetes
42W..00	hb. a1c - diabetic control	Diabetes
9OLA.11	diabetes monitored	Diabetes
68AB.00	diabetic digital retinopathy screening offered	Diabetes
8Hl1.00	referral for diabetic retinopathy screening	Diabetes
3883.00	diabetes treatment satisfaction questionnaire	Diabetes
66AJ.11	unstable diabetes	Diabetes
13L4.11	diabetic child	Diabetes
C10E411	unstable type i diabetes mellitus	Diabetes
C10E400	unstable type 1 diabetes mellitus	Diabetes
C10E412	unstable insulin dependent diabetes mellitus	Diabetes
9N2i.00	seen by diabetic liaison nurse	Diabetes
ZLD7500	discharge by diabetic liaison nurse	Diabetes
2G5A.00	o/e - right diabetic foot at risk	Diabetes
2G5B.00	o/e - left diabetic foot at risk	Diabetes
3881.00	education score - diabetes	Diabetes
14F4.00	h/o: admission in last year for diabetes foot problem	Diabetes

42c..00	hba1 - diabetic control	Diabetes
F420500	advanced diabetic retinal disease	Diabetes
8H7f.00	referral to diabetes nurse	Diabetes
9NM0.00	attending diabetes clinic	Diabetes
9N1v.00	seen in diabetic eye clinic	Diabetes
C10EA11	type i diabetes mellitus without complication	Diabetes
C10EA00	type 1 diabetes mellitus without complication	Diabetes
C10EA12	insulin-dependent diabetes without complication	Diabetes
C109900	non-insulin-dependent diabetes mellitus without complication	Diabetes
C10F900	type 2 diabetes mellitus without complication	Diabetes
C10F911	type ii diabetes mellitus without complication	Diabetes
66AV.00	diabetic on insulin and oral treatment	Diabetes
C10EE00	type 1 diabetes mellitus with hypoglycaemic coma	Diabetes
C10EE12	insulin dependent diabetes mellitus with hypoglycaemic coma	Diabetes
C10FD00	type 2 diabetes mellitus with hypoglycaemic coma	Diabetes
C10FD11	type ii diabetes mellitus with hypoglycaemic coma	Diabetes
C10EH00	type 1 diabetes mellitus with arthropathy	Diabetes
C10EJ00	type 1 diabetes mellitus with neuropathic arthropathy	Diabetes
C10FF11	type ii diabetes mellitus with peripheral angiopathy	Diabetes
C10FF00	type 2 diabetes mellitus with peripheral angiopathy	Diabetes
C109G00	non-insulin dependent diabetes mellitus with arthropathy	Diabetes
C10FG00	type 2 diabetes mellitus with arthropathy	Diabetes
C10FG11	type ii diabetes mellitus with arthropathy	Diabetes
C10FH11	type ii diabetes mellitus with neuropathic arthropathy	Diabetes
C10FH00	type 2 diabetes mellitus with neuropathic arthropathy	Diabetes
13AB.00	diabetic lipid lowering diet	Diabetes
13AC.00	diabetic weight reducing diet	Diabetes
TJ23.00	adverse reaction to insulins and antidiabetic agents	Diabetes
66An.00	diabetes type 1 review	Diabetes
66Ao.00	diabetes type 2 review	Diabetes
8Hl4.00	referral to community diabetes specialist nurse	Diabetes
C10C.00	diabetes mellitus autosomal dominant	Diabetes
C10C.11	maturity onset diabetes in youth	Diabetes
C109.11	niddm - non-insulin dependent diabetes mellitus	Diabetes
C10F.11	type ii diabetes mellitus	Diabetes
C10E.11	type i diabetes mellitus	Diabetes
C10N100	cystic fibrosis related diabetes mellitus	Diabetes
9OLF.00	diabetes structured education programme completed	Diabetes
9OLG.00	attended xpert diabetes structured education programme	Diabetes
9OLH.00	attended dafne diabetes structured education programme	Diabetes
9OLJ.00	dafne diabetes structured education programme completed	Diabetes
9OLK.00	desmond diabetes structured education programme completed	Diabetes
9OLL.00	xpert diabetes structured education programme completed	Diabetes
9OLM.00	diabetes structured education programme declined	Diabetes
8Hj3.00	referral to dafne diabetes structured education programme	Diabetes
8Hj4.00	referral to desmond diabetes structured education programme	Diabetes

8Hj5.00	referral to xpert diabetes structured education programme	Diabetes
9NiA.00	did not attend diabetes structured education programme	Diabetes
8I81.00	did not complete diabetes structured education programme	Diabetes
8I82.00	did not complete dafne diabetes structured education program	Diabetes
9NiC.00	did not attend dafne diabetes structured education programme	Diabetes
9NiE.00	did not attend xpert diabetes structured education programme	Diabetes
C109100	non-insulin-dependent diabetes mellitus with ophthalm comps	Diabetes
C109000	non-insulin-dependent diabetes mellitus with renal comps	Diabetes
66AO.00	date diabetic treatment stopp.	Diabetes
L180000	diabetes mellitus - unspec whether in pregnancy/puerperium	Diabetes
C10B.00	diabetes mellitus induced by steroids	Diabetes
C107.11	diabetes mellitus with gangrene	Diabetes
C103.00	diabetes mellitus with ketoacidotic coma	Diabetes
C106.00	diabetes mellitus with neurological manifestation	Diabetes
C105.00	diabetes mellitus with ophthalmic manifestation	Diabetes
C107.00	diabetes mellitus with peripheral circulatory disorder	Diabetes
C106.13	diabetes mellitus with polyneuropathy	Diabetes
C104.00	diabetes mellitus with renal manifestation	Diabetes
C106100	diabetes mellitus, adult onset, + neurological manifestation	Diabetes
C105100	diabetes mellitus, adult onset, + ophthalmic manifestation	Diabetes
C10z100	diabetes mellitus, adult onset, + unspecified complication	Diabetes
C100100	diabetes mellitus, adult onset, no mention of complication	Diabetes
C101100	diabetes mellitus, adult onset, with ketoacidosis	Diabetes
C103100	diabetes mellitus, adult onset, with ketoacidotic coma	Diabetes
C104100	diabetes mellitus, adult onset, with renal manifestation	Diabetes
C107200	diabetes mellitus, adult with gangrene	Diabetes
C10y100	diabetes mellitus, adult, + other specified manifestation	Diabetes
C107100	diabetes mellitus, adult, + peripheral circulatory disorder	Diabetes
C107000	diabetes mellitus, juvenile +peripheral circulatory disorder	Diabetes
C105000	diabetes mellitus, juvenile type, + ophthalmic manifestation	Diabetes
C10z000	diabetes mellitus, juvenile type, + unspecified complication	Diabetes
C100000	diabetes mellitus, juvenile type, no mention of complication	Diabetes
C101000	diabetes mellitus, juvenile type, with ketoacidosis	Diabetes
C103000	diabetes mellitus, juvenile type, with ketoacidotic coma	Diabetes
C104000	diabetes mellitus, juvenile type, with renal manifestation	Diabetes
C106000	diabetes mellitus, juvenile, + neurological manifestation	Diabetes
9OLA.00	diabetes monitor. check done	Diabetes
9OL8.00	diabetes monitor.phone invite	Diabetes
9OL7.00	diabetes monitor.verbal invite	Diabetes
9OL4.00	diabetes monitoring 1st letter	Diabetes
9OL5.00	diabetes monitoring 2nd letter	Diabetes
9OL6.00	diabetes monitoring 3rd letter	Diabetes
3882.00	diabetes well being questionnaire	Diabetes
C107.12	diabetes with gangrene	Diabetes
C106.11	diabetic amyotrophy	Diabetes
N030100	diabetic charcot arthropathy	Diabetes

8H4e.00	referral to diabetes special interest general practitioner	Diabetes
C10FS00	maternally inherited diabetes mellitus	Diabetes
66AY.00	diabetic diet - good compliance	Diabetes
66Aa.00	diabetic diet - poor compliance	Diabetes
C109600	non-insulin-dependent diabetes mellitus with retinopathy	Diabetes
F381311	diabetic amyotrophy	Diabetes
C107400	niddm with peripheral circulatory disorder	Diabetes
66Aq.00	diabetic foot screen	Diabetes
C10A.00	malnutrition-related diabetes mellitus	Diabetes
C10A000	malnutrition-related diabetes mellitus with coma	Diabetes
C10A500	malnutritn-relat diabetes melitus wth periph circul complctn	Diabetes
C108400	unstable insulin dependent diabetes mellitus	Diabetes
C108412	unstable type 1 diabetes mellitus	Diabetes
C108411	unstable type i diabetes mellitus	Diabetes
9m00.00	eligible for diabetic retinopathy screening	Diabetes
F464000	diabetic cataract	Diabetes
2BBr.00	impaired vision due to diabetic retinopathy	Diabetes
C100112	non-insulin dependent diabetes mellitus	Diabetes
9m05.00	excluded from diabetic retinopathy screening as moved away	Diabetes
9m06.00	excluded from diabetic retinopathy screening as deceased	Diabetes
9m08.00	excluded from diabetic retinopathy screening as blind	Diabetes
9m04.00	excluded from diabetic retinopathy screening	Diabetes
C108A00	insulin-dependent diabetes without complication	Diabetes
2G5C.00	foot abnormality - diabetes related	Diabetes
C108H00	insulin dependent diabetes mellitus with arthropathy	Diabetes
C108F00	insulin dependent diabetes mellitus with diabetic cataract	Diabetes
C108600	insulin dependent diabetes mellitus with gangrene	Diabetes
C108E00	insulin dependent diabetes mellitus with hypoglycaemic coma	Diabetes
C108B00	insulin dependent diabetes mellitus with mononeuropathy	Diabetes
C108300	insulin dependent diabetes mellitus with multiple complicatn	Diabetes
C108D00	insulin dependent diabetes mellitus with nephropathy	Diabetes
C108C00	insulin dependent diabetes mellitus with polyneuropathy	Diabetes
C108700	insulin dependent diabetes mellitus with retinopathy	Diabetes
C108500	insulin dependent diabetes mellitus with ulcer	Diabetes
C108200	insulin-dependent diabetes mellitus with neurological comps	Diabetes
C108100	insulin-dependent diabetes mellitus with ophthalmic comps	Diabetes
C108000	insulin-dependent diabetes mellitus with renal complications	Diabetes
C108J00	insulin dependent diab mell with neuropathic arthropathy	Diabetes
C108G00	insulin dependent diab mell with peripheral angiopathy	Diabetes
C108900	insulin dependent diabetes maturity onset	Diabetes
C100011	insulin dependent diabetes mellitus	Diabetes
C108.00	insulin dependent diabetes mellitus	Diabetes
C108800	insulin dependent diabetes mellitus - poor control	Diabetes
66AA.11	injection sites - diabetic	Diabetes
C10E.12	insulin dependent diabetes mellitus	Diabetes
C107300	iddm with peripheral circulatory disorder	Diabetes

C108.11	iddm-insulin dependent diabetes mellitus	Diabetes
F372.11	diabetic polyneuropathy	Diabetes
Q441.00	neonatal diabetes mellitus	Diabetes
C109J00	insulin treated type 2 diabetes mellitus	Diabetes
C10D.00	diabetes mellitus autosomal dominant type 2	Diabetes
F420800	high risk non proliferative diabetic retinopathy	Diabetes
C109J12	insulin treated type ii diabetes mellitus	Diabetes
C108.12	type 1 diabetes mellitus	Diabetes
C108812	type 1 diabetes mellitus - poor control	Diabetes
C108912	type 1 diabetes mellitus maturity onset	Diabetes
C108E12	type 1 diabetes mellitus with hypoglycaemic coma	Diabetes
C108212	type 1 diabetes mellitus with neurological complications	Diabetes
C108J12	type 1 diabetes mellitus with neuropathic arthropathy	Diabetes
C108112	type 1 diabetes mellitus with ophthalmic complications	Diabetes
C108012	type 1 diabetes mellitus with renal complications	Diabetes
C108712	type 1 diabetes mellitus with retinopathy	Diabetes
C108512	type 1 diabetes mellitus with ulcer	Diabetes
C109.12	type 2 diabetes mellitus	Diabetes
C109712	type 2 diabetes mellitus - poor control	Diabetes
C109G12	type 2 diabetes mellitus with arthropathy	Diabetes
C109E12	type 2 diabetes mellitus with diabetic cataract	Diabetes
C109512	type 2 diabetes mellitus with gangrene	Diabetes
C109D12	type 2 diabetes mellitus with hypoglycaemic coma	Diabetes
66AX.00	diabetes: shared care in pregnancy - diabetol and obstet	Diabetes
C109312	type 2 diabetes mellitus with multiple complications	Diabetes
C109C12	type 2 diabetes mellitus with nephropathy	Diabetes
C109212	type 2 diabetes mellitus with neurological complications	Diabetes
C109H12	type 2 diabetes mellitus with neuropathic arthropathy	Diabetes
C109112	type 2 diabetes mellitus with ophthalmic complications	Diabetes
C109F12	type 2 diabetes mellitus with peripheral angiopathy	Diabetes
C109012	type 2 diabetes mellitus with renal complications	Diabetes
C109612	type 2 diabetes mellitus with retinopathy	Diabetes
C109412	type 2 diabetes mellitus with ulcer	Diabetes
C109912	type 2 diabetes mellitus without complication	Diabetes
C108.13	type i diabetes mellitus	Diabetes
C108811	type i diabetes mellitus - poor control	Diabetes
C108911	type i diabetes mellitus maturity onset	Diabetes
C108H11	type i diabetes mellitus with arthropathy	Diabetes
C108F11	type i diabetes mellitus with diabetic cataract	Diabetes
C108E11	type i diabetes mellitus with hypoglycaemic coma	Diabetes
C108B11	type i diabetes mellitus with mononeuropathy	Diabetes
C108311	type i diabetes mellitus with multiple complications	Diabetes
C108D11	type i diabetes mellitus with nephropathy	Diabetes
C108211	type i diabetes mellitus with neurological complications	Diabetes
C108J11	type i diabetes mellitus with neuropathic arthropathy	Diabetes
C108C11	type i diabetes mellitus with polyneuropathy	Diabetes

C108011	type i diabetes mellitus with renal complications	Diabetes
C108711	type i diabetes mellitus with retinopathy	Diabetes
C108511	type i diabetes mellitus with ulcer	Diabetes
C108A11	type i diabetes mellitus without complication	Diabetes
C109.13	type ii diabetes mellitus	Diabetes
C109711	type ii diabetes mellitus - poor control	Diabetes
C109G11	type ii diabetes mellitus with arthropathy	Diabetes
C109E11	type ii diabetes mellitus with diabetic cataract	Diabetes
C109511	type ii diabetes mellitus with gangrene	Diabetes
C109D11	type ii diabetes mellitus with hypoglycaemic coma	Diabetes
C109A11	type ii diabetes mellitus with mononeuropathy	Diabetes
C109C11	type ii diabetes mellitus with nephropathy	Diabetes
C109211	type ii diabetes mellitus with neurological complications	Diabetes
C109H11	type ii diabetes mellitus with neuropathic arthropathy	Diabetes
C109111	type ii diabetes mellitus with ophthalmic complications	Diabetes
C109F11	type ii diabetes mellitus with peripheral angiopathy	Diabetes
C109B11	type ii diabetes mellitus with polyneuropathy	Diabetes
C109011	type ii diabetes mellitus with renal complications	Diabetes
C109611	type ii diabetes mellitus with retinopathy	Diabetes
C109411	type ii diabetes mellitus with ulcer	Diabetes
C109911	type ii diabetes mellitus without complication	Diabetes
C109K00	hyperosmolar non-ketotic state in type 2 diabetes mellitus	Diabetes
F420.00	diabetic retinopathy	Diabetes
C10E012	insulin-dependent diabetes mellitus with renal complications	Diabetes
C10E000	type 1 diabetes mellitus with renal complications	Diabetes
C10E112	insulin-dependent diabetes mellitus with ophthalmic comps	Diabetes
C10E100	type 1 diabetes mellitus with ophthalmic complications	Diabetes
C10E111	type i diabetes mellitus with ophthalmic complications	Diabetes
C10E212	insulin-dependent diabetes mellitus with neurological comps	Diabetes
C10E200	type 1 diabetes mellitus with neurological complications	Diabetes
C10E312	insulin dependent diabetes mellitus with multiple complicat	Diabetes
C10E300	type 1 diabetes mellitus with multiple complications	Diabetes
C10E311	type i diabetes mellitus with multiple complications	Diabetes
C10E512	insulin dependent diabetes mellitus with ulcer	Diabetes
C10E612	insulin dependent diabetes mellitus with gangrene	Diabetes
C10E712	insulin dependent diabetes mellitus with retinopathy	Diabetes
C10E700	type 1 diabetes mellitus with retinopathy	Diabetes
C10E711	type i diabetes mellitus with retinopathy	Diabetes
C10E812	insulin dependent diabetes mellitus - poor control	Diabetes
C10E912	insulin dependent diabetes maturity onset	Diabetes
C10EB00	type 1 diabetes mellitus with mononeuropathy	Diabetes
C10EC12	insulin dependent diabetes mellitus with polyneuropathy	Diabetes
C10EC00	type 1 diabetes mellitus with polyneuropathy	Diabetes
C10EC11	type i diabetes mellitus with polyneuropathy	Diabetes
C10ED12	insulin dependent diabetes mellitus with nephropathy	Diabetes
C10ED00	type 1 diabetes mellitus with nephropathy	Diabetes

C10EF12	insulin dependent diabetes mellitus with diabetic cataract	Diabetes
C10EF00	type 1 diabetes mellitus with diabetic cataract	Diabetes
C10EG00	type 1 diabetes mellitus with peripheral angiopathy	Diabetes
C10F000	type 2 diabetes mellitus with renal complications	Diabetes
C10F011	type ii diabetes mellitus with renal complications	Diabetes
C10F100	type 2 diabetes mellitus with ophthalmic complications	Diabetes
C10F111	type ii diabetes mellitus with ophthalmic complications	Diabetes
C10F200	type 2 diabetes mellitus with neurological complications	Diabetes
C10F211	type ii diabetes mellitus with neurological complications	Diabetes
C10F600	type 2 diabetes mellitus with retinopathy	Diabetes
C10F611	type ii diabetes mellitus with retinopathy	Diabetes
C10FA00	type 2 diabetes mellitus with mononeuropathy	Diabetes
C10FA11	type ii diabetes mellitus with mononeuropathy	Diabetes
C10FB00	type 2 diabetes mellitus with polyneuropathy	Diabetes
C10FB11	type ii diabetes mellitus with polyneuropathy	Diabetes
C10FC00	type 2 diabetes mellitus with nephropathy	Diabetes
C10FC11	type ii diabetes mellitus with nephropathy	Diabetes
C10FE00	type 2 diabetes mellitus with diabetic cataract	Diabetes
C10FE11	type ii diabetes mellitus with diabetic cataract	Diabetes
C10FJ00	insulin treated type 2 diabetes mellitus	Diabetes
C10EK00	type 1 diabetes mellitus with persistent proteinuria	Diabetes
C10EL11	type i diabetes mellitus with persistent microalbuminuria	Diabetes
C10EM00	type 1 diabetes mellitus with ketoacidosis	Diabetes
C10EM11	type i diabetes mellitus with ketoacidosis	Diabetes
C10EN00	type 1 diabetes mellitus with ketoacidotic coma	Diabetes
C10EN11	type i diabetes mellitus with ketoacidotic coma	Diabetes
C10FL00	type 2 diabetes mellitus with persistent proteinuria	Diabetes
C10FL11	type ii diabetes mellitus with persistent proteinuria	Diabetes
C10FM00	type 2 diabetes mellitus with persistent microalbuminuria	Diabetes
C10FM11	type ii diabetes mellitus with persistent microalbuminuria	Diabetes
C10FN00	type 2 diabetes mellitus with ketoacidosis	Diabetes
C10FN11	type ii diabetes mellitus with ketoacidosis	Diabetes
C10FP00	type 2 diabetes mellitus with ketoacidotic coma	Diabetes
C10FP11	type ii diabetes mellitus with ketoacidotic coma	Diabetes
9h41.00	excepted from diabetes qual indicators: patient unsuitable	Diabetes
C10EP00	type 1 diabetes mellitus with exudative maculopathy	Diabetes
C10EP11	type i diabetes mellitus with exudative maculopathy	Diabetes
C10FQ00	type 2 diabetes mellitus with exudative maculopathy	Diabetes
C10H000	dm induced by non-steroid drugs without complication	Diabetes
C10M.00	lipoatrophic diabetes mellitus	Diabetes
2BBk.00	o/e - right eye stable treated prolif diabetic retinopathy	Diabetes
2BBl.00	o/e - left eye stable treated prolif diabetic retinopathy	Diabetes
8Hj1.00	family/carer referral to diabetes structured education prog	Diabetes
F420100	proliferative diabetic retinopathy	Diabetes
ZC2C800	dietary advice for diabetes mellitus	Diabetes
250 CT	diabetic cataract	Diabetes

ZL62500	referral to diabetes nurse	Diabetes
ZL62600	referral to diabetic liaison nurse	Diabetes
250 AT	diabetic amyotrophy	Diabetes
250 N	diabetic nephropathy	Diabetes
ZC2CA00	dietary advice for type ii diabetes	Diabetes
ZRbH.00	perceived control of insulin-dependent diabetes	Diabetes
C104z00	diabetes mellitus with nephropathy nos	Diabetes
ZRB6.00	diabetes wellbeing questionnaire	Diabetes
ZL22500	under care of diabetic liaison nurse	Diabetes
1434.00	h/o: diabetes mellitus	Diabetes
ZRB4.00	diabetes clinic satisfaction questionnaire	Diabetes
ZRB5.11	dtsq - diabetes treatment satisfaction questionnaire	Diabetes
ZC2C900	dietary advice for type i diabetes	Diabetes
ZLA2500	seen by diabetic liaison nurse	Diabetes
ZRB4.11	csq - diabetes clinic satisfaction questionnaire	Diabetes
ZRB5.00	diabetes treatment satisfaction questionnaire	Diabetes
ZRB6.11	dwbq - diabetes wellbeing questionnaire	Diabetes
44I4200	low serum potassium level	Fluid and electrolyte disorders
C362200	lactic acidosis	Fluid and electrolyte disorders
C363100	respiratory alkalosis	Fluid and electrolyte disorders
C362100	respiratory acidosis	Fluid and electrolyte disorders
44I4100	raised serum potassium level	Fluid and electrolyte disorders
44I8100	raised serum calcium level	Fluid and electrolyte disorders
44L2100	blood magnesium abnormal	Fluid and electrolyte disorders
46M2.00	urine electrolytes abnormal	Fluid and electrolyte disorders
C36z100	hypochloraemia	Fluid and electrolyte disorders
C352z00	disorder of magnesium metabolism nos	Fluid and electrolyte disorders
C354000	hypocalcaemia nec	Fluid and electrolyte disorders
C354200	idiopathic hypercalcaemia	Fluid and electrolyte disorders
C354500	familial benign hypercalcaemia	Fluid and electrolyte disorders
C354z00	disorder of calcium metabolism nos	Fluid and electrolyte disorders
C362000	metabolic acidosis nec	Fluid and electrolyte disorders
C362y00	other specified acidosis	Fluid and electrolyte disorders
C363200	metabolic alkalaemia	Fluid and electrolyte disorders
C363y00	other specified alkalosis	Fluid and electrolyte disorders
C363z00	alkalosis nos	Fluid and electrolyte disorders
C365z00	volume depletion nos	Fluid and electrolyte disorders
C36zz00	electrolyte imbalance nos	Fluid and electrolyte disorders
C363000	metabolic alkalosis	Fluid and electrolyte disorders
C366111	idiopathic fluid retention	Fluid and electrolyte disorders
C36A.00	respiratory acidosis and metabolic alkalosis	Fluid and electrolyte disorders
C354111	secondary hypercalcaemia	Fluid and electrolyte disorders
C354900	calcium deficiency	Fluid and electrolyte disorders
C294400	magnesium deficiency	Fluid and electrolyte disorders
C363.00	alkalosis	Fluid and electrolyte disorders
C354100	hypercalcaemia nec	Fluid and electrolyte disorders

C360.00	hyperosmolality and or hypernatraemia	Fluid and electrolyte disorders
C36z.00	disorders of fluid, electrolyte and acid-base balance nec	Fluid and electrolyte disorders
8B77.00	potassium supplementation	Fluid and electrolyte disorders
C367.00	hyperkalaemia	Fluid and electrolyte disorders
C365.00	volume depletion	Fluid and electrolyte disorders
C365100	hypovolaemia	Fluid and electrolyte disorders
C360.11	hypernatraemia	Fluid and electrolyte disorders
C368.00	hypokalaemia	Fluid and electrolyte disorders
C36z000	hyperchloraemia	Fluid and electrolyte disorders
C363500	alkalaemia	Fluid and electrolyte disorders
C361.11	hyponatraemia	Fluid and electrolyte disorders
C36..00	disorders of fluid, electrolyte and acid-base balance	Fluid and electrolyte disorders
C352.00	disorders of magnesium metabolism	Fluid and electrolyte disorders
C36..11	electrolyte disorders	Fluid and electrolyte disorders
C362900	compensated metabolic acidosis	Fluid and electrolyte disorders
C361.00	hyposmolality and or hyponatraemia	Fluid and electrolyte disorders
A788.00	acquired immune deficiency syndrome	HIV/AIDS
66j..00	human immunodeficiency virus monitoring	HIV/AIDS
66j0.00	human immunodeficiency virus annual review	HIV/AIDS
A788.11	human immunodeficiency virus infection	HIV/AIDS
A788100	asymptomatic human immunodeficiency virus infection	HIV/AIDS
43h9.00	hiv proviral deoxyribonucleic acid polymerase chain reaction	HIV/AIDS
43w3.00	human immunodeficiency virus rna/dna ratio	HIV/AIDS
9kl..00	hiv pos gen health check serv declind - enhanc service admin	HIV/AIDS
43j7.00	hiv 1 nucleic acid detection	HIV/AIDS
8Hle.00	referral to community hiv nurse specialist	HIV/AIDS
A789311	hiv disease resulting in pneumocystis jirovecii pneumonia	HIV/AIDS
A789511	hiv disease resulting in kaposi sarcoma	HIV/AIDS
L179.00	hiv disease complicating pregnancy childbirth puerperium	HIV/AIDS
9Nt1000	seen by community hiv (human immunodeficiency virus) nurse	HIV/AIDS
43C3.11	hiv positive	HIV/AIDS
A789A00	hiv disease resulting in wasting syndrome	HIV/AIDS
65QA.00	aids carrier	HIV/AIDS
65VE.00	notification of aids	HIV/AIDS
A788400	human immunodeficiency virus with neurological disease	HIV/AIDS
A788600	human immunodeficiency virus with secondary cancers	HIV/AIDS
A788y00	human immunodeficiency virus with other clinical findings	HIV/AIDS
A788z00	acquired human immunodeficiency virus infection syndrome nos	HIV/AIDS
A789.00	human immunodef virus resulting in other disease	HIV/AIDS
A789000	hiv disease resulting in mycobacterial infection	HIV/AIDS
A789100	hiv disease resulting in cytomegaloviral disease	HIV/AIDS
A789200	hiv disease resulting in candidiasis	HIV/AIDS
A789400	hiv disease resulting in multiple infections	HIV/AIDS
A789800	hiv disease resulting in multiple malignant neoplasms	HIV/AIDS
AyuC.00	[x]human immunodeficiency virus disease	HIV/AIDS
AyuC000	[x]hiv disease resulting in other bacterial infections	HIV/AIDS

AyuC100	[x]hiv disease resulting in other viral infections	HIV/AIDS
AyuC200	[x]hiv disease resulting in other mycoses	HIV/AIDS
AyuC600	[x]hiv disease resulting in other non-hodgkin's lymphoma	HIV/AIDS
AyuC800	[x]hiv disease resulting in other malignant neoplasms	HIV/AIDS
AyuC900	[x]hiv disease resulting in unspecified malignant neoplasm	HIV/AIDS
AyuCC00	[x]hiv disease resulting in other specified conditions	HIV/AIDS
AyuCD00	[x]unspecified human immunodeficiency virus [hiv] disease	HIV/AIDS
R109.00	[d]laboratory evidence of human immunodeficiency virus [hiv]	HIV/AIDS
ZV01A00	[v]asymptomatic human immunodeficency virus infection status	HIV/AIDS
A788000	acute human immunodeficiency virus infection	HIV/AIDS
Eu02400	[x]dementia in human immunodef virus [hiv] disease	HIV/AIDS
AyuC700	[x]hiv dis reslt/oth mal neopl/lymph,h'matopoetc+reltd tissu	HIV/AIDS
AyuCB00	[x]hiv disease result/haematological+immunologic abnorms,nec	HIV/AIDS
AyuCA00	[x]hiv disease resulting in multiple diseases ce	HIV/AIDS
AyuC300	[x]hiv disease resulting in multiple infections	HIV/AIDS
AyuC400	[x]hiv disease resulting/other infectious+parasitic diseases	HIV/AIDS
AyuC500	[x]hiv disease resulting/unspcf infectious+parasitic disease	HIV/AIDS
4J34.00	hiv viral load	HIV/AIDS
4J3F.00	human immunodeficiency virus viral load by log rank	HIV/AIDS
A789X00	hiv dis reslt/oth mal neopl/lymph,h'matopoetc+reltd tissu	HIV/AIDS
A789700	hiv dis resulting oth types of non-hodgkin's lymphoma	HIV/AIDS
A788U00	hiv disease result/haematological+immunologic abnorms,nec	HIV/AIDS
A789500	hiv disease resulting in kaposi's sarcoma	HIV/AIDS
A788V00	hiv disease resulting in multiple diseases ce	HIV/AIDS
A789300	hiv disease resulting in pneumocystis carinii pneumonia	HIV/AIDS
A788W00	hiv disease resulting in unspecified malignant neoplasm	HIV/AIDS
A788X00	hiv disease resulting/unspcf infectious+parasitic disease	HIV/AIDS
A788200	hiv infection with persistent generalised lymphadenopathy	HIV/AIDS
43WK.00	human immunodeficiency virus antibody level	HIV/AIDS
A788300	human immunodeficiency virus with constitutional disease	HIV/AIDS
A788500	human immunodeficiency virus with secondary infection	HIV/AIDS
L7990A	acquired immune deficiency syndrome	HIV/AIDS
G21..00	hypertensive heart disease	Hypertension
G220.00	malignant hypertensive renal disease	Hypertension
G231.00	benign hypertensive heart and renal disease	Hypertension
G230.00	malignant hypertensive heart and renal disease	Hypertension
67H8.00	lifestyle advice regarding hypertension	Hypertension
F421300	hypertensive retinopathy	Hypertension
G200.00	malignant essential hypertension	Hypertension
G221.00	benign hypertensive renal disease	Hypertension
G23..00	hypertensive heart and renal disease	Hypertension
G240.00	secondary malignant hypertension	Hypertension
F404200	blind hypertensive eye	Hypertension
9N1y200	seen in hypertension clinic	Hypertension
G24z.00	secondary hypertension nos	Hypertension
662P000	hypertension 9 month review	Hypertension

8I3N.00	hypertension treatment refused	Hypertension
662c.00	hypertension six month review	Hypertension
662b.00	moderate hypertension control	Hypertension
662d.00	hypertension annual review	Hypertension
G25..00	stage 1 hypertension (nice - nat ins for hth clin excl 2011)	Hypertension
G26..00	severe hypertension (nat inst for health clinical ex 2011)	Hypertension
G28..00	stage 2 hypertension (nice - nat ins for hth clin excl 2011)	Hypertension
661M600	hypertension self-management plan agreed	Hypertension
8BL0.00	patient on maximal tolerated antihypertensive therapy	Hypertension
9OI..00	hypertension monitoring admin.	Hypertension
G26..11	severe hypertension	Hypertension
G25..11	stage 1 hypertension	Hypertension
G27..00	hypertension resistant to drug therapy	Hypertension
8CR4.00	hypertension clinical management plan	Hypertension
G241000	secondary benign renovascular hypertension	Hypertension
14A2.00	h/o: hypertension	Hypertension
9OI..11	hypertension clinic admin.	Hypertension
6146200	hypertension induced by oral contraceptive pill	Hypertension
6627.00	good hypertension control	Hypertension
6628.00	poor hypertension control	Hypertension
6629.00	hypertension:follow-up default	Hypertension
662F.00	hypertension treatm. started	Hypertension
662G.00	hypertensive treatm.changed	Hypertension
8HT5.00	referral to hypertension clinic	Hypertension
G210z00	malignant hypertensive heart disease nos	Hypertension
G211z00	benign hypertensive heart disease nos	Hypertension
G21zz00	hypertensive heart disease nos	Hypertension
G222.00	hypertensive renal disease with renal failure	Hypertension
G23z.00	hypertensive heart and renal disease nos	Hypertension
G240000	secondary malignant renovascular hypertension	Hypertension
G240z00	secondary malignant hypertension nos	Hypertension
G241.00	secondary benign hypertension	Hypertension
G241z00	secondary benign hypertension nos	Hypertension
G244.00	hypertension secondary to endocrine disorders	Hypertension
G24zz00	secondary hypertension nos	Hypertension
G24z000	secondary renovascular hypertension nos	Hypertension
G24z100	hypertension secondary to drug	Hypertension
G2y..00	other specified hypertensive disease	Hypertension
G2z..00	hypertensive disease nos	Hypertension
Gyu2.00	[x]hypertensive diseases	Hypertension
Gyu2000	[x]other secondary hypertension	Hypertension
Gyu2100	[x]hypertension secondary to other renal disorders	Hypertension
G201.00	benign essential hypertension	Hypertension
7Q01y00	other specified high cost hypertension drugs	Hypertension
U60C51A	[x] adverse reaction to antihypertensives nos	Hypertension
U60C511	[x] adverse reaction to other antihypertensives	Hypertension

G20z.00	essential hypertension nos	Hypertension
G22z.00	hypertensive renal disease nos	Hypertension
9OIA.11	hypertension monitored	Hypertension
G21z011	cardiomegaly - hypertensive	Hypertension
662..12	hypertension monitoring	Hypertension
662O.00	on treatment for hypertension	Hypertension
8B26.00	antihypertensive therapy	Hypertension
G211100	benign hypertensive heart disease with ccf	Hypertension
G2...11	bp - hypertensive disease	Hypertension
662q.00	trial reduction of antihypertensive therapy	Hypertension
G24..00	secondary hypertension	Hypertension
8IA5.00	trial withdrawal of antihypertensive therapy declined	Hypertension
G211.00	benign hypertensive heart disease	Hypertension
G2...00	hypertensive disease	Hypertension
G210100	malignant hypertensive heart disease with ccf	Hypertension
G210000	malignant hypertensive heart disease without ccf	Hypertension
G21z.00	hypertensive heart disease nos	Hypertension
G21z100	hypertensive heart disease nos with ccf	Hypertension
G21z000	hypertensive heart disease nos without ccf	Hypertension
G232.00	hypertensive heart&renal dis wth (congestive) heart failure	Hypertension
G234.00	hyperten heart&renal dis+both(congestv)heart and renal fail	Hypertension
662P.00	hypertension monitoring	Hypertension
G20z.11	hypertension nos	Hypertension
G203.00	diastolic hypertension	Hypertension
G672.11	hypertensive crisis	Hypertension
G210.00	malignant hypertensive heart disease	Hypertension
G202.00	systolic hypertension	Hypertension
G20..00	essential hypertension	Hypertension
G20..12	primary hypertension	Hypertension
C04..12	thyroid deficiency	Hypothyroidism
C04z.12	thyroid insufficiency	Hypothyroidism
C030.00	pendred's syndrome	Hypothyroidism
C0A5.00	subclinical iodine-deficiency hypothyroidism	Hypothyroidism
C04..00	acquired hypothyroidism	Hypothyroidism
C04z.11	pretibial myxoedema - hypothyroid	Hypothyroidism
C044.00	postinfectious hypothyroidism	Hypothyroidism
C040.11	post ablative hypothyroidism	Hypothyroidism
9Oj..00	hypothyroidism monitoring administration	Hypothyroidism
8CR5.00	hypothyroidism clinical management plan	Hypothyroidism
1432.00	h/o: hypothyroidism	Hypothyroidism
9Oj2.00	hypothyroidism monitoring third letter	Hypothyroidism
9Oj0.00	hypothyroidism monitoring first letter	Hypothyroidism
9Oj1.00	hypothyroidism monitoring second letter	Hypothyroidism
C040.00	postsurgical hypothyroidism	Hypothyroidism
9Oj3.00	hypothyroidism monitoring verbal invite	Hypothyroidism
C03..00	congenital hypothyroidism	Hypothyroidism

C03y.00	other specified congenital hypothyroidism	Hypothyroidism
C041.00	other postablative hypothyroidism	Hypothyroidism
C041000	irradiation hypothyroidism	Hypothyroidism
C041z00	postablative hypothyroidism nos	Hypothyroidism
C042.00	iodine hypothyroidism	Hypothyroidism
C043.00	other iatrogenic hypothyroidism	Hypothyroidism
C043000	hypothyroidism resulting from para-aminosalicylic acid	Hypothyroidism
C043100	hypothyroidism resulting from phenylbutazone	Hypothyroidism
C043200	hypothyroidism resulting from resorcinol	Hypothyroidism
C043z00	iatrogenic hypothyroidism nos	Hypothyroidism
C04y.00	other acquired hypothyroidism	Hypothyroidism
C04z000	premature puberty due to hypothyroidism	Hypothyroidism
F381400	myasthenic syndrome due to hypothyroidism	Hypothyroidism
Q433700	neonatal jaundice with congenital hypothyroidism	Hypothyroidism
C03z.12	cretinism	Hypothyroidism
C03y100	congenital hypothyroidism without goitre	Hypothyroidism
C04z.13	hypothyroid goitre, acquired	Hypothyroidism
C046.00	autoimmune myxoedema	Hypothyroidism
C045.00	acquired atrophy of thyroid	Hypothyroidism
C04z100	myxoedema coma	Hypothyroidism
C03z.00	congenital hypothyroidism nos	Hypothyroidism
C04z.00	hypothyroidism nos	Hypothyroidism
9Oj4.00	hypothyroidism monitoring telephone invitation	Hypothyroidism
Cyu1100	[x]other sp cified hypothyroidism	Hypothyroidism
C031.00	goitrous cretin	Hypothyroidism
C03y000	congenital hypothyroidism with diffuse goitre	Hypothyroidism
C04..11	myxoedema	Hypothyroidism
C03z.11	congenital thyroid insufficiency	Hypothyroidism
C03..11	cretinism	Hypothyroidism
66BB.00	hypothyroidism annual review	Hypothyroidism
C04..13	hypothyroidism	Hypothyroidism
F395300	myopathy due to myxoedema	Hypothyroidism
C047.00	subclinical hypothyroidism	Hypothyroidism
J615812	indian childhood cirrhosis	Liver disease
141F.00	history of viral hepatitis	Liver disease
J661100	chronic cholangitis	Liver disease
J622.00	hepatic coma	Liver disease
G850.00	oesophageal varices with bleeding	Liver disease
ZV02612	[v]hepatitis australia antigen carrier	Liver disease
J601100	subacute hepatitis - noninfective	Liver disease
A707300	chronic viral hepatitis b	Liver disease
J632000	hepatitis in malaria	Liver disease
J615711	congestive cirrhosis	Liver disease
A053.00	amoebic liver abscess	Liver disease
J612.11	florid cirrhosis	Liver disease
J614.00	chronic hepatitis	Liver disease

A916100	secondary syphilitic hepatitis	Liver disease
A72x000	mumps hepatitis	Liver disease
7Q05300	rsv treatment and hepatitis c treatment drugs band 1	Liver disease
A705z00	other specified viral hepatitis without mention of coma nos	Liver disease
760F300	rigid oesophagoscopic injection sclerotherapy oesoph varices	Liver disease
9kR..00	chronic hepatitis annual review - enhanced services admin	Liver disease
9kV..00	hepatitis c screening positive - enhanced services admin	Liver disease
9kX..00	hepatitis status 6 months post treatment - enhanced serv adm	Liver disease
9kZ..00	hepatitis b screening positive - enhanced services admin	Liver disease
C370800	cystic fibrosis related cirrhosis	Liver disease
J611.00	acute alcoholic hepatitis	Liver disease
4JQD.11	hepatitis c pcr positive	Liver disease
8LH..00	liver transplant planned	Liver disease
4JQD.00	hepatitis c viral ribonucleic acid pcr positive	Liver disease
J661200	recurrent cholangitis	Liver disease
43jJ.00	hepatitis d nucleic acid detection	Liver disease
4JQ3.00	hepatitis c virus genotype	Liver disease
43jG.00	hepatitis b nucleic acid detection	Liver disease
43j5.00	hepatitis c nucleic acid detection	Liver disease
C350012	pigmentary cirrhosis of liver	Liver disease
43k0.00	hepatitis b e antigen level	Liver disease
A709.00	viral hepatitis without hepatic coma	Liver disease
A707.00	chronic viral hepatitis	Liver disease
A70z100	acute viral hepatitis nos	Liver disease
43w7.00	hepatitis a nucleic acid detection	Liver disease
J615111	postnecrotic cirrhosis of liver	Liver disease
4JQF.00	hepatitis c antigen positive	Liver disease
J616100	secondary biliary cirrhosis	Liver disease
A70A.00	hepatitis c genotype 1	Liver disease
A70B.00	hepatitis c genotype 2	Liver disease
A70C.00	hepatitis c genotype 3	Liver disease
A70D.00	hepatitis c genotype 4	Liver disease
14i..00	h/o hepatitis c antiviral drug therapy	Liver disease
9NgR.00	on hepatitis c treatment plan	Liver disease
J63B.00	autoimmune hepatitis	Liver disease
J615.00	cirrhosis - non alcoholic	Liver disease
J622.11	encephalopathy - hepatic	Liver disease
67P4200	discussion about liver transplantation	Liver disease
A701.00	viral (infectious) hepatitis a	Liver disease
A701.11	infective hepatitis	Liver disease
7800112	piggy back liver transplant	Liver disease
J615A00	pipe-stem portal cirrhosis	Liver disease
G851.00	oesophageal varices without bleeding	Liver disease
ZV02C00	[v]hepatitis c carrier	Liver disease
J631100	hepatitis in cytomegalic inclusion virus	Liver disease
14C5.00	h/o: liver disease	Liver disease

Q434100	giant cell hepatitis causing neonatal jaundice	Liver disease
J615.11	portal cirrhosis	Liver disease
43B5.00	hepatitis e antigen present	Liver disease
J612.12	laennec's cirrhosis	Liver disease
43M2.00	hepatitis a test positive	Liver disease
J600200	acute yellow atrophy	Liver disease
65Q7.00	viral hepatitis carrier	Liver disease
7609.00	open operations on oesophageal varices	Liver disease
7609400	open injection sclerotherapy to oesophageal varices	Liver disease
7609z00	open operation on oesophageal varices nos	Liver disease
760C500	fibreoptic endoscopic banding of oesophageal varices	Liver disease
760F400	rigid oesophagoscopic banding of oesophageal varices	Liver disease
141E.00	history of hepatitis b	Liver disease
7800200	replacement of previous liver transplant	Liver disease
J61y800	nonalcoholic steatohepatitis	Liver disease
A700.00	viral hepatitis a with coma	Liver disease
A702.00	viral hepatitis b with coma	Liver disease
A704.00	other specified viral hepatitis with coma	Liver disease
A704000	viral hepatitis c with coma	Liver disease
A704z00	other specified viral hepatitis with hepatic coma nos	Liver disease
A705.00	other specified viral hepatitis without coma	Liver disease
A705000	viral hepatitis c without mention of hepatic coma	Liver disease
A707100	chronic viral hepatitis b without delta-agent	Liver disease
A70z.00	unspecified viral hepatitis	Liver disease
A785200	cytomegaloviral hepatitis	Liver disease
AD05.00	toxoplasma hepatitis	Liver disease
J632300	hepatitis in toxoplasmosis	Liver disease
AE23.00	sequelae of viral hepatitis	Liver disease
AyuB000	[x]other specified acute viral hepatitis	Liver disease
AyuB100	[x]other chronic viral hepatitis	Liver disease
AyuB200	[x]chronic viral hepatitis, unspecified	Liver disease
C376100	alpha-1-antitrypsin hepatitis	Liver disease
G852.00	oesophageal varices in diseases ec	Liver disease
G852000	oesophageal varices with bleeding in diseases ec	Liver disease
G852100	oesophageal varices without bleeding in diseases ec	Liver disease
G852z00	oesophageal varices in diseases ec nos	Liver disease
Gyu9400	[x]oesophageal varices in diseases classified elsewhere	Liver disease
J600011	acute liver failure	Liver disease
J601200	subacute yellow atrophy	Liver disease
J61..00	cirrhosis and chronic liver disease	Liver disease
J614100	chronic active hepatitis	Liver disease
J614111	autoimmune chronic active hepatitis	Liver disease
J614300	recurrent hepatitis	Liver disease
J614y00	chronic hepatitis unspecified	Liver disease
J614z00	chronic hepatitis nos	Liver disease
J615000	unilobular portal cirrhosis	Liver disease

J615300	diffuse nodular cirrhosis	Liver disease
J615400	fatty portal cirrhosis	Liver disease
J615600	capsular portal cirrhosis	Liver disease
J615B00	toxic portal cirrhosis	Liver disease
J615D00	bacterial portal cirrhosis	Liver disease
J615F00	syphilitic portal cirrhosis	Liver disease
J615G00	zooparasitic portal cirrhosis	Liver disease
J615H00	infectious cirrhosis nos	Liver disease
J615y00	portal cirrhosis unspecified	Liver disease
J616200	biliary cirrhosis of children	Liver disease
J616z00	biliary cirrhosis nos	Liver disease
J61y.00	other non-alcoholic chronic liver disease	Liver disease
J61y300	portal fibrosis without cirrhosis	Liver disease
J61yz00	other non-alcoholic chronic liver disease nos	Liver disease
J61z.00	chronic liver disease nos	Liver disease
J62..00	liver abscess and sequelae of chronic liver disease	Liver disease
J620.00	liver abscess - excluding amoebic liver abscess	Liver disease
J620000	liver abscess due to portal pyaemia	Liver disease
J620100	liver abscess due to cholangitis	Liver disease
J620200	liver abscess via hepatic artery	Liver disease
J620300	liver abscess via umbilicus	Liver disease
J620z00	liver abscess nos	Liver disease
J62z.00	liver abscess and chronic liver disease causing sequelae nos	Liver disease
J631.00	hepatitis in viral diseases ec	Liver disease
J631000	hepatitis in coxsackie virus	Liver disease
J631400	hepatitis in yellow fever	Liver disease
J631500	hepatitis in other viral disease	Liver disease
J631z00	hepatitis in viral diseases ec nos	Liver disease
J632.00	hepatitis in other infectious diseases ec	Liver disease
J632200	hepatitis in secondary syphilis	Liver disease
J632z00	hepatitis in infectious diseases ec nos	Liver disease
J633.00	hepatitis unspecified	Liver disease
J633000	toxic hepatitis	Liver disease
J633z00	hepatitis unspecified nos	Liver disease
J635.00	toxic liver disease	Liver disease
J635000	toxic liver disease with cholestasis	Liver disease
J635100	toxic liver disease with hepatic necrosis	Liver disease
J635200	toxic liver disease with acute hepatitis	Liver disease
J635300	toxic liver disease with chronic persistent hepatitis	Liver disease
J635400	toxic liver disease with chronic lobular hepatitis	Liver disease
J635500	toxic liver disease with chronic active hepatitis	Liver disease
J635600	toxic liver disease with fibrosis and cirrhosis of liver	Liver disease
9Op1.00	hepatitis c screening offered	Liver disease
J661600	obliterative cholangitis	Liver disease
J661700	primary sclerosing cholangitis	Liver disease
J661800	secondary sclerosing cholangitis	Liver disease

J661900	sclerosing cholangitis unspecified	Liver disease
Jyu7100	[x]other and unspecified cirrhosis of liver	Liver disease
Jyu7200	[x]other specified inflammatory liver diseases	Liver disease
Jyu7600	[x]toxic liver disease, unspecified	Liver disease
Jyu7700	[x]granulomatous hepatitis, not elsewhere classified	Liver disease
BB5D411	[m]hepatocellular adenoma	Liver disease
43jW.00	hepatitis e nucleic acid detection	Liver disease
PB62z00	congenital cystic liver disease nos	Liver disease
Q409.00	congenital viral hepatitis	Liver disease
Q409000	congenital hepatitis a infection	Liver disease
Q409z00	congenital viral hepatitis nos	Liver disease
Q434000	perinatal hepatitis causing jaundice, unspecified	Liver disease
SP08600	liver transplant failure and rejection	Liver disease
SP14211	liver failure as a complication of care	Liver disease
SP14300	hepatorenal syndrome as a complication of care	Liver disease
7Q05200	hepatitis b treatment drugs band 1	Liver disease
7805211	exploration of liver transplant	Liver disease
J63y100	nonspecific reactive hepatitis	Liver disease
A98yy11	gonococcal hepatitis	Liver disease
A705100	acute delta-(super)infection of hepatitis b carrier	Liver disease
A70G.00	acute hepatitis c	Liver disease
A705200	acute hepatitis e	Liver disease
A707000	chronic viral hepatitis b with delta-agent	Liver disease
J617.00	alcoholic hepatitis	Liver disease
J62y.12	liver failure nos	Liver disease
J615z13	cirrhosis of liver nos	Liver disease
J615z14	laennec's cirrhosis, non-alcoholic	Liver disease
A98yy13	gonococcal perihepatitis	Liver disease
J625.11	[x] liver failure	Liver disease
7609y00	other specified open operation on oesophageal varices	Liver disease
J615100	multilobular portal cirrhosis	Liver disease
J615700	cardiac portal cirrhosis	Liver disease
J615800	juvenile portal cirrhosis	Liver disease
J615z00	non-alcoholic cirrhosis nos	Liver disease
J62y.00	other sequelae of chronic liver disease	Liver disease
J616.00	biliary cirrhosis	Liver disease
65V3.11	hepatitis notification	Liver disease
2J2..00	hepatitis a status	Liver disease
AyuJ900	[x]sequelae of viral hepatitis	Liver disease
AyuB.00	[x]viral hepatitis	Liver disease
J617000	chronic alcoholic hepatitis	Liver disease
G852200	oesophageal varices in cirrhosis of the liver	Liver disease
G852300	oesophageal varices in alcoholic cirrhosis of the liver	Liver disease
2J23.00	hepatitis a - current infection	Liver disease
G858.00	oesophageal varices nos	Liver disease
J600100	acute hepatitis - noninfective	Liver disease

ZV02B00	[v]hepatitis b carrier	Liver disease
ZV02600	[v]viral hepatitis carrier	Liver disease
ZV42700	[v]liver transplanted	Liver disease
ZV7C000	[v]assessment for liver transplant	Liver disease
J612.00	alcoholic cirrhosis of liver	Liver disease
G85..11	oesophageal varices	Liver disease
J615500	hypertrophic portal cirrhosis	Liver disease
7800111	auxillary liver transplant	Liver disease
PB62000	congenital polycystic liver disease	Liver disease
PB62100	fibrocystic liver disease	Liver disease
J616000	primary biliary cirrhosis	Liver disease
7L1f.00	compensation for liver failure	Liver disease
7L1fz00	compensation for liver failure nos	Liver disease
J614200	chronic aggressive hepatitis	Liver disease
A707200	chronic viral hepatitis c	Liver disease
A707X00	chronic viral hepatitis, unspecified	Liver disease
J615C00	xanthomatous portal cirrhosis	Liver disease
J623.00	portal hypertension	Liver disease
PB62.00	congenital cystic liver disease	Liver disease
Q409100	congenital hepatitis b infection	Liver disease
J615z12	cryptogenic cirrhosis of liver	Liver disease
A703.00	viral (serum) hepatitis b	Liver disease
L176500	viral hepatitis comp pregnancy, childbirth & the puerperium	Liver disease
9kZ..11	hepatitis b screening positive	Liver disease
9kV..11	hepatitis c screening positive	Liver disease
J614000	chronic persistent hepatitis	Liver disease
760C300	fibreoptic endoscopic injection sclerotherapy oesoph varices	Liver disease
A70..00	viral hepatitis	Liver disease
J615z11	macronodular cirrhosis of liver	Liver disease
7609300	local ligation of oesophageal varices	Liver disease
TB00200	liver transplant with complication, without blame	Liver disease
C310400	glycogenosis with hepatic cirrhosis	Liver disease
J63X.00	granulomatous hepatitis, not elsewhere classified	Liver disease
J631600	hepatitis + adenovirus	Liver disease
43B4.00	hepatitis b surface antig +ve	Liver disease
J631200	hepatitis in infectious mononucleosis	Liver disease
A705400	hepatitis non a non b	Liver disease
A70z000	hepatitis c	Liver disease
J624.00	hepatorenal syndrome	Liver disease
J614400	chronic lobular hepatitis	Liver disease
J635X00	toxic liver disease, unspecified	Liver disease
573 B	hepatic coma	Liver disease
5719PH	portal hypertension	Liver disease
ZC2CH11	dietary advice for liver disease	Liver disease
G310.00	postmyocardial infarction syndrome	Myocardial infarction
G307.00	acute subendocardial infarction	Myocardial infarction

G35X.00	subsequent myocardial infarction of unspecified site	Myocardial infarction
G303.00	acute inferoposterior infarction	Myocardial infarction
14AT.00	history of myocardial infarction	Myocardial infarction
G30X000	acute st segment elevation myocardial infarction	Myocardial infarction
G307100	acute non-st segment elevation myocardial infarction	Myocardial infarction
G30..12	coronary thrombosis	Myocardial infarction
G30..16	thrombosis - coronary	Myocardial infarction
G38..00	postoperative myocardial infarction	Myocardial infarction
G380.00	postoperative transmural myocardial infarction anterior wall	Myocardial infarction
G381.00	postoperative transmural myocardial infarction inferior wall	Myocardial infarction
G383.00	postoperative transmural myocardial infarction unspec site	Myocardial infarction
G30..15	mi - acute myocardial infarction	Myocardial infarction
G32..12	personal history of myocardial infarction	Myocardial infarction
7929111	percut translum coronary thrombolytic therapy- streptokinase	Myocardial infarction
7929100	percut transluminal coronary thrombolysis with streptokinase	Myocardial infarction
323..00	ecg: myocardial infarction	Myocardial infarction
3232.00	ecg: old myocardial infarction	Myocardial infarction
323Z.00	ecg: myocardial infarct nos	Myocardial infarction
G311011	mi - myocardial infarction aborted	Myocardial infarction
G301.00	other specified anterior myocardial infarction	Myocardial infarction
G301z00	anterior myocardial infarction nos	Myocardial infarction
G304.00	posterior myocardial infarction nos	Myocardial infarction
G305.00	lateral myocardial infarction nos	Myocardial infarction
G306.00	true posterior myocardial infarction	Myocardial infarction
G308.00	inferior myocardial infarction nos	Myocardial infarction
G30y.00	other acute myocardial infarction	Myocardial infarction
G30yz00	other acute myocardial infarction nos	Myocardial infarction
G30z.00	acute myocardial infarction nos	Myocardial infarction
G35..00	subsequent myocardial infarction	Myocardial infarction
G350.00	subsequent myocardial infarction of anterior wall	Myocardial infarction
G351.00	subsequent myocardial infarction of inferior wall	Myocardial infarction
G353.00	subsequent myocardial infarction of other sites	Myocardial infarction
Gyu3500	[x]subsequent myocardial infarction of other sites	Myocardial infarction
Gyu3600	[x]subsequent myocardial infarction of unspecified site	Myocardial infarction
G344.00	silent myocardial ischaemia	Myocardial infarction
G30..17	silent myocardial infarction	Myocardial infarction
Gyu3400	[x]acute transmural myocardial infarction of unspecif site	Myocardial infarction
G30..14	heart attack	Myocardial infarction
G32..00	old myocardial infarction	Myocardial infarction
G32..11	healed myocardial infarction	Myocardial infarction
G307000	acute non-q wave infarction	Myocardial infarction
14AH.00	h/o: myocardial infarction in last year	Myocardial infarction
G384.00	postoperative subendocardial myocardial infarction	Myocardial infarction
G38z.00	postoperative myocardial infarction, unspecified	Myocardial infarction
G300.00	acute anterolateral infarction	Myocardial infarction
G301100	acute anteroseptal infarction	Myocardial infarction

G302.00	acute inferolateral infarction	Myocardial infarction
G30X.00	acute transmural myocardial infarction of unspecif site	Myocardial infarction
G30..13	cardiac rupture following myocardial infarction (mi)	Myocardial infarction
G36..00	certain current complication follow acute myocardial infarct	Myocardial infarction
889A.00	diab mellit insulin-glucose infus acute myocardial infarct	Myocardial infarction
G311000	myocardial infarction aborted	Myocardial infarction
14A3.00	h/o: myocardial infarct <60	Myocardial infarction
14A4.00	h/o: myocardial infarct >60	Myocardial infarction
G30..00	acute myocardial infarction	Myocardial infarction
G30B.00	acute posterolateral myocardial infarction	Myocardial infarction
429 AH	heart attack	Myocardial infarction
F251011	tonic-clonic epilepsy	Neurological disorders
F150.11	infantile spinal muscular atrophy	Neurological disorders
F250y00	other specified generalised nonconvulsive epilepsy	Neurological disorders
F135000	hemiballismus	Neurological disorders
F25A.00	juvenile myoclonic epilepsy	Neurological disorders
147F.00	history of parkinson's disease	Neurological disorders
8IAb.00	multiple sclerosis review declined	Neurological disorders
R003400	[d]nocturnal seizure	Neurological disorders
67IJ000	pre-conception advice for patients with epilepsy	Neurological disorders
67AF.00	pregnancy advice for patients with epilepsy	Neurological disorders
8IAg.00	contraceptive advice for patients with epilepsy declined	Neurological disorders
8IAh.00	pre-conception advice for patients with epilepsy declined	Neurological disorders
8IAi.00	pregnancy advice for patients with epilepsy declined	Neurological disorders
P235.00	x-linked hydrocephalus	Neurological disorders
R003z11	[d]seizure nos	Neurological disorders
F21y211	binswanger's encephalopathy	Neurological disorders
F25X.00	status epilepticus, unspecified	Neurological disorders
F252.00	petit mal status	Neurological disorders
F152300	pseudobulbar palsy	Neurological disorders
F040111	cerebellar abscess	Neurological disorders
P100z00	spina bifida with hydrocephalus nos	Neurological disorders
P110000	spina bifida without hydrocephalus, site unspecified	Neurological disorders
F114.00	acquired obstructive hydrocephalus	Neurological disorders
667K.00	epilepsy limits activities	Neurological disorders
9h6..00	exception reporting: epilepsy quality indicators	Neurological disorders
F151200	adult spinal muscular atrophy	Neurological disorders
667G.00	epilepsy restricts employment	Neurological disorders
667H.00	epilepsy prevents employment	Neurological disorders
F15..00	anterior horn cell disease	Neurological disorders
667M.00	epilepsy management plan given	Neurological disorders
F152000	amyotrophic lateral sclerosis	Neurological disorders
F152100	progressive muscular atrophy	Neurological disorders
F25y100	gelastic epilepsy	Neurological disorders
F255100	sensory induced epilepsy	Neurological disorders
1B1W.00	transient epileptic amnesia	Neurological disorders

F21y300	central demyelination of corpus callosum	Neurological disorders
F14..00	spinocerebellar disease	Neurological disorders
F12X.00	secondary parkinsonism, unspecified	Neurological disorders
667J.00	epilepsy impairs education	Neurological disorders
9mD0.00	multiple sclerosis monitoring first letter	Neurological disorders
9mD1.00	multiple sclerosis monitoring second letter	Neurological disorders
F207.00	relapsing and remitting multiple sclerosis	Neurological disorders
G021.00	rheumatic chorea without mention of heart involvement	Neurological disorders
8IB2.00	contraceptiv advice for patients with epilepsy not indicated	Neurological disorders
8Hkv.00	referral to community multiple sclerosis team	Neurological disorders
8BIF.00	epilepsy medication review	Neurological disorders
667E.00	epilepsy care arrangement	Neurological disorders
F25..00	epilepsy	Neurological disorders
F140.00	friedreich's ataxia	Neurological disorders
F29y300	toxic encephalopathy	Neurological disorders
F251600	grand mal seizure	Neurological disorders
667N.00	epilepsy severity	Neurological disorders
667Q.00	1 to 12 seizures a year	Neurological disorders
667R.00	2 to 4 seizures a month	Neurological disorders
667S.00	1 to 7 seizures a week	Neurological disorders
667T.00	daily seizures	Neurological disorders
667V.00	many seizures a day	Neurological disorders
667W.00	emergency epilepsy treatment since last appointment	Neurological disorders
1O30.00	epilepsy confirmed	Neurological disorders
F254500	complex partial epileptic seizure	Neurological disorders
F11X.00	post-traumatic hydrocephalus, unspecified	Neurological disorders
J622.11	encephalopathy - hepatic	Neurological disorders
F251000	grand mal (major) epilepsy	Neurological disorders
F250000	petit mal (minor) epilepsy	Neurological disorders
F253.00	grand mal status	Neurological disorders
F253.11	status epilepticus	Neurological disorders
F121.00	parkinsonism secondary to drugs	Neurological disorders
F135100	paroxysmal choreo-athetosis	Neurological disorders
F255.00	partial epilepsy without impairment of consciousness	Neurological disorders
F255z00	partial epilepsy without impairment of consciousness nos	Neurological disorders
F255y00	partial epilepsy without impairment of consciousness os	Neurological disorders
F25E.00	stress-induced epilepsy	Neurological disorders
SC20000	traumatic epilepsy	Neurological disorders
1473.00	h/o: epilepsy	Neurological disorders
F251111	otohara syndrome	Neurological disorders
9Of3.00	epilepsy monitoring verbal invite	Neurological disorders
297A.00	o/e - parkinsonian tremor	Neurological disorders
2987.00	o/e -parkinson flexion posture	Neurological disorders
F250.00	generalised nonconvulsive epilepsy	Neurological disorders
667..00	epilepsy monitoring	Neurological disorders
6672.00	follow-up epilepsy assessment	Neurological disorders

6674.00	epilepsy associated problems	Neurological disorders
6677.00	epilepsy drug side effects	Neurological disorders
6678.00	epilepsy treatment changed	Neurological disorders
6679.00	epilepsy treatment started	Neurological disorders
667A.00	epilepsy treatment stopped	Neurological disorders
667Z.00	epilepsy monitoring nos	Neurological disorders
F208.00	secondary progressive multiple sclerosis	Neurological disorders
F206.00	primary progressive multiple sclerosis	Neurological disorders
P22z.11	cerebellar hypoplasia	Neurological disorders
2987.11	o/e - parkinson posture	Neurological disorders
F037.00	transverse myelitis	Neurological disorders
F1y0.00	fragile x associated tremor ataxia syndrome	Neurological disorders
BBbW.00	[m]cerebellar sarcoma nos	Neurological disorders
F103100	cerebral degeneration in mucopolysaccharidoses	Neurological disorders
F10z.00	childhood cerebral degenerations nos	Neurological disorders
F113.00	acquired communicating hydrocephalus	Neurological disorders
F113z00	communicating hydrocephalus - acquired nos	Neurological disorders
F12z.00	parkinson's disease nos	Neurological disorders
F130300	parkinsonism with orthostatic hypotension	Neurological disorders
F132200	myoclonic encephalopathy	Neurological disorders
F135.00	other choreas	Neurological disorders
F135z00	other choreas nos	Neurological disorders
F142200	dyssynergia cerebellaris myoclonica	Neurological disorders
F142z00	primary cerebellar degeneration nos	Neurological disorders
F144.00	cerebellar ataxia in diseases ec	Neurological disorders
F144200	cerebellar ataxia due to neoplasia	Neurological disorders
F144z00	cerebellar ataxia in disease nos	Neurological disorders
F14y.00	other spinocerebellar diseases	Neurological disorders
F14yz00	other spinocerebellar disease nos	Neurological disorders
F14z.00	spinocerebellar disease nos	Neurological disorders
F151000	unspecified spinal muscular atrophy	Neurological disorders
F151z00	spinal muscular atrophy nos	Neurological disorders
F15y.00	other anterior horn cell disease	Neurological disorders
F15z.00	anterior horn cell disease nos	Neurological disorders
F201.00	multiple sclerosis of the spinal cord	Neurological disorders
F202.00	generalised multiple sclerosis	Neurological disorders
F203.00	exacerbation of multiple sclerosis	Neurological disorders
F20z.00	multiple sclerosis nos	Neurological disorders
F21..00	other central nervous system demyelinating diseases	Neurological disorders
F21y.00	other specified central nervous system demyelinating disease	Neurological disorders
F21yz00	other specified central nervous system demyelination nos	Neurological disorders
F21z.00	central nervous system demyelination nos	Neurological disorders
F250200	epileptic seizures - atonic	Neurological disorders
F250300	epileptic seizures - akinetic	Neurological disorders
F251100	neonatal myoclonic epilepsy	Neurological disorders
F251200	epileptic seizures - clonic	Neurological disorders

F251300	epileptic seizures - myoclonic	Neurological disorders
F251400	epileptic seizures - tonic	Neurological disorders
F251y00	other specified generalised convulsive epilepsy	Neurological disorders
F251z00	generalised convulsive epilepsy nos	Neurological disorders
F254.00	partial epilepsy with impairment of consciousness	Neurological disorders
F254000	temporal lobe epilepsy	Neurological disorders
F254200	psychosensory epilepsy	Neurological disorders
F254400	epileptic automatism	Neurological disorders
F254z00	partial epilepsy with impairment of consciousness nos	Neurological disorders
F255200	somatosensory epilepsy	Neurological disorders
F255311	partial epilepsy with autonomic symptoms	Neurological disorders
F255300	visceral reflex epilepsy	Neurological disorders
F255400	visual reflex epilepsy	Neurological disorders
F255500	unilateral epilepsy	Neurological disorders
F256z00	infantile spasms nos	Neurological disorders
F257.00	kojevnikov's epilepsy	Neurological disorders
F25y.00	other forms of epilepsy	Neurological disorders
F25y000	cursive (running) epilepsy	Neurological disorders
F25yz00	other forms of epilepsy nos	Neurological disorders
F283.00	unspecified encephalopathy	Neurological disorders
F365.00	neuropathy in association with hereditary ataxia	Neurological disorders
666A.00	multiple sclerosis review	Neurological disorders
Fyu1000	[x]other hereditary ataxias	Neurological disorders
Fyu2000	[x]other drug-induced secondary parkinsonism	Neurological disorders
Fyu2100	[x]other secondary parkinsonism	Neurological disorders
Fyu2200	[x]parkinsonism in diseases classified elsewhere	Neurological disorders
Fyu2600	[x]other chorea	Neurological disorders
Fyu2900	[x]secondary parkinsonism, unspecified	Neurological disorders
Fyu4.00	[x]demyelinating diseases of the central nervous system	Neurological disorders
Fyu4000	[x]other specified acute disseminated demyelination	Neurological disorders
Fyu4200	[x]acute disseminated demyelination, unspecified	Neurological disorders
Fyu5000	[x]other generalized epilepsy and epileptic syndromes	Neurological disorders
Fyu5100	[x]other epilepsy	Neurological disorders
Fyu5200	[x]other status epilepticus	Neurological disorders
Fyu5900	[x]status epilepticus, unspecified	Neurological disorders
FyuA100	[x]other hydrocephalus	Neurological disorders
FyuA400	[x]hydrocephalus in neoplastic disease classified elsewhere	Neurological disorders
FyuAG00	[x]post-traumatic hydrocephalus, unspecified	Neurological disorders
G02z.00	rheumatic chorea nos	Neurological disorders
8CS1.00	multiple sclerosis care plan agreed	Neurological disorders
F255600	simple partial epileptic seizure	Neurological disorders
9Of5.00	epilepsy monitoring call first letter	Neurological disorders
9Of6.00	epilepsy monitoring call second letter	Neurological disorders
P100.00	unspecified spina bifida with hydrocephalus	Neurological disorders
P100000	spina bifida with hydrocephalus, unspecified	Neurological disorders
P100100	cervical spina bifida with hydrocephalus	Neurological disorders

P100200	thoracic spina bifida with hydrocephalus	Neurological disorders
P100300	lumbar spina bifida with hydrocephalus	Neurological disorders
P102200	thoracic spina bifida with hydrocephalus - open	Neurological disorders
P102300	lumbar spina bifida with hydrocephalus - open	Neurological disorders
P102400	sacral spina bifida with hydrocephalus - open	Neurological disorders
P102z00	spina bifida with hydrocephalus - open nos	Neurological disorders
P103.00	spina bifida with hydrocephalus - closed	Neurological disorders
P103300	lumbar spina bifida with hydrocephalus - closed	Neurological disorders
P103400	sacral spina bifida with hydrocephalus - closed	Neurological disorders
P104.00	spina bifida with hydrocephalus of late onset	Neurological disorders
P10y.00	other specified spina bifida with hydrocephalus	Neurological disorders
P10z.00	spina bifida with hydrocephalus nos	Neurological disorders
P11..00	spina bifida without mention of hydrocephalus	Neurological disorders
P110100	cervical spina bifida without mention of hydrocephalus	Neurological disorders
P110200	thoracic spina bifida without mention of hydrocephalus	Neurological disorders
P110300	lumbar spina bifida without mention of hydrocephalus	Neurological disorders
P117200	thoracic spina bifida without hydrocephalus - open	Neurological disorders
P117300	lumbar spina bifida without hydrocephalus - open	Neurological disorders
P117400	sacral spina bifida without hydrocephalus - open	Neurological disorders
P117z00	spina bifida without hydrocephalus - open nos	Neurological disorders
P118.00	spina bifida without hydrocephalus - closed	Neurological disorders
P118000	unspecified spina bifida without hydrocephalus - closed	Neurological disorders
P118100	cervical spina bifida without hydrocephalus - closed	Neurological disorders
P118300	lumbar spina bifida without hydrocephalus - closed	Neurological disorders
P118400	sacral spina bifida without hydrocephalus - closed	Neurological disorders
P23z.00	congenital hydrocephalus nos	Neurological disorders
Pyu0100	[x]other congenital hydrocephalus	Neurological disorders
Pyu0400	[x]unspecified spina bifida with hydrocephalus	Neurological disorders
666B.00	multiple sclerosis multidisciplinary review	Neurological disorders
R003300	[d]reflex anoxic seizure	Neurological disorders
R013000	[d]ataxia nos	Neurological disorders
SL6yz00	antiparkinsonian drug poisoning nos	Neurological disorders
SL6z.00	anticonvulsant or antiparkinsonian drug poisoning nos	Neurological disorders
TJ64z00	adverse reaction to antiparkinsonism drugs nos	Neurological disorders
F123.00	postencephalitic parkinsonism	Neurological disorders
F037000	varicella transverse myelitis	Neurological disorders
F146.00	early onset cerebellar ataxia with hypogonadism	Neurological disorders
F135200	drug-induced chorea	Neurological disorders
F255011	focal epilepsy	Neurological disorders
F250400	juvenile absence epilepsy	Neurological disorders
F250500	lennox-gastaut syndrome	Neurological disorders
F259.11	ohtahara syndrome	Neurological disorders
F25G.00	severe myoclonic epilepsy in infancy	Neurological disorders
F25C.00	drug-induced epilepsy	Neurological disorders
F25D.00	menstrual epilepsy	Neurological disorders
667B.00	nocturnal epilepsy	Neurological disorders

F25y300	complex partial status epilepticus	Neurological disorders
F115.00	hydrocephalus	Neurological disorders
U606719	[x] adverse react to anticonvuls or antiparkinson drug nos	Neurological disorders
U606.11	[x] adverse react to anticonvulsants & anti-parkinsons drugs	Neurological disorders
U606711	[x] adverse reaction to antiparkinsonism drug	Neurological disorders
U606718	[x] adverse reaction to antiparkinsonism drugs nos	Neurological disorders
7Q04100	amyotrophic lateral sclerosis drugs band 1	Neurological disorders
C253.00	wernicke's encephalopathy	Neurological disorders
Eu80300	[x]acquired aphasia with epilepsy [landau - kleffner]	Neurological disorders
U606.00	[x]antiepilept + antiparksn drug caus advers eff therap use	Neurological disorders
U606700	[x]antiparkinsonism drugs caus advers effects in therap use	Neurological disorders
F25H.00	generalised seizure	Neurological disorders
A413.11	progressive multifocal leukoencephalopathy	Neurological disorders
Eu02400	[x]dementia in human immunodef virus [hiv] disease	Neurological disorders
P102.14	rachischisis with hydrocephalus	Neurological disorders
P102.13	myelocele with hydrocephalus	Neurological disorders
P102.11	fissured spine with hydrocephalus	Neurological disorders
P103z11	thoracolumbar spina bifida with hydrocephalus - closed	Neurological disorders
Eu05y11	[x]epileptic psychosis nos	Neurological disorders
Eu06013	[x]limbic epilepsy personality	Neurological disorders
F142.00	primary cerebellar degeneration	Neurological disorders
F132100	progressive myoclonic epilepsy	Neurological disorders
F143.00	cerebellar ataxia nos	Neurological disorders
F25z.00	epilepsy nos	Neurological disorders
P102.00	spina bifida with hydrocephalus - open	Neurological disorders
P117.00	spina bifida without hydrocephalus - open	Neurological disorders
P118z00	spina bifida without hydrocephalus - closed nos	Neurological disorders
P11y.00	other specified spina bifida without hydrocephalus	Neurological disorders
P11z.00	spina bifida without mention of hydrocephalus nos	Neurological disorders
2994.00	o/e-festination-parkinson gait	Neurological disorders
U606600	[x]oth unspec antiepileptics caus adverse eff in therap use	Neurological disorders
9Of4.00	epilepsy monitoring telephone invite	Neurological disorders
F20..00	multiple sclerosis	Neurological disorders
Fyu4100	[x]other specified demyelinating diseases/the cns	Neurological disorders
F210.00	neuromyelitis optica	Neurological disorders
Q402311	congenital hydrocephalus due to toxoplasmosis	Neurological disorders
F255012	motor epilepsy	Neurological disorders
F255000	jacksonian, focal or motor epilepsy	Neurological disorders
F25z.11	fit (in known epileptic) nos	Neurological disorders
F21X.00	acute disseminated demyelination, unspecified	Neurological disorders
667C.00	epilepsy control good	Neurological disorders
667D.00	epilepsy control poor	Neurological disorders
TJ64.00	adverse reaction to antiparkinsonism drugs	Neurological disorders
F256.12	west syndrome	Neurological disorders
F256.00	infantile spasms	Neurological disorders
F256000	hypsarrhythmia	Neurological disorders

F256100	salaam attacks	Neurological disorders
F256.11	lightning spasms	Neurological disorders
F251500	tonic-clonic epilepsy	Neurological disorders
F254100	psychomotor epilepsy	Neurological disorders
F144000	cerebellar ataxia due to alcoholism	Neurological disorders
F11y100	cerebral ataxia	Neurological disorders
A413.00	progressive multifocal leucoencephalopathy	Neurological disorders
F113011	low pressure hydrocephalus	Neurological disorders
F258.00	post-ictal state	Neurological disorders
F24y000	progressive supranuclear palsy	Neurological disorders
F281.11	anoxic - ischaemic encephalopathy	Neurological disorders
F132z12	myoclonic seizure	Neurological disorders
SL6y.00	antiparkinsonism drug poisoning	Neurological disorders
F250100	pykno-epilepsy	Neurological disorders
F14y000	ataxia-telangiectasia	Neurological disorders
F251.00	generalised convulsive epilepsy	Neurological disorders
F374100	polyneuropathy in beriberi	Neurological disorders
2828.00	absence seizure	Neurological disorders
F25F.00	photosensitive epilepsy	Neurological disorders
F113000	normal pressure hydrocephalus	Neurological disorders
Q417100	cerebellar (nontraum) and post fossa haemorhage fet newborn	Neurological disorders
F14..11	cerebellar disease	Neurological disorders
F040100	cerebellar intracranial abscess	Neurological disorders
C251.11	wernicke's encephalopathy	Neurological disorders
C250.00	beriberi	Neurological disorders
F121.11	drug induced parkinsonism	Neurological disorders
F152111	duchenne aran muscular atrophy	Neurological disorders
F25y500	panayiotopoulos syndrome	Neurological disorders
F204.00	benign multiple sclerosis	Neurological disorders
F250011	epileptic absences	Neurological disorders
A94y100	syphilitic parkinsonism	Neurological disorders
F13..11	extrapyramidal disease excluding parkinson's disease	Neurological disorders
F200.00	multiple sclerosis of the brain stem	Neurological disorders
8Cc0.00	management of multiple sclerosis in onset phase	Neurological disorders
8Cc1.00	management of multiple sclerosis in early disease phase	Neurological disorders
8Cc2.00	management of multiple sclerosis in stable disability phase	Neurological disorders
8Cc4.00	management of multiple sclerosis in palliative phase	Neurological disorders
F14y100	corticostriatal-spinal degeneration	Neurological disorders
F25y400	benign rolandic epilepsy	Neurological disorders
F25y200	locl-rlt(foc)(part)idiop epilep&epilptic syn seiz locl onset	Neurological disorders
A940.11	locomotor ataxia	Neurological disorders
F254300	limbic system epilepsy	Neurological disorders
F142000	marie's cerebellar ataxia	Neurological disorders
G02..00	rheumatic chorea	Neurological disorders
G02..11	sydenham's chorea	Neurological disorders
P23..00	congenital hydrocephalus	Neurological disorders

P110z00	unspecified spina bifida without hydrocephalus nos	Neurological disorders
F250z00	generalised nonconvulsive epilepsy nos	Neurological disorders
F12..00	parkinson's disease	Neurological disorders
F120.00	paralysis agitans	Neurological disorders
F211.00	schilder's disease	Neurological disorders
P233.12	hydrocephalus with atresia of foramina of magendie+luschka	Neurological disorders
Q437000	bilirubin encephalopathy	Neurological disorders
G672.00	hypertensive encephalopathy	Neurological disorders
F361.00	peroneal muscular atrophy	Neurological disorders
P10..00	spina bifida with hydrocephalus	Neurological disorders
F151111	juvenile spinal muscular atrophy	Neurological disorders
F03..13	transverse myelitis	Neurological disorders
C315100	mitochond encephalopathy, lact acidosis & strokelike episode	Neurological disorders
667F.00	seizure free >12 months	Neurological disorders
F259.00	early infant epileptic encephalopathy wth suppression bursts	Neurological disorders
G020.00	rheumatic chorea with heart involvement	Neurological disorders
F134.00	huntington's chorea	Neurological disorders
9kG..00	spec serv for pat with multiple sclerosis - enh serv admin	Neurological disorders
F151.00	spinal muscular atrophy	Neurological disorders
Z7E4400	cerebellar ataxia	Neurological disorders
Z7E4.00	ataxia	Neurological disorders
Z7E4300	truncal ataxia	Neurological disorders
ZS42400	cerebellar dysarthria	Neurological disorders
ZS82.00	acquired epileptic aphasia	Neurological disorders
ZS42111	pseudobulbar palsy type of dysarthria	Neurological disorders
ZS42113	suprabulbar palsy type of dysarthria	Neurological disorders
F241.00	paraplegia	Paralysis or paresis
F36y000	supranuclear paralysis	Paralysis or paresis
F232.11	tetraplegia - congenital	Paralysis or paresis
F137000	athetoid cerebral palsy	Paralysis or paresis
Q206111	erb-duchenne paralysis	Paralysis or paresis
Q206112	erb's palsy	Paralysis or paresis
F221.00	spastic hemiplegia	Paralysis or paresis
F243.00	monoplegia of lower limb	Paralysis or paresis
F220.00	flaccid hemiplegia	Paralysis or paresis
7L0D115	sever operation for erb's palsy	Paralysis or paresis
E201400	hysterical paralysis	Paralysis or paresis
2949.00	lower limb spasticity	Paralysis or paresis
294A.00	upper limb spasticity	Paralysis or paresis
294B.00	worsening limb spasticity	Paralysis or paresis
294E.00	upper limb flaccidity	Paralysis or paresis
7L0D114	l'episcopo operation for obstetric palsy	Paralysis or paresis
F230111	spastic diplegic cerebral palsy	Paralysis or paresis
F2B0.00	spastic quadriplegic cerebral palsy	Paralysis or paresis
F2By.00	other cerebral palsy	Paralysis or paresis
F2Bz.00	cerebral palsy nos	Paralysis or paresis

38Gw.00	gross motor function classification system cerebral palsy	Paralysis or paresis
F240.00	quadriplegia	Paralysis or paresis
F240.11	tetraplegia	Paralysis or paresis
E260000	psychogenic paralysis	Paralysis or paresis
F2B..00	cerebral palsy	Paralysis or paresis
F2B1.00	spastic hemiplegic cerebral palsy	Paralysis or paresis
F230.11	paraplegia - congenital	Paralysis or paresis
F23y600	choreoathetoid cerebral palsy	Paralysis or paresis
F23y400	ataxic diplegic cerebral palsy	Paralysis or paresis
SN48111	paralysis following electric shock	Paralysis or paresis
1B33.00	paralysis present	Paralysis or paresis
283..00	o/e - paralysis	Paralysis or paresis
2833.00	o/e - hemiplegia	Paralysis or paresis
2834.00	o/e - monoplegia	Paralysis or paresis
2835.00	o/e - paraplegia	Paralysis or paresis
2836.00	o/e - quadriplegia	Paralysis or paresis
2837.00	o/e - diplegia	Paralysis or paresis
283Z.00	o/e - paralysis nos	Paralysis or paresis
2992.00	o/e - gait spastic	Paralysis or paresis
2992.11	o/e - spastic gait	Paralysis or paresis
8E53.00	exercises for paralysis	Paralysis or paresis
8E54.00	exercises for spasticity	Paralysis or paresis
F22z.00	hemiplegia nos	Paralysis or paresis
F230000	congenital paraplegia	Paralysis or paresis
F230z00	congenital diplegia nos	Paralysis or paresis
F234.00	infantile hemiplegia nos	Paralysis or paresis
F23y000	ataxic infantile cerebral palsy	Paralysis or paresis
F23yz00	other infantile cerebral palsy nos	Paralysis or paresis
F23z.00	congenital cerebral palsy nos	Paralysis or paresis
F240000	flaccid tetraplegia	Paralysis or paresis
F240100	spastic tetraplegia	Paralysis or paresis
F241000	flaccid paraplegia	Paralysis or paresis
F241100	spastic paraplegia	Paralysis or paresis
F245.00	monoplegia unspecified	Paralysis or paresis
F246.00	cauda equina syndrome	Paralysis or paresis
F246000	cauda equina syndrome not affecting bladder	Paralysis or paresis
F246z00	cauda equina syndrome nos	Paralysis or paresis
F24z.00	paralysis nos	Paralysis or paresis
Fyu9.00	[x]cerebral palsy and other paralytic syndromes	Paralysis or paresis
Fyu9000	[x]other infantile cerebral palsy	Paralysis or paresis
N12C300	lumbar disc prolapse with cauda equina compression	Paralysis or paresis
Q206.00	brachial plexus palsy due to birth trauma	Paralysis or paresis
Q206000	brachial palsy unspecified, due to birth trauma	Paralysis or paresis
Q206y00	other specified brachial plexus palsy due to birth trauma	Paralysis or paresis
Q206z00	brachial plexus palsy due to birth trauma nos	Paralysis or paresis
Q207.00	other cranial or peripheral nerve palsy due to birth trauma	Paralysis or paresis

Q207z00	cranial or peripheral nerve palsy due to birth trauma nos	Paralysis or paresis
R012200	[d]spastic gait	Paralysis or paresis
R014.00	[d]transient paralysis of a limb	Paralysis or paresis
R014000	[d]transient monoplegia nos	Paralysis or paresis
S114500	closed spinal fracture with cauda equina lesion	Paralysis or paresis
SJ24000	closed injury cauda equina	Paralysis or paresis
F23..12	infantile cerebral palsy	Paralysis or paresis
F23y200	spastic cerebral palsy	Paralysis or paresis
F233.11	congenital spastic foot	Paralysis or paresis
F23y300	dyskinetic cerebral palsy	Paralysis or paresis
7L0D111	correction of erb's palsy	Paralysis or paresis
F393.11	familial hypokalaemic periodic paralysis	Paralysis or paresis
SN48112	paralysis following electric shock	Paralysis or paresis
7L0D100	correction of obstetric palsy	Paralysis or paresis
F246100	cauda equina syndrome with cord bladder	Paralysis or paresis
F393.00	familial periodic paralysis	Paralysis or paresis
F23..00	congenital cerebral palsy	Paralysis or paresis
F23..11	congenital spastic cerebral palsy	Paralysis or paresis
F232.00	congenital quadriplegia	Paralysis or paresis
F230.00	congenital diplegia	Paralysis or paresis
F223.00	right hemiplegia	Paralysis or paresis
F222.00	left hemiplegia	Paralysis or paresis
F221.11	spastic foot	Paralysis or paresis
F24yz11	specified palsy nec	Paralysis or paresis
G669.00	cerebral palsy, not congenital or infantile, acute	Paralysis or paresis
F038.00	tropical spastic paraplegia	Paralysis or paresis
A941.11	general paralysis of insane	Paralysis or paresis
F137.11	athetoid cerebral palsy	Paralysis or paresis
F233.00	congenital monoplegia	Paralysis or paresis
SJ24.00	cauda equina injury without bony injury	Paralysis or paresis
F230100	cerebral palsy with spastic diplegia	Paralysis or paresis
F141.00	hereditary spastic paraplegia	Paralysis or paresis
F244.00	monoplegia of upper limb	Paralysis or paresis
N231011	massive muscular calcification associated with paraplegia	Paralysis or paresis
F231.00	congenital hemiplegia	Paralysis or paresis
F23y100	flaccid infantile cerebral palsy	Paralysis or paresis
F22..00	hemiplegia	Paralysis or paresis
F343000	acute radial nerve palsy	Paralysis or paresis
F242.00	diplegia of upper limbs	Paralysis or paresis
F152200	progressive bulbar palsy	Paralysis or paresis
F342100	tardy ulnar nerve palsy	Paralysis or paresis
344 SH	spastic hemiplegia	Paralysis or paresis
2833	o/e - hemiplegia	Paralysis or paresis
344 G	paraplegia	Paralysis or paresis
2835	o/e - paraplegia	Paralysis or paresis
343 PR	spastic paraplegia	Paralysis or paresis

J120200	acute duodenal ulcer with perforation	Peptic ulcer
J140200	acute gastrojejunal ulcer with perforation	Peptic ulcer
7627100	suture of duodenal ulcer not elsewhere classified	Peptic ulcer
J13..11	stress ulcer nos	Peptic ulcer
J111111	bleeding chronic gastric ulcer	Peptic ulcer
J121111	bleeding chronic duodenal ulcer	Peptic ulcer
J110111	bleeding acute gastric ulcer	Peptic ulcer
J130200	acute peptic ulcer with perforation	Peptic ulcer
J113.00	non steroidal anti inflammatory drug induced gastric ulcer	Peptic ulcer
J126.00	non steroidal anti inflammatory drug induced duodenal ulcer	Peptic ulcer
J110.00	acute gastric ulcer	Peptic ulcer
J111.00	chronic gastric ulcer	Peptic ulcer
J121.00	chronic duodenal ulcer	Peptic ulcer
J131.00	chronic peptic ulcer	Peptic ulcer
J141.00	chronic gastrojejunal ulcer	Peptic ulcer
J11..00	gastric ulcer - (gu)	Peptic ulcer
J14..00	gastrojejunal ulcer (gju)	Peptic ulcer
1956	peptic ulcer symptoms	Peptic ulcer
J112.00	anti-platelet induced gastric ulcer	Peptic ulcer
J125.00	anti-platelet induced duodenal ulcer	Peptic ulcer
7612500	resection of gastric ulcer by cautery	Peptic ulcer
761J.11	stomach ulcer operations	Peptic ulcer
761J.00	operations on gastric ulcer	Peptic ulcer
761J000	closure of perforated gastric ulcer	Peptic ulcer
761Jy00	other specified operation on gastric ulcer	Peptic ulcer
761Jz00	operation on gastric ulcer nos	Peptic ulcer
7627.00	operations on duodenal ulcer	Peptic ulcer
7627000	closure of perforated duodenal ulcer	Peptic ulcer
7627y00	other specified operation on duodenal ulcer	Peptic ulcer
7627z00	operation on duodenal ulcer nos	Peptic ulcer
J14..15	stomal ulcer	Peptic ulcer
J110000	acute gastric ulcer without mention of complication	Peptic ulcer
J110400	acute gastric ulcer with obstruction	Peptic ulcer
J110y00	acute gastric ulcer unspecified	Peptic ulcer
J110z00	acute gastric ulcer nos	Peptic ulcer
J111000	chronic gastric ulcer without mention of complication	Peptic ulcer
J111400	chronic gastric ulcer with obstruction	Peptic ulcer
J111y00	chronic gastric ulcer unspecified	Peptic ulcer
J111z00	chronic gastric ulcer nos	Peptic ulcer
J11y.00	unspecified gastric ulcer	Peptic ulcer
J11y000	unspecified gastric ulcer without mention of complication	Peptic ulcer
J11y100	unspecified gastric ulcer with haemorrhage	Peptic ulcer
J11y200	unspecified gastric ulcer with perforation	Peptic ulcer
J11y300	unspecified gastric ulcer with haemorrhage and perforation	Peptic ulcer
J11y400	unspecified gastric ulcer with obstruction	Peptic ulcer
J11yz00	unspecified gastric ulcer nos	Peptic ulcer

J120.00	acute duodenal ulcer	Peptic ulcer
J120000	acute duodenal ulcer without mention of complication	Peptic ulcer
J120400	acute duodenal ulcer with obstruction	Peptic ulcer
J120y00	acute duodenal ulcer unspecified	Peptic ulcer
J120z00	acute duodenal ulcer nos	Peptic ulcer
J121000	chronic duodenal ulcer without mention of complication	Peptic ulcer
J121400	chronic duodenal ulcer with obstruction	Peptic ulcer
J121y00	chronic duodenal ulcer unspecified	Peptic ulcer
J121z00	chronic duodenal ulcer nos	Peptic ulcer
J124.00	recurrent duodenal ulcer	Peptic ulcer
J12y.00	unspecified duodenal ulcer	Peptic ulcer
J12y000	unspecified duodenal ulcer without mention of complication	Peptic ulcer
J12y100	unspecified duodenal ulcer with haemorrhage	Peptic ulcer
J12y200	unspecified duodenal ulcer with perforation	Peptic ulcer
J12y300	unspecified duodenal ulcer with haemorrhage and perforation	Peptic ulcer
J12y400	unspecified duodenal ulcer with obstruction	Peptic ulcer
J12yz00	unspecified duodenal ulcer nos	Peptic ulcer
J12z.00	duodenal ulcer nos	Peptic ulcer
J130.00	acute peptic ulcer	Peptic ulcer
J130000	acute peptic ulcer without mention of complication	Peptic ulcer
J130400	acute peptic ulcer with obstruction	Peptic ulcer
J130y00	acute peptic ulcer unspecified	Peptic ulcer
J130z00	acute peptic ulcer nos	Peptic ulcer
J131000	chronic peptic ulcer without mention of complication	Peptic ulcer
J131400	chronic peptic ulcer with obstruction	Peptic ulcer
J131y00	chronic peptic ulcer unspecified	Peptic ulcer
J131z00	chronic peptic ulcer nos	Peptic ulcer
J13y.00	unspecified peptic ulcer	Peptic ulcer
J13y000	unspecified peptic ulcer without mention of complication	Peptic ulcer
J13y100	unspecified peptic ulcer with haemorrhage	Peptic ulcer
J13y200	unspecified peptic ulcer with perforation	Peptic ulcer
J13y300	unspecified peptic ulcer with haemorrhage and perforation	Peptic ulcer
J13y400	unspecified peptic ulcer with obstruction	Peptic ulcer
J13yz00	unspecified peptic ulcer nos	Peptic ulcer
J13z.00	peptic ulcer nos	Peptic ulcer
J140.00	acute gastrojejunal ulcer	Peptic ulcer
J140000	acute gastrojejunal ulcer without mention of complication	Peptic ulcer
J140400	acute gastrojejunal ulcer with obstruction	Peptic ulcer
J140y00	acute gastrojejunal ulcer unspecified	Peptic ulcer
J140z00	acute gastrojejunal ulcer nos	Peptic ulcer
J141000	chronic gastrojejunal ulcer without mention of complication	Peptic ulcer
J141400	chronic gastrojejunal ulcer with obstruction	Peptic ulcer
J141y00	chronic gastrojejunal ulcer unspecified	Peptic ulcer
J141z00	chronic gastrojejunal ulcer nos	Peptic ulcer
J14y.00	unspecified gastrojejunal ulcer	Peptic ulcer
J14y100	unspecified gastrojejunal ulcer with haemorrhage	Peptic ulcer

J14y200	unspecified gastrojejunal ulcer with perforation	Peptic ulcer
J14y400	unspecified gastrojejunal ulcer with obstruction	Peptic ulcer
J14yz00	unspecified gastrojejunal ulcer nos	Peptic ulcer
J14z.00	gastrojejunal ulcer nos	Peptic ulcer
J17y800	healed gastric ulcer leaving a scar	Peptic ulcer
J112z00	anti-platelet induced gastric ulcer nos	Peptic ulcer
J110200	acute gastric ulcer with perforation	Peptic ulcer
ZV12712	[v]personal history of duodenal ulcer	Peptic ulcer
ZV12711	[v]personal history of peptic ulcer	Peptic ulcer
J11z.11	gastric erosions	Peptic ulcer
J123.00	duodenal erosion	Peptic ulcer
J130300	acute peptic ulcer with haemorrhage and perforation	Peptic ulcer
7627200	oversew of blood vessel of duodenal ulcer	Peptic ulcer
J11..11	prepyloric ulcer	Peptic ulcer
761J100	closure of gastric ulcer nec	Peptic ulcer
J11z.00	gastric ulcer nos	Peptic ulcer
J13..00	peptic ulcer - (pu) site unspecified	Peptic ulcer
14C1.00	h/o: peptic ulcer	Peptic ulcer
J14..12	gastrocolic ulcer	Peptic ulcer
761J111	suture of ulcer of stomach nec	Peptic ulcer
14C1.11	h/o: duodenal ulcer	Peptic ulcer
14C1.12	h/o: gastric ulcer	Peptic ulcer
ZV12C00	[v] personal history of gastric ulcer	Peptic ulcer
J11z.12	multiple gastric ulcers	Peptic ulcer
761D500	endoscopic injection haemostasis of duodenal ulcer	Peptic ulcer
761D600	endoscopic injection haemostasis of gastric ulcer	Peptic ulcer
J130100	acute peptic ulcer with haemorrhage	Peptic ulcer
J120100	acute duodenal ulcer with haemorrhage	Peptic ulcer
J111211	perforated chronic gastric ulcer	Peptic ulcer
J121300	chronic duodenal ulcer with haemorrhage and perforation	Peptic ulcer
J141300	chronic gastrojejunal ulcer with haemorrhage and perforation	Peptic ulcer
J110300	acute gastric ulcer with haemorrhage and perforation	Peptic ulcer
J131100	chronic peptic ulcer with haemorrhage	Peptic ulcer
J121211	perforated chronic duodenal ulcer	Peptic ulcer
J111100	chronic gastric ulcer with haemorrhage	Peptic ulcer
J102000	peptic ulcer of oesophagus	Peptic ulcer
J131300	chronic peptic ulcer with haemorrhage and perforation	Peptic ulcer
J141100	chronic gastrojejunal ulcer with haemorrhage	Peptic ulcer
J140100	acute gastrojejunal ulcer with haemorrhage	Peptic ulcer
7612111	balfour excision of gastric ulcer	Peptic ulcer
J140300	acute gastrojejunal ulcer with haemorrhage and perforation	Peptic ulcer
J120300	acute duodenal ulcer with haemorrhage and perforation	Peptic ulcer
J121100	chronic duodenal ulcer with haemorrhage	Peptic ulcer
J110100	acute gastric ulcer with haemorrhage	Peptic ulcer
J111200	chronic gastric ulcer with perforation	Peptic ulcer
J111300	chronic gastric ulcer with haemorrhage and perforation	Peptic ulcer

J141200	chronic gastrojejunal ulcer with perforation	Peptic ulcer
J12..00	duodenal ulcer - (du)	Peptic ulcer
J11..12	pyloric ulcer	Peptic ulcer
J131200	chronic peptic ulcer with perforation	Peptic ulcer
J14y000	unspecified gastrojejunal ulcer without mention complication	Peptic ulcer
J12yy00	unspec duodenal ulcer; unspec haemorrhage and/or perforation	Peptic ulcer
J11yy00	unspec gastric ulcer; unspec haemorrhage and/or perforation	Peptic ulcer
J14y300	unspec gastrojejunal ulcer with haemorrhage and perforation	Peptic ulcer
J14yy00	unspec gastrojejunal ulcer; unspec haemorrhage/perforation	Peptic ulcer
J13yy00	unspec peptic ulcer; unspec haemorrhage and/or perforation	Peptic ulcer
J121200	chronic duodenal ulcer with perforation	Peptic ulcer
J122.00	duodenal ulcer disease	Peptic ulcer
J14..13	jejunal ulcer	Peptic ulcer
7627	operations on duodenal ulcer	Peptic ulcer
G72y100	aneurysm of external carotid artery	Peripheral vascular disease
G73z000	intermittent claudication	Peripheral vascular disease
G72y800	aneurysm of superior mesenteric artery	Peripheral vascular disease
G71..00	aortic aneurysm	Peripheral vascular disease
G72y500	aneurysm of splenic artery	Peripheral vascular disease
G713.00	abdominal aortic aneurysm which has ruptured	Peripheral vascular disease
G673.00	cerebral aneurysm, nonruptured	Peripheral vascular disease
G701.00	renal artery atherosclerosis	Peripheral vascular disease
7A28200	percutaneous transluminal angioplasty of vertebral artery	Peripheral vascular disease
G712.00	thoracic aortic aneurysm without mention of rupture	Peripheral vascular disease
7A23800	percutaneous coil embolisation of cerebral artery aneurysm	Peripheral vascular disease
G723000	aneurysm of femoral artery	Peripheral vascular disease
7A1B000	endovascular stenting infrarenal abdominal aortic aneurysm	Peripheral vascular disease
7A58000	percutaneous transluminal coil embolisation aneurysm small	Peripheral vascular disease
7A58100	percutaneous transluminal coil embolisation aneurysm medium	Peripheral vascular disease
7A58300	percutaneous transluminal coil embolisation aneurysm giant	Peripheral vascular disease
7A59200	perc translum balloon assist coil embolisat single aneurysm	Peripheral vascular disease
7A59z00	transluminal balloon assisted coil embolisation aneurysm nos	Peripheral vascular disease
7A5A000	perc translumin stent assis coil embolis three more aneurysm	Peripheral vascular disease
7A5A200	perc trans stent assisted coil embolisation single aneurysm	Peripheral vascular disease
7A5Az00	transluminal stent assisted coil embolisation aneurysm nos	Peripheral vascular disease
7A5B000	percutaneous translumin liquid polymer embolisation aneurysm	Peripheral vascular disease
7A1C.00	translum insert stent graft for aneurysmal segment of aorta	Peripheral vascular disease
7A1C000	endovas ins stent graft for infrarenal abdom aortic aneurysm	Peripheral vascular disease
7A1C100	endovas insert of stent graft for suprarenal aortic aneurysm	Peripheral vascular disease
7A1C200	endov insertion of stent graft for thoracic aortic aneurysm	Peripheral vascular disease
7A1C300	endov ins stent graft for aortic dissection in any position	Peripheral vascular disease
7A1C500	endovas insertion of stent graft for aorto-uniiliac aneurysm	Peripheral vascular disease
7A1Cy00	os translum ins stent graft for aneurysmal segment of aorta	Peripheral vascular disease
7A1Cz00	translum ins stent graft for aneurysmal segment of aorta nos	Peripheral vascular disease
7A1B800	endovascul insert stent infrarenal abdominal aortic aneurysm	Peripheral vascular disease
7A1B900	endovascular insertion stent for suprarenal aortic aneurysm	Peripheral vascular disease

7A1BB00	endovascular ins stent for aortic dissection in any position	Peripheral vascular disease
7A1BC00	endovas insert stent for aortic aneurysm of bifurcation nec	Peripheral vascular disease
G73z012	vascular claudication	Peripheral vascular disease
7A46.11	other replacement aneurysmal femoral artery by anastomosis	Peripheral vascular disease
7A14400	replace aneurysm abdominal aorta by anast aorta to aorta nec	Peripheral vascular disease
7A14000	replace aneurysm ascend aorta by anast of aorta/aorta nec	Peripheral vascular disease
7A11100	replace aneurysm bifurc aorta by anast aorta to femoral art	Peripheral vascular disease
7A11300	replace aneurysm bifurc aorta by anast aorta to iliac artery	Peripheral vascular disease
7A46z00	other replacement of aneurysmal femoral/popliteal artery nos	Peripheral vascular disease
7A46000	replace aneurysm fem art by fem/pop art anastom c prosth nec	Peripheral vascular disease
7A46C00	replace aneurysm fem artery by fem/fem art anastomosis nec	Peripheral vascular disease
7A40A00	replace aneurysm iliac art by aorta/ext iliac art anast nec	Peripheral vascular disease
7A46300	replace aneurysm pop art by pop/pop a anast c vein graft nec	Peripheral vascular disease
7A46y00	other replacement of aneurysmal femoral/popliteal artery os	Peripheral vascular disease
7A46100	replace aneurysm pop art by pop/pop art anastom c prosth nec	Peripheral vascular disease
7A46D00	replace aneurysm popliteal artery by pop/fem anastomosis nec	Peripheral vascular disease
7A14100	replace aneurysm thoracic aorta by anast of aorta/aorta nec	Peripheral vascular disease
7A11.00	replacement of aneurysmal bifurcation of aorta	Peripheral vascular disease
7A11y00	replacement of aneurysmal bifurcation of aorta os	Peripheral vascular disease
7A14y00	other replacement of aneurysmal segment of aorta os	Peripheral vascular disease
G714200	infrarenal abdominal aortic aneurysm	Peripheral vascular disease
7A10400	bypass aorta anastomosis axillary artery bi femoral arteries	Peripheral vascular disease
66f3.00	aortic aneurysm monitoring	Peripheral vascular disease
G726.00	dissection of renal artery	Peripheral vascular disease
G728.00	dissection of artery of lower extremity	Peripheral vascular disease
G72A.00	dissection of other specified arteries	Peripheral vascular disease
G72B.00	dissection of artery	Peripheral vascular disease
G734.00	peripheral arterial disease	Peripheral vascular disease
G72y700	aneurysm of coeliac artery	Peripheral vascular disease
585I000	abdominal aortic aneurysm screen ultrasound scan abnormal	Peripheral vascular disease
662U.00	peripheral vascular disease monitoring	Peripheral vascular disease
G723600	post radiological femoral false aneurysm	Peripheral vascular disease
G727.00	dissection of iliac artery	Peripheral vascular disease
G700.00	aortic atherosclerosis	Peripheral vascular disease
9m11.00	peripheral vascular disease monitoring second letter	Peripheral vascular disease
9m12.00	peripheral vascular disease monitoring third letter	Peripheral vascular disease
G722.00	aneurysm of iliac artery	Peripheral vascular disease
7A35300	percutaneous transluminal angioplasty suprarenal artery nec	Peripheral vascular disease
P766.00	peripheral arterio-venous aneurysm	Peripheral vascular disease
G73z.00	peripheral vascular disease nos	Peripheral vascular disease
7A35200	percutaneous transluminal angioplasty inf mesenteric art nec	Peripheral vascular disease
7A28100	percutaneous transluminal angioplasty of brachial artery	Peripheral vascular disease
7A4B100	percutaneous transluminal angioplasty of popliteal artery	Peripheral vascular disease
7A28000	percutaneous transluminal angioplasty of subclavian artery	Peripheral vascular disease
7A35100	percutaneous transluminal angioplasty sup mesenteric art nec	Peripheral vascular disease
G713.11	ruptured abdominal aortic aneurysm	Peripheral vascular disease

14AE.00	h/o: aortic aneurysm	Peripheral vascular disease
G710.00	dissecting aortic aneurysm	Peripheral vascular disease
9N4h.00	dna - did not attend peripheral vascular disease clinic	Peripheral vascular disease
2456.00	o/e - arterial wall - aneurysm	Peripheral vascular disease
2I16.00	o/e - gangrene	Peripheral vascular disease
G714300	aneurysm of suprarenal aorta	Peripheral vascular disease
7A10300	axillo-unifemoral ptfe bypass graft	Peripheral vascular disease
7A11z00	replacement of aneurysmal bifurcation of aorta nos	Peripheral vascular disease
7A12.00	other bypass of bifurcation of aorta	Peripheral vascular disease
7A12y00	other specified other bypass of bifurcation of aorta	Peripheral vascular disease
7A12z00	other bypass of bifurcation of aorta nos	Peripheral vascular disease
7A13.00	emergency replacement of aneurysmal segment of aorta	Peripheral vascular disease
7A13.11	emergency repair of aortic aneurysm	Peripheral vascular disease
7A13z00	emergency replacement of aneurysmal segment of aorta nos	Peripheral vascular disease
7A14z00	other replacement of aneurysmal segment of aorta nos	Peripheral vascular disease
7A15.00	other emergency bypass of segment of aorta	Peripheral vascular disease
7A15y00	other specified other emergency bypass of segment of aorta	Peripheral vascular disease
7A15z00	other emergency bypass of segment of aorta nos	Peripheral vascular disease
7A16.00	other bypass of segment of aorta	Peripheral vascular disease
7A16y00	other specified other bypass of segment of aorta	Peripheral vascular disease
7A16z00	other bypass of segment of aorta nos	Peripheral vascular disease
7A17100	revision of prosthesis of bifurcation of aorta	Peripheral vascular disease
7A19200	open embolectomy of bifurcation of aorta	Peripheral vascular disease
7A19400	operation on aneurysm of aorta nec	Peripheral vascular disease
7A1A100	percutaneous transluminal angioplasty of aorta nec	Peripheral vascular disease
7A21400	operation on aneurysm of carotid artery	Peripheral vascular disease
7A22000	percutaneous transluminal angioplasty of carotid artery	Peripheral vascular disease
7A26D00	reconstruction of subclavian artery with vein graft	Peripheral vascular disease
7A26F00	reconstruction of brachial artery with vein graft	Peripheral vascular disease
7A27C00	operation on aneurysm of subclavian artery	Peripheral vascular disease
7A27D00	operation on aneurysm of axillary artery	Peripheral vascular disease
7A27E00	operation on aneurysm of brachial artery	Peripheral vascular disease
7A28C00	percutaneous transluminal angioplasty of axillary artery	Peripheral vascular disease
7A31300	operation on aneurysm of renal artery	Peripheral vascular disease
7A34D00	operation on aneurysm of superior mesenteric artery nec	Peripheral vascular disease
7A34E00	operation on aneurysm of inferior mesenteric artery nec	Peripheral vascular disease
7A34F00	operation on aneurysm of suprarenal artery nec	Peripheral vascular disease
7A35000	percutaneous transluminal angioplasty of coeliac artery nec	Peripheral vascular disease
7A40y00	other specified replacement of aneurysmal iliac artery	Peripheral vascular disease
7A40z00	replacement of aneurysmal iliac artery nos	Peripheral vascular disease
7A41y00	other specified other bypass of iliac artery	Peripheral vascular disease
7A41z00	other bypass of iliac artery nos	Peripheral vascular disease
7A43200	operation on aneurysm of iliac artery nec	Peripheral vascular disease
7A44000	percutaneous transluminal angioplasty of iliac artery	Peripheral vascular disease
7A48z00	other bypass of femoral artery or popliteal artery nos	Peripheral vascular disease
7A49800	reconstruction of femoral artery with vein graft	Peripheral vascular disease

7A49900	reconstruction of popliteal artery with vein graft	Peripheral vascular disease
7A4A400	ligation of aneurysm of popliteal artery	Peripheral vascular disease
7A4A500	operation on aneurysm of femoral artery nec	Peripheral vascular disease
7A4B000	percutaneous transluminal angioplasty of femoral artery	Peripheral vascular disease
7A52300	repair of artery using vein graft nec	Peripheral vascular disease
7A53400	operation on aneurysm of artery nec	Peripheral vascular disease
7A54000	percutaneous transluminal angioplasty of artery nec	Peripheral vascular disease
P72z100	congenital aneurysm of aorta	Peripheral vascular disease
G341z00	aneurysm of heart nos	Peripheral vascular disease
G421.00	aneurysm of pulmonary artery	Peripheral vascular disease
G702.00	extremity artery atheroma	Peripheral vascular disease
G702z00	extremity artery atheroma nos	Peripheral vascular disease
G70z.00	arteriosclerotic vascular disease nos	Peripheral vascular disease
G711.00	thoracic aortic aneurysm which has ruptured	Peripheral vascular disease
G711.11	ruptured thoracic aortic aneurysm	Peripheral vascular disease
G714.00	abdominal aortic aneurysm without mention of rupture	Peripheral vascular disease
G714.11	aaa - abdominal aortic aneurysm without mention of rupture	Peripheral vascular disease
G715.00	ruptured aortic aneurysm nos	Peripheral vascular disease
G715000	thoracoabdominal aortic aneurysm, ruptured	Peripheral vascular disease
G716.00	aortic aneurysm without mention of rupture nos	Peripheral vascular disease
G716000	thoracoabdominal aortic aneurysm, without mention of rupture	Peripheral vascular disease
G718.00	leaking abdominal aortic aneurysm	Peripheral vascular disease
G71z.00	aortic aneurysm nos	Peripheral vascular disease
G72..00	other aneurysm	Peripheral vascular disease
G720000	aneurysm of brachial artery	Peripheral vascular disease
G720100	aneurysm of radial artery	Peripheral vascular disease
G720200	aneurysm of ulnar artery	Peripheral vascular disease
G720z00	aneurysm of arm artery nos	Peripheral vascular disease
G722000	aneurysm of common iliac artery	Peripheral vascular disease
G722100	aneurysm of external iliac artery	Peripheral vascular disease
G722200	aneurysm of internal iliac artery	Peripheral vascular disease
G722z00	aneurysm of iliac artery nos	Peripheral vascular disease
G723200	aneurysm of anterior tibial artery	Peripheral vascular disease
G723300	aneurysm of dorsalis pedis artery	Peripheral vascular disease
G723400	aneurysm of posterior tibial artery	Peripheral vascular disease
G723z00	aneurysm of leg artery nos	Peripheral vascular disease
G72y.00	aneurysm of other artery	Peripheral vascular disease
G72y300	aneurysm of neck artery nos	Peripheral vascular disease
G72y900	aneurysm of inferior mesenteric artery	Peripheral vascular disease
G72yB00	aneurysm of other visceral artery	Peripheral vascular disease
G72yz00	other aneurysm nos	Peripheral vascular disease
G72z.00	aneurysm nos	Peripheral vascular disease
G731100	presenile gangrene	Peripheral vascular disease
G731z00	thromboangiitis obliterans nos	Peripheral vascular disease
G732.00	peripheral gangrene	Peripheral vascular disease
G732000	gangrene of toe	Peripheral vascular disease

G732100	gangrene of foot	Peripheral vascular disease
G732200	gangrene of finger	Peripheral vascular disease
G732300	gangrene of thumb	Peripheral vascular disease
G732400	gangrene of hand	Peripheral vascular disease
G73y.00	other specified peripheral vascular disease	Peripheral vascular disease
G73yz00	other specified peripheral vascular disease nos	Peripheral vascular disease
G73zz00	peripheral vascular disease nos	Peripheral vascular disease
G741.00	embolism and thrombosis of the thoracic aorta	Peripheral vascular disease
G742.00	embolism and thrombosis of an arm or leg artery	Peripheral vascular disease
G742000	embolism and thrombosis of the brachial artery	Peripheral vascular disease
G742100	embolism and thrombosis of the radial artery	Peripheral vascular disease
G742200	embolism and thrombosis of the ulnar artery	Peripheral vascular disease
G742300	embolism and thrombosis of an arm artery nos	Peripheral vascular disease
G742400	embolism and thrombosis of the femoral artery	Peripheral vascular disease
G742500	embolism and thrombosis of the popliteal artery	Peripheral vascular disease
G742600	embolism and thrombosis of the anterior tibial artery	Peripheral vascular disease
G742700	embolism and thrombosis of the dorsalis pedis artery	Peripheral vascular disease
G742800	embolism and thrombosis of the posterior tibial artery	Peripheral vascular disease
G742900	embolism and thrombosis of a leg artery nos	Peripheral vascular disease
G742z00	peripheral arterial embolism and thrombosis nos	Peripheral vascular disease
G74y.00	embolism and thrombosis of other specified artery	Peripheral vascular disease
G74y000	embolism and/or thrombosis of the common iliac artery	Peripheral vascular disease
G74y300	embolism and thrombosis of the iliac artery unspecified	Peripheral vascular disease
G74y500	embolism and thrombosis of the subclavian artery	Peripheral vascular disease
G74y600	embolism and thrombosis of the splenic artery	Peripheral vascular disease
G74y700	embolism and thrombosis of the axillary artery	Peripheral vascular disease
G74y800	embolism and thrombosis of the coeliac artery	Peripheral vascular disease
G74y900	embolism and thrombosis of the hepatic artery	Peripheral vascular disease
G74yz00	embolism and thrombosis of other arteries nos	Peripheral vascular disease
G74z.00	arterial embolism and thrombosis nos	Peripheral vascular disease
G76A.00	arterial insufficiency	Peripheral vascular disease
Gyu7000	[x]atherosclerosis of other arteries	Peripheral vascular disease
Gyu7100	[x]aortic aneurysm of unspecified site, ruptured	Peripheral vascular disease
Gyu7200	[x]aortic aneurysm of unspecified site, nonruptured	Peripheral vascular disease
Gyu7300	[x]aneurysm of other specified arteries	Peripheral vascular disease
Gyu7400	[x]other specified peripheral vascular diseases	Peripheral vascular disease
Gyu7800	[x]aneurysm of aorta in diseases classified elsewhere	Peripheral vascular disease
7A47z00	other emergency bypass of femoral or popliteal artery nos	Peripheral vascular disease
P76z.00	peripheral vascular system anomaly nos	Peripheral vascular disease
7A47y00	other emergency bypass of femoral or popliteal artery os	Peripheral vascular disease
R054.00	[d]gangrene	Peripheral vascular disease
R054000	[d]gangrene, spreading cutaneous	Peripheral vascular disease
R054z00	[d]gangrene nos	Peripheral vascular disease
SP12z00	peripheral vascular complications of care nos	Peripheral vascular disease
7A42.11	reconstruction of common iliac artery	Peripheral vascular disease
7A48.16	other bypass of superficial femoral artery	Peripheral vascular disease

7A48.15	other bypass of popliteal artery	Peripheral vascular disease
7A48.14	other bypass of femoral artery	Peripheral vascular disease
7A48.12	other bypass of common femoral artery	Peripheral vascular disease
7A48.11	other bypass of femoral or popliteal artery by anastomosis	Peripheral vascular disease
7A47.16	other emergency bypass of femoral artery	Peripheral vascular disease
7A47.15	other emergency bypass of superficial femoral artery	Peripheral vascular disease
7A47.14	other emergency bypass of popliteal artery	Peripheral vascular disease
7A47.13	other emergency bypass of deep femoral artery	Peripheral vascular disease
7A47.12	other emergency bypass of common femoral artery	Peripheral vascular disease
7A45.14	emergency replacement of aneurysmal popliteal artery	Peripheral vascular disease
7A45.12	emergency replacement of aneurysmal common femoral artery	Peripheral vascular disease
7A41.11	other bypass of iliac artery by anastomosis	Peripheral vascular disease
7A14.11	aortic aneurysm repair	Peripheral vascular disease
7A11211	y graft of abdominal aortic aneurysm (emergency)	Peripheral vascular disease
7A14411	tube graft of abdominal aortic aneurysm	Peripheral vascular disease
7A13411	tube graft abdominal aortic aneurysm (emergency)	Peripheral vascular disease
7A40.11	replacement of aneurysmal iliac artery by anastomosis	Peripheral vascular disease
G73..13	peripheral ischaemia	Peripheral vascular disease
G73..11	peripheral ischaemic vascular disease	Peripheral vascular disease
7A1B.00	transluminal operations on aneurysmal segment of aorta	Peripheral vascular disease
7A1B200	endovascular stenting of thoracic aortic aneurysm	Peripheral vascular disease
M271400	mixed venous and arterial leg ulcer	Peripheral vascular disease
M271300	arterial leg ulcer	Peripheral vascular disease
7A58.00	transluminal coil embolisation of aneurysm	Peripheral vascular disease
7A1B300	endovascular stenting of aortic dissection in any position	Peripheral vascular disease
7A1B500	endovascular stenting of aorto-uniiliac aneurysm	Peripheral vascular disease
7A1B100	endovascular stenting of suprarenal aortic aneurysm	Peripheral vascular disease
7A5B.00	other transluminal embolisation of aneurysm	Peripheral vascular disease
7A48y00	other bypass of femoral artery or popliteal artery os	Peripheral vascular disease
16I..00	claudication distance	Peripheral vascular disease
7A58y00	other specified transluminal coil embolisation of aneurysm	Peripheral vascular disease
7A59.00	transluminal balloon assisted coil embolisation of aneurysm	Peripheral vascular disease
7A58z00	transluminal coil embolisation of aneurysm nos	Peripheral vascular disease
7A5By00	other specified other transluminal embolisation of aneurysm	Peripheral vascular disease
7A1Bz00	transluminal operations on aneurysmal segment of aorta nos	Peripheral vascular disease
7A5A.00	transluminal stent assisted coil embolisation of aneurysm	Peripheral vascular disease
7A5Bz00	other transluminal embolisation of aneurysm nos	Peripheral vascular disease
7A14.00	other replacement of aneurysmal segment of aorta	Peripheral vascular disease
7A40.00	replacement of aneurysmal iliac artery	Peripheral vascular disease
7A41.00	other bypass of iliac artery	Peripheral vascular disease
7A46.00	other replacement of aneurysmal femoral artery	Peripheral vascular disease
7A47.00	other emergency bypass of femoral artery or popliteal artery	Peripheral vascular disease
7A48.00	other bypass of femoral artery or popliteal artery	Peripheral vascular disease
G341100	other cardiac wall aneurysm	Peripheral vascular disease
G73..00	other peripheral vascular disease	Peripheral vascular disease
G740.00	embolism and thrombosis of the abdominal aorta	Peripheral vascular disease

P76..00	other peripheral vascular system anomalies	Peripheral vascular disease
P767.00	congenital peripheral aneurysm	Peripheral vascular disease
7A11311	y graft abdominal aortic aneurysm	Peripheral vascular disease
7A42011	endarterectomy and patch repair of common iliac artery	Peripheral vascular disease
7A42111	endarterectomy of common iliac artery nec	Peripheral vascular disease
7A43.11	other open operations on common iliac artery	Peripheral vascular disease
7A43011	repair of common iliac artery nec	Peripheral vascular disease
7A44.11	transluminal operations on common iliac artery	Peripheral vascular disease
7A46.15	other replacement of aneurysmal superficial femoral artery	Peripheral vascular disease
7A46.14	other replacement of aneurysmal popliteal artery	Peripheral vascular disease
7A43111	open embolectomy of common iliac artery	Peripheral vascular disease
G73z011	claudication	Peripheral vascular disease
G72y200	aneurysm of internal carotid artery	Peripheral vascular disease
G72yA00	aneurysm of hepatic artery	Peripheral vascular disease
G701011	aras - atherosclerotic renal artery stenosis	Peripheral vascular disease
14NB.00	h/o: peripheral vascular disease procedure	Peripheral vascular disease
G723500	ruptured popliteal artery aneurysm	Peripheral vascular disease
7A6H400	percutaneous transluminal angioplasty of vascular graft	Peripheral vascular disease
G713000	ruptured suprarenal aortic aneurysm	Peripheral vascular disease
G714000	juxtarenal aortic aneurysm	Peripheral vascular disease
G714100	inflammatory abdominal aortic aneurysm	Peripheral vascular disease
7A23z00	operation on cerebral artery/ circle of willis aneurysm nos	Peripheral vascular disease
7A34K00	operation on aneurysm visceral branch of abdominal aorta nec	Peripheral vascular disease
G723.00	aneurysm of leg artery	Peripheral vascular disease
G72y000	aneurysm of common carotid art	Peripheral vascular disease
G720.00	aneurysm of artery of arm	Peripheral vascular disease
7A1B600	endovascular stenting for aortic aneurysm of bifurcation nec	Peripheral vascular disease
7A1B700	endovascular stenting for aorto-uniiliac aneurysm	Peripheral vascular disease
G74..00	arterial embolism and thrombosis	Peripheral vascular disease
7A44211	arteriography of common iliac artery	Peripheral vascular disease
G70..00	atherosclerosis	Peripheral vascular disease
7A10200	axillo-bifemoral bypass graft	Peripheral vascular disease
P7y0100	congenital cerebral arteriovenous aneurysm	Peripheral vascular disease
7A10100	bypass aorta by anastomosis axillary to femoral artery nec	Peripheral vascular disease
7A12100	bypass bifurc aorta by anastom aorta to femoral artery nec	Peripheral vascular disease
7A41900	bypass common iliac artery by aorta/com iliac art anast nec	Peripheral vascular disease
7A48800	bypass femoral artery by fem/peron a anast c prosthesis nec	Peripheral vascular disease
7A48A00	bypass femoral artery by fem/peron a anast c vein graft nec	Peripheral vascular disease
7A48000	bypass femoral artery by fem/pop art anast c prosthesis nec	Peripheral vascular disease
7A48400	bypass femoral artery by fem/tib art anast c prosthesis nec	Peripheral vascular disease
7A48C00	bypass femoral artery by femoral/femoral art anastomosis nec	Peripheral vascular disease
7A41300	bypass iliac artery by femoral/femoral art anastomosis nec	Peripheral vascular disease
7A41100	bypass iliac artery by iliac/femoral artery anastomosis nec	Peripheral vascular disease
7A41D00	bypass iliac artery by iliac/iliac artery anastomosis nec	Peripheral vascular disease
7A48300	bypass popliteal artery by pop/pop a anast c vein graft nec	Peripheral vascular disease
7A48700	bypass popliteal artery by pop/tib a anast c vein graft nec	Peripheral vascular disease

G721.00	aneurysm of renal artery	Peripheral vascular disease
G72y400	aneurysm of subclavian artery	Peripheral vascular disease
G743.00	embolism and thrombosis of other and unspec parts aorta	Peripheral vascular disease
7A12000	emerg bypass bifurc aorta by anast aorta to femoral artery	Peripheral vascular disease
7A47200	emerg bypass femoral art by fem/pop a anast c vein graft nec	Peripheral vascular disease
7A47600	emerg bypass femoral art by fem/tib a anast c vein graft nec	Peripheral vascular disease
7A47C00	emerg bypass femoral artery by fem/fem art anastomosis nec	Peripheral vascular disease
7A41000	emerg bypass iliac art by iliac/femoral art anastomosis nec	Peripheral vascular disease
7A41200	emerg bypass iliac artery by femoral/femoral art anast nec	Peripheral vascular disease
7A47300	emerg bypass pop art by pop/pop art anast c vein graft nec	Peripheral vascular disease
7A47700	emerg bypass pop art by pop/tib art anast c vein graft nec	Peripheral vascular disease
7A11000	emerg repl aneurysm bifurc aorta by anast aorta to fem art	Peripheral vascular disease
7A11200	emerg repl aneurysm bifurc aorta by anast aorta to iliac a	Peripheral vascular disease
7A13400	emerg replace aneurysm abdom aorta by anast aorta/aorta nec	Peripheral vascular disease
7A13000	emerg replace aneurysm asc aorta by anastom aorta to aorta	Peripheral vascular disease
7A45200	emerg replace aneurysm fem art by fem/pop anast c vein graft	Peripheral vascular disease
7A45000	emerg replace aneurysm fem art by fem/pop art anast c prosth	Peripheral vascular disease
7A40000	emerg replace aneurysm iliac art by iliac/femoral art anast	Peripheral vascular disease
7A13300	emerg replace aneurysm infrarenal aorta by anast aorta/aorta	Peripheral vascular disease
7A45700	emerg replace aneurysm pop art by pop/tib anast c vein graft	Peripheral vascular disease
7A45D00	emerg replace aneurysm pop artery by pop/fem art anastomosis	Peripheral vascular disease
7A13100	emerg replace aneurysm thor aorta by anastom aorta to aorta	Peripheral vascular disease
7A40200	emerg replace aneurysmal iliac artery by fem/fem art anast	Peripheral vascular disease
7A45y00	emergency replacement aneurysmal femoral/popliteal artery os	Peripheral vascular disease
7A45.15	emergency replacement aneurysmal superficial femoral artery	Peripheral vascular disease
7A45.00	emergency replacement of aneurysmal femoral/popliteal artery	Peripheral vascular disease
7A13y00	emergency replacement of aneurysmal segment of aorta os	Peripheral vascular disease
G72y600	aneurysm of axillary artery	Peripheral vascular disease
9m1..00	peripheral vascular disease monitoring invitation	Peripheral vascular disease
9m10.00	peripheral vascular disease monitoring first letter	Peripheral vascular disease
G73..12	ischaemia of legs	Peripheral vascular disease
7A1BA00	endovascular insertion of stent for thoracic aortic aneurysm	Peripheral vascular disease
7A1BD00	endovascular insertion of stent for aorto-uniiliac aneurysm	Peripheral vascular disease
G723100	aneurysm of popliteal artery	Peripheral vascular disease
G731.00	thromboangiitis obliterans	Peripheral vascular disease
G731000	buerger's disease	Peripheral vascular disease
G70y000	carotid artery atherosclerosis	Peripheral vascular disease
7A32000	percutaneous transluminal angioplasty of renal artery	Peripheral vascular disease
4439A	intermittent claudication	Peripheral vascular disease
g71..00	aortic aneurysm	Peripheral vascular disease
4419	aortic aneurysm	Peripheral vascular disease
G40..00	acute pulmonary heart disease	Pulmonary circulation disorders
H541.00	pulmonary congestion	Pulmonary circulation disorders
G41z.11	chronic cor pulmonale	Pulmonary circulation disorders
G41..00	chronic pulmonary heart disease	Pulmonary circulation disorders
G41y000	secondary pulmonary hypertension	Pulmonary circulation disorders

P736.12	pulmonary arterio-venous malformation	Pulmonary circulation disorders
G4...00	pulmonary circulation diseases	Pulmonary circulation disorders
G42..11	pulmonary vessel disease	Pulmonary circulation disorders
G401000	post operative pulmonary embolus	Pulmonary circulation disorders
7A0B000	pulmonary thromboendarterectomy	Pulmonary circulation disorders
L43z.00	obstetric pulmonary embolism nos	Pulmonary circulation disorders
G40z.00	acute pulmonary heart disease nos	Pulmonary circulation disorders
G41y.00	other chronic pulmonary heart disease	Pulmonary circulation disorders
G41yz00	other chronic pulmonary heart disease nos	Pulmonary circulation disorders
G4y..00	other specified pulmonary circulation disease	Pulmonary circulation disorders
G42yz00	other specified pulmonary circulation disease nos	Pulmonary circulation disorders
G42z.00	other pulmonary circulation disease nos	Pulmonary circulation disorders
G4z..00	pulmonary circulation disease nos	Pulmonary circulation disorders
Gyu4100	[x]other diseases of pulmonary vessels	Pulmonary circulation disorders
H54..00	pulmonary congestion and hypostasis	Pulmonary circulation disorders
L096400	pulmonary embolism following abortive pregnancy	Pulmonary circulation disorders
L43..00	obstetric pulmonary embolism	Pulmonary circulation disorders
L430.00	obstetric air pulmonary embolism	Pulmonary circulation disorders
L431100	amniotic fluid pulmonary embolism - delivered	Pulmonary circulation disorders
L432.00	obstetric blood-clot pulmonary embolism	Pulmonary circulation disorders
L43yz00	other obstetric pulmonary embolism nos	Pulmonary circulation disorders
L43zz00	obstetric pulmonary embolism nos	Pulmonary circulation disorders
L43z000	obstetric pulmonary embolism nos, unspecified	Pulmonary circulation disorders
L43z100	obstetric pulmonary embolism nos - delivered	Pulmonary circulation disorders
L43z400	obstetric pulmonary embolism nos with postnatal complication	Pulmonary circulation disorders
SB16.00	pulmonary blood vessel injury	Pulmonary circulation disorders
G41y100	thromboembolic pulmonary hypertension	Pulmonary circulation disorders
7Q01000	primary pulmonary hypertension drugs band 1	Pulmonary circulation disorders
7Q01100	primary pulmonary hypertension drugs band 2	Pulmonary circulation disorders
7Q01200	primary pulmonary hypertension drugs band 3	Pulmonary circulation disorders
7Q01300	primary pulmonary hypertension drugs band 4	Pulmonary circulation disorders
G42y.00	other specified pulmonary circulation disease	Pulmonary circulation disorders
P737.11	dilatation of pulmonary artery	Pulmonary circulation disorders
7A6Ky00	other specified operations on individual pulmonary veins	Pulmonary circulation disorders
G41z.00	chronic pulmonary heart disease nos	Pulmonary circulation disorders
G42..00	other pulmonary circulation disease	Pulmonary circulation disorders
Gyu4.00	[x]pulmon heart disease & diseases of pulmonary circulation	Pulmonary circulation disorders
G410.00	primary pulmonary hypertension	Pulmonary circulation disorders
ZV12900	[v] personal history of pulmonary embolism	Pulmonary circulation disorders
L431.00	amniotic fluid pulmonary embolism	Pulmonary circulation disorders
G401100	recurrent pulmonary embolism	Pulmonary circulation disorders
G411.00	kyphoscoliotic heart disease	Pulmonary circulation disorders
G4...11	heart disease - pulmonary	Pulmonary circulation disorders
G400.00	acute cor pulmonale	Pulmonary circulation disorders
G401.00	pulmonary embolism	Pulmonary circulation disorders
K00..11	acute nephritis	Renal disease

K100.00	chronic pyelonephritis	Renal disease
7L1A011	thomas intravascular shunt for dialysis	Renal disease
A160200	tuberculous pyelonephritis	Renal disease
K01x411	lupus nephritis	Renal disease
K13yB00	ischaemic nephropathy	Renal disease
7L1A100	peritoneal dialysis	Renal disease
K081.00	nephrogenic diabetes insipidus	Renal disease
K08y511	ain - acute interstitial nephritis	Renal disease
K08y513	acute tubulo-interstitial nephritis	Renal disease
ZV56011	[v]aftercare involving renal dialysis nos	Renal disease
ZV56y11	[v]aftercare involving peritoneal dialysis	Renal disease
K08y700	acute infective interstitial nephritis	Renal disease
K013.12	steroid sensitive nephrotic syndrome	Renal disease
K13yz11	salt-losing nephritis	Renal disease
7B00400	allotransplantation kidney from cadaver, heart non-beating	Renal disease
K0A1300	rpd prog neph syn df mesangial prolifratv glomerulonephritis	Renal disease
7L1B200	flushing of peritoneal dialysis catheter	Renal disease
SP06B00	continuous ambulatory peritoneal dialysis associated perit	Renal disease
TB11.11	renal dialysis with complication, without blame	Renal disease
TB00111	renal transplant with complication, without blame	Renal disease
7L1B100	removal of ambulatory peritoneal dialysis catheter	Renal disease
K06..12	kidney failure unspecified	Renal disease
G72D200	aneurysm of anastomotic site of dialysis av fistula	Renal disease
K01w112	wilms' tumour + nephrotic syndrome + pseudohermaphroditism	Renal disease
SP0E.00	disorders associated with peritoneal dialysis	Renal disease
Gy41.00	infection of dialysis arteriovenous fistula	Renal disease
K0A2700	recur+persist haematuria difus crescentic glomerulonephritis	Renal disease
K0A2200	recur+persist haematuria difus membranous glomerulonephritis	Renal disease
K0A2300	recur+persist haemuria df mesangial prolif glomerulnephritis	Renal disease
K0A2500	recur+persist hmuria df mesangiocapilary glomerulonephritis	Renal disease
K0A1200	rapid progres neph syn diffuse membranous glomerulonephritis	Renal disease
K0A1700	rapid progres nephritic syn df crescentic glomerulonephritis	Renal disease
K0A1100	rapid progres nephritic syn focal+segmental glomerulr lesion	Renal disease
K0A1600	rapid progressive nephritic syndrome, dense deposit disease	Renal disease
661M200	chronic kidney disease self-management plan agreed	Renal disease
F374A00	polyneuropathy in uraemia	Renal disease
K050.00	end stage renal failure	Renal disease
SP08T00	urological complication of renal transplant	Renal disease
SP08W00	vascular complication of renal transplant	Renal disease
G72C.00	ruptured aneurysm of dialysis vascular access	Renal disease
G72D.00	aneurysm of dialysis arteriovenous fistula	Renal disease
G72D100	aneurysm of needle site of dialysis arteriovenous fistula	Renal disease
K04E.00	acute kidney injury stage 3	Renal disease
Gy1..00	stenosis of dialysis vascular access	Renal disease
SP0F.00	haemodialysis first use syndrome	Renal disease
SP0G.00	anaphylactoid reaction due to haemodialysis	Renal disease

67P4100	discussion about kidney transplantation	Renal disease
Gy3..00	occlusion of dialysis vascular access	Renal disease
Gy5..00	haemorrhage of dialysis vascular access	Renal disease
Gy10.00	stenosis of dialysis arteriovenous graft	Renal disease
Gy21.00	thrombosis of dialysis arteriovenous fistula	Renal disease
Gy30.00	occlusion of dialysis arteriovenous graft	Renal disease
Gy31.00	occlusion of dialysis arteriovenous fistula	Renal disease
Gy40.00	infection of dialysis arteriovenous graft	Renal disease
Gy51.00	haemorrhage of dialysis arteriovenous fistula	Renal disease
Gy60.00	rupture of dialysis arteriovenous graft	Renal disease
7L1B.00	placement ambulatory apparatus compensation renal failure	Renal disease
7L1By00	placement ambulatory apparatus- compensate renal failure os	Renal disease
7L1B.11	placement ambulatory dialysis apparatus - compens renal fail	Renal disease
7L1C.00	placement other apparatus for compensation for renal failure	Renal disease
7L1Cz00	placement other apparatus- compensate for renal failure nos	Renal disease
7L1Cy00	placement other apparatus- compensate for renal failure os	Renal disease
K190X00	persistent proteinuria, unspecified	Renal disease
K0G..00	sickle cell nephropathy	Renal disease
4N0..00	dialysis fluid urea level	Renal disease
4N2..00	dialysis fluid glucose level	Renal disease
A786.00	haemorrhagic nephrosonephritis	Renal disease
14D1.00	h/o: nephritis	Renal disease
14S2.00	h/o: kidney recipient	Renal disease
14V2.11	h/o: kidney dialysis	Renal disease
14V2.00	h/o: renal dialysis	Renal disease
7261011	cyclodialysis	Renal disease
4I29.00	peritoneal dialysis sample	Renal disease
SP07G00	stenosis of arteriovenous dialysis fistula	Renal disease
7257300	correction iridodialysis nec	Renal disease
7L1f000	extracorporeal albumin haemodialysis	Renal disease
7A60600	creation of graft fistula for dialysis	Renal disease
7L1A400	automated peritoneal dialysis	Renal disease
7B00212	cadaveric renal transplant	Renal disease
7B06300	exploration of renal transplant	Renal disease
PD23.00	congenital hydronephrosis	Renal disease
K055.00	chronic kidney disease stage 5	Renal disease
K051.00	chronic kidney disease stage 1	Renal disease
K052.00	chronic kidney disease stage 2	Renal disease
K054.00	chronic kidney disease stage 4	Renal disease
K05..13	chronic kidney disease	Renal disease
K053.00	chronic kidney disease stage 3	Renal disease
7L1A200	haemodialysis nec	Renal disease
7L1Ay00	other specified compensation for renal failure	Renal disease
7L1Az00	compensation for renal failure nos	Renal disease
7L1C000	insertion of temporary peritoneal dialysis catheter	Renal disease
8882.00	intestinal dialysis	Renal disease

K080.00	renal osteodystrophy	Renal disease
K080300	renal rickets	Renal disease
8L50.00	renal transplant planned	Renal disease
A160000	tuberculous nephropathy	Renal disease
A844100	plasmodium malariae malaria with nephropathy	Renal disease
C341.00	gouty nephropathy	Renal disease
C341z00	gouty nephropathy nos	Renal disease
Cyu2300	[x]unspecified diabetes mellitus with renal complications	Renal disease
G222.00	hypertensive renal disease with renal failure	Renal disease
G233.00	hypertensive heart and renal disease with renal failure	Renal disease
SP01500	mechanical complication of dialysis catheter	Renal disease
K0...00	nephritis, nephrosis and nephrotic syndrome	Renal disease
K000.00	acute proliferative glomerulonephritis	Renal disease
K001.00	acute nephritis with lesions of necrotising glomerulitis	Renal disease
K00y.00	other acute glomerulonephritis	Renal disease
K00y000	acute glomerulonephritis in diseases ec	Renal disease
K00y100	acute exudative nephritis	Renal disease
K00y200	acute focal nephritis	Renal disease
K00y300	acute diffuse nephritis	Renal disease
K00yz00	other acute glomerulonephritis nos	Renal disease
K00z.00	acute glomerulonephritis nos	Renal disease
K010.00	nephrotic syndrome with proliferative glomerulonephritis	Renal disease
K011.00	nephrotic syndrome with membranous glomerulonephritis	Renal disease
K014.00	nephrotic syndrome, minor glomerular abnormality	Renal disease
K015.00	nephrotic syndrome, focal and segmental glomerular lesions	Renal disease
K016.00	nephrotic syndrome, diffuse membranous glomerulonephritis	Renal disease
K01A.00	nephrotic syndrome, dense deposit disease	Renal disease
K01B.00	nephrotic syndrome, diffuse crescentic glomerulonephritis	Renal disease
K01x.00	nephrotic syndrome in diseases ec	Renal disease
K01x000	nephrotic syndrome in amyloidosis	Renal disease
K01x100	nephrotic syndrome in diabetes mellitus	Renal disease
K01x111	kimmelstiel - wilson disease	Renal disease
K01x200	nephrotic syndrome in malaria	Renal disease
K01x300	nephrotic syndrome in polyarteritis nodosa	Renal disease
K01y.00	nephrotic syndrome with other pathological kidney lesions	Renal disease
K01z.00	nephrotic syndrome nos	Renal disease
K023.00	chronic rapidly progressive glomerulonephritis	Renal disease
K02y.00	other chronic glomerulonephritis	Renal disease
K02y200	chronic focal glomerulonephritis	Renal disease
K02y300	chronic diffuse glomerulonephritis	Renal disease
K02yz00	other chronic glomerulonephritis nos	Renal disease
K02z.00	chronic glomerulonephritis nos	Renal disease
K030.00	proliferative nephritis unspecified	Renal disease
K031.00	membranous nephritis unspecified	Renal disease
K032.00	membranoproliferative nephritis unspecified	Renal disease
K032000	focal membranoproliferative glomerulonephritis	Renal disease

K032300	anaphylactoid glomerulonephritis	Renal disease
K032500	other familial glomerulonephritis	Renal disease
K032600	berger's iga or igg nephropathy	Renal disease
K033.00	rapidly progressive nephritis unspecified	Renal disease
K034.00	renal cortical necrosis unspecified	Renal disease
K035.00	renal medullary necrosis unspecified	Renal disease
K03y.00	other nephritis and nephrosis unspecified	Renal disease
K03y000	other nephritis and nephrosis in diseases ec	Renal disease
K03y200	other interstitial nephritis	Renal disease
K03yz00	other nephritis and nephrosis nos	Renal disease
K03z.00	unspecified glomerulonephritis nos	Renal disease
K04y.00	other acute renal failure	Renal disease
K04z.00	acute renal failure nos	Renal disease
K08..00	impaired renal function disorder	Renal disease
K080000	phosphate-losing tubular disorders	Renal disease
K080200	renal infantilism	Renal disease
K080z00	renal osteodystrophy nos	Renal disease
K08y.00	other impaired renal function disorder	Renal disease
K08z.00	impaired renal function disorder nos	Renal disease
K0A0000	acute nephritic syndrome, minor glomerular abnormality	Renal disease
K0A0600	acute nephritic syndrome, dense deposit disease	Renal disease
K0A1.00	rapidly progressive nephritic syndrome	Renal disease
K0A3.00	chronic nephritic syndrome	Renal disease
K0A3000	chronic nephritic syndrome, minor glomerular abnormality	Renal disease
K0A3600	chronic nephritic syndrome, dense deposit disease	Renal disease
K0A4.00	isolated proteinuria with specified morphological lesion	Renal disease
K0A5.00	hereditary nephropathy not elsewhere classified	Renal disease
1Z10.00	chronic kidney disease stage 1	Renal disease
1Z11.00	chronic kidney disease stage 2	Renal disease
K0C4.00	toxic nephropathy, not elsewhere classified	Renal disease
K0y..00	other specified nephritis, nephrosis or nephrotic syndrome	Renal disease
K0z..00	nephritis, nephrosis and nephrotic syndrome nos	Renal disease
K100000	chronic pyelonephritis without medullary necrosis	Renal disease
K100100	chronic pyelonephritis with medullary necrosis	Renal disease
K100200	chronic pyelitis	Renal disease
K100300	chronic pyonephrosis	Renal disease
K100z00	chronic pyelonephritis nos	Renal disease
K101000	acute pyelonephritis without medullary necrosis	Renal disease
K101100	acute pyelonephritis with medullary necrosis	Renal disease
1Z12.00	chronic kidney disease stage 3	Renal disease
K10y.00	pyelonephritis and pyonephrosis unspecified	Renal disease
K10y300	pyelonephritis in diseases ec	Renal disease
1Z13.00	chronic kidney disease stage 4	Renal disease
1Z14.00	chronic kidney disease stage 5	Renal disease
Kyu1000	[x]other chronic tubulo-interstitial nephritis	Renal disease
Kyu1100	[x]other and unspecified hydronephrosis	Renal disease

Kyu2000	[x]other acute renal failure	Renal disease
Kyu2100	[x]other chronic renal failure	Renal disease
Kyu5G00	[x]persistent proteinuria, unspecified	Renal disease
SP08300	kidney transplant failure and rejection	Renal disease
K00..00	acute glomerulonephritis	Renal disease
TA22000	failure of sterile precautions during kidney dialysis	Renal disease
K02..00	chronic glomerulonephritis	Renal disease
7B00600	xenograft renal transplant	Renal disease
K03..12	nephropathy, unspecified	Renal disease
K105.00	chronic infective interstitial nephritis	Renal disease
K100500	chronic obstructive pyelonephritis	Renal disease
K032y15	mixed membranous and proliferative glomerulonephritis nec	Renal disease
K032y13	mesangioproliferative glomerulonephritis nec	Renal disease
K032y11	hypocomplementaemic persistent glomerulonephritis nec	Renal disease
K0A8.00	rapidly progressive glomerulonephritis	Renal disease
K000100	crescentic glomerulonephritis	Renal disease
K000111	cgn - crescentic glomerulonephritis	Renal disease
K0A2800	iga nephropathy	Renal disease
K032y14	mesangiocapillary glomerulonephritis nec	Renal disease
K060.11	impaired renal function	Renal disease
1Z1..00	chronic renal impairment	Renal disease
K0E..00	acute-on-chronic renal failure	Renal disease
C372400	urate nephropathy	Renal disease
C372411	uric acid nephropathy	Renal disease
D310100	henoch-schonlein nephritis	Renal disease
SP08R00	renal transplant rejection	Renal disease
SP08V00	very mild acute rejection of renal transplant	Renal disease
SP08H00	acute rejection of renal transplant	Renal disease
SP08E00	acute rejection of renal transplant - grade i	Renal disease
SP08F00	acute rejection of renal transplant - grade ii	Renal disease
SP08G00	acute rejection of renal transplant - grade iii	Renal disease
SP08J00	chronic rejection of renal transplant	Renal disease
SP08D00	acute-on-chronic rejection of renal transplant	Renal disease
C353600	renal failure-associated hyperphosphataemia	Renal disease
7L1A500	continuous ambulatory peritoneal dialysis	Renal disease
7L1A600	peritoneal dialysis nec	Renal disease
7B0Fz00	interventions associated with transplantation of kidney nos	Renal disease
Kyu0F00	[x]hereditary nephropathy, unspecif morphological changes	Renal disease
K0A5500	[x]heredtry nephrpthy nec difus mesangiocapilry glomneph	Renal disease
7L1A.00	compensation for renal failure	Renal disease
7L1A000	renal dialysis	Renal disease
K013.00	nephrotic syndrome with minimal change glomerulonephritis	Renal disease
K03..11	nephritis and nephropathy unspecified	Renal disease
K06..00	renal failure unspecified	Renal disease
K08yz00	other impaired renal function disorder nos	Renal disease
K100600	calculous pyelonephritis	Renal disease

7B00500	allotransplantation of kidney from cadaver nec	Renal disease
Kyu1400	[x]nephropathy induced by other drugs+biological substances	Renal disease
Q48y000	congenital renal failure	Renal disease
SP15411	kidney failure as a complication of care	Renal disease
K042.00	acute renal medullary necrosis	Renal disease
9Ot..00	chronic kidney disease monitoring administration	Renal disease
66i..00	chronic kidney disease monitoring	Renal disease
9Ot0.00	chronic kidney disease monitoring first letter	Renal disease
9Ot1.00	chronic kidney disease monitoring second letter	Renal disease
9Ot2.00	chronic kidney disease monitoring third letter	Renal disease
9Ot3.00	chronic kidney disease monitoring verbal invite	Renal disease
9Ot4.00	chronic kidney disease monitoring telephone invite	Renal disease
6AA..00	chronic kidney disease annual review	Renal disease
SP08N00	unexplained episode of renal transplant dysfunction	Renal disease
Kyu2.00	[x]renal failure	Renal disease
Kyu1E00	[x]tubulo-interstit nephritis, not specif as acute or chron	Renal disease
Kyu0900	[x]unsp nephrit synd, diff mesang prolif glomerulonephritis	Renal disease
K0B6.00	balkan nephropathy	Renal disease
K0A0400	ac neph syn difus endocaplry prolifrative glomerulonephritis	Renal disease
TA02000	accid cut,puncture,perf,h'ge - kidney dialysis	Renal disease
SP05613	[x] peritoneal dialysis associated peritonitis	Renal disease
D215.00	anaemia secondary to renal failure	Renal disease
K0A0300	acut neph syn, diffuse mesangial prolifrative glomnephritis	Renal disease
K0A0500	acute neph syn, diffuse mesangiocapillary glomerulonephritis	Renal disease
K0A0200	acute nephritic syn, diffuse membranous glomerulonephritis	Renal disease
K0A0100	acute nephritic syndrome, focal+segmental glomerular lesions	Renal disease
K0A0700	acute nephrotic syndrm diffuse crescentic glomerulonephritis	Renal disease
K041.00	acute renal cortical necrosis	Renal disease
ZV42000	[v]kidney transplanted	Renal disease
ZV45100	[v]renal dialysis status	Renal disease
ZV56.00	[v]aftercare involving intermittent dialysis	Renal disease
ZV56000	[v]aftercare involving extracorporeal dialysis	Renal disease
ZV56100	[v]preparatory care for dialysis	Renal disease
ZV56y00	[v]other specified aftercare involving intermittent dialysis	Renal disease
ZV56z00	[v]unspecified aftercare involving intermittent dialysis	Renal disease
ZVu3G00	[x]other dialysis	Renal disease
G22z.11	renal hypertension	Renal disease
7B00211	allotransplantation of kidney from cadaver	Renal disease
K08y500	acute interstitial nephritis	Renal disease
D215000	anaemia secondary to chronic renal failure	Renal disease
K0A0.00	acute nephritic syndrome	Renal disease
K05..11	chronic uraemia	Renal disease
K05..00	chronic renal failure	Renal disease
9Ni9.00	did not attend chronic kidney disease monitoring clinic	Renal disease
K0A3300	chron neph syn difus mesangial prolifrtiv glomerulonephritis	Renal disease
K0A3400	chron neph syn difuse endocap prolifrativ glomerulonephritis	Renal disease

K0A3200	chron nephritic syndrom difuse membranous glomerulonephritis	Renal disease
K02y000	chronic glomerulonephritis + diseases ec	Renal disease
K022.00	chronic membranoproliferative glomerulonephritis	Renal disease
K021.00	chronic membranous glomerulonephritis	Renal disease
K0A3500	chronic neph syn difus mesangiocapillary glomerulonephritis	Renal disease
K0A3700	chronic nephritic syn diffuse crescentic glomerulonephritis	Renal disease
K0A3100	chronic nephritic syndrm focal+segmental glomerular lesions	Renal disease
K020.00	chronic proliferative glomerulonephritis	Renal disease
1Z15.00	chronic kidney disease stage 3a	Renal disease
1Z16.00	chronic kidney disease stage 3b	Renal disease
K100400	nonobstructive reflux-associated chronic pyelonephritis	Renal disease
1Z17.00	chronic kidney disease stage 1 with proteinuria	Renal disease
1Z18.00	chronic kidney disease stage 1 without proteinuria	Renal disease
1Z19.00	chronic kidney disease stage 2 with proteinuria	Renal disease
1Z1A.00	chronic kidney disease stage 2 without proteinuria	Renal disease
1Z1B.00	chronic kidney disease stage 3 with proteinuria	Renal disease
1Z1C.00	chronic kidney disease stage 3 without proteinuria	Renal disease
1Z1D.00	chronic kidney disease stage 3a with proteinuria	Renal disease
1Z1E.00	chronic kidney disease stage 3a without proteinuria	Renal disease
1Z1F.00	chronic kidney disease stage 3b with proteinuria	Renal disease
1Z1G.00	chronic kidney disease stage 3b without proteinuria	Renal disease
1Z1H.00	chronic kidney disease stage 4 with proteinuria	Renal disease
1Z1J.00	chronic kidney disease stage 4 without proteinuria	Renal disease
1Z1K.00	chronic kidney disease stage 5 with proteinuria	Renal disease
1Z1L.00	chronic kidney disease stage 5 without proteinuria	Renal disease
K080100	renal dwarfism	Renal disease
1Z17.11	ckd stage 1 with proteinuria	Renal disease
1Z19.11	ckd stage 2 with proteinuria	Renal disease
1Z1A.11	ckd stage 2 without proteinuria	Renal disease
1Z1B.11	ckd stage 3 with proteinuria	Renal disease
1Z1C.11	ckd stage 3 without proteinuria	Renal disease
1Z1D.11	ckd stage 3a with proteinuria	Renal disease
1Z1E.11	ckd stage 3a without proteinuria	Renal disease
1Z1F.11	ckd stage 3b with proteinuria	Renal disease
1Z1G.11	ckd stage 3b without proteinuria	Renal disease
1Z1H.11	ckd stage 4 with proteinuria	Renal disease
1Z1J.11	ckd stage 4 without proteinuria	Renal disease
1Z1K.11	ckd stage 5 with proteinuria	Renal disease
1Z1L.11	ckd stage 5 without proteinuria	Renal disease
7L1A.11	dialysis for renal failure	Renal disease
7A61900	ligation of arteriovenous dialysis fistula	Renal disease
7A61A00	ligation of arteriovenous dialysis graft	Renal disease
K05..12	end stage renal failure	Renal disease
K0D..00	end-stage renal disease	Renal disease
G22..00	hypertensive renal disease	Renal disease
K104.00	xanthogranulomatous pyelonephritis	Renal disease

K032400	familial glomerulonephritis in alport's syndrome	Renal disease
K017.00	nephrotic syn difus mesangial prolifertiv glomerulonephritis	Renal disease
K019.00	nephrotic syn,diffuse mesangiocapillary glomerulonephritis	Renal disease
K018.00	nephrotic syn,difus endocapilary proliftv glomerulonephritis	Renal disease
K012.00	nephrotic syndrome+membranoproliferative glomerulonephritis	Renal disease
K02..11	nephritis - chronic	Renal disease
K03..00	nephritis and nephropathy unspecified	Renal disease
K032z00	nephritis unsp+membranoprolif glomerulonephritis lesion nos	Renal disease
K032y00	nephritis unsp+os membranoprolif glomerulonephritis lesion	Renal disease
K02..12	nephropathy - chronic	Renal disease
K0C1.00	nephropathy induced by other drugs meds and biologl substncs	Renal disease
K0C2.00	nephropathy induced by unspec drug medicament or biol subs	Renal disease
K01w000	finnish nephrosis syndrome	Renal disease
K06..11	uraemia nos	Renal disease
K0A4X00	isolated proteinuria, with oth specif morpholog changes	Renal disease
K0A4W00	isolated proteinuria, with unspecified morpholog changes	Renal disease
K013.11	lipoid nephrosis	Renal disease
TB00100	kidney transplant with complication, without blame	Renal disease
TB11.00	kidney dialysis with complication, without blame	Renal disease
K032200	focal glomerulon + focal recurr macroscop glomerulonephritis	Renal disease
F447C00	iridodialysis	Renal disease
7L1B000	insertion of ambulatory peritoneal dialysis catheter	Renal disease
K03V.00	unspecified nephritic syndrome, dense deposit disease	Renal disease
K03W.00	unsp nephrit synd, diff endocap prolif glomerulonephritis	Renal disease
K03X.00	unsp nephrit synd, diff mesang prolif glomerulonephritis	Renal disease
K01w.00	congenital nephrotic syndrome	Renal disease
K03U.00	unspecif nephr synd, diff concentric glomerulonephritis	Renal disease
K0A5000	hereditary nephropathy nec, minor glomerular abnormality	Renal disease
K0A5100	hereditary nephropathy nec,focal+segmnt glomerular lesion	Renal disease
K0A5600	hereditary nephropathy, nec, dense deposit disease	Renal disease
K0A5X00	hereditary nephropathy, unspecif morphological changes	Renal disease
K0A5200	hereditry nephropathy nec,difus membran glomerulnephritis	Renal disease
K0A5300	heredtry nephrpthy nec difus mesangial prolif glomnephrit	Renal disease
K03T.00	tubulo-interstit nephritis, not specif as acute or chron	Renal disease
K01..00	nephrotic syndrome	Renal disease
5932E	renal failure	Renal disease
7B00200	transplantation of kidney from cadaver	Renal disease
K08y000	hypokalaemic nephropathy	Renal disease
K0C0.00	analgesic nephropathy	Renal disease
Z1A..00	dialysis training	Renal disease
Z1A1.00	peritoneal dialysis training	Renal disease
5932EC	chronic renal failure	Renal disease
Z919.00	care of haemodialysis equipment	Renal disease
Z919300	reversing haemodialysis lines	Renal disease
Z91A.00	peritoneal dialysis bag procedure	Renal disease
Z919100	priming haemodialysis lines	Renal disease

Z1A2.00	haemodialysis training	Renal disease
N20..00	polymyalgia rheumatica	Rheumatic and collagen disease
N000300	systemic lupus erythematosus with organ or sys involv	Rheumatic and collagen disease
N040.00	rheumatoid arthritis	Rheumatic and collagen disease
N043200	pauciarticular juvenile rheumatoid arthritis	Rheumatic and collagen disease
N043100	acute polyarticular juvenile rheumatoid arthritis	Rheumatic and collagen disease
C37z.11	marinesco-sjogren syndrome	Rheumatic and collagen disease
N044.11	jaccoud's syndrome	Rheumatic and collagen disease
N002.00	sicca (sjogren's) syndrome	Rheumatic and collagen disease
N003.11	poikilodermatomyositis	Rheumatic and collagen disease
N001.12	systemic sclerosis	Rheumatic and collagen disease
N001000	progressive systemic sclerosis	Rheumatic and collagen disease
N04X.00	seropositive rheumatoid arthritis, unspecified	Rheumatic and collagen disease
N040A00	rheumatoid arthritis of dip joint of finger	Rheumatic and collagen disease
N040600	rheumatoid arthritis of distal radio-ulnar joint	Rheumatic and collagen disease
N040M00	rheumatoid arthritis of ip joint of toe	Rheumatic and collagen disease
N040L00	rheumatoid arthritis of lesser mtp joint	Rheumatic and collagen disease
N040800	rheumatoid arthritis of mcp joint	Rheumatic and collagen disease
N040900	rheumatoid arthritis of pip joint of finger	Rheumatic and collagen disease
N040C00	rheumatoid arthritis of sacro-iliac joint	Rheumatic and collagen disease
N040E00	rheumatoid arthritis of tibio-fibular joint	Rheumatic and collagen disease
N042z00	rheumatoid arthropathy + visceral/systemic involvement nos	Rheumatic and collagen disease
H570.00	rheumatoid lung	Rheumatic and collagen disease
N040R00	rheumatoid nodule	Rheumatic and collagen disease
N100.00	ankylosing spondylitis	Rheumatic and collagen disease
N04..00	rheumatoid arthritis and other inflammatory polyarthropathy	Rheumatic and collagen disease
N040K00	rheumatoid arthritis of 1st mtp joint	Rheumatic and collagen disease
N042.00	other rheumatoid arthropathy + visceral/systemic involvement	Rheumatic and collagen disease
N04y300	remitting seronegative symmetrical synovitis pitting oedema	Rheumatic and collagen disease
N003.00	dermatomyositis	Rheumatic and collagen disease
N042100	rheumatoid lung disease	Rheumatic and collagen disease
N040N00	rheumatoid vasculitis	Rheumatic and collagen disease
N003000	juvenile dermatomyositis	Rheumatic and collagen disease
N001.11	acrosclerosis	Rheumatic and collagen disease
9hR..00	exception reporting: rheumatoid arthritis quality indicators	Rheumatic and collagen disease
9hR0.00	except rheumatoid arthritis quality indicator: pt unsuitable	Rheumatic and collagen disease
9hR1.00	except rheumatoid arthritis qual indicator: informed dissent	Rheumatic and collagen disease
N04y011	caplan's syndrome	Rheumatic and collagen disease
N04y000	rheumatoid lung	Rheumatic and collagen disease
N101.00	spinal enthesopathy	Rheumatic and collagen disease
N04y111	sero negative polyarthritis	Rheumatic and collagen disease
66H..13	rheumatoid arthrit. monitoring	Rheumatic and collagen disease
66HB000	rheumatoid arthritis annual review	Rheumatic and collagen disease
PH12.11	sjogren - larsson syndrome	Rheumatic and collagen disease
9mM..00	rheumatoid arthritis monitoring invitation	Rheumatic and collagen disease
9mM0.00	rheumatoid arthritis monitoring invitation first letter	Rheumatic and collagen disease

9mM1.00	rheumatoid arthritis monitoring invitation second letter	Rheumatic and collagen disease
9mM2.00	rheumatoid arthritis monitoring invitation third letter	Rheumatic and collagen disease
9mM3.00	rheumatoid arthritis monitoring verbal invitation	Rheumatic and collagen disease
9mM4.00	rheumatoid arthritis monitoring telephone invitation	Rheumatic and collagen disease
N045500	juvenile rheumatoid arthritis	Rheumatic and collagen disease
N045100	juvenile seronegative polyarthritis	Rheumatic and collagen disease
14G1.00	h/o: rheumatoid arthritis	Rheumatic and collagen disease
N04..11	inflammatory polyarthropathy	Rheumatic and collagen disease
388p.00	basdai - bath ankylosing spondylitis disease activity index	Rheumatic and collagen disease
38DZ000	disease activity score 28 joint in rheumatoid arthritis	Rheumatic and collagen disease
38DZ.00	disease activity score in rheumatoid arthritis	Rheumatic and collagen disease
F371000	polyneuropathy in disseminated lupus erythematosus	Rheumatic and collagen disease
F371200	polyneuropathy in rheumatoid arthritis	Rheumatic and collagen disease
F396100	myopathy due to disseminated lupus erythematosus	Rheumatic and collagen disease
F396400	myopathy due to rheumatoid arthritis	Rheumatic and collagen disease
F396600	myopathy due to scleroderma	Rheumatic and collagen disease
F396700	myopathy due to sjogren's disease	Rheumatic and collagen disease
F4A3200	keratoconjunctivitis sicca (excluding sjogren's syndrome)	Rheumatic and collagen disease
G5y8.00	rheumatoid myocarditis	Rheumatic and collagen disease
G759.00	juvenile polyarteritis	Rheumatic and collagen disease
H121500	pharyngitis sicca	Rheumatic and collagen disease
H572.00	lung disease with systemic sclerosis	Rheumatic and collagen disease
H57y300	lung disease with sjogren's disease	Rheumatic and collagen disease
H57y400	lung disease with systemic lupus erythematosus	Rheumatic and collagen disease
M210000	unspecified circumscribed scleroderma	Rheumatic and collagen disease
M210.00	circumscribed scleroderma	Rheumatic and collagen disease
M210.11	addison's keloid	Rheumatic and collagen disease
M210z00	circumscribed scleroderma nos	Rheumatic and collagen disease
N000200	drug-induced systemic lupus erythematosus	Rheumatic and collagen disease
N000z00	systemic lupus erythematosus nos	Rheumatic and collagen disease
N001.00	scleroderma	Rheumatic and collagen disease
N001200	systemic sclerosis induced by drugs and chemicals	Rheumatic and collagen disease
N00z.00	collagen disease nos	Rheumatic and collagen disease
N040000	rheumatoid arthritis of cervical spine	Rheumatic and collagen disease
N040100	other rheumatoid arthritis of spine	Rheumatic and collagen disease
N040200	rheumatoid arthritis of shoulder	Rheumatic and collagen disease
N040300	rheumatoid arthritis of sternoclavicular joint	Rheumatic and collagen disease
N040400	rheumatoid arthritis of acromioclavicular joint	Rheumatic and collagen disease
N040500	rheumatoid arthritis of elbow	Rheumatic and collagen disease
N040700	rheumatoid arthritis of wrist	Rheumatic and collagen disease
N040B00	rheumatoid arthritis of hip	Rheumatic and collagen disease
N040D00	rheumatoid arthritis of knee	Rheumatic and collagen disease
N040F00	rheumatoid arthritis of ankle	Rheumatic and collagen disease
N040G00	rheumatoid arthritis of subtalar joint	Rheumatic and collagen disease
N040H00	rheumatoid arthritis of talonavicular joint	Rheumatic and collagen disease
N040J00	rheumatoid arthritis of other tarsal joint	Rheumatic and collagen disease

N040Q00	rheumatoid bursitis	Rheumatic and collagen disease
N040T00	flare of rheumatoid arthritis	Rheumatic and collagen disease
N043000	juvenile rheumatoid arthropathy unspecified	Rheumatic and collagen disease
N043300	monarticular juvenile rheumatoid arthritis	Rheumatic and collagen disease
N043z00	juvenile rheumatoid arthritis nos	Rheumatic and collagen disease
N045.00	other juvenile arthritis	Rheumatic and collagen disease
N04y.00	other specified inflammatory polyarthropathy	Rheumatic and collagen disease
N04yz00	other specified inflammatory polyarthropathy nos	Rheumatic and collagen disease
N04z.00	inflammatory polyarthropathy nos	Rheumatic and collagen disease
N10..00	inflammatory spondylopathies	Rheumatic and collagen disease
N102.00	sacroiliitis nec	Rheumatic and collagen disease
N10y.00	other inflammatory spondylopathies	Rheumatic and collagen disease
N10y000	inflammatory spondylopathies in diseases ec	Rheumatic and collagen disease
N10yz00	other inflammatory spondylopathies nos	Rheumatic and collagen disease
N240.00	rheumatism and fibrositis unspecified	Rheumatic and collagen disease
N240000	rheumatism unspecified	Rheumatic and collagen disease
N240200	muscular rheumatism	Rheumatic and collagen disease
N240700	hand rheumatism	Rheumatic and collagen disease
N240z00	rheumatism or fibrositis nos	Rheumatic and collagen disease
N2y..00	other specified nonarticular rheumatism	Rheumatic and collagen disease
N2z..00	nonarticular rheumatism nos	Rheumatic and collagen disease
Nyu1.00	[x]inflammatory polyarthropathies	Rheumatic and collagen disease
Nyu1100	[x]other seropositive rheumatoid arthritis	Rheumatic and collagen disease
Nyu1200	[x]other specified rheumatoid arthritis	Rheumatic and collagen disease
Nyu1G00	[x]seropositive rheumatoid arthritis, unspecified	Rheumatic and collagen disease
Nyu4300	[x]other forms of systemic lupus erythematosus	Rheumatic and collagen disease
Nyu4400	[x]other dermatomyositis	Rheumatic and collagen disease
Nyu4500	[x]other forms of systemic sclerosis	Rheumatic and collagen disease
Nyu4E00	[x]dermatopolymyositis, unspecified	Rheumatic and collagen disease
Nyu6.00	[x]spondylopathies	Rheumatic and collagen disease
Nyu6100	[x]other specified inflammatory spondylopathies	Rheumatic and collagen disease
Nyu6500	[x]other specified spondylopathies	Rheumatic and collagen disease
K0J0.00	renal involvement in scleroderma	Rheumatic and collagen disease
K0H..00	acute scleroderma renal crisis	Rheumatic and collagen disease
N040P00	seronegative rheumatoid arthritis	Rheumatic and collagen disease
N045000	juvenile ankylosing spondylitis	Rheumatic and collagen disease
N00y.11	fibrosclerosis - systemic	Rheumatic and collagen disease
N003100	dermatopolymyositis in neoplastic disease	Rheumatic and collagen disease
N200.00	giant cell arteritis with polymyalgia rheumatica	Rheumatic and collagen disease
7P20300	delivery of rehabilitation for rheumatoid arthritis	Rheumatic and collagen disease
N060.11	endemic polyarthritis	Rheumatic and collagen disease
M210400	linear scleroderma	Rheumatic and collagen disease
N042000	rheumatic carditis	Rheumatic and collagen disease
N00y.00	other specified diffuse collagen diseases	Rheumatic and collagen disease
Nyu1000	[x]rheumatoid arthritis+involvement/other organs or systems	Rheumatic and collagen disease
N040S00	rheumatoid arthritis - multiple joint	Rheumatic and collagen disease

N002.11	keratoconjunctivitis sicca	Rheumatic and collagen disease
N047.00	seropositive errosive rheumatoid arthritis	Rheumatic and collagen disease
N000400	systemic lupus erythematosus with pericarditis	Rheumatic and collagen disease
N005.00	adult still's disease	Rheumatic and collagen disease
N04y200	adult-onset still's disease	Rheumatic and collagen disease
G5yA.00	rheumatoid carditis	Rheumatic and collagen disease
N042200	rheumatoid nodule	Rheumatic and collagen disease
N000100	libman-sacks disease	Rheumatic and collagen disease
N045600	pauciarticular onset juvenile chronic arthritis	Rheumatic and collagen disease
N004.00	polymyositis	Rheumatic and collagen disease
N001100	crest syndrome	Rheumatic and collagen disease
N044.00	chronic post-rheumatic arthropathy	Rheumatic and collagen disease
N00..11	collagen diseases	Rheumatic and collagen disease
N003X00	dermatopolymyositis, unspecified	Rheumatic and collagen disease
N044.12	nodular fibrositis of chronic rheumatic disease	Rheumatic and collagen disease
K01x400	nephrotic syndrome in systemic lupus erythematosus	Rheumatic and collagen disease
N231400	polymyositis ossificans	Rheumatic and collagen disease
N043.00	juvenile rheumatoid arthritis - still's disease	Rheumatic and collagen disease
F37X.00	inflammatory polyneuropathy, unspecified	Rheumatic and collagen disease
N000.00	systemic lupus erythematosus	Rheumatic and collagen disease
N000000	disseminated lupus erythematosus	Rheumatic and collagen disease
N041.00	felty's syndrome	Rheumatic and collagen disease
H160400	laryngitis sicca	Rheumatic and collagen disease
ZRq8.11	slam - systemic lupus activity measure	Rheumatic and collagen disease
7179PR	polymyalgia rheumatica	Rheumatic and collagen disease
7161	polymyositis	Rheumatic and collagen disease
7340A	scleroderma	Rheumatic and collagen disease
ZRq9.00	systemic lupus erythematosus disease activity index	Rheumatic and collagen disease
ZRq8.00	systemic lupus activity measure	Rheumatic and collagen disease
7340BA	acrosclerosis	Rheumatic and collagen disease
6954	systemic lupus erythematosus	Rheumatic and collagen disease
7123	rheumatoid arthritis	Rheumatic and collagen disease
7919y00	therapeutic transluminal operation on heart valve os	Valvular disease
7919.00	therapeutic transluminal operations on valve of heart	Valvular disease
A932.00	syphilitic endocarditis	Valvular disease
G541600	aortic valve sclerosis	Valvular disease
P722400	supra-valvular aortic stenosis	Valvular disease
G120.00	rheumatic aortic stenosis	Valvular disease
P641.00	bicuspid aortic valve	Valvular disease
G140412	tricuspid incompetence, cause unspecified	Valvular disease
G140413	tricuspid regurgitation, cause unspecified	Valvular disease
G140514	tricuspid stenosis and regurgitation, cause unspecified	Valvular disease
G541212	aortic regurgitation alone, cause unspecified	Valvular disease
G543215	pulmonary regurgitation, cause unspecified	Valvular disease
G54z014	insufficiency of unspecified heart valve	Valvular disease
G54z013	regurgitation of unspecified heart valve	Valvular disease

G111.11	mitral incompetence - rheumatic	Valvular disease
P611.00	congenital tricuspid stenosis	Valvular disease
G541500	aortic stenosis	Valvular disease
G140112	tricuspid incompetence - rheumatic	Valvular disease
G140100	rheumatic tricuspid insufficiency	Valvular disease
G110.00	mitral stenosis	Valvular disease
P651.00	fused commissure of the mitral valve	Valvular disease
7914212	starr prosthetic replacement of valve of heart	Valvular disease
P65..00	congenital mitral stenosis	Valvular disease
Gy0..00	cardiovascular syphilis	Valvular disease
G110.11	rheumatic mitral stenosis	Valvular disease
7913.12	replacement of pulmonary valve	Valvular disease
7910.11	mitral valvuloplasty	Valvular disease
G141200	rheumatic pulmonary stenosis and insufficiency	Valvular disease
7919500	percutaneous transluminal pulmonary valve perfor dilation	Valvular disease
791D.00	excision of valve of heart	Valvular disease
7N40400	[so]truncal valve	Valvular disease
G114.00	ruptured mitral valve cusp	Valvular disease
G141000	rheumatic pulmonary stenosis	Valvular disease
G140000	rheumatic tricuspid stenosis	Valvular disease
G14z.11	rheumatic valvulitis, chronic nos	Valvular disease
G54z500	valvular heart disease	Valvular disease
7911500	transapical aortic valve implantation	Valvular disease
7911600	transluminal aortic valve implantation	Valvular disease
791A300	repair of supraaortic stenosis	Valvular disease
791A.00	remove obstruction from structure adjacent to valve of heart	Valvular disease
791Ay00	removal of obstruction from struct adjacent heart valve os	Valvular disease
G543300	pulmonary stenosis, cause unspecified	Valvular disease
P60..00	pulmonary valve anomalies	Valvular disease
G543.00	pulmonary valve disorders	Valvular disease
7911200	prosthetic replacement of aortic valve	Valvular disease
7910200	prosthetic replacement of mitral valve	Valvular disease
7913200	prosthetic replacement of pulmonary valve	Valvular disease
7912200	prosthetic replacement of tricuspid valve	Valvular disease
G12..00	rheumatic aortic valve disease	Valvular disease
G11..00	mitral valve diseases	Valvular disease
G540200	mitral valve prolapse	Valvular disease
P652.00	parachute deformity of the mitral valve	Valvular disease
14S4.00	h/o: heart valve recipient	Valvular disease
14T3.00	h/o: artificial heart valve	Valvular disease
P60z000	congenital insufficiency of the pulmonary valve	Valvular disease
7912.12	tricuspid valvuloplasty	Valvular disease
24D5.00	o/e - aortic systolic murmur	Valvular disease
24D6.00	o/e - aortic diastolic murmur	Valvular disease
24D7.00	o/e -pulmonary systolic murmur	Valvular disease
24D8.00	o/e-pulmonary diastolic murmur	Valvular disease

24D9.00	o/e - tricuspid murmur	Valvular disease
7902500	repair of tetralogy of fallot with absent pulmonary valve	Valvular disease
790D700	replacement of valved cardiac conduit	Valvular disease
791C500	aortoventriculoplasty with pulmonary valve autograft	Valvular disease
790Dy00	other specified creation of valved cardiac conduit	Valvular disease
791..00	valves of heart and adjacent structures operations	Valvular disease
7910300	replacement of mitral valve nec	Valvular disease
7910400	mitral valvuloplasty nec	Valvular disease
7910y00	other specified plastic repair of mitral valve	Valvular disease
7910z00	plastic repair of mitral valve nos	Valvular disease
7911000	allograft replacement of aortic valve	Valvular disease
7911300	replacement of aortic valve nec	Valvular disease
7911y00	other specified plastic repair of aortic valve	Valvular disease
7911z00	plastic repair of aortic valve nos	Valvular disease
7912300	replacement of tricuspid valve nec	Valvular disease
7912500	tricuspid valvuloplasty nec	Valvular disease
7912y00	other specified plastic repair of tricuspid valve	Valvular disease
7912z00	plastic repair of tricuspid valve nos	Valvular disease
7913300	replacement of pulmonary valve nec	Valvular disease
7913y00	other specified plastic repair of pulmonary valve	Valvular disease
7913z00	plastic repair of pulmonary valve nos	Valvular disease
7914000	allograft replacement of valve of heart nec	Valvular disease
7914100	xenograft replacement of valve of heart nec	Valvular disease
7914300	replacement of valve of heart nec	Valvular disease
7914y00	other specified plastic repair of unspecified valve of heart	Valvular disease
7914z00	plastic repair of unspecified valve of heart nos	Valvular disease
7915.00	revision of plastic repair of valve of heart	Valvular disease
7915000	revision of plastic repair of mitral valve	Valvular disease
7915100	revision of plastic repair of aortic valve	Valvular disease
7915200	revision of plastic repair of tricuspid valve	Valvular disease
7915300	revision of plastic repair of pulmonary valve	Valvular disease
7915y00	other specified revision of plastic repair of valve of heart	Valvular disease
7915z00	revision of plastic repair of valve of heart nos	Valvular disease
7916.00	open incision of heart valve	Valvular disease
7916000	open mitral valvotomy	Valvular disease
7916200	open tricuspid valvotomy	Valvular disease
7916y00	other specified open specified incision of valve of heart	Valvular disease
7917.00	closed incision of heart valve	Valvular disease
7917y00	other specified closed incision of valve of heart	Valvular disease
7917z00	closed incision of valve of heart nos	Valvular disease
7918.00	other open operations on valve of heart	Valvular disease
7918000	annuloplasty of mitral valve	Valvular disease
7918200	annuloplasty of valve of heart nec	Valvular disease
7918300	excision of vegetations of valve of heart	Valvular disease
7918y00	other specified other open operation on valve of heart	Valvular disease
7918z00	other open operation on valve of heart nos	Valvular disease

7919000	percutaneous transluminal mitral valvotomy	Valvular disease
7919200	percutaneous transluminal tricuspid valvotomy	Valvular disease
7919z00	therapeutic transluminal operation on heart valve nos	Valvular disease
791A200	repair of subaortic stenosis	Valvular disease
791B.00	other operations on structure adjacent to valve of heart	Valvular disease
791z.00	heart valve and adjacent structures operations nos	Valvular disease
7931000	inspection of valve of heart	Valvular disease
7A65100	interposition of valve of vein	Valvular disease
7A65y00	other specified repair of valve of vein	Valvular disease
A932100	syphilitic endocarditis of mitral valve	Valvular disease
A932300	syphilitic endocarditis of tricuspid valve	Valvular disease
G542X00	nonrheumatic tricuspid valve disorder, unspecified	Valvular disease
G542200	nonrheumatic tricuspid valve stenosis with insufficiency	Valvular disease
G112.00	mitral stenosis with insufficiency	Valvular disease
G112.13	mitral stenosis with regurgitation	Valvular disease
G112.12	mitral stenosis with incompetence	Valvular disease
G11z.00	mitral valve disease nos	Valvular disease
G12z.00	rheumatic aortic valve disease nos	Valvular disease
G13..00	diseases of mitral and aortic valves	Valvular disease
G130.00	mitral and aortic stenosis	Valvular disease
G131.00	mitral stenosis and aortic insufficiency	Valvular disease
G131.14	mitral stenosis and aortic regurgitation	Valvular disease
G131.13	mitral stenosis and aortic incompetence	Valvular disease
G132.12	mitral incompetence and aortic stenosis	Valvular disease
G132.13	mitral regurgitation and aortic stenosis	Valvular disease
G132.00	mitral insufficiency and aortic stenosis	Valvular disease
G133.00	mitral and aortic incompetence	Valvular disease
G133.11	mitral and aortic insufficiency	Valvular disease
G133.12	mitral and aortic regurgitation	Valvular disease
G13y.00	multiple mitral and aortic valve involvement	Valvular disease
G13z.00	mitral and aortic valve disease nos	Valvular disease
G140.00	tricuspid valve disease nec	Valvular disease
G14021Y	rheumatic tricuspid stenosis and incompetence	Valvular disease
G14021X	rheumatic tricuspid stenosis and regurgitation	Valvular disease
G140200	rheumatic tricuspid stenosis and insufficiency	Valvular disease
G140300	tricuspid stenosis, cause unspecified	Valvular disease
G140400	tricuspid insufficiency, cause unspecified	Valvular disease
G140500	tricuspid stenosis and insufficiency, cause unspecified	Valvular disease
G140z00	rheumatic tricuspid valve disease nos	Valvular disease
G141z00	rheumatic pulmonary valve disease nos	Valvular disease
G540000	mitral incompetence, non-rheumatic	Valvular disease
G540100	mitral incompetence, cause unspecified	Valvular disease
G540z00	mitral valve disorders nos	Valvular disease
G541012	aortic regurgitation, non-rheumatic	Valvular disease
G541100	aortic stenosis, non-rheumatic	Valvular disease
G541300	aortic stenosis alone, cause unspecified	Valvular disease

G541400	aortic valve stenosis with insufficiency	Valvular disease
G541z00	aortic valve disorders nos	Valvular disease
G542.00	tricuspid valve disorders, non-rheumatic	Valvular disease
G542000	tricuspid incompetence, non-rheumatic	Valvular disease
G542011	tricuspid insufficiency, non-rheumatic	Valvular disease
G542012	tricuspid regurgitation, non-rheumatic	Valvular disease
G542100	tricuspid stenosis, non-rheumatic	Valvular disease
G542z00	tricuspid valve disorders nos	Valvular disease
G543012	pulmonary regurgitation, non-rheumatic	Valvular disease
G543100	pulmonary stenosis, non-rheumatic	Valvular disease
G543311	pulmonary stenosis, cause unspecified	Valvular disease
G543400	pulmonary valve stenosis with insufficiency	Valvular disease
G543z00	pulmonary valve disorders nos	Valvular disease
G544000	disorders of both aortic and tricuspid valves	Valvular disease
G544100	disorders of both mitral and tricuspid valves	Valvular disease
G544200	combined disorders of mitral, aortic and tricuspid valves	Valvular disease
G54z.00	endocarditis, valve unspecified	Valvular disease
G54z000	incompetence of unspecified heart valve	Valvular disease
G54z100	stenosis of unspecified heart valve	Valvular disease
G54z300	endocarditis, valve unspecified, os	Valvular disease
G54zz00	endocarditis, valve unspecified, nos	Valvular disease
G122.00	rheumatic aortic stenosis with insufficiency	Valvular disease
Gyu1000	[x]other mitral valve diseases	Valvular disease
Gyu1100	[x]other rheumatic aortic valve diseases	Valvular disease
Gyu1200	[x]other tricuspid valve diseases	Valvular disease
Gyu5600	[x]other aortic valve disorders	Valvular disease
Gyu5800	[x]other pulmonary valve disorders	Valvular disease
Gyu5A00	[x]aortic valve disorders in diseases classified elsewhere	Valvular disease
P600.00	pulmonary valve anomaly, unspecified	Valvular disease
P601.00	congenital atresia of the pulmonary valve	Valvular disease
P601000	hypoplasia of pulmonary valve	Valvular disease
P601z00	congenital atresia of pulmonary valve nos	Valvular disease
P602100	congenital fusion of pulmonary valve segment	Valvular disease
P602z00	congenital pulmonary stenosis nos	Valvular disease
P60z.00	other pulmonary valve anomalies	Valvular disease
P60zz00	other pulmonary valve anomaly nos	Valvular disease
P61..00	congenital tricuspid atresia and stenosis	Valvular disease
P61z.00	congenital tricuspid atresia or stenosis nos	Valvular disease
P640.00	congenital aortic valve insufficiency, unspecified	Valvular disease
P64z.00	congenital aortic valve insufficiency nos	Valvular disease
P650.00	congenital mitral stenosis, unspecified	Valvular disease
P65z.00	congenital mitral stenosis nos	Valvular disease
P6y0.00	subaortic stenosis	Valvular disease
P6yy700	atresia of heart valve nec	Valvular disease
P6yyC00	fusion of mitral valve cusps	Valvular disease
P6z0.00	unspecified anomaly of heart valve	Valvular disease

G141.00	rheumatic pulmonary valve disease	Valvular disease
Pyu2200	[x]other congenital malformations of pulmonary valve	Valvular disease
Pyu2G00	[x]congenital malformation of tricuspid valve, unspecified	Valvular disease
7N40100	[so]aortic valve	Valvular disease
SP00200	mechanical complication of heart valve prosthesis	Valvular disease
7N40000	[so]mitral valve	Valvular disease
7N40300	[so]pulmonary valve	Valvular disease
7N40200	[so]tricuspid valve	Valvular disease
7N40.00	[so]valve of heart	Valvular disease
7N40z00	[so]valve of heart nec	Valvular disease
7910212	bjork-shiley prosthetic replacement of mitral valve	Valvular disease
7918111	de vega tricuspid annuloplasty	Valvular disease
7918400	closure of tricuspid valve	Valvular disease
791D000	tricuspid valvectomy	Valvular disease
7918500	closure of pulmonary valve	Valvular disease
791D100	pulmonary valvectomy	Valvular disease
7914600	replacement of truncal valve	Valvular disease
A932.11	syphilitic valve disease	Valvular disease
7914500	truncal valve repair	Valvular disease
791B200	operations on mitral subvalvar apparatus	Valvular disease
791Dy00	other specified excision of valve of heart	Valvular disease
7919600	percutaneous transluminal pulmonary valve replacement	Valvular disease
SyuK611	[x] embolism from prosthetic heart valve	Valvular disease
Pyu2H00	[x]congenital malformation of aortic and mitral valves unsp	Valvular disease
G541700	aortic valve calcification	Valvular disease
G540300	mitral valve leaf prolapse	Valvular disease
24G..00	aortic valve gradient	Valvular disease
P711.13	preductal aortic stenosis	Valvular disease
P712.13	postductal aortic stenosis	Valvular disease
7910.00	plastic repair of mitral valve	Valvular disease
7911.00	plastic repair of aortic valve	Valvular disease
7912.00	plastic repair of tricuspid valve	Valvular disease
7913.00	plastic repair of pulmonary valve	Valvular disease
7914.00	plastic repair of unspecified valve of heart	Valvular disease
7914200	prosthetic replacement of valve of heart nec	Valvular disease
7916z00	open incision of valve of heart nos	Valvular disease
7918100	annuloplasty of tricuspid valve	Valvular disease
Gyu5D00	[x]multiple valve disorders/diseases ce	Valvular disease
7914.11	replacement of unspecified valve of heart	Valvular disease
24D..11	o/e - aortic murmur	Valvular disease
24D..15	o/e - pulmonary murmur	Valvular disease
Gyu5500	[x]other nonrheumatic mitral valve disorders	Valvular disease
A932200	syphilitic endocarditis of aortic valve	Valvular disease
7912.11	replacement of tricuspid valve	Valvular disease
7911.12	replacement of aortic valve	Valvular disease
ZV42200	[v]heart valve transplanted	Valvular disease

ZV43300	[v]has artificial heart valve	Valvular disease
ZV45H00	[v]presence of prosthetic heart valve	Valvular disease
ZVu6e00	[x]presence of other heart valve replacement	Valvular disease
7911411	aortic valve repair nec	Valvular disease
7912511	tricuspid valve repair nec	Valvular disease
7910411	mitral valve repair nec	Valvular disease
7913411	pulmonary valve repair nec	Valvular disease
7914411	repair of valve of heart nec	Valvular disease
7917200	closed tricuspid valvotomy	Valvular disease
7910000	allograft replacement of mitral valve	Valvular disease
7913000	allograft replacement of pulmonary valve	Valvular disease
7912000	allograft replacement of tricuspid valve	Valvular disease
G121.12	aortic regurgitation - rheumatic	Valvular disease
G541.00	aortic valve disorders	Valvular disease
7917000	closed mitral valvotomy	Valvular disease
P66..00	congenital mitral insufficiency	Valvular disease
G11..11	rheumatic mitral valve disease	Valvular disease
7910211	bjork-shiley prosthetic replacement of mitral valve	Valvular disease
G111.00	rheumatic mitral insufficiency	Valvular disease
A93..00	cardiovascular syphilis	Valvular disease
7910213	carpentier prosthetic replacement of mitral valve	Valvular disease
7911100	xenograft replacement of aortic valve	Valvular disease
7910100	xenograft replacement of mitral valve	Valvular disease
7913100	xenograft replacement of pulmonary valve	Valvular disease
7912100	xenograft replacement of tricuspid valve	Valvular disease
P64..00	congenital aortic valve insufficiency	Valvular disease
P63..00	congenital aortic valve stenosis	Valvular disease
P6W..00	congenital malformation of aortic and mitral valves unsp	Valvular disease
P6X..00	congenital malformation of tricuspid valve, unspecified	Valvular disease
P602.00	congenital pulmonary stenosis	Valvular disease
G113.00	nonrheumatic mitral valve stenosis	Valvular disease
P610.00	congenital tricuspid atresia	Valvular disease
7902000	correct fallot tetralogy- valved right ventr outflow conduit	Valvular disease
790D300	creation of valved conduit between left ventricle and aorta	Valvular disease
32A3.00	ecg: p mitrale	Valvular disease
7910214	edwards prosthetic replacement of mitral valve	Valvular disease
7914211	edwards prosthetic replacement of valve of heart	Valvular disease
G544X00	multiple valve disease, unspecified	Valvular disease
G544.00	multiple valve diseases	Valvular disease
G111.12	mitral regurgitation - rheumatic	Valvular disease
G540.15	mitral valve prolapse	Valvular disease
SP00400	infect and inflammatory reaction due to cardiac valve pros	Valvular disease
TB01200	implant of heart valve prosthesis + complication, no blame	Valvular disease
P6yy.11	hypoplastic aortic orifice or valve	Valvular disease
G540.14	mitral valve regurgitation	Valvular disease
G540.00	mitral valve incompetence	Valvular disease

G540.12	mitral valve insufficiency	Valvular disease
G540.16	mitral regurgitation	Valvular disease
G54..11	heart valve disorders - non rheumatic	Valvular disease
791y.00	heart valve or adjacent structures operations os	Valvular disease
G140111	tricuspid regurgitation - rheumatic	Valvular disease
7910.12	replacement of mitral valve	Valvular disease

**Additional EMIS codes**	**Term**	**Group**
JHCAS1	asthma causes daytime asthma symptoms less than weekly	Asthma
JHCAS2	asthma causes daytime symptoms more than weekly, less than daily	Asthma
JHCAS3	asthma causes daytime asthma symptoms daily	Asthma
JHCAS4	asthma causes night time symptoms less than 2 times per month	Asthma
JHCAS5	asthma causes night time symp more than 2 times a month,not wkly	Asthma
JHCAS6	asthma causes night time asthma symptoms weekly or more often	Asthma
JHCFR1	frequent night time asthma symptoms	Asthma
JHCIN17	infrequent asthma exacerbations	Asthma
JHCFR2	frequent asthma exacerbations	Asthma
EMISNQAS34	asthma monitoring in primary care	Asthma
EMISNQAS35	asthma monitoring in secondary care	Asthma
EMISNQDA36	date of asthma diagnosis	Asthma
EMISNQAS41	asthma clinical management plan no longer in place	Asthma
EMISNQNO108	no change in asthma management plan	Asthma
EMISNQAC840	access to online patient asthma education given	Asthma
EMISNQAC876	acute infective exacerbation of asthma	Asthma
ESCTAS6	asthma trigger - perfume	Asthma
EMISASC1	asthma stable < 3 months	Asthma
EMISASC2	asthma stable > 3 months	Asthma
EMISASC3	asthma control unsatisfactory	Asthma
HNG0606	[rfc] asthma	Asthma
EGTON2B	cause of death- malignant neoplasms	Cancer
EGTON2EGTON203	cause of death- carcinoma of oesophagus	Cancer
EMISNQMA24	malignant melanoma of the cilary body	Cancer
EMISNQMA25	malignant melanoma of choroid	Cancer
EMISNQMA27	malignant melanoma of iris	Cancer
EMISNQHA31	has cancer key worker	Cancer
EMISNQHE41	hereditary nonpolyposis colon cancer	Cancer
EMISNQLY4	lymphangiomatosis	Cancer
EMISNQCA85	carcinoid tumour of lung	Cancer
EMISNQPR69	primary malignant neoplasm of lung	Cancer
EMISNQSM10	small cell lung cancer	Cancer
EMISNQNO51	non-small cell lung cancer	Cancer
EMISNQSQ1	squamous cell carcinoma of lung	Cancer
EMISNQAD45	adenocarcinoma of lung	Cancer
EMISNQLA74	large cell carcinoma of lung	Cancer
EMISNQTU10	tumour stage finding	Cancer
EMISNQME40	metastasis stage finding	Cancer

EMISNQTU11	tumour stage t1a	Cancer
EMISNQTU12	tumour stage t1b	Cancer
EMISNQTU13	tumour stage t2a	Cancer
EMISNQTU14	tumour stage t2b	Cancer
EMISNQTU15	tumour stage t3a	Cancer
EMISNQTU16	tumour stage t3b	Cancer
EMISNQTU17	tumour stage t4	Cancer
EMISNQME41	metastasis stage m0	Cancer
EMISNQME42	metastasis stage m1	Cancer
EMISNQME43	metastasis stage m2	Cancer
EMISNQME44	metastasis stage m3	Cancer
EMISNQME45	metastasis stage m4	Cancer
EMISNQME46	metastasis stage m1a	Cancer
EMISNQME47	metastasis stage m1b	Cancer
EMISNQME48	metastasis stage m1c	Cancer
JHCMA19	malignant neoplasm of skin	Cancer
EMISNQCA974	cancer follow up - enhanced services administration	Cancer
EMISNQBR35	breast cancer follow up - enhanced services administration	Cancer
EMISNQPR149	prostate cancer follow up - enhanced services administration	Cancer
EMISNQRE382	referral by lung cancer nurse specialist	Cancer
EMISNQCA1045	cancer care plan given no longer in place	Cancer
EMISNQEX43	extended consultation for cancer	Cancer
EMISNQEX44	extended consultation for cancer declined	Cancer
EMISNQHI51	history of malignant tumour of pelvis	Cancer
EMISNQME101	[m]metaplastic melanoma	Cancer
EMISNQFI39	first definitive treatment for a new primary cancer	Cancer
EMISNQFI40	first treatment for metastatic disease following an unknown primary	Cancer
EMISNQSE135	second or subsequent treatment for a new primary cancer	Cancer
EMISNQSE136	second or subsequent treatment for metastatic disease following an unknown primary	Cancer
EMISNQTR67	treatment for a local recurrence of a primary cancer	Cancer
EMISNQTR68	treatment for a regional recurrence of cancer	Cancer
EMISNQTR69	treatment for a distant recurrence of cancer (metastatic disease)	Cancer
EMISNQTR70	treatment for relapse of primary cancer (second or subsequent)	Cancer
EMISNQTR71	treatment for progression of primary cancer (second or subsequent)	Cancer
EMISNQTR72	treatment for multiple recurrence of cancer (local and/or regional and/or distant)	Cancer
142D	h/o: malignant neoplasm of vulva	Cancer
142E	h/o: malignant neoplasm of cervix	Cancer
142F	h/o: malignant neoplasm of uterine body	Cancer
142G	h/o: malignant neoplasm of ovary	Cancer
8Hgg0	discharge from secondary care breast cancer service	Cancer
8Hgg1	discharge from secondary care colorectal cancer service	Cancer
8Hgg2	discharge from secondary care prostate cancer service	Cancer
G8614	lymphoedema following cancer	Cancer
G8615	lymphoedema following breast cancer	Cancer
EGTON299	non hodgkin lymphoma	Cancer

EGTON83	excision melanoma	Cancer
HNG0200	[rfc] cancer of the prostate	Cancer
HNG0201	[rfc] cancer of the rectum	Cancer
HNG0202	[rfc] cancer of the testes	Cancer
HNG0203	[rfc] cancer of the ovary	Cancer
HNG0204	[rfc] cancer of the uterus	Cancer
HNG0205	[rfc] cancer of the cervix	Cancer
HNG0207	[rfc] lung cancer	Cancer
HNG0208	[rfc] cancer of the larynx	Cancer
HNG0182	[rfc] neoplasm	Cancer
HNG0189	[rfc] bowel cancer	Cancer
HNG0190	[rfc] breast cancer	Cancer
HNG0191	[rfc] cancer of the thyroid	Cancer
HNG0193	[rfc] cancer of the stomach	Cancer
HNG0194	[rfc] liver cancer	Cancer
HNG0196	[rfc] cancer of the mouth	Cancer
HNG0199	[rfc] neoplasm	Cancer
HNG0600	[rfc] cancer	Cancer
HNGNQRF58	[rfc] malignant wound	Cancer
EMISNQCA31	cancer medication review	Cancer
EMISNQCA33	cancer review invite 1	Cancer
EMISNQRE38	recurrence of cancer confirmed	Cancer
ALLERGY2.3	adverse reaction to anti-arrhythmic drugs	Cardiac arrythmia
EMISNQST5	stopping implantable cardiac defib. (icd) discussed with patient	Cardiac arrythmia
EMISNQAT22	atrial fibrillation confirmed	Cardiac arrythmia
EMISNQAT24	atrial fibrillation clinical pathway protocol not followed	Cardiac arrythmia
EMISNQAT28	atrial fibrillation monitoring in primary care	Cardiac arrythmia
EMISNQAT29	atrial fibrillation monitoring in secondary care	Cardiac arrythmia
EMISNQAT30	atrial fibrillation follow-up	Cardiac arrythmia
ZV45W	implantable cardiac electronic device in situ	Cardiac arrythmia
EMISNQAF6	af screen using handheld ecg monitor with af detector abnormal	Cardiac arrythmia
EMISNQCA1097	cardiac pacing	Cardiac arrythmia
EMISNQEC15	ecg: corrected qt interval (qtc) abnormal	Cardiac arrythmia
EMISNQEC16	ecg: corrected qt interval (qtc) prolonged	Cardiac arrythmia
EMISNQCA1102	cardiac resynchronisation therapy defibrillator device in situ	Cardiac arrythmia
EMISNQ123	12 lead ecg abnormal	Cardiac arrythmia
ESCTAT2	atrioventricular conduction disorder	Cardiac arrythmia
G5739	atypical atrial flutter	Cardiac arrythmia
321C1	ecg: sinus tachycardia	Cardiac arrythmia
ESCTMA2	management of internal defibrillation	Cardiac arrythmia
^ESCTIN704652	inappropriate sinus tachycardia	Cardiac arrythmia
HNG0017	[rfc] heart block	Cardiac arrythmia
EMISCEX7	excessively rapid heart rate	Cardiac arrythmia
ESCTOC3	occlusive stroke	Cerebrovascular disease
EMISNQCE11	cerebral venous thrombosis	Cerebrovascular disease
EMISNQSU26	suspected transient ischaemic attack	Cerebrovascular disease

EMISNQSU28	suspected stroke	Cerebrovascular disease
EMISNQCE16	cerebrovascular accident care plan	Cerebrovascular disease
EMISNQTR28	transient ischaemic attack monitoring	Cerebrovascular disease
EMISNQCE29	cerebrovascular disease monitoring default	Cerebrovascular disease
TESTPCE1	cerebrovascular disease management	Cerebrovascular disease
EMISNQRE381	referral by stroke nurse specialist	Cerebrovascular disease
EMISNQTI19	tia clinical management plan no longer in place	Cerebrovascular disease
Q4114	intraventric (nontraumatic) haemorrhage grade 4 fet newborn	Cerebrovascular disease
EMISNQRE602	referral to stroke rehabilitation service	Cerebrovascular disease
EMISNQRE623	referral to community stroke service	Cerebrovascular disease
EMISNQDI251	discharge from community stroke service	Cerebrovascular disease
HNG0234	[rfc] stroke/cva	Cerebrovascular disease
HNG0235	[rfc] stroke	Cerebrovascular disease
HNG0237	[rfc] ischaemic attack	Cerebrovascular disease
HNG0602	[rfc] stroke	Cerebrovascular disease
HNGP003	[rfc] cva	Cerebrovascular disease
EMISNQST2	stroke monitoring invite 1	Cerebrovascular disease
EMISNQST3	stroke monitoring invite 2	Cerebrovascular disease
EMISNQST4	stroke monitoring invite 3	Cerebrovascular disease
EMISCPU4	purpura	Coagulopathy
EMISNQHE13	heart failure lifestyle plan commenced	Congestive heart failure
EMISNQHE14	heart failure information starter pack provided	Congestive heart failure
EMISNQHE15	heart failure monitoring - unstable symptoms	Congestive heart failure
EMISNQHE16	heart failure monitoring - specialist clinical needs	Congestive heart failure
EMISNQHE17	heart failure monitoring - social issues	Congestive heart failure
EMISNQHE18	heart failure monitoring - psychological issues	Congestive heart failure
EMISNQHE19	heart failure monitoring - multiple readmissions	Congestive heart failure
EMISNQHE20	heart failure monitoring - co-medications	Congestive heart failure
EMISNQHE21	heart failure monitoring - co-morbidities	Congestive heart failure
EMISNQHE22	heart failure monitoring - palliative care	Congestive heart failure
EGTON2G6A1	cause of death- congestive cardiac failure	Congestive heart failure
EMISNQHE42	heart failure resolved	Congestive heart failure
EMISNQEM10	emergency heart failure admission since last appointment	Congestive heart failure
EMISNQHE58	heart failure pathway protocol not followed	Congestive heart failure
EMISNQHE70	heart failure monitoring in primary care	Congestive heart failure
EMISNQHE71	heart failure monitoring in secondary care	Congestive heart failure
EMISNQHE72	heart failure monitoring default	Congestive heart failure
EMISNQSE142	severe left ventricular systolic dysfunction	Congestive heart failure
EMISHGT40	echocardiogram shows lvh	Congestive heart failure
EMISHGT41	echocardiography - poor lv function	Congestive heart failure
EMISNQDI3	diastolic dysfunction	Congestive heart failure
EMISNQCO295	copd gold group a	COPD
EMISNQCO296	copd gold group b	COPD
EMISNQCO297	copd gold group c	COPD
EMISNQCO298	copd gold group d	COPD
EMISNQCL15	clinical copd questionnaire	COPD

EMISNQCH102	chronic obstructive pulmonary disease confirmed	COPD
EMISNQSE79	seen in chronic obstructive pulmonary disease clinic	COPD
EMISNQCH158	chronic obstructive pulmonary disease monitoring in primary care	COPD
EMISNQCH159	chronic obstructive pulmonary disease monitoring secondary care	COPD
EMISNQCH160	chronic obstructive pulmonary disease severity	COPD
EMISNQCH161	chronic obstructive pulmonary disease monitoring default	COPD
EMISNQCH162	chronic obstructive pulmonary disease follow-up assessment	COPD
EMISNQSH45	shared care chronic obstructive pulmonary disease monitoring	COPD
EMISNQ1C1	1 copd exacerbation in past year	COPD
EMISNQ3C1	3+ copd exacerbations in past year	COPD
EMISNQ2C1	2 copd exacerbations in past year	COPD
EMISNQNO109	no copd exacerbations in past year	COPD
EMISNQAC842	access to online patient copd education given	COPD
EMISNQCO268	copd assessment test score - cough	COPD
EMISNQCO269	copd assessment test score - phlegm (mucus)	COPD
EMISNQCO270	copd assessment test score - chest tightness	COPD
EMISNQCO271	copd assessment test score - breathless walking up hill/stairs	COPD
EMISNQCO272	copd assessment test score - activity limitation at home	COPD
EMISNQCO273	copd assessment test score - confidence leaving home	COPD
EMISNQCO274	copd assessment test score - sleeping soundly	COPD
EMISNQCO275	copd assessment test score - energy	COPD
EMISNQSU104	suitable for step down change in copd management plan	COPD
EMISNQAC878	acute non-infective exacerbation of chronic obstructive pulmonary disease	COPD
ESCTAC8	acute infective exacerbation of chronic obstructive airways disease	COPD
EMISR4QCH1	chronic bronchitis, acute exac	COPD
HNGNQRF16	[rfc] chronic obstructive pulmonary disease (copd)	COPD
EMISNQCO1	copd medication review	COPD
EMISNQCO3	copd monitoring invite 1	COPD
HNG0130	[rfc] pernicious anaemia	Deficiency anaemia
HNG0131	[rfc] iron deficiency anaemia	Deficiency anaemia
ALLERGY6.1	adverse reaction to drugs used in diabetes	Diabetes
EMISNQRE56	referral for diabetic retinopathy screening	Diabetes
EMISNQIN21	ineligibility for diabetic eye screening	Diabetes
EMISNQIN23	ineligibility for diabetic eye screening: persistent dna	Diabetes
EMISNQIN24	ineligibility for diabetic eye screening: no contact details	Diabetes
EMISNQIN25	ineligibility for diab. eye screening: declined to participate	Diabetes
EMISNQIN26	ineligib for diab. eye screen: no longer categorised as diabetic	Diabetes
EMISNQIN28	ineligibility for diabetic eye screening: under ophthalmic care	Diabetes
EMISNQIN29	ineligibility for diabetic eye screening: deceased	Diabetes
EMISNQIN30	ineligibility for diabetic eye screening: moved away	Diabetes
EMISNQNO16	no evidence of diabetic peripheral neuropathy	Diabetes
EMISNQIN33	ineligibility for diabetic eye screening: physical disability	Diabetes
EMISNQIN35	ineligibility for diabetic eye screening: terminal illness	Diabetes
EMISNQDE12	declined referral to desmond diabet. structured educat programme	Diabetes
EMISNQDI36	diabetic sick day rules advice	Diabetes
EMISNQNE23	newly diagnosed diabetes	Diabetes

EMISNQDI38	diabetic dermopathy	Diabetes
EMISNQRE174	referred to diabetes service	Diabetes
EMISNQRE216	reason for referral: diabetes	Diabetes
EMISNQDI70	diabetic on non-insulin injectable	Diabetes
ESCTDI20	diabetic ketoacidosis without coma	Diabetes
EMISNQDI80	diabetic retinopathy screen done by optician	Diabetes
EMISNQDI94	diabetes mellitus high risk review	Diabetes
EMISNQDI96	diabetes mellitus confirmed	Diabetes
EMISNQHO54	home visit for diabetes monitoring	Diabetes
EMISNQDI101	diabetic short review	Diabetes
EMISNQDI106	diabetic foot screening invitation letter	Diabetes
EMISNQDI111	diabetic annual review by practice nurse	Diabetes
EMISNQRE335	reason for influenza vaccine - diabetes mellitus	Diabetes
EMISNQDI118	diabetes monitoring in primary care	Diabetes
EMISNQDI119	diabetes monitoring in secondary care	Diabetes
TESTPDI5	diabetes treatment type	Diabetes
EMISNQCA1013	care contact service: diabetes	Diabetes
EMISNQDI129	diabetic 3 month review	Diabetes
EMISNQPA326	participant in diabetes aerobic and resistance exercise study	Diabetes
EMISNQDI158	diabetes care planning test results sent to patient	Diabetes
EMISNQHO137	h/o: hypoglycaemic event in diabetes	Diabetes
EMISNQNO149	no h/o hypoglycaemic event in diabetes	Diabetes
EMISNQDI212	diabetic foot examination - no action required	Diabetes
9OLP	x-pert first steps diabetes self-management programme complt	Diabetes
L180B	pre-existing type 2 diabetes mellitus in pregnancy	Diabetes
EMISNQNE127	new diabetic foot ulcer	Diabetes
9NlP1	seen by diabetes specialist nurse	Diabetes
9NN90	under care of hospital-based diabetes specialist nurse	Diabetes
9NN91	under care of community-based diabetes specialist nurse	Diabetes
9N6v	referred by diabetes uk roadshow clinician	Diabetes
L180A	pre-existing type 1 diabetes mellitus in pregnancy	Diabetes
ESCTDI1	discharge by diabetic liaison nurse	Diabetes
EMISHGT48	diabetic - moderate control	Diabetes
EMISHGT200	diabetic foot risk assessment	Diabetes
EMISHGT46	emergency diabetic admission since last appointment	Diabetes
EMISQDI1	diabetic - gp eye check	Diabetes
EMISQDI2	diabetic - hospital eye check	Diabetes
EMISQLA1	laser treated diabetic retinopathy	Diabetes
EMISQLE11	left laser treated diabetic retinopathy	Diabetes
EMISQLE13	left advanced diabetic retinal disease	Diabetes
EMISQLE14	left diabetic foot active ulceration	Diabetes
EMISQLE15	left diabetic foot high risk	Diabetes
EMISQLE16	left diabetic foot moderate risk	Diabetes
EMISQLE17	left diabetic foot low current risk	Diabetes
EMISTO1	patient has been told has diabetes	Diabetes
EMISQLE8	left non-proliferative diabetic retinopathy	Diabetes

EMISQRI10	right proliferative diabetic retinopathy	Diabetes
EMISQRI11	right laser treated diabetic retinopathy	Diabetes
EMISQRI12	right diabetic maculopathy	Diabetes
EMISQRI13	right advanced diabetic retinal disease	Diabetes
EMISQRI14	right diabetic foot active ulceration	Diabetes
EMISQRI15	right diabetic foot high risk	Diabetes
EMISQRI16	right diabetic foot moderate risk	Diabetes
EMISQRI17	right diabetic foot low current risk	Diabetes
EMISQRI8	right non-proliferative diabetic retinopathy	Diabetes
EMISQRI9	right preproliferative diabetic retinopathy	Diabetes
HNG0036	[rfc] diabetes	Diabetes
HNG0167	[rfc] diabetic foot/leg ulcer	Diabetes
HNG0605	[rfc] diabetes mellitus	Diabetes
HNGNQRF66	[rfc] diabetic health promotion	Diabetes
HNGNQRF94	[rfc] diabetic leg ulcer	Diabetes
HNGNQRF95	[rfc] diabetic foot ulcer	Diabetes
PC0646	pt advised re diabetic diet	Diabetes
EMISNQSI2	six month diabetic review	Diabetes
EMISCEL1	electrolyte imbalance	Fluid and electrolyte disorders
EMISNQHO13	h/o: hiv	HIV/AIDS
H-SHHAPT	[shhapt] hiv positive	HIV/AIDS
H2-SHHAPT	[shhapt] attendance for hiv related care	HIV/AIDS
EMISNQHI56	hiv-1/hiv-2 antigen/antibody combination immunoassay positive	HIV/AIDS
E3A-KC60	aids - first presentation	HIV/AIDS
E3B-KC60	aids - subsequent presentation	HIV/AIDS
E1A-KC60	asymptomatic hiv infection - first presentation	HIV/AIDS
E1B-KC60	asymptomatic hiv infection - subsequent presentation	HIV/AIDS
E2A-KC60	hiv infection with symptoms, not aids - first presentation	HIV/AIDS
E2B-KC60	hiv infection with symptoms, not aids - subsequent presentation	HIV/AIDS
EGTON41	hiv infection	HIV/AIDS
HNG0143	[rfc] hiv/aids	HIV/AIDS
PCNQAI2	aids carrier	HIV/AIDS
ALLERGY2.5	adverse reaction to antihypertensive drugs	Hypertension
ALLERGY2.5.1	adverse reaction to vasodilator antihypertensive drugs	Hypertension
EMISNQST24	stage 1 hypertension	Hypertension
EMISNQSE73	severe hypertension	Hypertension
EMISNQHY12	hypertension confirmed	Hypertension
EMISNQHY15	hypertension monitoring in primary care	Hypertension
EMISNQHY16	hypertension monitoring in secondary care	Hypertension
EMISNQHY19	hypertension clinical management plan no longer in place	Hypertension
EMISHGT69	hypertension annual review	Hypertension
EGTONHY3	hypertensive clinic	Hypertension
HNGZ016	[rfc] hypertension	Hypertension
EMISNQHY2	hypertension medication review	Hypertension
EMISNQSE16	secondary hypothyroidism	Hypothyroidism
EMISNQHY13	hypothyroidism confirmed	Hypothyroidism

EMISNQHY20	hypothyroidism clinical management plan no longer in place	Hypothyroidism
EMISNQHY1	hypothyroidism medication review	Hypothyroidism
EMISNQHY4	hypothyroidism monitoring invite 1	Hypothyroidism
EMISNQHY5	hypothyroidism monitoring invite 2	Hypothyroidism
EMISNQHY6	hypothyroidism monitoring invite 3	Hypothyroidism
EMISNQDN3	dna - did not attend hypothyroidism clinic	Hypothyroidism
EMISNQHE29	hepatitis c pcr positive	Liver disease
EMISNQLI28	liver disease due to cystic fibrosis	Liver disease
C13-SHHAPT	[shhapt] viral hepatitis b (hbsag positive) - first diagnosis	Liver disease
C14-SHHAPT	[shhapt] viral hepatitis c - first diagnosis	Liver disease
C15-SHHAPT	[shhapt] viral hepatitis a - acute infection	Liver disease
EMISNQVI15	viral hepatitis d	Liver disease
EMISNQRE332	reason for influenza vaccine - chronic liver disease	Liver disease
EMISNQNO107	non-alcoholic fatty liver disease (nafld) fibrosis score	Liver disease
EMISNQHE176	hepatitis a total antibody test positive	Liver disease
EMISNQHE178	hepatitis b surface antibody test positive	Liver disease
C14-KC60	other viral hepatitis	Liver disease
C13-KC60	antigen positive viral hepatitis b	Liver disease
EMISHGT56	fetor hepatis	Liver disease
HNG0084	[rfc] liver failure	Liver disease
HNG0090	[rfc] cirrhosis	Liver disease
HNG0091	[rfc] hepatitis	Liver disease
PCNQVI1	viral hepatitis carrier	Liver disease
EMISNQHE6	hepatitis c carrier	Liver disease
EMISNQHO3	h/o: hepatitis b	Liver disease
EMISNQHE11	hepatitis c positive	Liver disease
EGTON2EGTON433	cause of death- myocardial infarction	Myocardial infarction
EGTON2G41	cause of death- acute myocardial infarction	Myocardial infarction
EMISNQNO74	no fh: myocardial infarction	Myocardial infarction
HNG0009	[rfc] myocardial infarction (mi)	Myocardial infarction
EMISR4QFI1	first myocardial infarction	Myocardial infarction
EMISNQCA49	cause of learning disabilities: congenital hydrocephalus	Neurological disorders
ALLERGY4.8	adverse reaction to antiepileptics	Neurological disorders
ALLERGY4.8.1	adverse reaction to drugs used in control of epilepsy	Neurological disorders
EMISNQEP4	epilepsy treatment continued	Neurological disorders
EMISNQHO38	honos-ld item 13 - seizures	Neurological disorders
EMISNQRE225	reason for referral: epilepsy	Neurological disorders
EMISNQRE248	reason for referral: parkinsons disease	Neurological disorders
EMISNQVA13	variant creutzfeldt-jakob disease	Neurological disorders
JHCHI32	history of creutzfeldt-jakob disease	Neurological disorders
JHCHI33	history of suspected creutzfeldt-jakob disease	Neurological disorders
EMISNQEP19	epilepsy monitoring administration	Neurological disorders
EMISNQEP20	epilepsy monitoring in primary care	Neurological disorders
EMISNQEP21	epilepsy monitoring in secondary care	Neurological disorders
EMISNQEP23	epilepsy treatment compliance good	Neurological disorders
EMISNQEP24	epilepsy treatment compliance poor	Neurological disorders

EMISNQRE380	referral by parkinsons disease nurse specialist	Neurological disorders
EMISNQRE383	referral by multiple sclerosis nurse specialist	Neurological disorders
JHCHO1	h/o: seizures	Neurological disorders
EMISNQSE120	seen by parkinson's service	Neurological disorders
EMISNQLI62	lindop parkinson's assessment scale - gait mobility score	Neurological disorders
EMISNQLI69	lindop parkinson's assessment scale - bed mobility score	Neurological disorders
38VT	amyotrophic lateral sclerosis functional rating scale - rvsd	Neurological disorders
EMISNQCO292	contraceptive advice for patients on valproate for epilepsy	Neurological disorders
94F1	sudep - sudden unexpected death in epilepsy	Neurological disorders
Q48H	acquired hydrocephalus of newborn	Neurological disorders
F1740	multiple system atrophy, cerebellar variant	Neurological disorders
F1741	multiple system atrophy, parkinson variant	Neurological disorders
EMISR4QPA1	partial epilepsy	Neurological disorders
EMISR4QSP2	spinal demyelination	Neurological disorders
HNG0054	[rfc] parkinson's disease	Neurological disorders
HNG0067	[rfc] epilepsy/convulsion	Neurological disorders
HNG0603	[rfc] multiple sclerosis	Neurological disorders
HNG0633	[rfc] parkinson's disease	Neurological disorders
PC0037	epilepsy monitoring	Neurological disorders
PC0676	initial epilepsy assessment	Neurological disorders
PC0677	follow-up epilepsy assessment	Neurological disorders
EMISCTR3	tremors/seizures	Neurological disorders
EMISNQAN3	annual stroke/cva blood test	Neurological disorders
EMISNQMU8	multiple sclerosis - personal health plan	Neurological disorders
EMISNQMU11	multiple sclerosis - primary progressive	Neurological disorders
EMISNQMU12	multiple sclerosis - secondary progressive	Neurological disorders
EMISNQMU13	multiple sclerosis - relapsing remitting	Neurological disorders
EMISNQRE209	reason for referral: cerebral palsy	Paralysis or paresis
JHCAC3	acute flaccid paralysis	Paralysis or paresis
EMISR4QSP1	spastic paraparesis	Paralysis or paresis
HNG0180	[rfc] cerebral palsy	Paralysis or paresis
HNG0197	[rfc] cerebral palsy	Paralysis or paresis
HNG0086	[rfc] peptic and duodenal ulcers	Peptic ulcer
EMISNQPE9	peripheral vascular disease annual review	Peripheral vascular disease
EMISNQPE10	peripheral vascular disease monitoring administration	Peripheral vascular disease
EMISNQPE11	peripheral vascular disease monitoring first letter	Peripheral vascular disease
EMISNQPE15	percutaneous embolisation of bronchial artery	Peripheral vascular disease
EMISNQIN59	infrarenal abdominal aortic aneurysm	Peripheral vascular disease
EMISNQPE38	peripheral arterial disease confirmed	Peripheral vascular disease
ESCTUP1	upper limb ischaemia	Peripheral vascular disease
G72yC	vertebral artery aneurysm	Peripheral vascular disease
HNG0015	[rfc] arterio-sclerosis	Peripheral vascular disease
HNG0025	[rfc] aneurysms	Peripheral vascular disease
HNG0028	[rfc] arterial embolism of limbs	Peripheral vascular disease
HNG0169	[rfc] arterial ulcer	Peripheral vascular disease
HNG0172	[rfc] peripheral vascular disease	Peripheral vascular disease

HNG0621	[rfc] leg ulcer - arterial	Peripheral vascular disease
HNG0622	[rfc] leg ulcer - both arterial & venous	Peripheral vascular disease
EMISNQAT9	at risk of chronic kidney disease	Renal disease
EMISNQCH107	chronic kidney disease diagnosis discussed with patient	Renal disease
EMISNQRE331	reason for influenza vaccine - chronic kidney disease	Renal disease
EMISNQPA337	patient kidney care card given	Renal disease
14D8	h/o: acute kidney injury	Renal disease
EMISNQCH956	chronic kidney disease six monthly review	Renal disease
8DE	dialysis therapy started by renal service	Renal disease
44yC1	peritoneal dialysis fluid adenosine deaminase level	Renal disease
EGTON129	non-functioning kidney	Renal disease
EGTON89	chronic kidney rejection	Renal disease
HNG0107	[rfc] continuous ambulatory peritoneal dialysis	Renal disease
HNG0111	[rfc] renal transplant	Renal disease
EMISNQCH17	chronic kidney disease stage	Renal disease
EMISNQCH21	chronic kidney disease stage 4	Renal disease
ALLERGY10.1	adverse reaction to drugs used in rheumatic diseases and gout	Rheumatic and collagen disease
ALLERGY10.1.3	adverse reaction to drugs which may affect the rheumatic disease process	Rheumatic and collagen disease
EMISNQSY6	systemic lupus erythematosus encephalitis	Rheumatic and collagen disease
EMISNQRE122	remitting seronegative symmetrical synovitis with pitting oedema	Rheumatic and collagen disease
EMISNQRH17	rheumatoid arthritis monitoring invitation	Rheumatic and collagen disease
EMISNQRH20	rheumatoid arthritis monitoring invitation first letter	Rheumatic and collagen disease
EMISNQRH21	rheumatoid arthritis monitoring invitation second letter	Rheumatic and collagen disease
EMISNQRH22	rheumatoid arthritis monitoring invitation third letter	Rheumatic and collagen disease
EMISNQRH23	rheumatoid arthritis resolved	Rheumatic and collagen disease
38Vs	rheumatoid arthritis impact of disease questionnaire	Rheumatic and collagen disease
EGTONSE2	sero-negative polyarthritis	Rheumatic and collagen disease
HNGP005	[rfc] rheumatoid arthritis	Rheumatic and collagen disease
EMISHGT105	aortic valve opening restricted	Valvular disease
HNG0024	[rfc] arterial stenosis	Valvular disease
